# Divergent reactivity of sulfinates with pyridinium salts based on one- *versus* two-electron pathways†

**DOI:** 10.1039/d1sc00776a

**Published:** 2021-03-31

**Authors:** Myojeong Kim, Euna You, Seongjin Park, Sungwoo Hong

**Affiliations:** Department of Chemistry, Korea Advanced Institute of Science and Technology (KAIST) Daejeon 34141 Korea hongorg@kaist.ac.kr; Center for Catalytic Hydrocarbon Functionalizations, Institute for Basic Science (IBS) Daejeon 34141 Korea

## Abstract

One of the main goals of modern synthesis is to develop distinct reaction pathways from identical starting materials for the efficient synthesis of diverse compounds. Herein, we disclose the unique divergent reactivity of the combination sets of pyridinium salts and sulfinates to achieve sulfonative pyridylation of alkenes and direct C4-sulfonylation of pyridines by controlling the one- *versus* two-electron reaction manifolds for the selective formation of each product. Base-catalyzed cross-coupling between sulfinates and *N*-amidopyridinium salts led to the direct introduction of a sulfonyl group into the C4 position of pyridines. Remarkably, the reactivity of this set of compounds is completely altered upon exposure to visible light: electron donor–acceptor complexes of *N*-amidopyridinium salts and sulfinates are formed to enable access to sulfonyl radicals. In this catalyst-free radical pathway, both sulfonyl and pyridyl groups could be incorporated into alkenes *via* a three-component reaction, which provides facile access to a variety of β-pyridyl alkyl sulfones. These two reactions are orthogonal and complementary, achieving a broad substrate scope in a late-stage fashion under mild reaction conditions.

## Abstract 2

Divergent reactions of sulfinates with pyridinium salts were developed by controlling the one- *versus* two-electron reaction manifolds.

## Graphical abstract

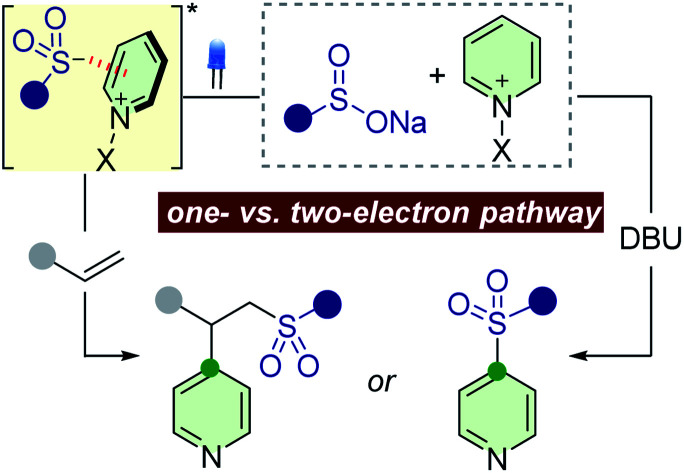

## Introduction

Sulfonyl groups are key functional motifs embedded in a myriad of biologically relevant molecules and functional materials, as their intrinsic properties can access unexplored chemical space by imparting hydrophilicity and biological affinity.^1^ They can also serve as versatile building blocks for further modification or as reagents in many chemical transformations.^2^ Accordingly, selective incorporation of sulfonyl moieties into organic commodities has received considerable attention in both chemistry and biology.^3^ Conventional approaches include Friedel–Craft type sulfonylation of arenes,^4^ oxidation of sulfides,^5^ and nucleophilic aromatic substitution (S_N_Ar).^6^ However, the practicality of these methods is largely limited by harsh conditions, such as high temperature and the need of stoichiometric acids and strong oxidants. For milder conditions, readily available sulfinates have emerged as practical and bench-stable reagents, and their synthetic potential has been widely demonstrated.^7^ For instance, substitution reactions of halogenated (hetero)arenes with sulfinates *via* transition-metal catalysis have provided efficient access to (hetero)aryl sulfones.^8,9^ In addition, the Willis group has reported a robust synthetic strategy to employ 1,4-diazabicyclo[2.2.2]octane-bis(sulfur dioxide) for incorporating sulfur dioxide, generating a diverse set of metal sulfinates to deliver a wide range of sulfone compounds.^8*d*–*f*,10^ The Larionov group reported a transition metal-free method, where sulfinate can serve as a nucleophile in a persulfate-initiated S_N_Ar-type process.^11^

Alternatively, the recent advent of a photocatalytic platform enables the utilization of sulfinates as convenient sulfonyl radical precursors^9,12^ toward the difunctionalization of π-systems.^13^ Taking advantage of dual photoredox and Ni catalysis, Nevado and colleagues developed a carbosulfonylation reaction of activated alkenes using aryl halides to furnish β-sulfonylated aryl products.^13*a*^ In addition, unconjugated dienes can also be applied to such transformations, as recently demonstrated by the Rueping group.^13*c*^ Despite significant advances within these catalytic manifolds, the majority of the known methods are still restricted to the use of (hetero)aryl halides as electrophilic coupling partners and exogenous catalysts. Therefore, the development of more sustainable strategies that diversify the utility of sulfinates would be highly demanding. Given the versatility of sulfinates, we wondered whether it would be possible to exploit their divergent reactivity with pyridinium salts by carefully adjusting the reaction conditions based on one- *vs.* two-electron reaction manifolds. However, the difficulty in discriminating the inherent reactivity of two distinct reaction pathways makes this strategy challenging, and the complete exclusion of the other reaction pathway is required. In this regard, our design strategy arose from the initial observation of almost no reactivity between sulfinates and pyridinium salts under catalyst-free conditions, and thus, their reactivity would depend entirely on the suitable activation process *via* either open-shell or polar reaction pathways.

**Scheme 1:**
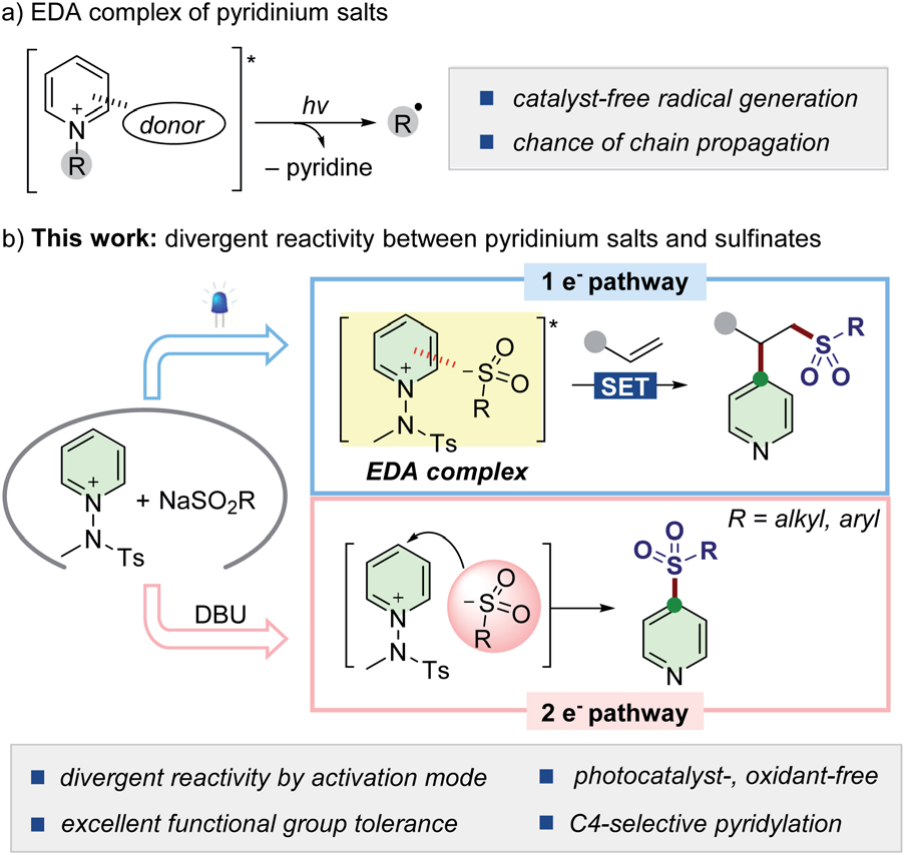
Divergent reactivity of sulfinates based on one- *versus* two-electron reaction manifolds.

Synthetic strategies involving photoactive electron donor–acceptor (EDA) complexes with certain anions have been widely explored as powerful tools to promote radical-mediated transformations by harvesting visible light (Scheme 1a).^14,15^ We envisioned that sulfinates could also serve as a new type of electron-donor for delivering such a radical-based reactivity with *N*-activated pyridinium salts.^16,17^ Combined with an alkene moiety, the generated sulfonyl radicals could be embraced in the three-component assembly process^18^ without the need of external photocatalysts and oxidants, which is preferable from the viewpoint of green chemistry. If successful, such a modular approach would enable the net addition of pyridyl and sulfonyl moieties across double bonds and further expand the synthetic horizon of sulfinates (Scheme 1b, top). As another mechanistic scenario, we postulated the possibility of direct insertion of sulfinates into pyridinium salts by exploiting them as electrophiles within a two-electron process^19,20^ to give C4-sulfonyl heteroarene derivatives (Scheme 1b, bottom).

In the course of our investigation, we discovered that the poor kinetic profile of sulfinates with *N*-amidopyridinium salts could be dramatically accelerated by adding a catalytic amount of a base, which allows an otherwise unfavorable addition pathway in this system. Herein, we disclose the divergent use of sulfinates and pyridinium salts to explore visible-light-driven sulfonative pyridylation of alkenes under photocatalyst-free conditions and C4-sulfonylation of N-heteroarenes. In our approach, a versatile platform provides distinct modes of reaction pathways to access a variety of value-added sulfones by controlling the one- *versus* two-electron reaction manifolds under mild conditions. Remarkably, all tested reactions displayed excellent C4-positional selectivity regarding the pyridine scaffolds.

## Results and discussion

**Fig. 1:**
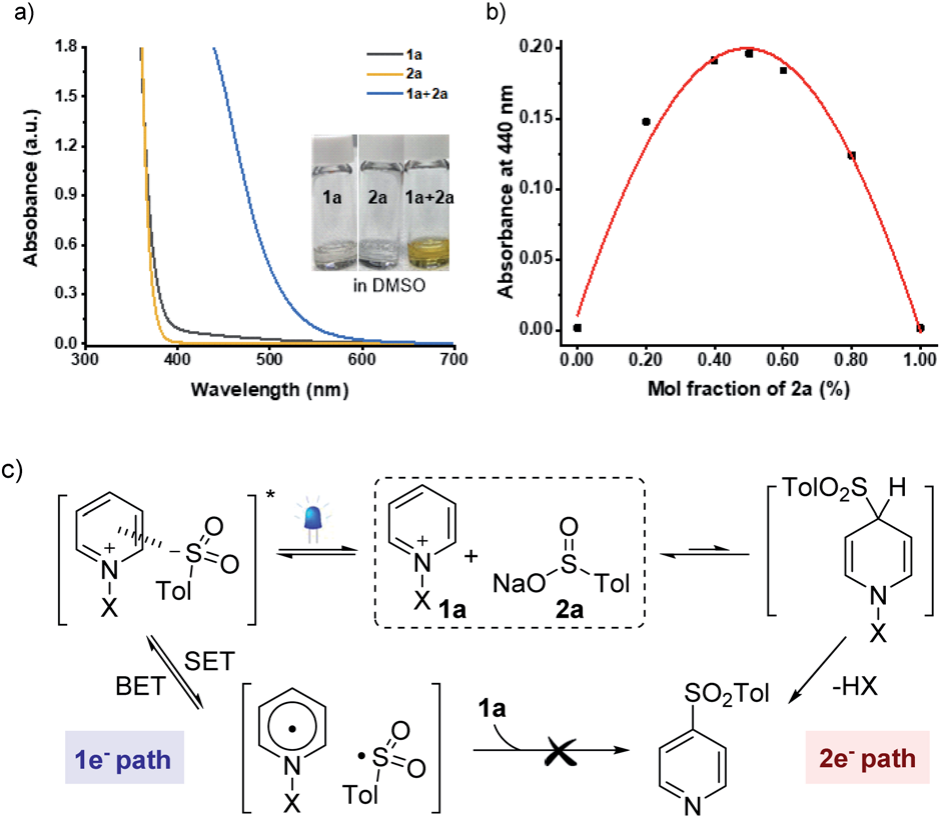
(a) UV-Vis absorption spectra of *N*-amidopyridinium salt (**1a**), sodium *p*-toluenesulfinate (**2a**), and the mixture of **1a** and **2a** (0.15 M in DMSO). (b) Job's plot for the ratio between **1a** and **2a**. (c) Two orthogonal resting states (X = NTs(Me)). SET = single-electron transfer. BET = back-electron transfer.

An essential objective of our approach was to identify the activation modes that would selectively operate one- *vs.* two-electron reaction manifolds. With this design in mind, we commenced our investigations into the ability to induce the association of sulfinate **2a** (*E*^ox^ = +0.32 V *vs.* SCE)^21^ with *N*-amidopyridinium salt **1a** (*E*^red^ = −0.82 V *vs.* SCE) (see the ESI†). Indeed, when both reagents were mixed, a redshift was observed by UV/vis absorption spectroscopy as a result of the formation of colored EDA complexes (Fig. 1a). Further analyses using Job's method of continuous variations exhibited that the donor–acceptor complex of **1a** and **2a** was formed in a 1 : 1 ratio of mole fractions (Fig. 1b). Given that sulfonyl radicals are generally considered to be electrophilic in nature,^22^ it was expected that their polarity-mismatched addition to electron-deficient heteroarenium salts would result in a poor reactivity between them. Indeed, we did not observe any productive reactivity between **1a** and **2a** under blue LED irradiation (see the ESI†). In this context, equilibrium between two orthogonal resting states provides the basis for controlling the divergent reaction pathways driven by different activation modes (Fig. 1c).

Based on the above-mentioned results, we first focused on the proposed three-component ‘one-electron pathway’ reaction mode, which enables access to a polarity-reversing strategy in a radical mechanism, as illustrated in Table 1 (see the ESI†). We exploited butyl vinyl ether (**3a**) to conjugate pyridyl and tosyl groups in a single step upon irradiation with blue LEDs. After extensive screening of parameters such as solvent, concentration, and temperature, the optimal reaction conditions were identified without the need for an external photocatalyst or additives. DMSO proved to be the optimal solvent for this protocol, with the reaction proceeding to 90% product yield (entry 1). The light source at longer wavelengths (525 nm, green LEDs) led to a reduced yield (entry 3). External photocatalysts did not offer any useful improvements (entries 4 and 5). Under neutral conditions, a two-component coupling product **5a** was not observed (entries 1–5). The use of NaOAc (1.2 equiv.) provided a comparable yield of 80% for **4a**, while compound **5a** was obtained with a 6% yield (entry 6). However, the reactivity dramatically changed with the use of DBU (20 mol%), regardless of light irradiation, resulting in the formation of **5a** as a major product (entries 7 and 8). Control experiments for the optimized conditions verified the necessity of light for the three-component reaction to proceed (entry 9). The reactivity was completely inhibited when the radical scavenger (2,2,6,6,-tetramethylpiperidin-1-yl)oxyl (TEMPO) was added (entry 10). The presence of oxygen decreased the reaction efficiency (entry 11).

**Table 1:** Optimization of the reaction conditions^a^

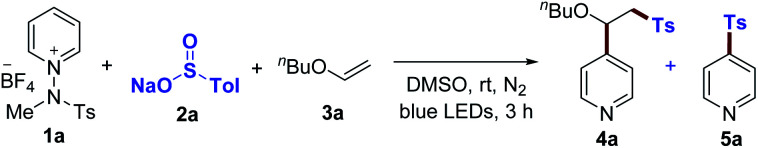			
Entry	Variations from standard conditions	Yield^b^ (%)	
		**4a**	**5a**
1	None	90	n.d.
2	DMF instead of DMSO	77	n.d.
3	Green LED (525 nm) instead of blue LED	31	n.d.
4	Using [Ir(dFCF_3_ppy)_2_(bpy)]PF_6_ (5 mol%)	65	n.d.
5	Using Eosin Y (2 mol%)	67	n.d.
6	Using NaOAc (1.2 equiv.) for 12 h	80	6
7^c^	Using DBU (20 mol%) for 12 h	<5	61
8^c^	Using DBU (20 mol%) for 12 h in the dark	n.d.	78
9	In the dark	n.d.	n.d.
10	Addition of TEMPO (2.0 equiv.)	n.d.	n.d.
11	Under air atmosphere	39	n.d.

a Reaction conditions: **1a** (0.075 mmol), **2a** (0.075 mmol), and **3a** (0.05 mmol) in DMSO (0.5 mL) at rt under irradiation using blue LEDs (440 nm, 10 W) for 3 h under N_2_.

b Yields were determined by ^1^H NMR spectroscopy.

c **1a** (0.05 mmol), **2a** (0.075 mmol) and **3a** (0.075 mmol) were used. Tol = *p*-tolyl. DMSO = dimethylsulfoxide. DMF = *N*,*N*-dimethylformamide. TEMPO = (2,2,6,6,-tetramethylpiperidin-1-yl)oxyl. n.d. = not detected.

After the successful development of sulfonative pyridylation reactions of alkenes, we set out to examine the scope of the current method by subjecting a wide range of substituted *N*-amidopyridinium salts, as shown in Table 2. First, unsubstituted and C2-phenyl derivatives readily reacted to afford the corresponding β-arylsulfonyl alkylpyridine products **4a** and **4b**. Substrates bearing *para*-methoxy and trifluoromethyl groups on the phenyl rings and dimethyl groups at the C2 position smoothly underwent the reaction to furnish the desired products (**4c–4e**). Notably, pyridinium salts containing a wide range of functional groups, such as methyl, phenyl, halide, and ester, at the C3 position, proved to be compatible with the process (**4f–4j**). Remarkably, excellent C4-selectivity was obtained in all cases (>20 : 1 r.r. if not denoted otherwise). Next, we explored the reaction with respect to sulfinates, and a number of aryl sulfinates with electronic perturbations on the aromatic ring were observed to be suitable sulfonyl sources (**4l–4o**). The structure of **4m** was elucidated based on X-ray crystallographic analysis.^23^ Sodium 2-naphthalenesulfinate also underwent the desired reaction smoothly, delivering **4p**. Likewise, a pyrimidyl sulfinate was effectively converted into the desired sulfone (**4q**). In addition, the method could be readily extended to alkyl sulfinate substrates (**4r**, **4s**, and **4t**), including cycloalkyl (**4u** and **4v**). Next, the generality with respect to the alkene component was subsequently investigated, and various electron-rich alkenes, such as vinyl ethers and enamides, and unactivated alkenes were subjected to the optimized reaction conditions. Aliphatic vinyl ethers bearing ethyl and cyclohexyl groups reacted readily, leading to the formation of **4w** and **4x**. Substrates containing polar functionalities, such as hydroxyl and chlorine groups, could be successfully engaged in this transformation to furnish the products (**4y** and **4z**). Phenyl vinyl ethers also served as an effective coupling partner (**4aa**), and employing substrates including *tert*-butyl at the *para*-position, benzodioxole, and coumarin functionalities was suitable under the reaction conditions to afford the desired products (**4ab**, **4ac**, and **4ad**). Notably, this method could be extended to the reactions of substrates bearing vinyl formamide and amide groups (**4ae–4ag**). Additionally, 2,3-dihydrofuran bearing internal olefins could be employed in this protocol to afford the desired product **4ah**. Remarkably, this strategy was successfully extended to aliphatic unactivated alkenes (**4ai–4ak**), which could not be achieved by typical strategies as reported before. Moreover, the generality of the mild reaction conditions was well demonstrated by the facile late-stage modification of a series of structurally diverse complex molecules. For example, this coupling could be extended to d-fructopyranose, β-citronellol, l-tyrosine, and estrone derivatives to afford desired products (**4al–4ao**) with C4 selectivity, highlighting the functional group compatibility. Finally, we examined the scope of dienes. The diallyldiphenylsilane was converted to the corresponding product *via* 6-*endo*-trig cyclization (**4ap**).^24^ Likewise, we could observe a transformation of 1,5-cyclooctadiene into a bicyclic product (**4aq**). In addition, a disubstituted tricyclic pyridine was produced in the reaction of 2,4-norbornadiene (**4ar**).^25^

**Table 2:** Substrate scope of three-component sulfonative pyridylation of alkenes^a^

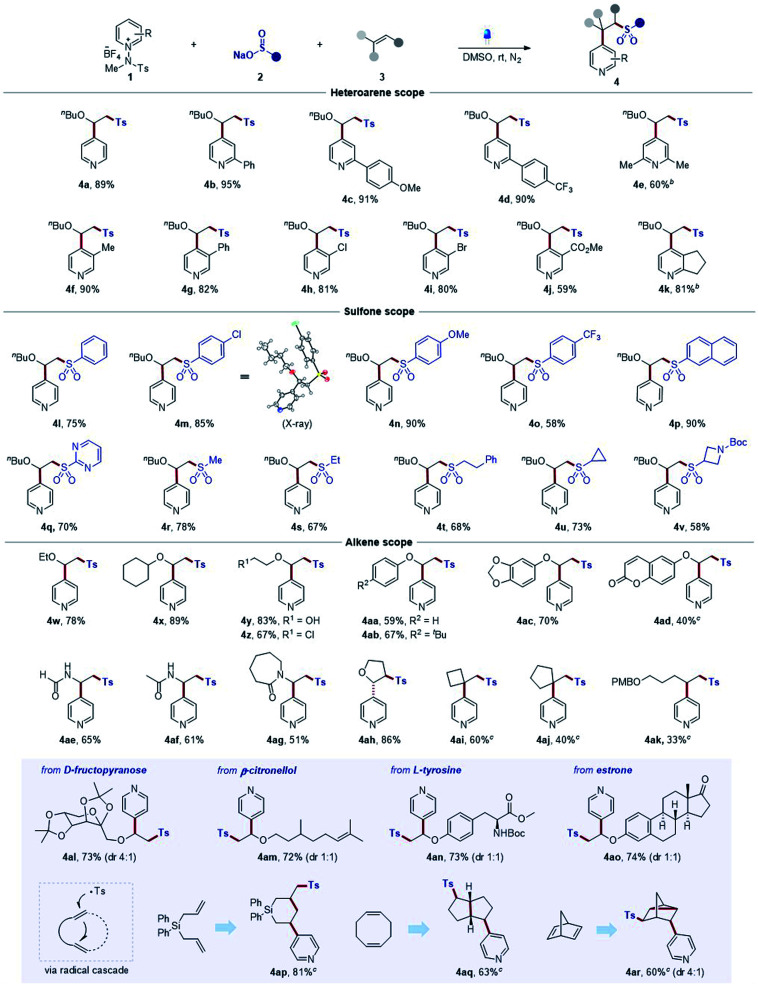

a Reaction conditions: **1** (0.15 mmol), **2** (0.15 mmol), and **3** (0.1 mmol) in DMSO (1.0 mL) at rt under irradiation using blue LEDs (440 nm, 10 W) for 3 h under N_2_. Isolated yield. Diastereomeric ratios were measured by ^1^H NMR spectroscopy. Unless indicated, the C4/C2 ratio >20 : 1.

b MeOH was used instead of DMSO for 12 h.

c 3.0 equiv. of sulfinate were used for 24 h. PMB = *p*-methoxybenzoyl.

**Scheme 2:**
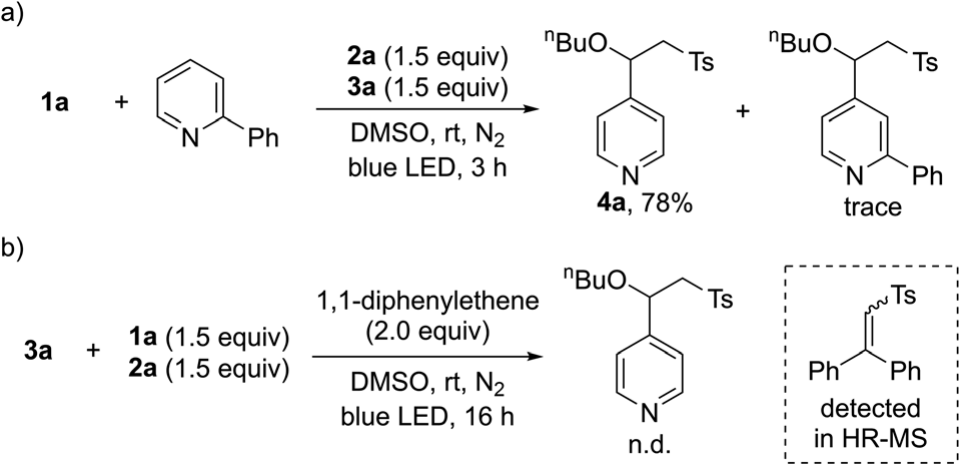
Control experiments for the three-component reactions. (a) Reaction with mixtures of **1a** and 2-phenyl-substituted pyridine. (b) Radical trapping experiments using 1,1-diphenylethene.

After demonstrating the general applicability of the broad substrate scope, we performed a series of control reactions to gain insight into the reaction mechanism. To determine whether the pyridines generated by intermolecular charge transfer could act as substrates, crossover experiments were carried out with a mixture of **1a** and 2-phenyl-substituted pyridines. As anticipated, the reaction occurred at the C4 position of **1a**, providing **4a** (Scheme 2a). Next, we conducted radical trapping experiments by adding a radical scavenger, 1,1-diphenylethene (Scheme 2b). The formation of the desired product was completely inhibited, suggesting that a radical pathway is involved in this transformation. In addition, a direct-trapping product of the sulfonyl radical was observed by HR-MS (see the ESI†).

**Fig. 2:**
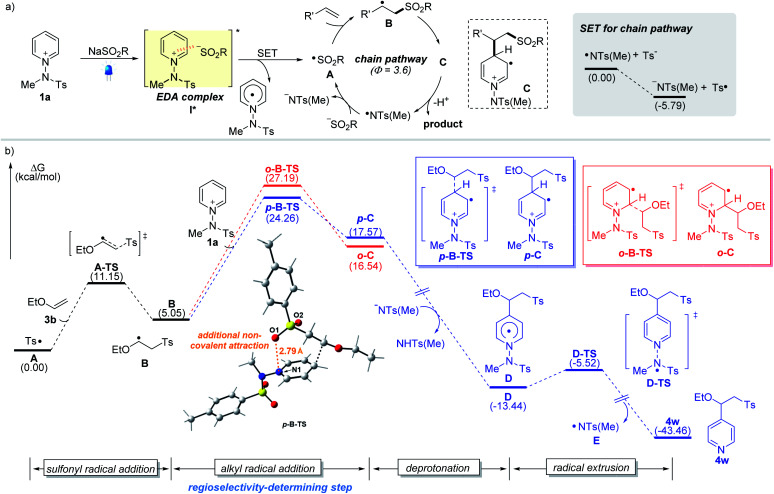
(a) Proposed mechanism for the sulfonative pyridylation of alkenes. (b) Free-energy profile for the site-selective sulfonative pyridylation of alkenes. The blue traces represent the C4-product formation pathway. The red traces represent the C2-product formation pathway.

To better understand the observed regioselectivity and mechanism, we investigated the reaction pathways using density functional theory (DFT) calculations. A plausible reaction mechanism involving the formation of photoactive EDA complexes and the computed reaction energy profiles are depicted (Fig. 2). Upon excitation by visible light, EDA complex I* undergoes an intermolecular single-electron transfer from the sulfinate to the salt. The resulting sulfonyl radical **A** adds to the double bond to yield the corresponding alkyl radical intermediate **B**, which adds to pyridinium salt **1a**. Our calculation suggests that the radical addition step between alkyl radical **B** and **1a** determines the regioselectivity (C4 *vs.* C2), resulting in two regioisomeric intermediates, **p-C** and **o-C**. The transition state **p-B-TS** leading to the C4-intermediate **p-C** was found to be favored by 2.9 kcal mol^−1^ over the other transition state **o-B-TS** (the red route), which is consistent with the observed C4-selectivity. An interaction energy difference is caused by the additional non-covalent attraction between the negatively charged sulfonyl oxygen and the nitrogen of the pyridinium substrate across a distance of 2.8 Å (see the ESI†).^16*a*^ Next, the reaction pathway proceeds through deprotonation of cationic radical **C** and homolytic N–N bond cleavage from **D***via* transition state **D-TS**, which delivers the desired product along with an amidyl radical **E** that makes possible a radical chain propagation reaction. The quantum yield (*Φ* = 3.6) measured under the standard reaction conditions suggests that the radical chain pathway is quite productive for the overall reaction. The generated amidyl radical appears to react with a sulfinate to furnish sulfonyl radical **A** and initiate the radical chain pathway. In addition, the SET between **E** and tosyl sulfinate is exergonic, making the production of the sulfonyl radical **A** 5.8 kcal mol^−1^ lower in Gibbs free energy than that for the amidyl radical **E**. Although it is reversible and possible to regenerate an amidyl radical, the sulfonyl radical will be predominantly populated considering the use of stoichiometric amounts of sulfinate and the thermodynamics for the generation of sulfonyl and amidyl radicals.

Given the pivotal importance of C4-sulfonylated pyridines and quinolines as key moieties in pharmaceuticals^26^ and synthetically useful components in organic chemistry,^27^ we next set out to expand the utility of our strategy by investigating the C4-sulfonylation of pyridinium salts. After a survey of the reaction parameters such as base, solvent, and concentration, the optimized reaction conditions were identified (see the ESI†), and the generality with respect to this protocol was explored, as illustrated in Table 3. Pleasingly, a wide range of pyridinium salts bearing ester, phenyl, bromide, chloride, and trifluoromethyl groups underwent the two-component reactions to provide the desired products (**5b–5g**) under the optimized conditions with excellent C4 regioselectivity. The structure of sulfone **5e** was confirmed by X-ray diffraction analysis.^28^ Expanding the scope from pyridine derivatives to other heterocycles, such as quinolines, was possible and desired products **5h**, **5i**, and **5j** wereyielded. Next, we evaluated cross-coupling with regard to the sulfinate coupling partner. Aryl sulfinates with electron-deficient and electron-rich functional groups were all tolerated (**5k–5m**). Similarly, 2-naphthyl and thiophenyl sulfinates were also suitable substrates, leading to products **5n** and **5o**. Moreover, this method worked well with a series of alkyl sulfinates to give the desired products (**5p–5r**). In addition, cyclic sulfinates readily participated in this transformation to afford **5s** and **5t**.

**Table 3:** Substrate scope of C4-sulfonylation^a^

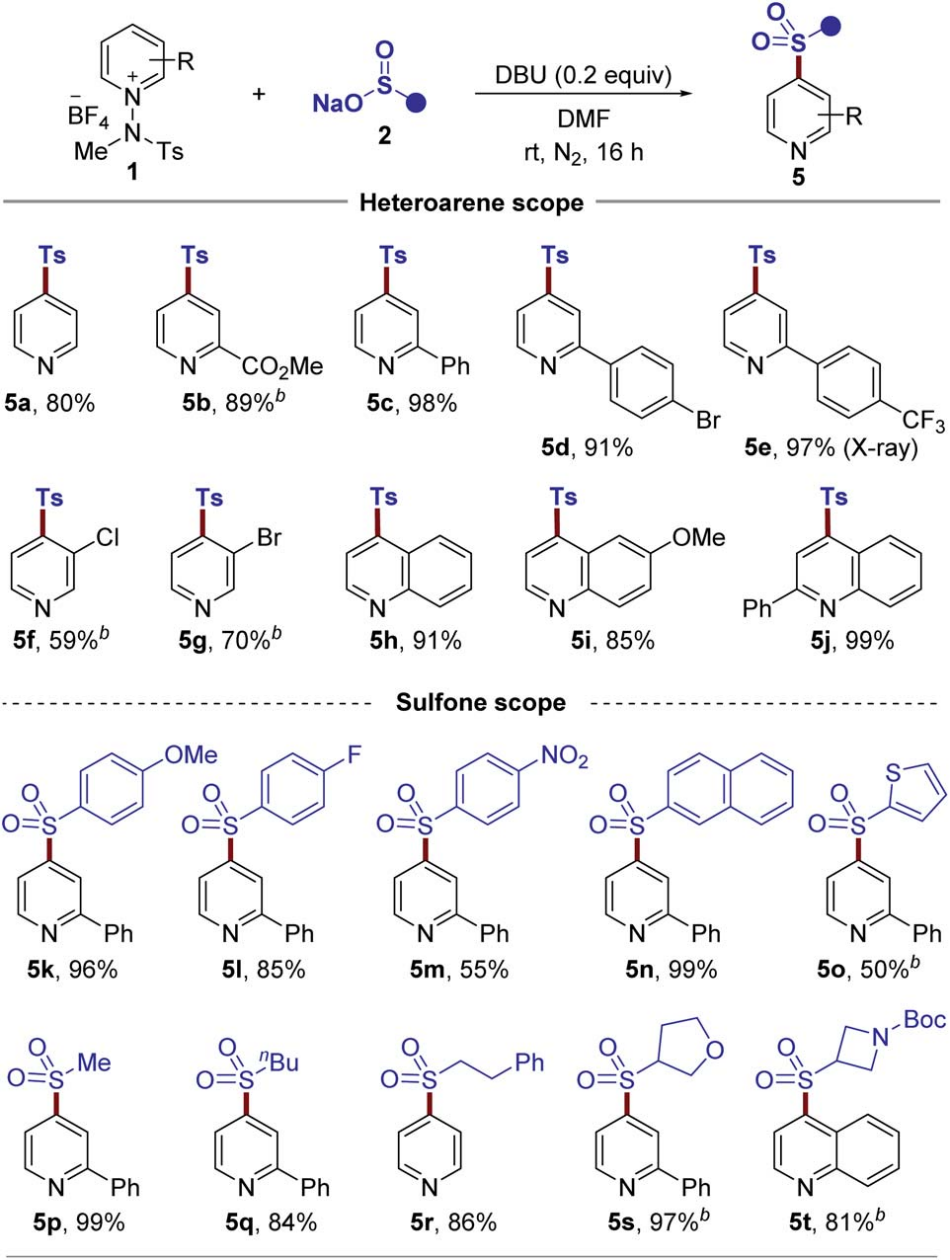

a Reaction conditions: **1** (0.1 mmol), **2** (0.11 mmol) and DBU (0.02 mmol) in DMF (1.0 mL) at rt for 16 h under N_2_. Isolated yield.

b 0.5 equiv. of DBU were used.

**Fig. 3:**
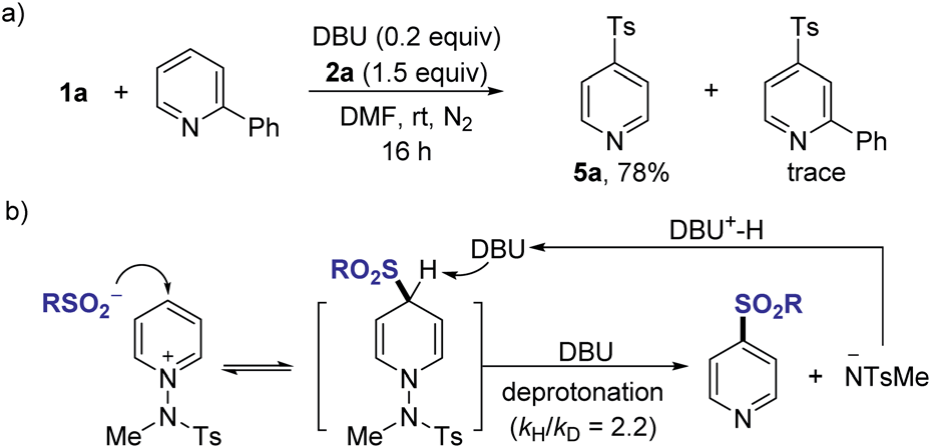
Control experiments and the proposed mechanism for the two-component reactions. (a) Reaction with mixtures of **1a** and 2-phenyl-substituted pyridine. (b) Proposed mechanism.

Further, experiments aimed at elucidating the reaction mechanism were performed. No reactivity was observed when an external base was not used (see the ESI†). When using TEMPO as an additive, the reactivity was not significantly suppressed, and no TEMPO-sulfonyl radical adduct was observed in the NMR or LC-MS of the crude reaction mixture, suggesting that a radical intermediate pathway is not predominant in this system (see the ESI†). A competitive experiment using a mixture of **1a** and 2-phenylpyridine showed that the reaction took place only with **1a**, while 2-phenylpyridine was not involved in the nucleophilic addition (Fig. 3a). Based on the several results, we could propose a mechanism for DBU-mediated sulfonylation of pyridinium salts as described (Fig. 3b). Parallel competition reactions between **1a** and its deuterated analog **1a–d5** were performed, in which the *k*_H_/*k*_D_ was found to be 2.2 (see the ESI†), indicating the reversible process of nucleophilic addition of sulfinates.^29^

**Scheme 3:**
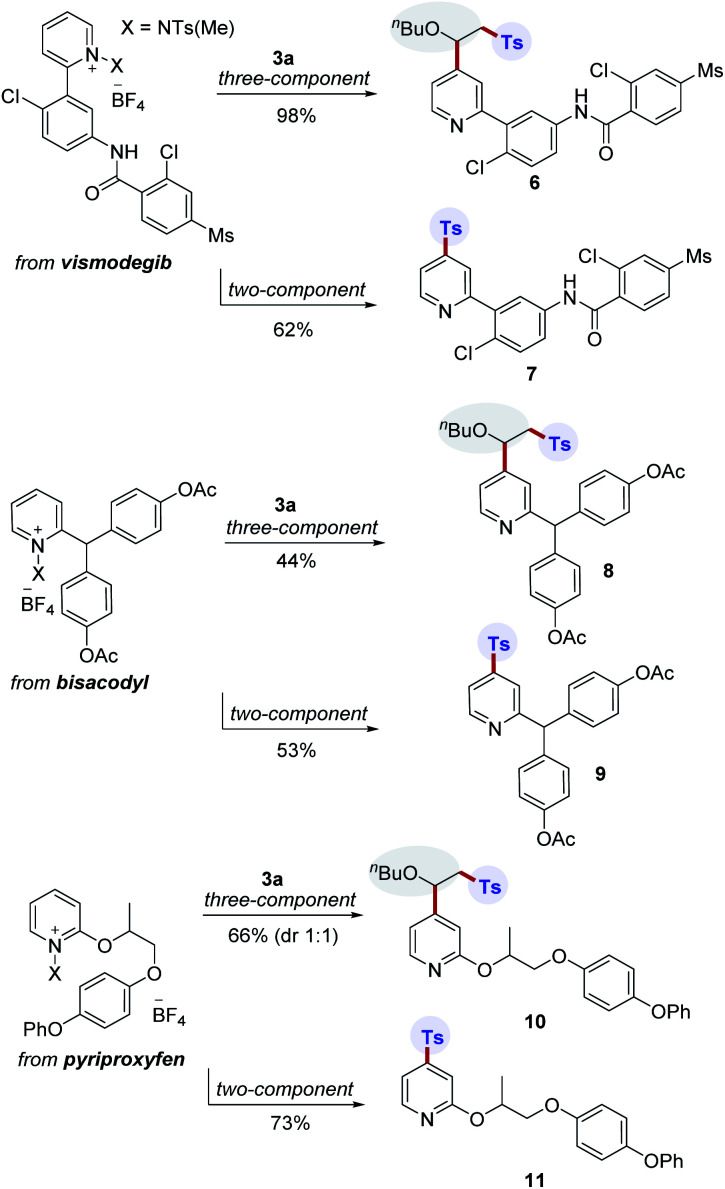
Late-stage modifications of pyridine-based drug derivatives.

The incorporation of sulfonyl groups onto pharmaceutically relevant building blocks is important for modulating bioactivity and properties for the construction of diverse compound libraries. To further highlight the broad utility of this transformation, divergent late-stage modifications of pharmaceuticals were next investigated (Scheme 3). Pleasingly, we were able to conduct site-selective functionalization of complex pyridine-based drug molecules such as vismodegib, bisacodyl, and pyriproxyfen using two- and three-component protocols. In this manner, the corresponding products (**6–11**) were successfully generated.

**Scheme 4:**
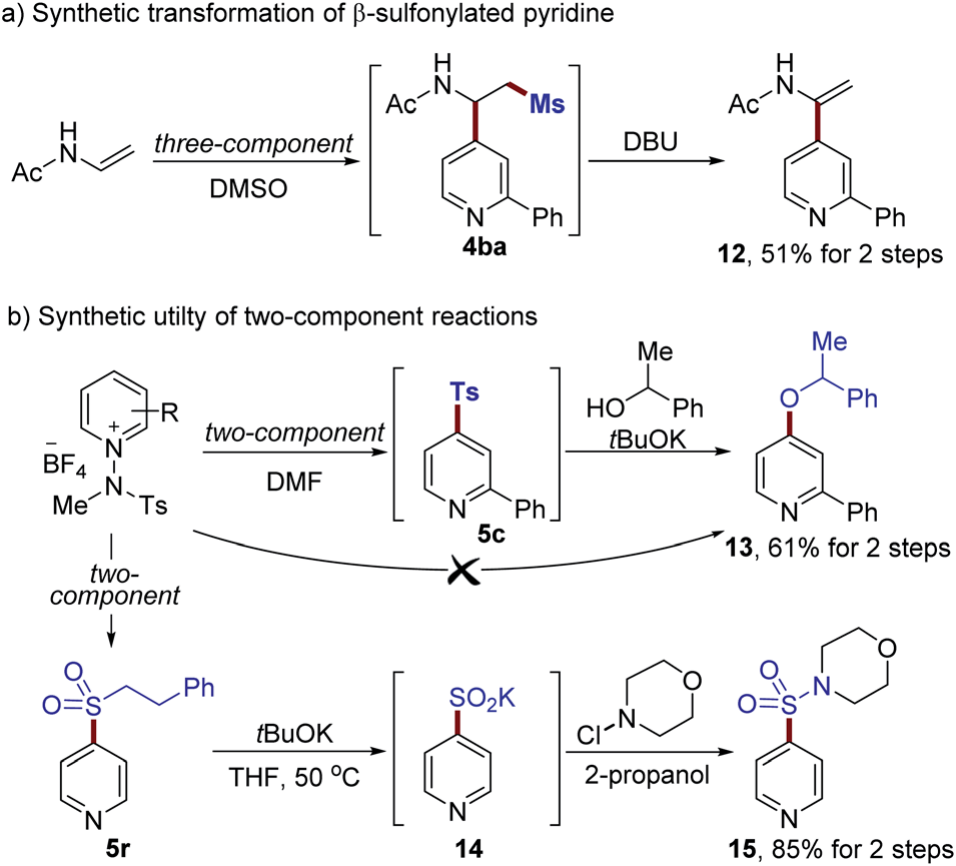
Synthetic utilities of sulfones **4ba**, **5c** and **5r** (see the ESI† for details).

Finally, we set out to explore the utility of the current methods for further diversification, as demonstrated in Scheme 4 (see the ESI†). A β-methylsulfonyl pyridine derivative **4ba** was readily transformed into an enamide **12** (51% for 2-steps, Scheme 4a). In addition, the displacement of a sulfonyl group with alcohol *via* S_N_Ar could be achieved using alkoxides to afford **13** (61% yield for 2-steps, Scheme 4b). Phenyl ethyl sulfone **5r** is a versatile moiety that can easily be converted to the corresponding sulfinate. For example, **5r** could be modified by using ^*t*^BuOK, enabling the formation of a sulfinate **14** in excellent yield.^30^ Further transformations using *N*-chloromorpholine as an electrophile provided sulfonamide **15** in 85% yield.

## Conclusions

In summary, we have developed a divergent system to selectively enable the sulfonative pyridylation of alkenes and C4-sulfonylation of pyridines through two distinct pathways. Harnessing the photochemical activity of EDA complexes from sulfinates and *N*-amidopyridinium salts, a three-component reaction with alkenes was orthogonally achieved without using external photocatalysts. This radical pathway allows for a rapid assembly of sulfonyl and pyridyl moieties at the ethylene linker with high functional group tolerance. DBU is found to be capable of promoting coupling between the identical reactants, thus enabling the selective insertion of a sulfonyl group at the C4 position of pyridines. Furthermore, these operationally simple protocols can be readily applied to the late-stage modification of complex molecules to provide new retrosynthetic disconnections that would otherwise be difficult to access. This study highlights the synthetic potential of divergent transformations by leveraging the intrinsic nature of sulfinates and *N*-amidopyridinium salts.

## Author contributions

M. K., E. Y. and S. P. performed the experiments. M. K. conducted computational studies. S. H. directed the project. All authors contributed to the preparation of the manuscript.

## Conflicts of interest

There are no conflicts to declare.

## Supplementary Material

SC-012-D1SC00776A-s001

SC-012-D1SC00776A-s002
