# Supplementary material for: Divergent reactivity of sulfinates with pyridinium salts based on one- versus two-electron pathways
Source: Chem Sci. 2021 Mar 31;12(19):6629–37. doi: 10.1039/d1sc00776a (PMC8132931; doi:10.1039/d1sc00776a)
Supplement: SC-012-D1SC00776A-s001 [file SC-012-D1SC00776A-s001.pdf]

Electronic Supplementary Material (ESI) for Chemical Science.
This journal is © The Royal Society of Chemistry 2021

Supplementary Information

Divergent Reactivity of Sulfinates with Pyridinium Salts Based on One- versus Two-Electron Pathways

Myojeong Kim, Euna You, Seongjin Park, and Sungwoo Hong*

Department of Chemistry, Korea Advanced Institute of Science & Technology (KAIST), Daejeon 34341, Korea

Center for Catalytic Hydrocarbon Functionalizations, Institute for Basic Science (IBS), Daejeon 34141, Korea

Ⅰ. General Methods and Materials **S2**

Ⅱ. Experimental Procedure **S3**

III. Absorption Spectra **S8**

IV. Cyclic Voltammetry Measurement **S9**

V. Control Experiments **S15**

VI. Synthetic Utility **S28**

VII. Computational Study **S31**

VIII. Compound Characterizations **S41**

Appendix I
Spectral Copies of ^{1}H-, ^{13}C- and ^{19}F-NMR Data Obtained in this Study **S72**

Appendix II
DFT Calculation Data **S152**

Appendix III
Crystallographic Data Obtained in this Study **S162**

I. General Methods and Materials

Commercial grade reagents and solvents were used without further purification except as indicated below. Unless stated otherwise, reactions were performed in flame-dried glassware. Analytical thin layer chromatography (TLC) was performed on precoated silica gel 60 F^{254} plates and silica gel 60 RP-18 F^{254}s, and visualization on TLC was achieved by UV light (254 and 365 nm). Flash column chromatography was performed on silica gel (400-630 mesh) or a Combi*Flash*® R_f^+ system with Redi*Sep*® R_f silica columns (230-400 mesh) using a proper eluent. ^{1}H NMR was recorded on Brucker Avance 400 MHz, 500 MHz or Agilent Technologies DD2 600 MHz. Chemical shifts were quoted in parts per million (ppm) referenced to the appropriate solvent peak or 0.0 ppm for tetramethylsilane. The following abbreviations were used to describe peak splitting patterns when appropriate: br = broad, s = singlet, d = doublet, t = triplet, q = quartet, m = multiplet, dd = doublet of doublet, td = triplet of doublet, ddd = doublet of doublet of doublet. Coupling constants, *J*, were reported in hertz unit (Hz). ^{13}C NMR was recorded on Brucker Avance 100 MHz or Agilent Technologies DD2 150 MHz and was fully decoupled by broad band proton decoupling. Chemical shifts were reported in ppm referenced to the centerline of a triplet at 77.0 ppm of $CDCl_3$. ^{19}F NMR was recorded on Brucker Advance 376 MHz. High resolution mass spectroscopy was conducted on a Bruker Daltonik micrOTOF-QII and obtained by using EI or FAB from Korea Basic Science Institute (Daegu) or ESI from KBSI (Ochang). Infrared (IR) spectra were recorded on Bruker Alpha FT-IR Spectrometer. The Absorption spectra were measured by a spectrophotometer (V-530 UV/Vis Spectrophotometer, Jasco, Inc.). The emission spectra were measured by a fluorimeter (RF-5301PC Spectro-fluorophotometer, SHIMADZU Corp.).

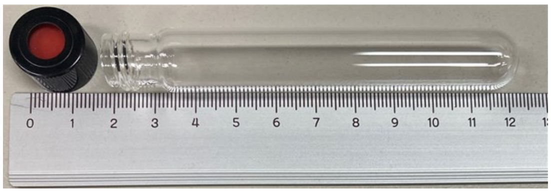

Fig. S1. Test tube (12 mL, 15 mm x 100 mm) used in this study (Samwoo Kurex, Borosilicate, Type 1 glass).

II. Experimental Procedure

General procedure for visible-light-induced sulfonative pyridylation of alkenes (GP1)

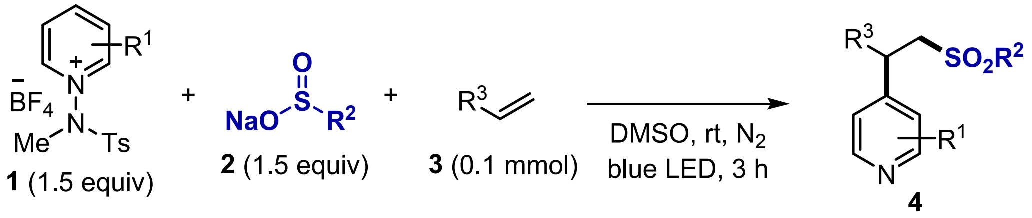

To a 12 mL test tube equipped with a magnetic bar were added *N*-aminopyridinium salt **1** (1.5 equiv, 0.15 mmol) and sodium sulfinate (**2**) (1.5 equiv, 0.15 mmol), and the tube sealed with a PTFE septum. After evacuating the tube and back-filling with nitrogen, olefin (**3**) (1.0 equiv, 0.10 mmol) and dimethylsulfoxide (DMSO, 1.0 mL) were added via syringe. The sealed test tube was placed at the reaction bath equipped with Kessill PR1160-440 nm blue LEDs (25% intensity). The reaction mixture was stirred at room temperature for 3 h. After reaction completion, the reaction mixture was diluted with ethyl acetate (EtOAc, 30 mL), and then washed with water (20 mL x 2) and brine (20 mL). The organic layer was dried over anhydrous sodium sulfate, filtered, and concentrated under reduced pressure. The crude mixture was purified by flash column chromatography on silica gel (eluent: acetone/*n*-hexane = 1:4~1:2) to give the desired product **4**.

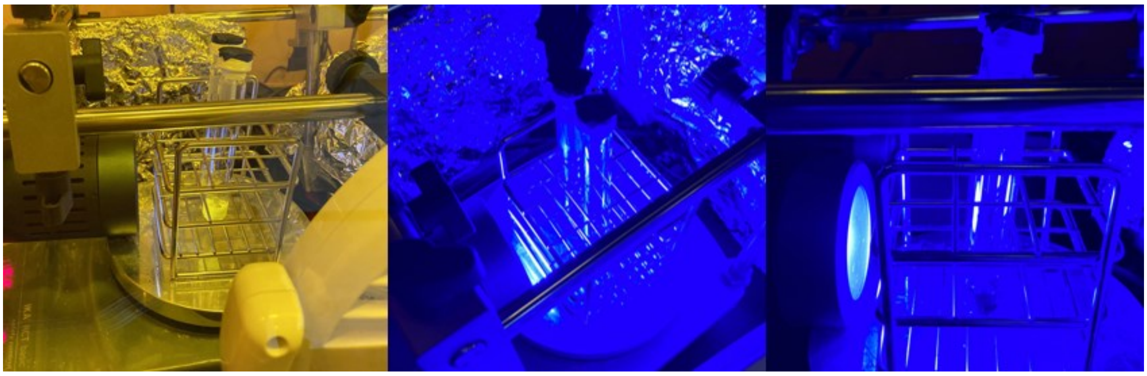

Fig. S2. Reaction set-up for **GP1** (photo provided by the authors).

Table S1. Optimization of reaction conditions for β-sulfonylated pyridine (4a)[a]

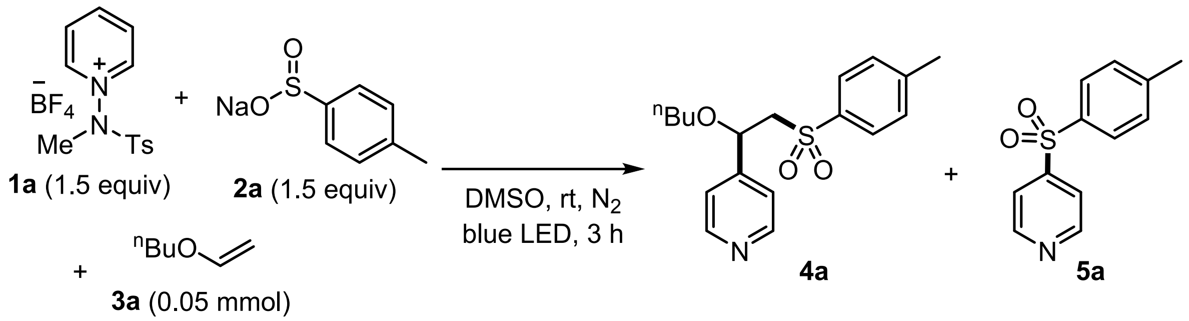

entry	variations from standard conditions	yield of 4a (%)[b]	yield of 5a (%)[b]
1	none	90	n.d.
2	DMF instead of DMSO	77	n.d.
3	green LED (525 nm) instead of blue LED	31	n.d.
4	green LED (525 nm) instead of blue LED for 12 h	90	n.d.
5	using $[Ir(dFCF_3ppy)_2(bpy)]PF_6$ (5.0 mol%)	65	n.d.
6	using Eosin Y (0.5 mol%)	67	n.d.
7	using Eosin Y (2.0 mol%)	67	n.d.
8	using Eosin Y (0.5 mol%), green LED (525 nm)	66	n.d.
9	using Eosin Y (2.0 mol%), green LED (525 nm)	63	n.d.
10	0.05 M instead of 0.1 M	79	n.d.
12	0.2 M instead of 0.1 M	89	n.d.
13	for 12 h	90	<5
14	at 50 °C	68	<5
15	using NaOAc (1.2 equiv) for 12 h	n.d.	6
16	using NaOAc (1.2 equiv) for 12 h in the dark	n.d.	20
17[c]	using DBU (20 mol%) for 12 h	<5	61
18[c]	using DBU (20 mol%) for 12 h in the dark	n.d.	78
19d]	in the dark	n.d.	n.d.

[a] Reaction conditions: Pyridinium salt **1a** (0.075 mmol), **2a** (0.075 mmol) and **3a** (0.05 mmol) in DMSO (0.5 mL) upon irradiation using blue LEDs (440 nm, 10 W) at rt for 3 h under N_2. [b] Yields were measured by ^1H NMR spectroscopy. [c] **1a** (0.05 mmol), **2a** (0.075 mmol) and **3a** (0.075 mmol) were used. [d] >90% of alkene reactants was remained. TEMPO = (2,2,6,6-tetramethylpiperidin-1-yl)oxyl.

Table S2. Screening of solvents[a]

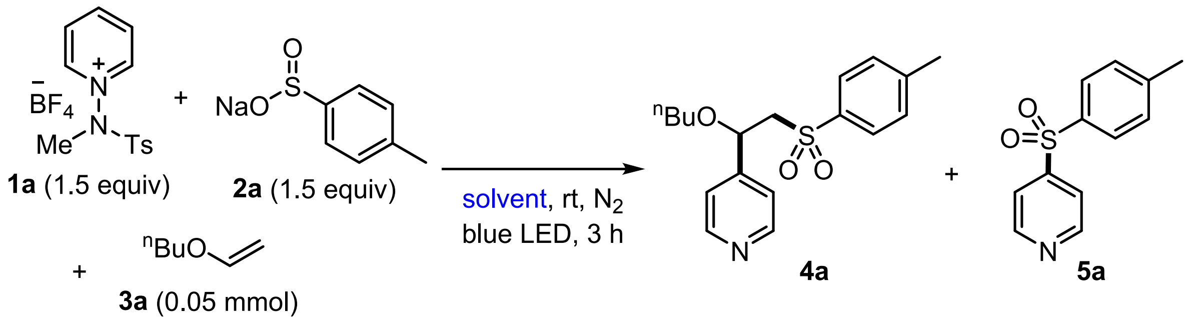

entry	solvent	yield of 4a (%)[b]	yield of 5a (%)[b]
1	DMSO	90	n.d.
2	DMF	77	n.d.
3	DMA	75	n.d.
4	methanol	27	n.d.
5	1,2-dichloroethane	<5	n.d.
6	acetonitrile	20	n.d.

[a] Reaction conditions: **1a** (0.075 mmol), **2a** (0.075 mmol) and **3a** (0.05 mmol) in the indicated solvent (0.5 mL) upon irradiation using blue LEDs (440 nm, 10 W) at rt for 12 h under N_2. [b] Yields were measured by ^1H NMR spectroscopy.

General procedure for DBU-mediated sulfonylation (GP2)

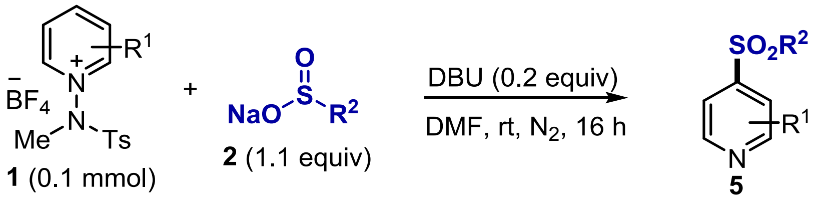

To a 12 mL test tube equipped with a magnetic bar were added **1** (1.0 equiv, 0.1 mmol) and sulfinate **2** (1.1 equiv, 0.11 mmol), and the tube was sealed with a PTFE septum. After evacuating the tube and back-filling with nitrogen, dimethylformamide (DMF, 1 mL) and 1,8-diazabicyclo(5,4,o)undec-7-ene (DBU, 0.2 equiv, 0.02 mmol) were added via syringe at 0 °C. The resulting mixture was stirred at room temperature for 16 h. After reaction completion, the reaction mixture was diluted with EtOAc (30 mL), and then washed with water (20 mL x 2) and brine (20 mL). The organic layer was dried over anhydrous sodium sulfate, filtered, and concentrated under reduced pressure. The crude mixture was purified by flash column chromatography on silica gel (EtOAc/*n*-hexane = 1:4~1:2) to give the desired product **5**.

Table S3. Optimization of reaction conditions for synthesis of sulfonylated pyridine (5a)[a]

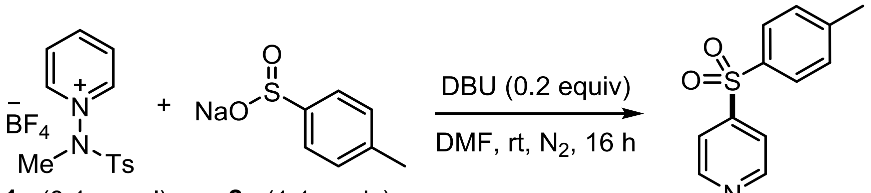

1a (0.1 mmol) **2a** (1.1 equiv) **5a**

entry	variations from standard conditions	yield of 5a (%)[b]
1	none	80
2	DMSO instead of DMF	78
3	THF instead of DMF	24
4	DBU (0.1 equiv)	24
5	DBU (0.5 equiv)	80
6	using NaOAc (0.5 equiv) instead of DBU	15
7	using TBAOAc (0.5 equiv) instead of DBU	14
8	using triethylamine (0.5 equiv) instead of DBU	10
9	using quinuclidine (0.5 equiv) instead of DBU	38
10	0.05 M instead of 0.1 M	77
11	0.2 M instead of 0.1 M	46

[a] Reaction conditions: **1a** (0.1 mmol) and **2a** (0.11 mmol) in DMF (1.0 mL) at 0 °C to rt for 16 h under N_2. [b] Yields were measured by ^{1}H NMR spectroscopy.

Table S4. Screening for visible-light-induced sulfonylation[a]

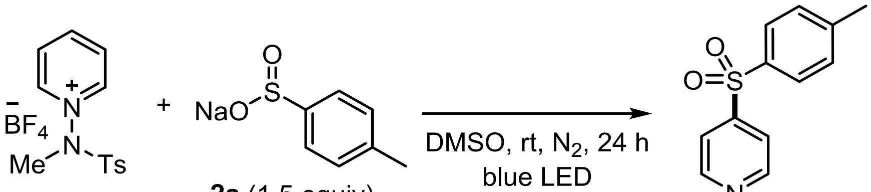

1a (0.05 mmol) **2a** (1.5 equiv) **5a**

entry	variations from standard conditions	yield of 5a (%)[b]
1	none	7
2	DMF instead of DMSO	<5
3	acetonitrile instead of DMSO	n.d.
4	for 3 h	n.d.
5	for 48 h	7
6	under violet LED (390 nm, 10 W)	<5
7	under blue LED (440 nm, 40 W)	7

8	using **2a** (4.0 equiv)	27

[a] Reaction conditions: **1a** (0.05 mmol) and **2a** (0.075 mmol) in DMSO (0.5 mL) upon irradiation using the indicated light sources at rt for 12 h under N_2. [b] Yields were measured by ^1H NMR spectroscopy.

Preparation of *N*-tosyl 1-aminopyridinium salts

All *N*-aminopyridinium salts employed in this study were prepared according to the previously reported procedures.[1]

Preparation of alkenes

Vinyl ethers and amides were purchased from commercial source or synthesized according to the previously reported vinylation methods.[2]

2.0 mmol scale procedure for visible-light induced sulfonative pyridylation of alkenes

Scheme S1

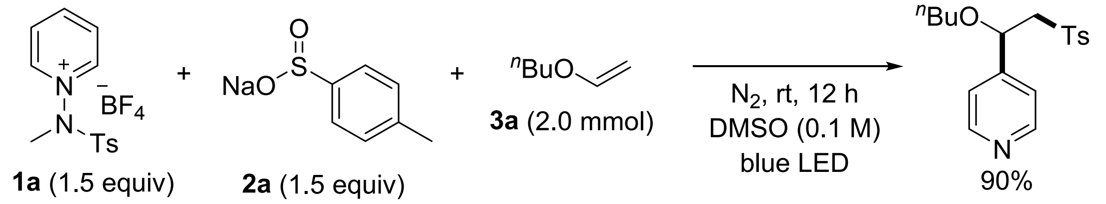

Reactions were conducted in Schlenk tube (50 mL) with rubber septa. *N*-aminopyridinium tetrafluoroborate (**1a**) (3.0 mmol) and sulfinate **2a** (3.0 mmol) were combined in DMSO (20 mL) under N_2 atmosphere. To the reaction mixture was added *n*-butylvinylether (**3a**) (2.0 mmol) via syringe. The sealed test tube was sonicated for 10 seconds, and immediately placed in the irradiation apparatus equipped with Kessill PR1160-440 nm blue LEDs (100% intensity x 2). The reaction mixture was stirred at room temperature for 12 h. After reaction completion, the reaction mixture was diluted with EA (120 mL), and washed with water (100 mL x 2) and brine (100 mL). The organic layer was dried over anhydrous sodium sulfate, filtered, and concentrated under reduced pressure. The crude mixture was purified by flash chromatography on silica gel (eluent: acetone/*n*-hexane = 1:4) to give the desired product compound **4a** (90%, 600.0 mg) as a white solid.

2.0 mmol scale procedure for C4-sulfonylation

Scheme S2

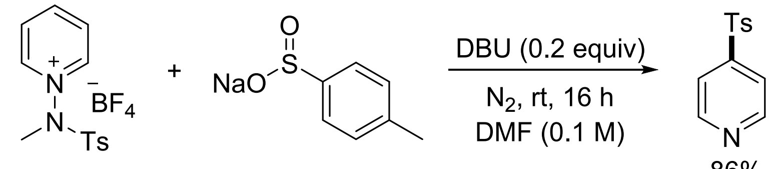

1a (2.0 mmol) **2a** (1.1 equiv)

Reactions were conducted in Schlenk tube (50 mL) with rubber septa. **1a** (2.0 mmol) and **2a** (2.2 mmol) were combined in DMF (20 mL) under N_2 atmosphere. To the reaction mixture was added DBU (0.4 mmol) via syringe at 0 °C. The resulting mixture was stirred at room temperature for 16 h. After reaction completion, the reaction mixture was diluted with EtOAc (120 mL), and then washed with water (100 mL x 2) and brine (100 mL). The organic layer was dried over anhydrous sodium sulfate, filtered, and concentrated under reduced pressure. The crude mixture was purified by flash column chromatography on silica gel (EtOAc/*n*-hexane = 1:4~1:2) to give the desired product **5a** (86%, 401.3 mg) as a white solid.

III. Absorption Spectra

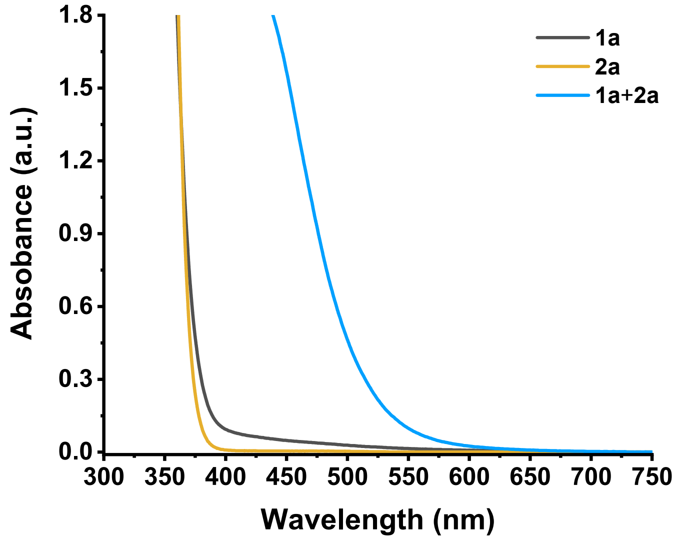

Fig. S3. Absorption spectra for **1a** (0.15 M in DMSO) with sodium *p*-tolylsulfinate **2a** (0.15 M in DMSO)

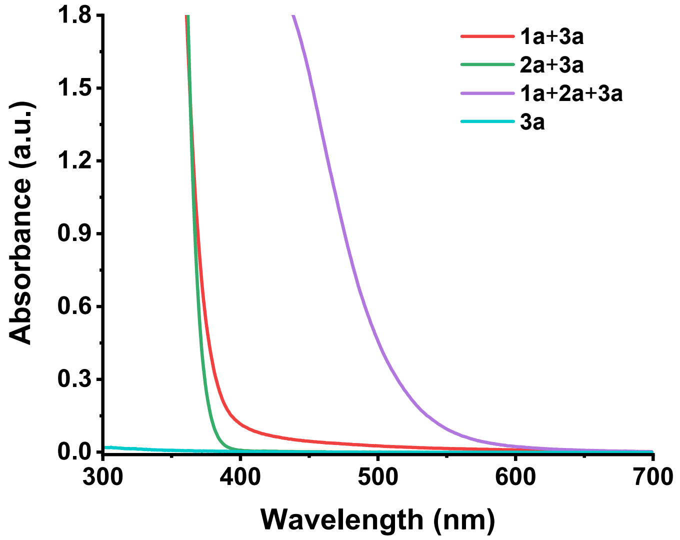

Fig. S4. Absorption spectra for **1a** (0.15 M in DMSO) and **2a** (0.15 M in DMSO) with **3a** (0.1 M in DMSO)

IV. Cyclic Voltammetry Measurement

Cyclic voltammetry was measured by a potentiostat (CH instrument, 600E) with conventional three electrode system (Reference electrode: Ag/Ag+, working electrode: Glassy carbon, counter electrode : Pt wire, Supporting electrolyte: 0.1 M NBu_4PF_6 in CH_3CN) at 100 mV/sec of scan rate. The potentials are given relative to the Fc/Fc^+ redox couple with ferrocene as internal standard.[3] The measurements were carried out as follows: 0.1 M solution of NBu_4PF_6 in acetonitrile was added to the measuring cell and the solution was degassed by argon purge for 3 min. After recording the baseline the electroactive compound was added (0.01 M) and the solution was again degassed a stream of argon for 3 min. The cyclic voltammogram was recorded with one to three scans. Afterwards ferrocene (2.20 mg) was added to the solution and the final measurement was performed with three scans.

Table S5. Reduction potentials of **1**.[a]

entry	compound		E° (red) vs. SCE [V]
1	**1a**	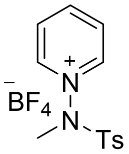	-0.82
2	**1b**	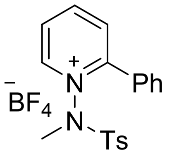	-0.75
3	**1c**	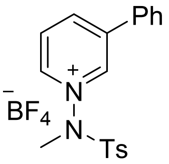	-0.78
4	**1d**	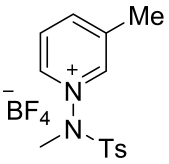	-0.87
5	**1e**	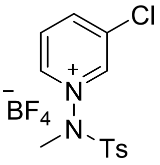	-0.62
6	**1f**	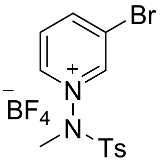	-0.67

7	**1g**	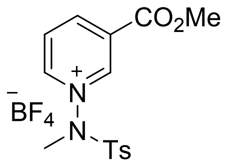	-0.65

[a]Determined with cyclic voltammetry in CH_3CN under argon and ferrocene as internal standard.

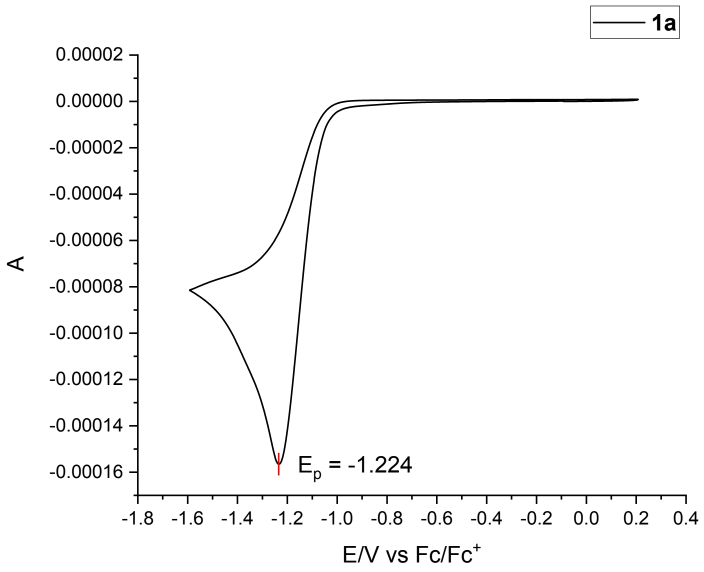

Fig. S5. CV of **1a** (10 mM in CH_3CN)

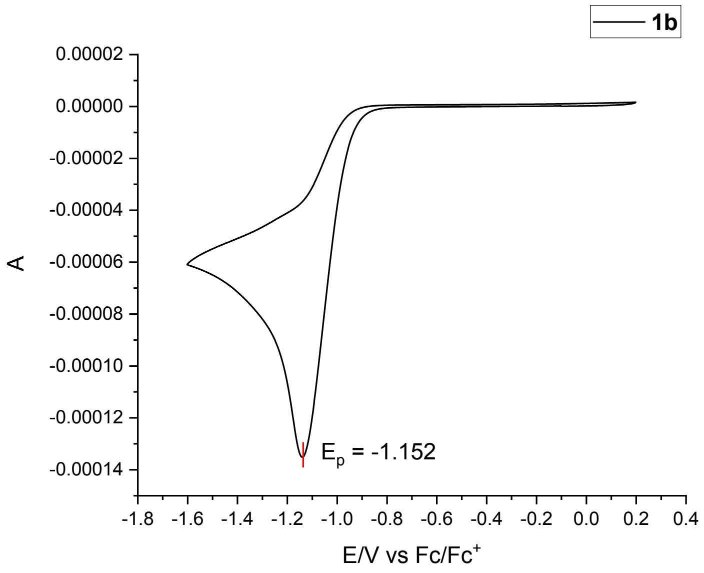

Fig. S6. CV of **1b** (10 mM in CH_3CN)

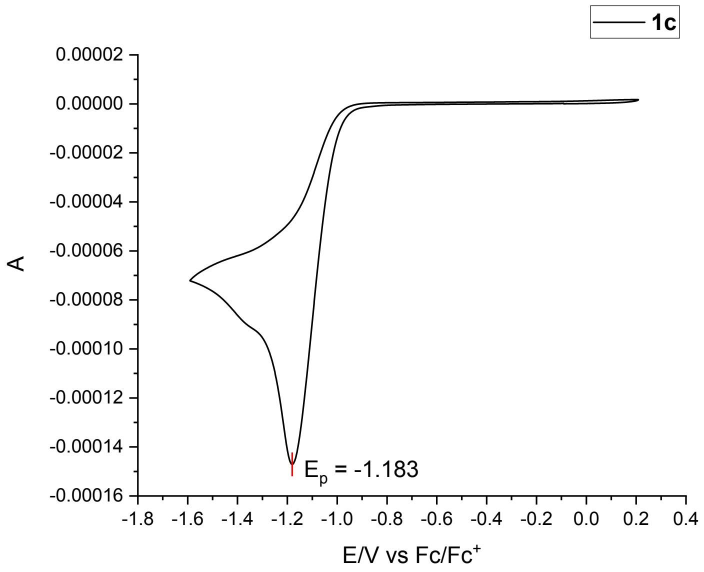

Fig. S7. CV of **1c** (10 mM in CH_3CN)

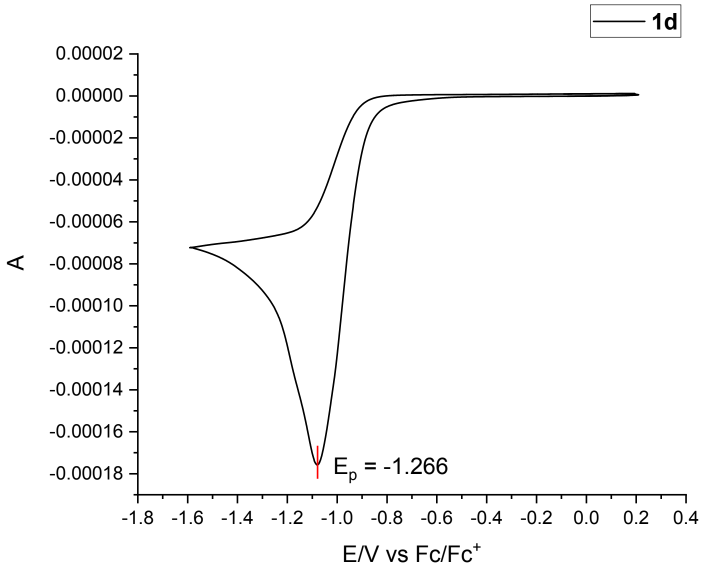

Fig. S8. CV of **1d** (10 mM in CH_3CN)

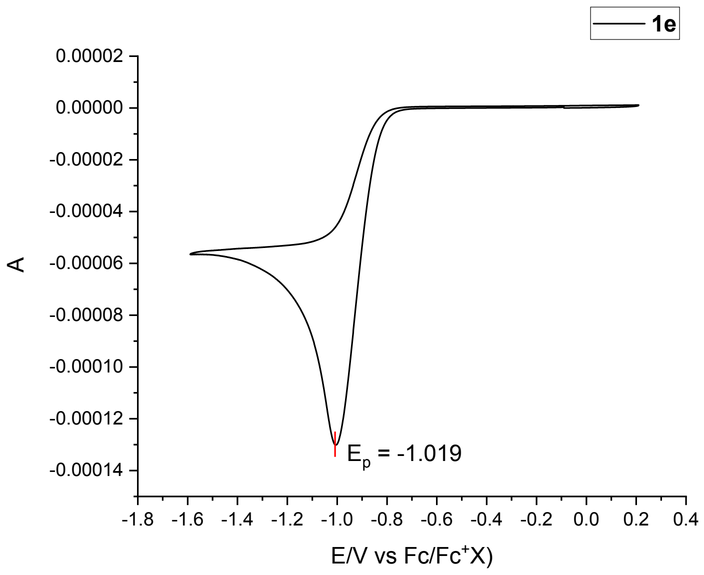

Fig. S9. CV of **1e** (10 mM in CH_3CN)

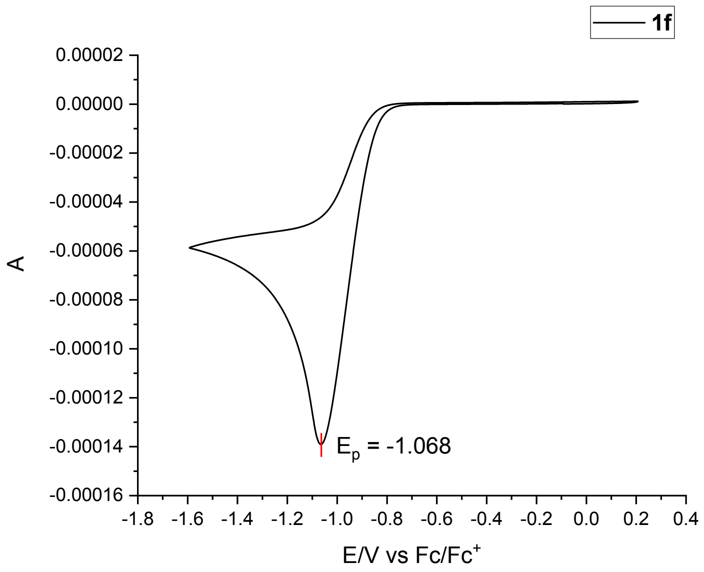

Fig. S10. CV of **1f** (10 mM in CH_3CN)

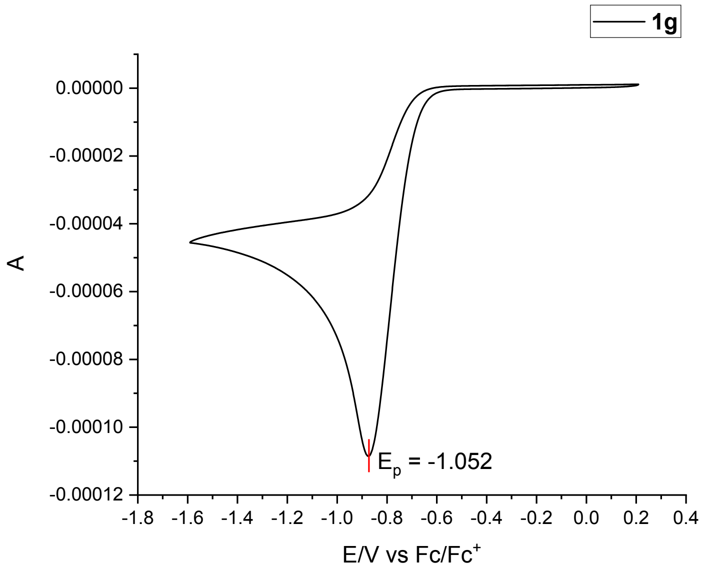

Fig. S11. CV of **1g** (10 mM in CH_3CN)

V. Control Experiments

(a) Control experiments for three-component reactions

Competition experiments

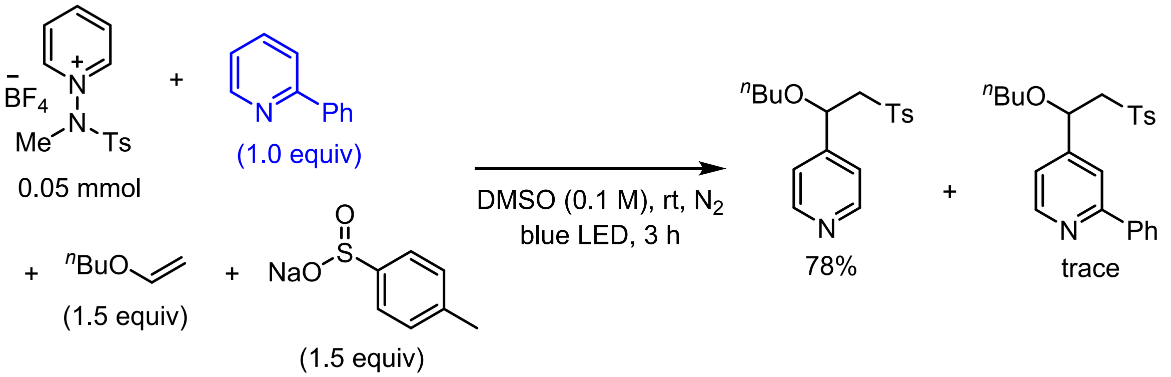

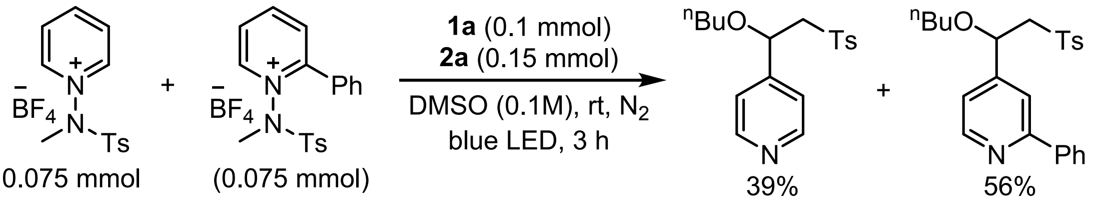

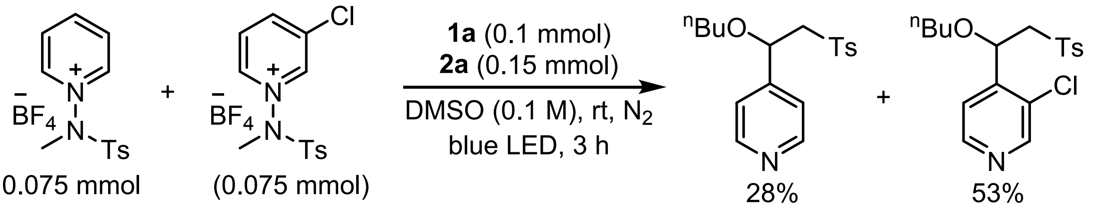

Under Air

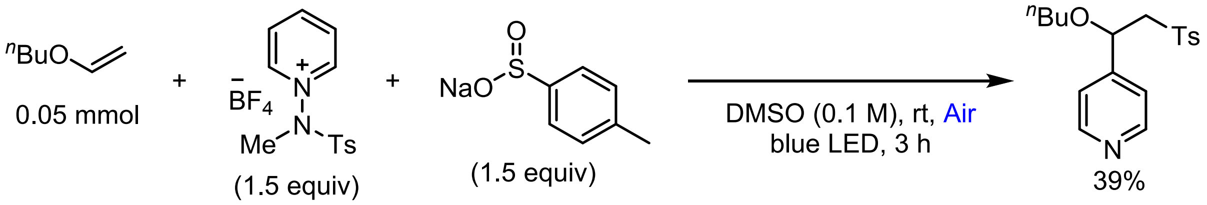

Under O_2

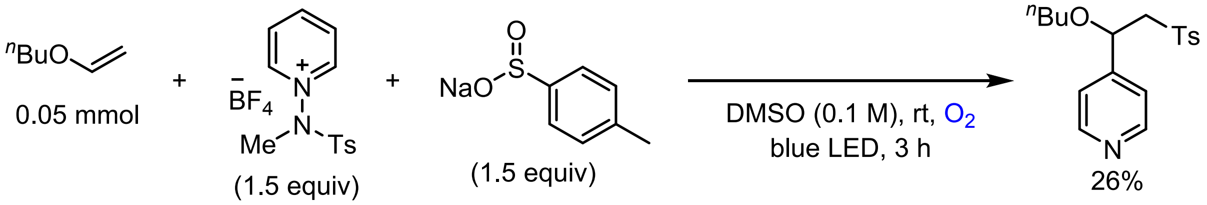

With water

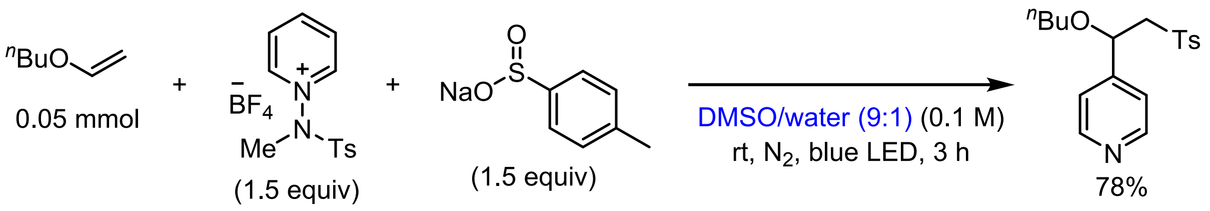

With TEMPO

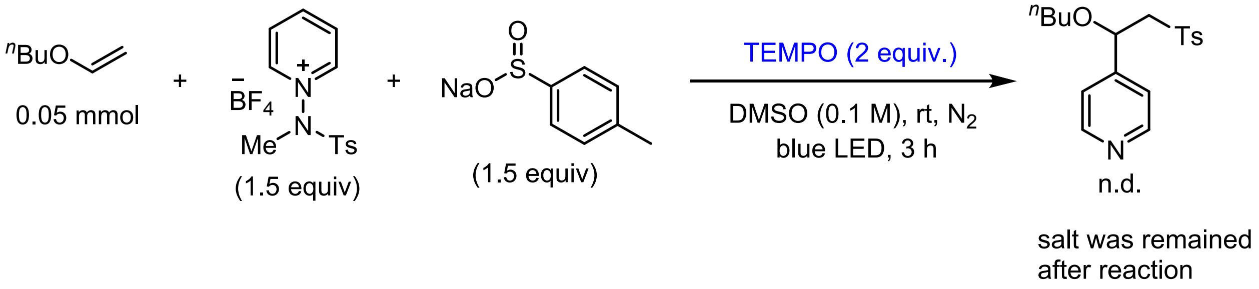

With C4-blocked pyridinium salts

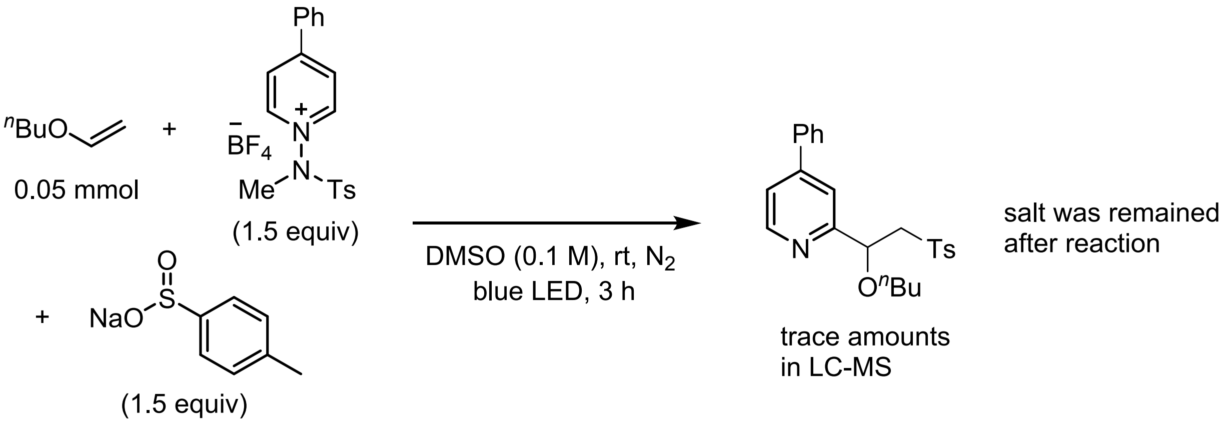

Radical trapping experiments using 1,1-diphenylethene

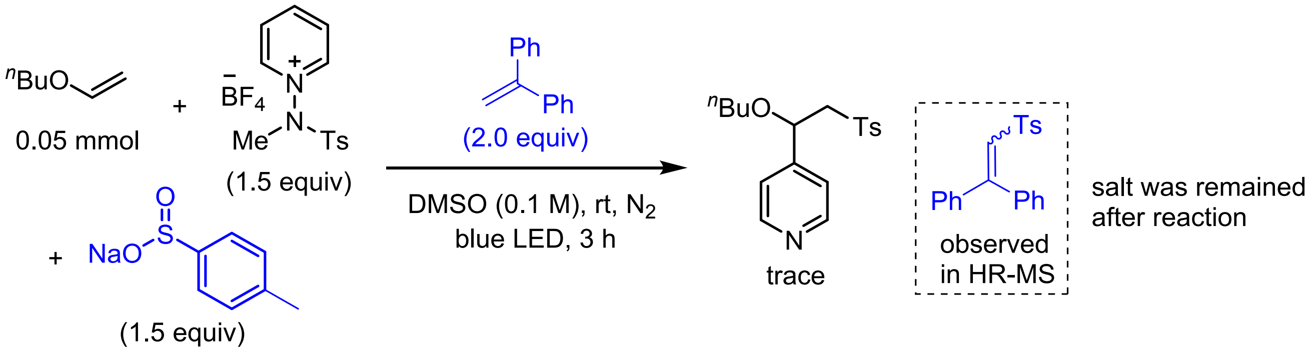

Phenylsulfonyl-diphenylethene adduct was obtained by radical trapping experiment.

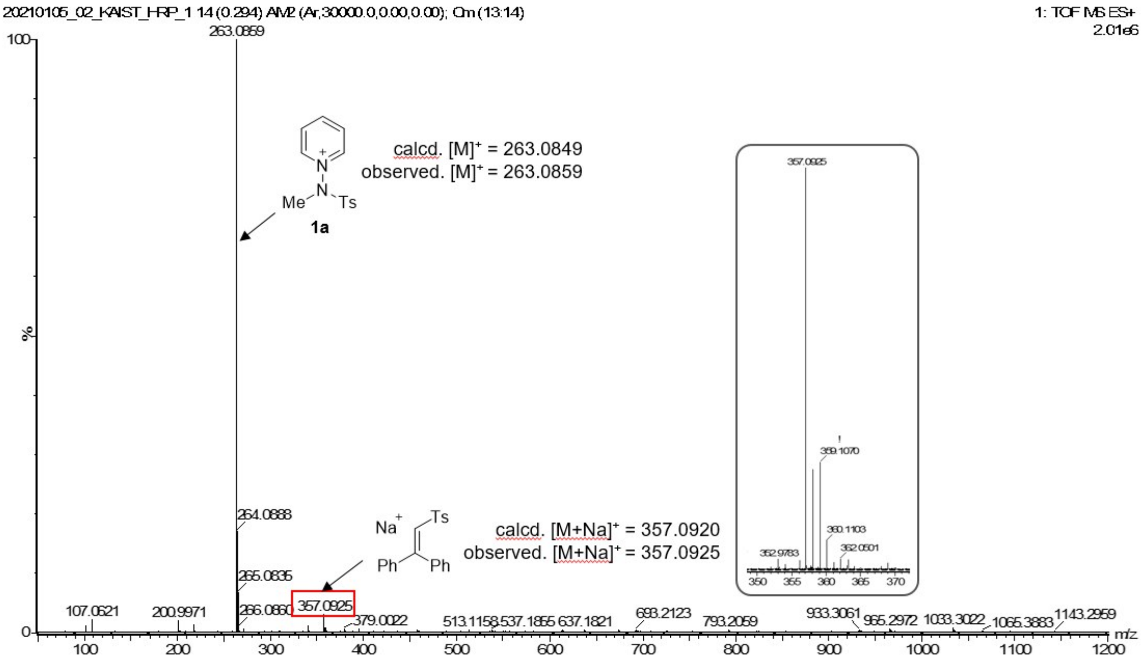

Fig. S12. Phenylsulfonyl-diphenylethene adduct detected on HR-MS.

NMR analysis

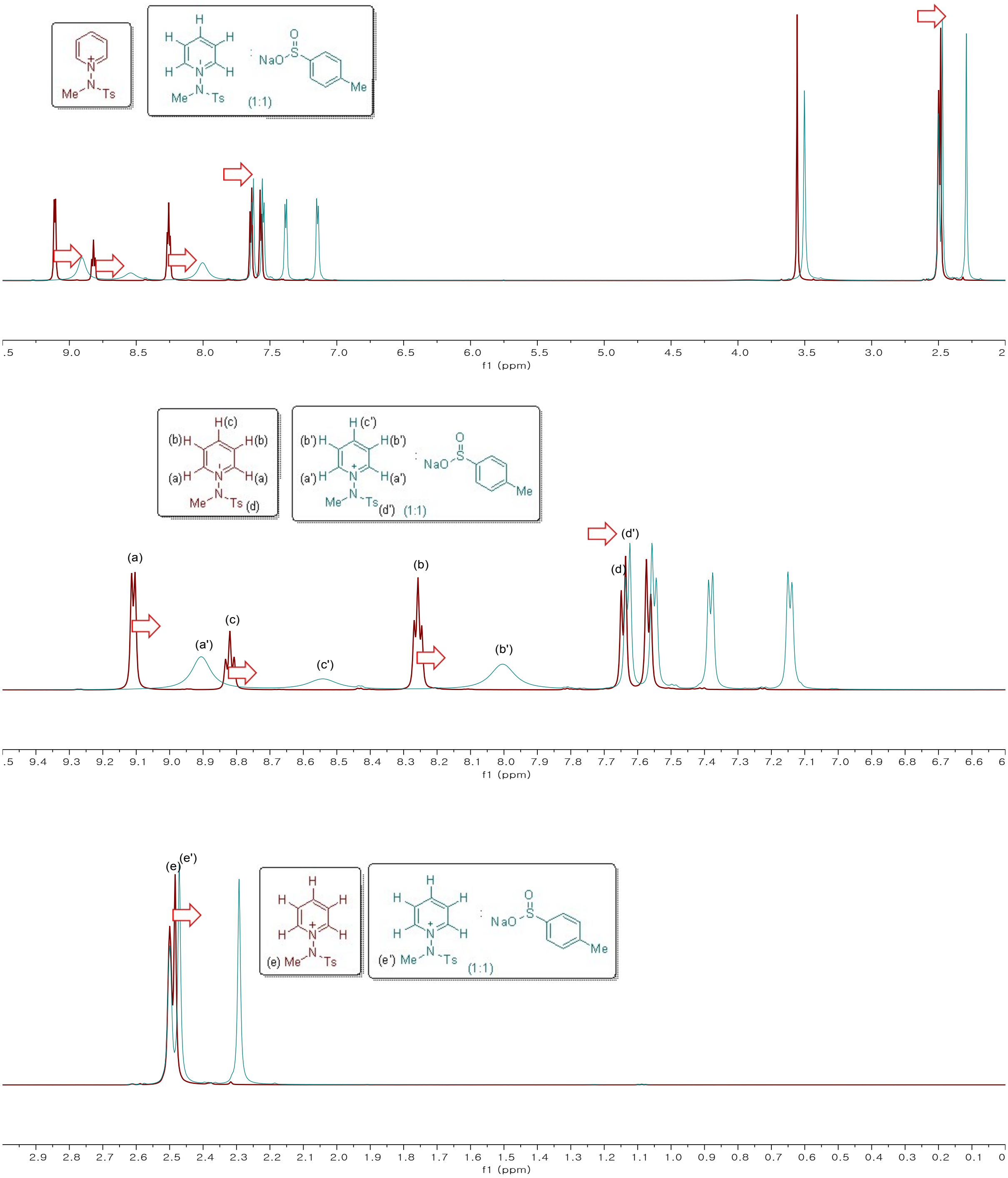

Fig. S13. ^{1}H NMR spectrum overlay of pyridinium salt **1a** and the mixture of **1a** and **2a** (0.1 M in DMSO)

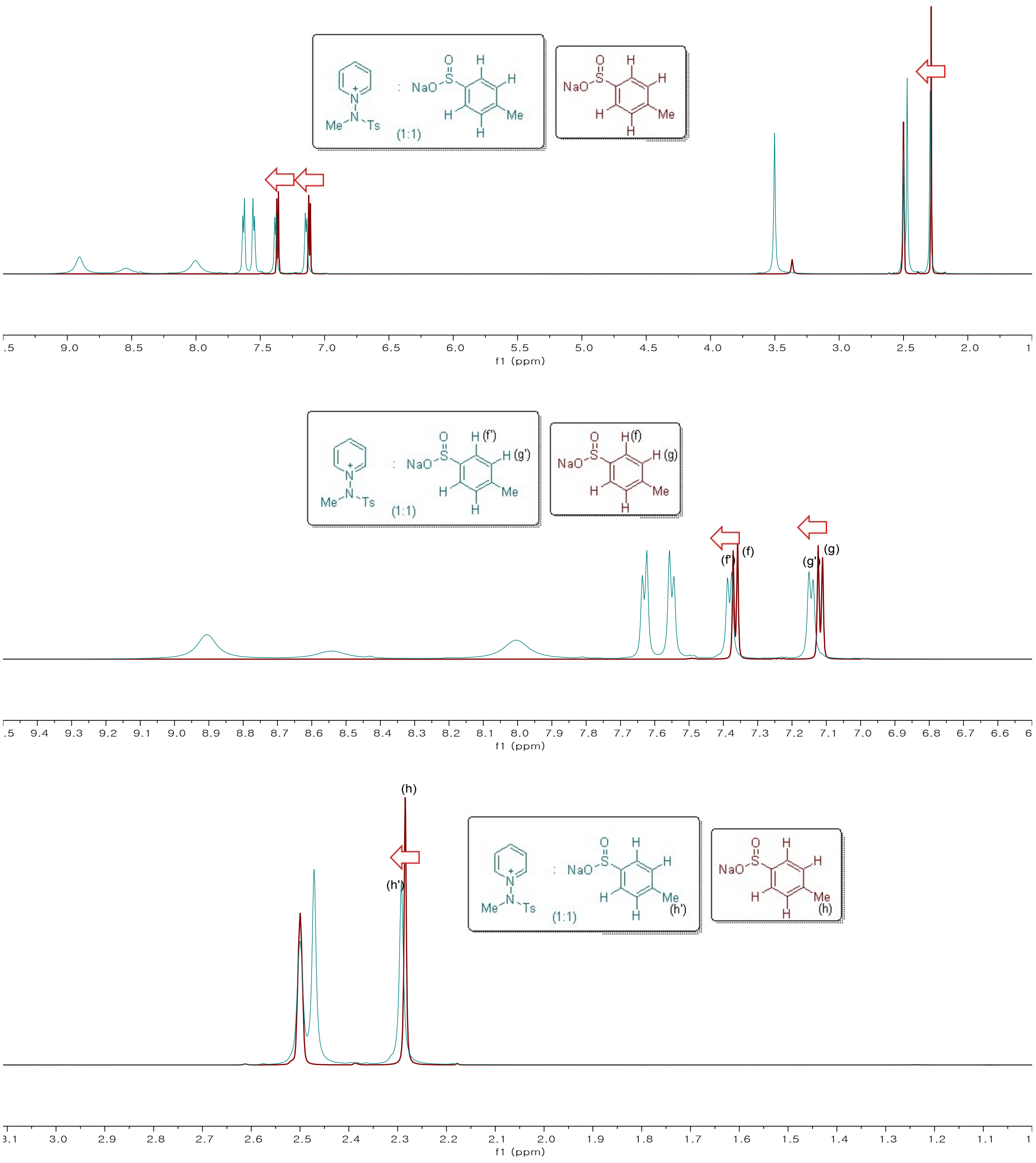

Fig. S14. ^{1}H NMR spectrum overlay of sulfinate **2a** and the mixture of **1a** and **2a** (0.1 M in DMSO)

Job's analysis.

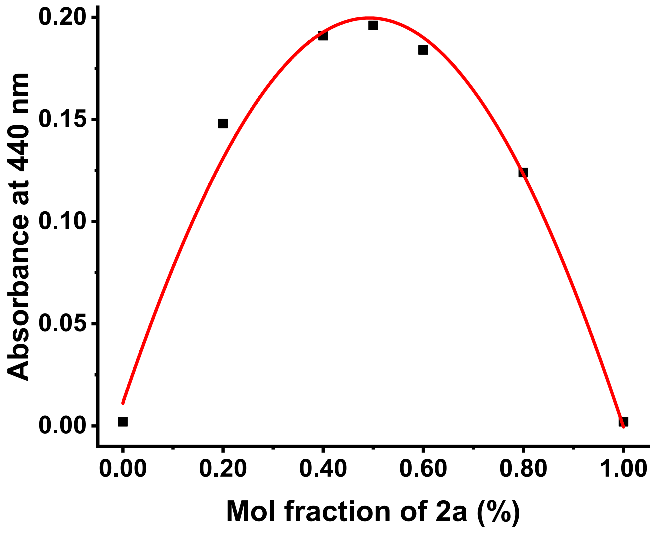

Fig. S15. Job's plot for the ratio between pyridinium salt **1a** and sulfinate **2a** using UV-Vis absorption spectrometry.

Quantum Yield Measurements

Determination of the light intensity at 440nm

According to the procedure reported by Yoon,[4] the photon flux of the LED (λ_{max} = 440 nm) was measured by standard ferrioxalate actinometry. A 0.15 M solution of ferrioxalate was prepared by dissolving potassium ferrioxalate hydrate (0.737 g) in H_2SO_4 (10 mL of a 0.05 M solution). A buffered solution of 1,10-phenanthroline was prepared by dissolving 1,10-phenanthroline (5.0 mg) and sodium acetate (1.13 g) in H_2SO_4 (5.0 mL of a 0.5 M solution). Both solutions were stored in the dark. To determine the photon flux of the LED, the ferrioxalate solution (2.0 mL) was placed in a cuvette and irradiated for 90 seconds at λ_{max} = 440 nm. After irradiation, the phenanthroline solution (0.35 mL) was added to the cuvette and the mixture was allowed to stir in the dark for 1 h to allow the ferrous ions to completely coordinate to the phenanthroline. The absorbance of the solution was measured at 510 nm. A non-irradiated sample was also prepared and the absorbance at 510 nm was measured. Conversion was calculated using eq 1.

$$mol\ of\ Fe^{2+} = \frac{V \cdot \Delta A_{510\ nm}}{l \cdot \epsilon} = \frac{(0.00235\ L) \cdot (2.41)}{(1.00\ cm) \cdot (11{,}100 \frac{L}{mol} \cdot cm)} = 5.10 \times 10^{-7}\ mol \qquad (1)$$

V is the total volume (0.00235 L) of the solution after addition of phenanthroline, ΔA is the difference in absorbance at 510 nm between the irradiated and non-irradiated solutions, l is the path length (1.00 cm), and ε is the molar absorptivity of the ferrioxalate actinometer at 510 nm (11,100 $Lmol^{-1}cm^{-1}$).[5] The photon flux can be calculated using eq 2.

$$Photon\ flux = \frac{mol\ of\ Fe^{2+}}{\emptyset \cdot t \cdot f} = \frac{5.10 \times 10^{-7}\ mol}{(1.01) \cdot (90\ s) \cdot (0.992)} = 5.66 \times 10^{-9}\ einstein{\cdot}s^{-1} \qquad (2)$$

Where Φ is the quantum yield for the ferrioxalate actinometer (1.12 at λ = 440 nm)[6] the irradiation time (90 s), and f is the fraction of light absorbed at 440 nm by the ferrioxalate actinometer. This value is calculated using eq 3 where $A_{440\ nm}$ is the absorbance of the ferrioxalate solution at 440 nm. An absorption spectrum gave an $A_{440\ nm}$ value of >3, indicating that the fraction of absorbed light (f) is 0.999.

$$f = 1 - 10^{-A_{440\ nm}} \qquad (3)$$

The photon flux was thus calculated (average of three experiments) to be 5.66 × 10^{-9} einstein·s^{-1}

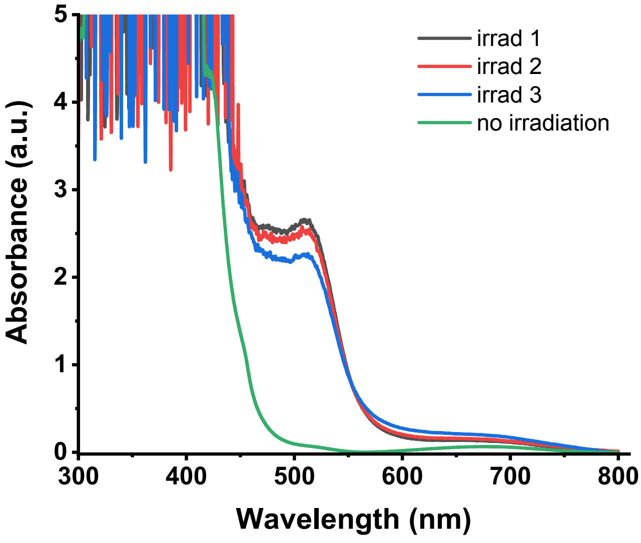

Fig. S16. Absorption spectra of three irradiation experiments and non-irradiation experiment.

Measurement of the reaction quantum yield

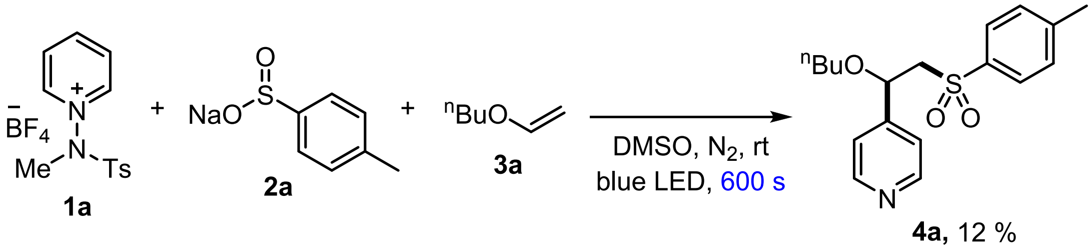

To a 12 mL test tube equipped with a magnetic bar were added **1a** (1.5 equiv, 0.15 mmol) and **2a** (1.5 equiv, 0.15 mmol), and the tube sealed with a PTFE septum. After evacuating the tube and back-filling with nitrogen, **3a** (1.0 equiv, 0.10 mmol) and DMSO (1.0 mL) were added via syringe. The reaction mixture was stirred upon irradiation using blue LED (λ_{max} = 440 nm) for 600 s. After completion, the reaction mixture was diluted with ethylacetate (30 mL), and washed with water (20 mL x 2) and brine (20 mL). The yield of product **4a** was measured to be 12% (12×10^{-6} mol of **4a**) by ^{1}H NMR analysis using 1,1,2,2-tetrabromoethane. The reaction quantum yield (Φ) was calculated using eq 4 where the photon flux is 5.66×10^{-9} einstein·s^{-1} (measured by actinometry as described above), t is the reaction time (600 s) and f is the fraction of incident light absorbed by the catalyst, determined using eq 3. An absorption spectrum of the reation mixture gave an absorbance value of 1.77 at 440 nm (**Fig. S4**), indicating that the fraction of light absorbed by the photocatalyst (f) is 0.983.

$$\Phi R = \frac{mol\ of\ product}{flux \cdot t \cdot f} \tag{4}$$

$$\Phi R = \frac{13 \times 10^{-6}\, mol}{5.66 \times 10^{-9}\, einstein{\cdot}s^{-1} \cdot 300\, s \cdot 0.983} = 3.60$$

The reaction quantum yield (Φ) was calculated to be 3.60.

Reaction profile

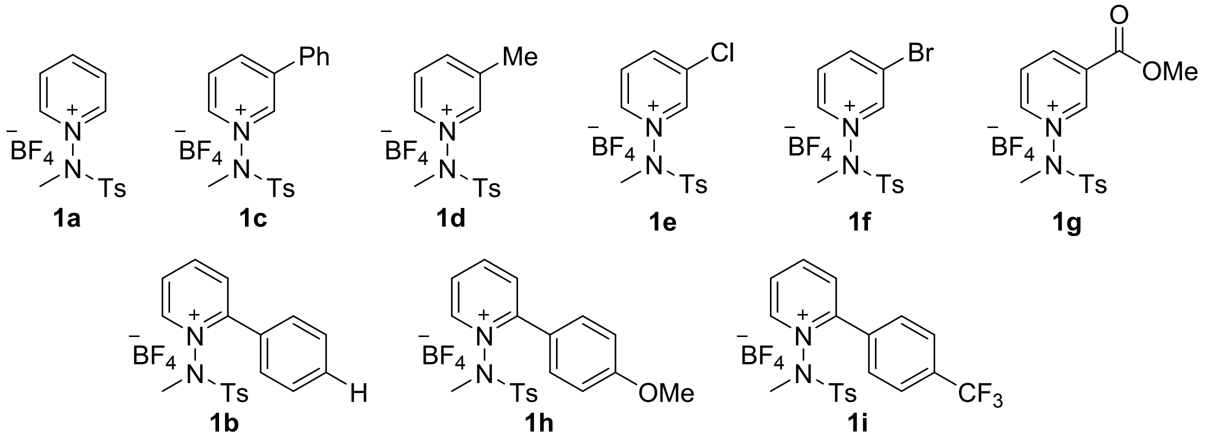

Fig. S17. Structure of pyridinium salts **1a**-**1i**..

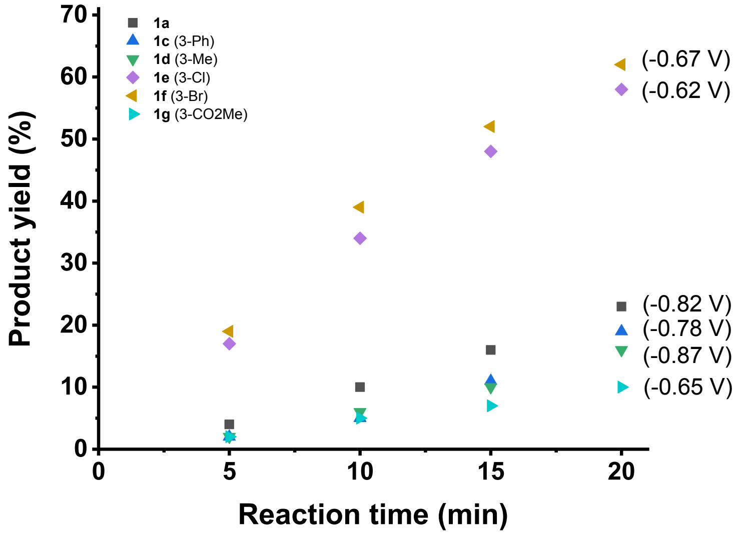

	1a	1c	1d	1e	1f	1g
E^{red} = (vs. SCE) [V]	(-0.82 V)	(-0.78 V)	(-0.87 V)	(-0.62 V)	(-0.67 V)	(-0.65 V)

Fig. S18. Reaction profile of **1a** and C3-substituted pyridinium salts (**1c**, **1d**, **1e**, **1f** and **1g**).

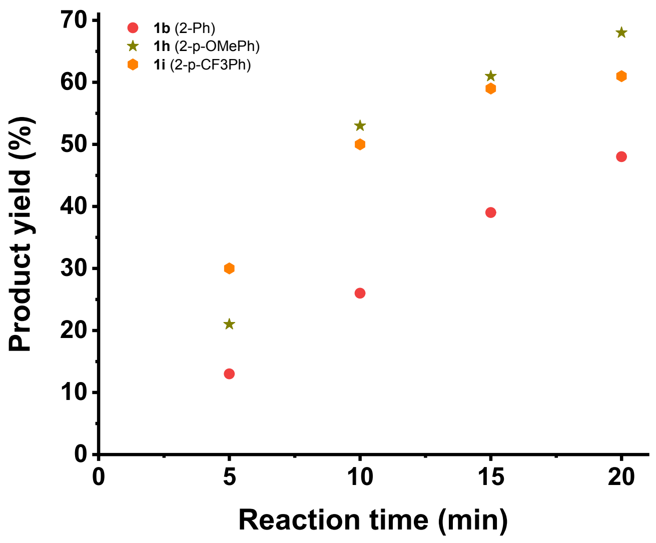

Fig. S19. Reaction profile of C2-substituted pyridinium salts (**1b**, **1h** and **1i**).

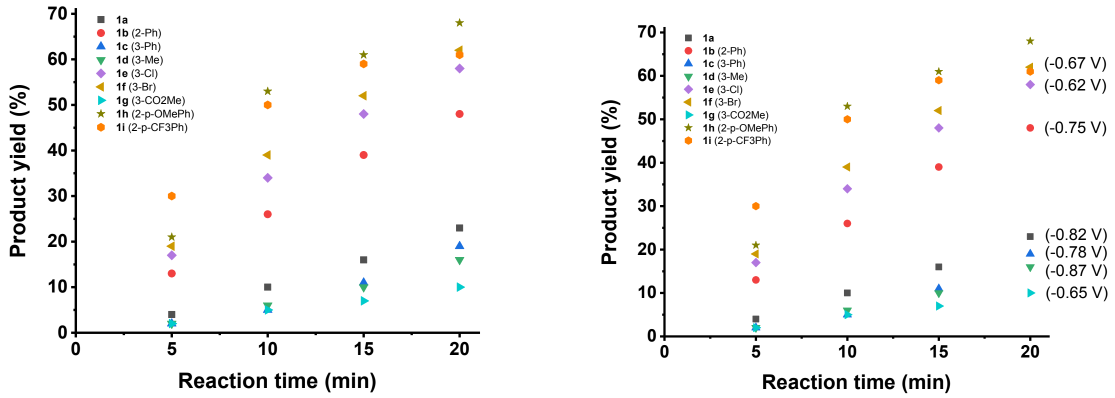

Fig. S20. Overall reaction profile of **1a-1i**.

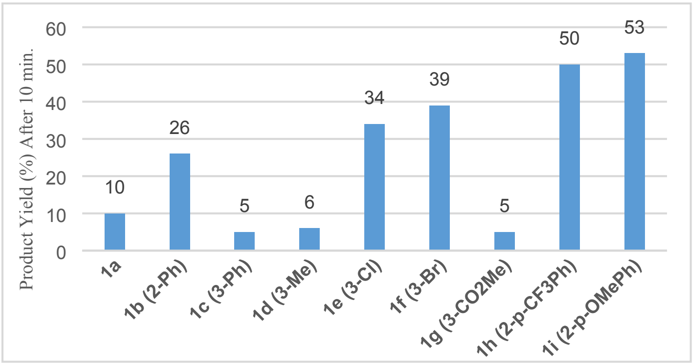

Fig. S21. Yield of product **4** after 10 min.

(b) Control experiments for two-component reactions

Without base

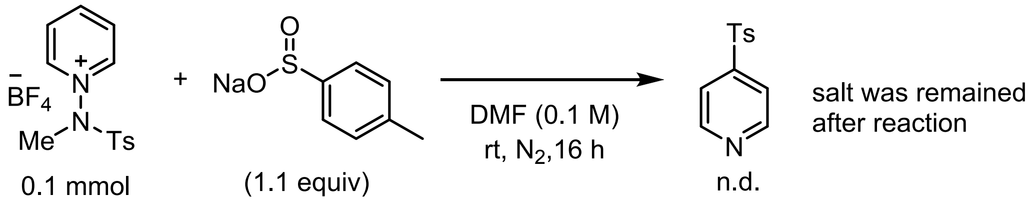

Competition experiments

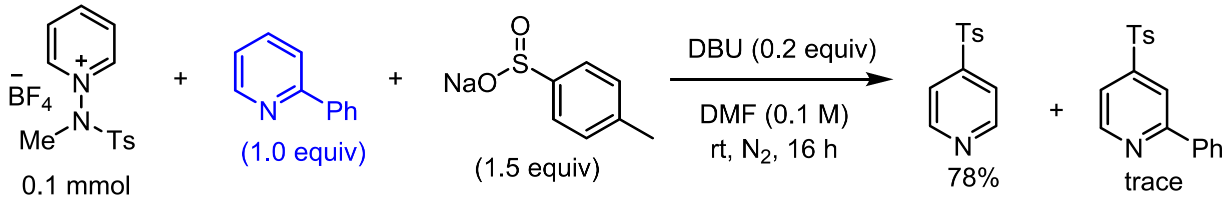

Under Air

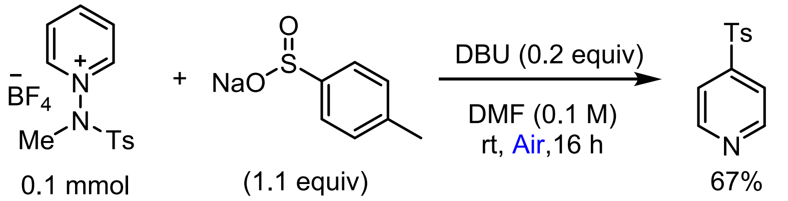

With TEMPO

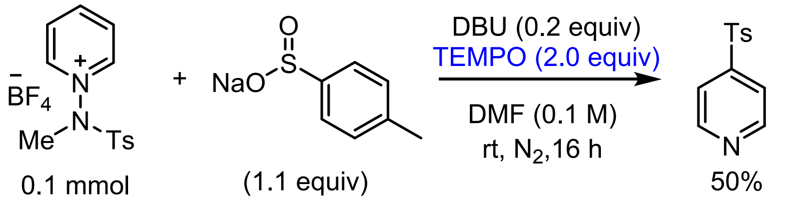

With C4-blocked pyridinium salts

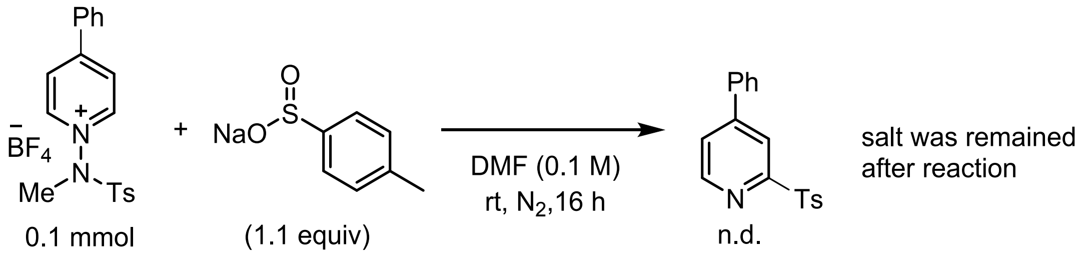

Reaction with other nucleophiles

(a) For Nu = NaCN:

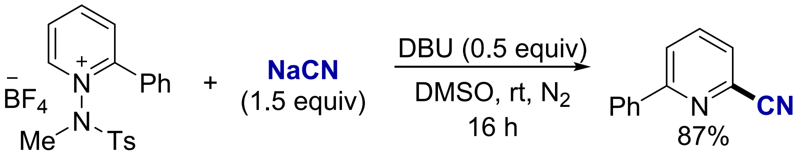

To a 12 mL test tube equipped with a magnetic bar were added 2-phenyl-substituted pyridinium salt **1b** (1.0 equiv, 0.05 mmol) and sodium cyanide (1.5 equiv, 0.075 mmol), and the tube was sealed with a PTFE septum. After evacuating the tube and back-filling with nitrogen, DMSO (0.5 mL) and DBU (0.5 equiv, 0.05 mmol) were added via syringe at 0 °C. The resulting mixture was stirred at room temperature for 16 h and then analyzed by LC-MS. The yield of the desired product was measured to be 87% by ^{1}H-NMR analysis of the crude mixture using 1,1,2,2,-tetrabromoethane as an internal standard by comparison with that of the authentic product reported in literature.[7]

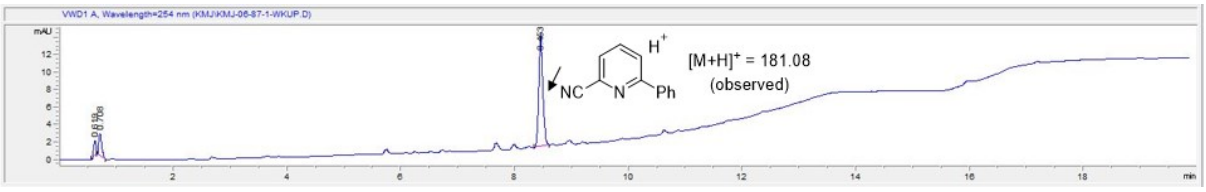

Fig. S22. LC-MS spectra of the crude mixture.

(b) For Nu = Et_2Zn:

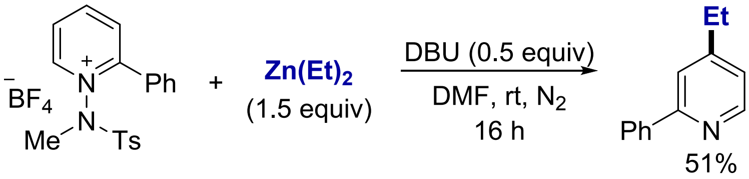

To a 12 mL test tube equipped with a magnetic bar was added 2-phenyl-substituted pyridinium salt **1b** (1.0 equiv, 0.05 mmol) and the tube was sealed with a PTFE septum. After evacuating the tube and back-filling with nitrogen, DMF (0.5 mL), diethylzinc (1.0 M in hexanes, 1.5 equiv, 0.075 mmol) and DBU (0.5 equiv, 0.05 mmol) were added via syringe at 0 °C. The resulting mixture was stirred at room temperature for 16 h and then analyzed by LC-MS. The yield of the desired product was measured to be 51% by ^{1}H-NMR analysis of the crude mixture using 1,1,2,2-tetrabromoethane as an internal standard by comparison with that of the authentic product reported in literature.[8]

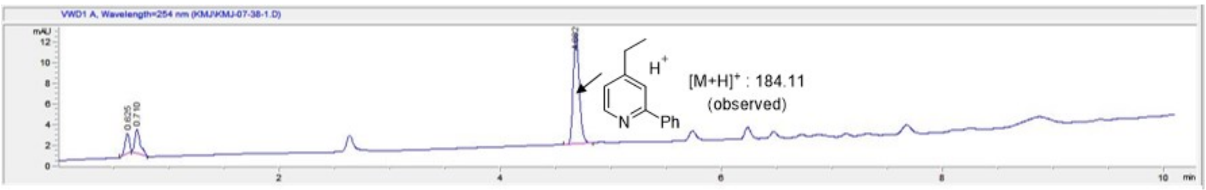

Fig. S23. LC-MS spectra of the crude mixture.

KIE Experiments

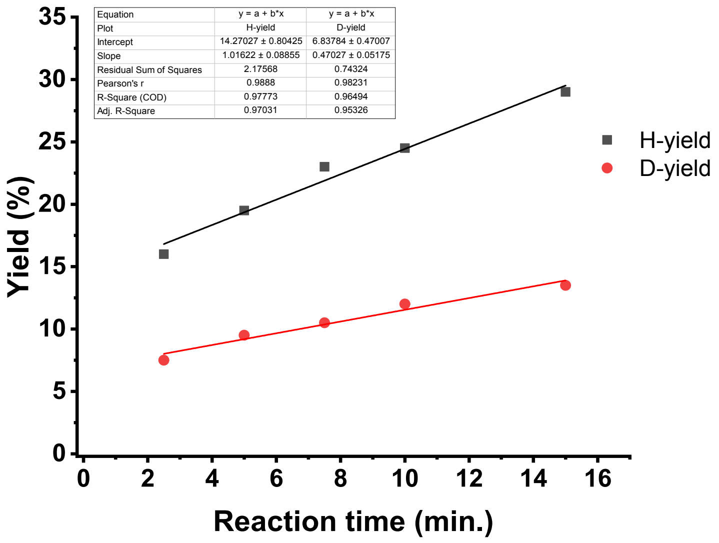

$$k_H/k_D = 2.16$$

Fig. S24. Measurement of kinetic isotope effect (two parallel reactions) on pyridyl ring[9]

The reactions for KIE measurements were conducted following to **GP2**.

VI. Synthetic Utility.

(a) Synthetic transformation of β-sulfonylated pyridine

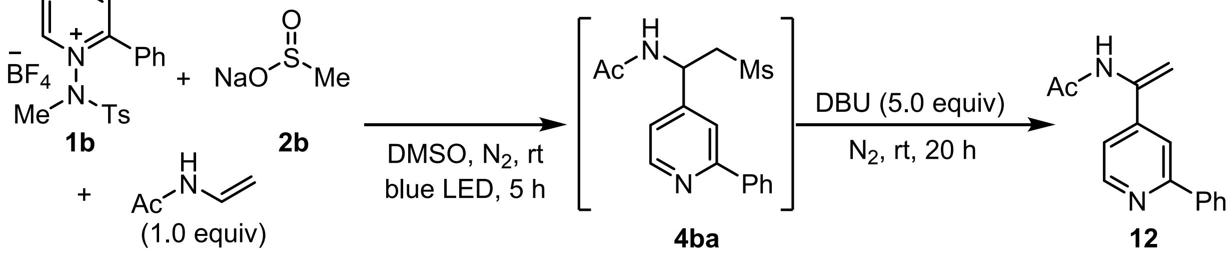

To a 12 mL test tube equipped with a magnetic bar were added **1b** (1.5 equiv, 0.225 mmol) and **2b** (2 equiv, 0.30 mmol), and the tube sealed with a PTFE septum. After evacuating the tube and back-filling with nitrogen, DMSO (1.5 mL) and *N*-vinylacetamide (1.0 equiv, 0.15 mmol) were added via syringe. The sealed test tube was placed at the reaction bath equipped with Kessill PR1160-440 nm blue LEDs (25% intensity). The reaction mixture was stirred at room temperature for 8 h. After reaction completion, the reaction mixture was diluted with EtOAc (30 mL), then washed with water (20 mL). Combined aqueous layer was extracted with EtOAc (20 mL x 4). The organic layer was dried over anhydrous sodium sulfate, filtered, and concentrated under reduced pressure. The crude mixture was purified by flash column chromatography on silica gel (eluent: MeOH/CH_2Cl_2 = 1:20) to give the desired product **4ba** (72%).

To a 12 mL test tube equipped with a magnetic bar were added **1b** (1.5 equiv, 0.15 mmol) and **2b** (1.5 equiv, 0.15 mmol), and the tube sealed with a PTFE septum. After evacuating the tube and back-filling with nitrogen, DMSO (1.0 mL) and *N*-vinylacetamide (1.0 equiv, 0.1 mmol) were added via syringe. The sealed test tube was placed at the reaction bath equipped with Kessill PR1160-440 nm blue LEDs (25% intensity). The reaction mixture was stirred at room temperature for 5 h. After reaction completion, DBU (5 equiv, 0.5 mmol) was added to the reaction mixture via microsyringe in the water bath. The reaction mixture was stirred at room temperature for 20 h. After reaction completion, the reaction mixture was diluted with EtOAc (30 mL), then washed with water (20 mL x 2) and brine (20 mL). The organic layer was dried over anhydrous sodium sulfate, filtered, and concentrated under reduced pressure. The crude mixture was purified by flash column chromatography on silica gel (eluent: CH_2Cl_2/EtOAc = 2:1) to give the desired product **12** (51% for one-pot, 2-steps).

(b) Synthetic transformation of C4-sulfonylated pyridines

Synthesis of 4-pyridine ethers

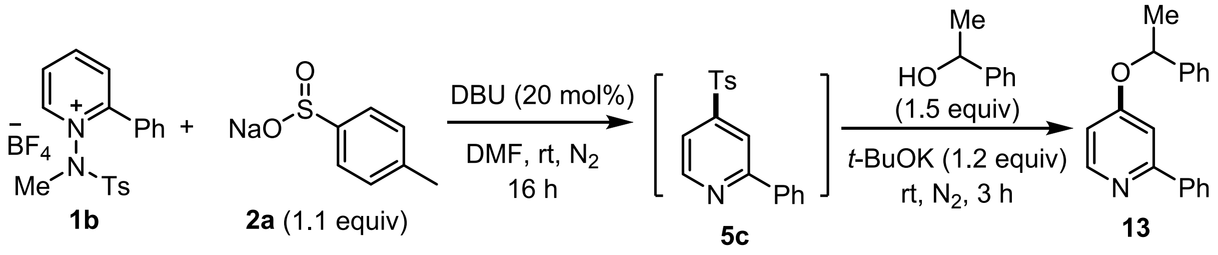

To a 12 mL test tube equipped with a magnetic bar were added **1b** (1.0 equiv, 0.1 mmol) and **2a** (1.1 equiv, 0.11 mmol), and the tube sealed with a PTFE septum. After evacuating the tube and back-filling with nitrogen, DMF (1.0 mL) and DBU (0.2 equiv, 0.02 mmol) were added at 0 °C. The reaction mixture was stirred at room temperature for 16 h. To the reaction mixture, the stock solution of 1-phenylethanol 0.75 M in DMF (1.5 equiv, 0.15 mmol) and *t*BuOK 1 M in THF (1.2 equiv, 0.12 mmol) were added at 0 °C. The reaction mixture was stirred at room temperature for 3 h. After reaction completion, the reaction mixture was diluted with EtOAc (30 mL), and then washed with water (20 mL x 2) and brine (20 mL). The organic layer was dried over anhydrous sodium sulfate, filtered, and concentrated under reduced pressure. The crude mixture was purified by flash column chromatography on silica gel (eluent: acetone/*n*-hexane = 1:4) to give the desired product **13** (61% for one-pot, 2-steps).

Synthesis of potassium pyridinesulfinate 14 and pyridiylsulfonamide 15

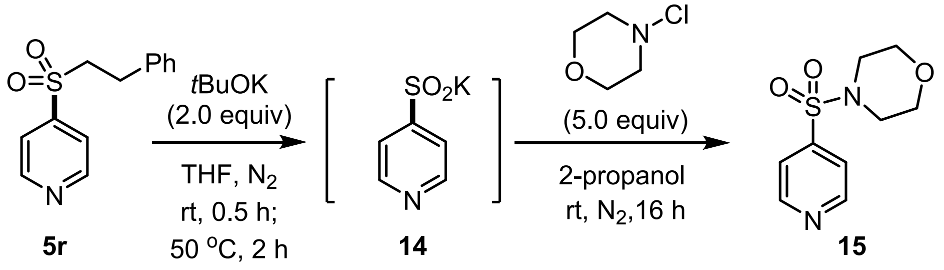

To a 12 mL test tube equipped with a magnetic bar were added **5r** (1.0 equiv, 0.1 mmol) and the tube sealed with a PTFE septum. After evacuating the tube and back-filling with nitrogen, THF (0.5ml) and *t*BuOK 1 M in THF (2.0 equiv, 0.2 mmol) were added at 0 °C. The reaction mixture was stirred at room temperature for 30 min and then stirred at 50 °C for 2 h. After reaction completion, the reaction mixture of **14** was quenched with water (0.1 ml) and the solvent was evaporated under reduced pressure and vacuum overnight. After evacuating the tube and back-filling with nitrogen, anhydrous 2-propanol (0.8 mL) and *N*-chloromorpholine (5.0 equiv, 0.5 mmol) were added to the crude mixture of **14.** The reaction

mixture was stirred at room temperature for 16 h. After reaction completion, the reaction mixture was concentrated under reduced pressure. The crude mixture was purified by flash column chromatography on silica gel (eluent: MeOH/CH_2Cl_2 = 1:30) to give the desired product **15** (85% for one-pot, 2-steps).

VI. Computational studies.

All calculations except single point calculations were conducted using DFT[10] as implemented in the Jaguar 9.1 suite[11] of ab initio quantum chemistry programs with B3LYP-D3 levels of theory.[12] Geometry optimizations were proceeded using the LACVP** basis set. Analytical vibrational frequencies within the harmonic approximation were calculated using the LACVP** basis to confirm proper convergence to well-defined minima or saddle points on the potential energy surface. Solvation energies were calculated using a self-consistent reaction field (SCRF)[13-15] approach based on accurate numerical solutions of the Poisson-Boltzmann equation and were performed with the LACVP** basis at the optimized gas-phase geometry with the ε =46.48 for dimethylsulfoxide. As is the case for all continuum models, the solvation energies are subject to the empirical parametrization of the atomic radii that are used to generate the solute surface. The standard set of optimized radii in Jaguar was used for H (1.150 Å), C (1.900 Å), N (1.600 Å), O (1.600 Å), and S (1.900 Å).[16] At last, the energies of the optimized structures were reevaluated by additional single point calculations on each optimized geometry using DFT as implemented in ORCA 4.0.1 suite[17] of ab initio quantum chemistry programs with Truhlar's PW6B95 functinoal[18] including Grimme's D3 dispersion correction and Weigend's quadruple-Z quality basis set def2-QZVP.[19],[20] The Gibbs free energies in solution phase G(sol) were computed with the following protocol.

$$G(sol) = G(gas) + G^{solv} \quad (5)$$

$$G(gas) = H(gas) – TS(gas) \quad (6)$$

$$H(gas) = E(SCF) + ZPE \quad (7)$$

$$\Delta E(SCF) = \Sigma E(SCF) \text{ for products} – \Sigma E(SCF) \text{ for reactants} \quad (8)$$

$$\Delta G(sol) = \Sigma G(sol) \text{ for products} – \Sigma G(sol) \text{ for reactants} \quad (9)$$

G(gas) is the free energy in gas phase; G^{solv} is the free energy of solvation; H(gas) is the enthalpy in gas phase; T is the temperature (298.15K); S(gas) is the entropy in gas phase; E(SCF) is "raw" electronic energy as computed from the SCF procedure which is the self-consistent field energy, and ZPE is the zero point energy. The entropy we refer is specifically vibrational/rotational/translational entropy of the solute(s), and the entropy of the solvent is implicitly comprised in the continuum solvation +-+-model.

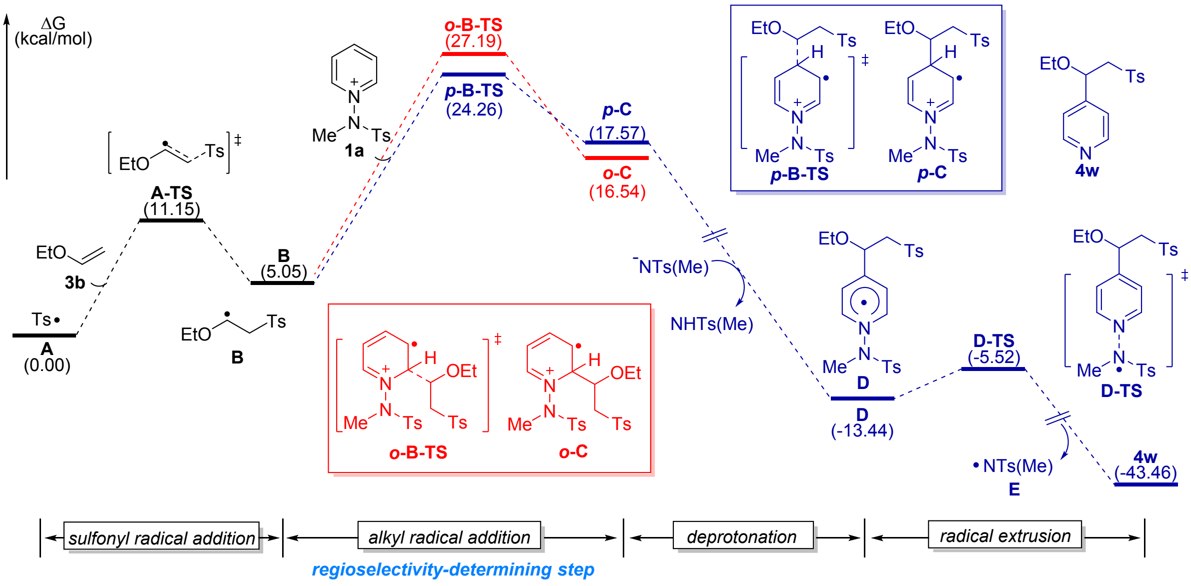

Fig. S25. Energy diagram of the reaction pathway.

$$\bullet\,\text{NTsMe} + \text{Ts}^{-} \longrightarrow {}^{-}\text{NTsMe} + \text{Ts}\,\bullet \quad (\Delta G = -5.79 \text{ kcal/mol})$$

Scheme S3. Energy profile of SET process for chain process.

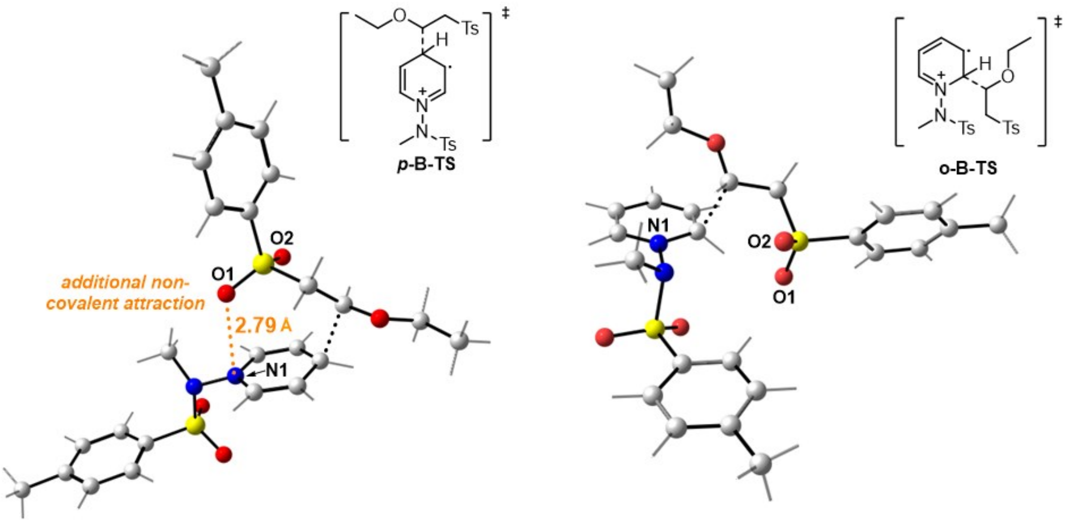

Fig. S26. Optimized structures of ***p*-B-TS** and ***o*-B-TS**.

An additional non-covalent attraction between the sulfonyl oxygens and the nitrogens of the pyridinium substrate across a distance of 2.79A in the key geometry ***p*-B-TS**, as illustrated in **Fig. S14**.[1] This interaction is structurally not possible in ***o*-B-TS**.

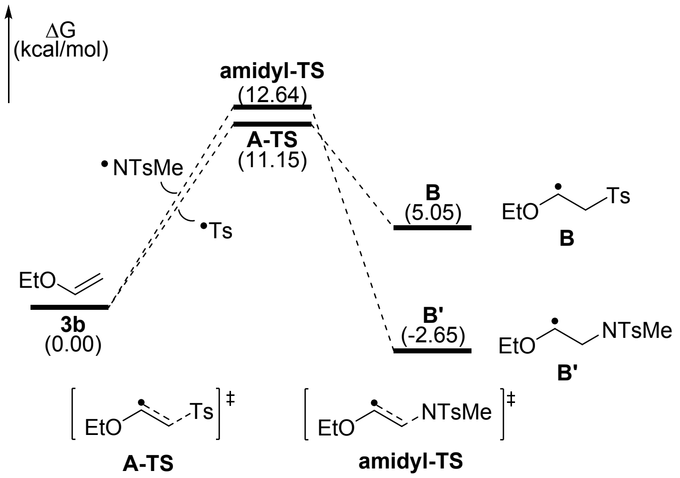

Fig. S27. Calculated free energy of radical addition step of tosyl radical **A** and amidyl radical **E**.

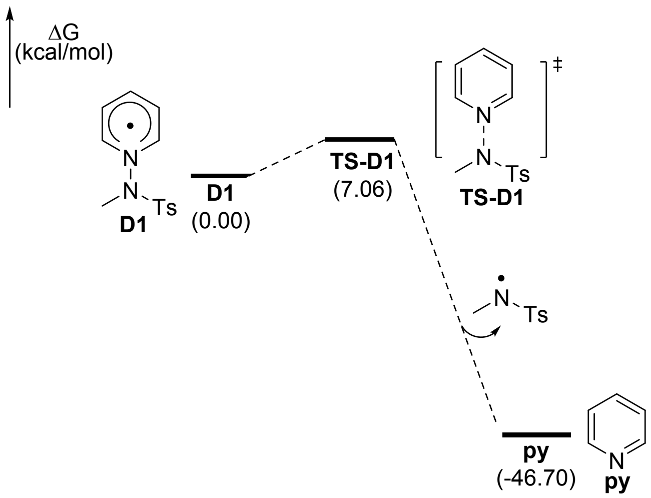

Fig. S28. Calculated free energy of *N-N* bond cleavage of **D1.**

TD-DFT calculations

The ground state geometry of EDA complex between **1a** and **2a** was analyzed by time-dependent density functional theory (TDDFT) at the CAM-B3LYP def2-TZVPP level using the ORCA 4.0.1 software package. The first 10 excited states are reported below.

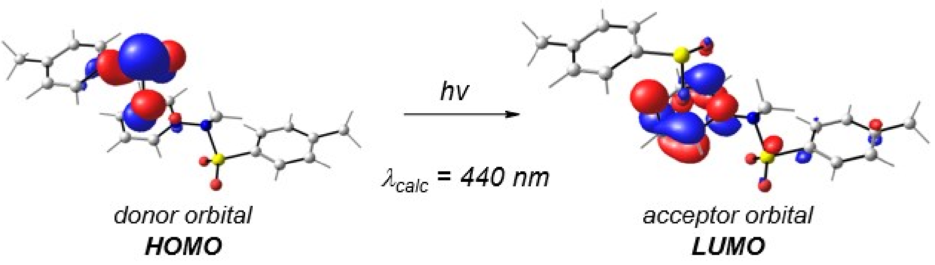

Fig. S29. TDDFT analysis.

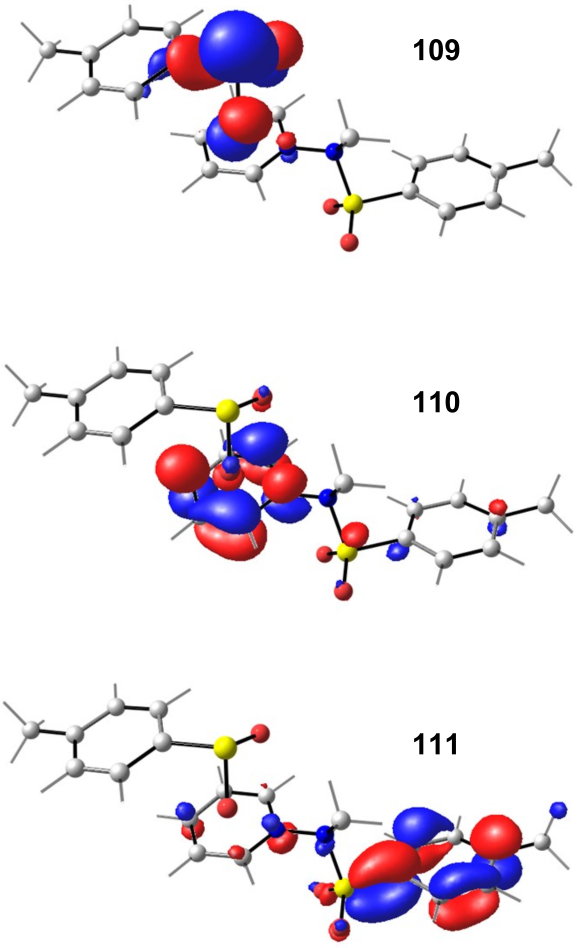

Fig. S30. Calclated molecular orbitals 109, 110 and 111 representing the orbitals of the sulfinate **2a** HOMO and pyridinium salt **1a** LUMO & LUMO+1.

Table 1. Cartesian coordinates of the optimized geometries The cartesian coordinates of optimized geometries are given below in the standard XYZ format, and units are in Å.

==============================

EDA complex between **1a** and **2a**

==============================

C	-0.301262736	1.502207041	-3.166728973
C	-0.380594969	2.583126545	-2.299488306
C	-0.505783558	2.362147093	-0.935169339
N	-0.695883274	1.089458823	-0.490843982
C	-0.507638276	0.013585041	-1.301474333
C	-0.335385919	0.199649662	-2.652983904
H	-0.144457102	1.663980484	-4.227482319
H	-0.278042227	3.601510048	-2.651088715
H	-0.607332945	3.147691011	-0.202977598
H	-0.542868018	-0.944664598	-0.807003498
H	-0.193979621	-0.663918257	-3.290155649
N	-0.940707028	0.806415558	0.865653276
C	0.180686265	1.136377931	1.774026036
H	1.033304095	0.553546429	1.426086903
H	0.437471479	2.197408438	1.763268828
H	-0.118311226	0.817929983	2.774509430
S	-2.508940935	1.426156878	1.324908495
O	-3.387772799	1.078862429	0.207462400
O	-2.385858774	2.814078808	1.789103866
C	-2.838150501	0.372981787	2.720792532
C	-2.791860104	0.904423237	4.009132862
C	-3.148836851	-0.969799399	2.495190620
H	-2.561906815	1.954904914	4.150141716
H	-3.194965839	-1.354316950	1.482068539
C	-3.051525831	0.064328223	5.090361595
C	-3.403304338	-1.791126132	3.588375807
H	-3.020085335	0.468985528	6.098109245
H	-3.646848202	-2.837208748	3.424440622
C	-3.352819443	-1.291469574	4.899699211
C	-3.588383198	-2.202742815	6.078673840
H	-4.021194458	-1.660825729	6.924378395
H	-4.257734299	-3.028223276	5.820267200
H	-2.642576218	-2.641857386	6.419691086
H	3.093187809	3.973624229	-4.956826210
C	3.354908943	1.993121147	-6.821552277
H	2.880227804	2.906634808	-7.193884373
H	2.918653965	1.142283201	-7.354926109
H	4.414761543	2.041420937	-7.101392746
C	3.194127321	1.852847338	-5.325490952
C	3.174682140	0.588114440	-4.720011711
C	3.054161310	0.454250872	-3.336053610
C	2.939384222	1.591030121	-2.538250923
C	2.969103575	2.858152866	-3.118666172
C	3.089303970	2.984375954	-4.503242493
H	3.246875763	-0.300690204	-5.343965530
H	3.002825260	-0.522654116	-2.864681959
S	2.763382435	1.417724490	-0.709389508
H	2.850548029	3.730251074	-2.482089043
O	1.846264720	2.614415646	-0.368074656
O	2.063569784	0.053894084	-0.594489038

Table 2. Vibrational frequencies (in cm^{-1}) of the optimized structures.

==============================

EDA complex between 1a and **2a**

==============================

7.02	18.49	29.63	35.04	39.43	49.85
57.22	59.32	65.05	81.87	84.98	99.86
125.63	135.92	153.30	157.81	176.03	187.62
198.77	230.50	246.98	257.81	271.39	289.59
301.99	327.09	329.93	339.91	358.45	393.55
401.92	409.29	415.89	421.46	439.51	452.63
470.41	484.63	498.33	531.89	566.69	569.50
574.84	612.26	644.36	646.98	648.06	656.17
670.50	693.27	716.38	726.21	750.55	809.07
811.13	817.49	827.87	834.75	840.14	856.43
865.23	913.95	931.25	945.02	971.33	972.59
980.40	987.91	991.71	1012.28	1019.83	1021.65
1033.71	1035.11	1042.25	1055.33	1062.85	1064.80
1074.43	1094.09	1094.98	1123.07	1143.38	1146.56
1155.80	1161.73	1192.70	1210.96	1213.26	1223.51
1226.54	1233.41	1236.83	1247.27	1324.16	1329.72
1341.90	1347.56	1355.96	1358.40	1375.59	1426.95
1428.02	1435.90	1444.94	1467.91	1497.76	1501.99
1502.98	1503.92	1504.63	1505.93	1520.15	1524.89
1533.11	1538.60	1600.74	1625.90	1629.68	1652.44
1655.10	1676.50	3033.59	3041.24	3069.96	3092.21
3104.71	3116.68	3133.01	3161.57	3162.68	3163.71
3178.13	3188.19	3189.71	3197.84	3201.67	3217.73
3219.88	3223.28	3240.69	3245.41	3292.08	3295.83

ABSORPTION SPECTRUM VIA TRANSITION ELECTRIC DIPOLE MOMENTS

State	Energy (cm-1)	Wavelength (nm)	fosc	T2 (au**2)	TX (au)	TY (au)	TZ (au)
1	19919.0	502.0	0.000000000	0.00000	0.00000	0.00000	-0.00000
2	24392.3	410.0	0.116909530	1.57788	-1.24017	-0.14987	0.13187
3	27882.9	358.6	0.000000000	0.00000	0.00000	0.00000	0.00000
4	28940.7	345.5	0.000000000	0.00000	0.00000	-0.00000	0.00000
5	29651.9	337.2	0.001687678	0.01874	-0.11888	-0.00771	-0.06742
6	30824.1	324.4	0.000000000	0.00000	-0.00000	-0.00000	-0.00000
7	31439.4	318.1	0.000000000	0.00000	-0.00000	-0.00000	-0.00000
8	33116.4	302.0	0.000000000	0.00000	-0.00000	-0.00000	0.00000

```
   9    33371.1      299.7    0.000000000    0.00000    0.00000    0.00000   -0.00000
  10    34325.0      291.3    0.020382487    0.19549   -0.16734   -0.40763    0.03643
-----------------------------------------------------------------------------------------------------------------


-----------------------------------------
TD-DFT/TDA EXCITED STATES
-----------------------------------------
the weight of the individual excitations are printed if larger than 0.01
STATE   1:  E=   0.090757 au      2.470 eV    19919.0 cm**-1
   109a -> 110a  :     0.451461 (c=  0.67190876)
   109a -> 111a  :     0.032131 (c= -0.17925115)
   109b -> 110b  :     0.451461 (c=  0.67190876)
   109b -> 111b  :     0.032131 (c= -0.17925115)

STATE   2:  E=   0.111139 au      3.024 eV    24392.3 cm**-1
   109a -> 110a  :     0.459166 (c=  0.67761767)
   109a -> 111a  :     0.025631 (c= -0.16009800)
   109b -> 110b  :     0.459166 (c= -0.67761767)
   109b -> 111b  :     0.025631 (c=  0.16009800)

STATE   3:  E=   0.127044 au      3.457 eV    27882.9 cm**-1
   100a -> 110a  :     0.047779 (c=  0.21858473)
   101a -> 110a  :     0.034257 (c=  0.18508680)
   105a -> 110a  :     0.073456 (c= -0.27102825)
   107a -> 110a  :     0.277429 (c=  0.52671558)
   107a -> 111a  :     0.023438 (c= -0.15309448)
   100b -> 110b  :     0.047779 (c= -0.21858473)
   101b -> 110b  :     0.034257 (c= -0.18508680)
   105b -> 110b  :     0.073456 (c=  0.27102825)
   107b -> 110b  :     0.277429 (c=  0.52671558)
   107b -> 111b  :     0.023438 (c= -0.15309448)

STATE   4:  E=   0.131864 au      3.588 eV    28940.7 cm**-1
```

109a -> 112a : 0.056092 (c= -0.23683823)

109a -> 113a : 0.407515 (c= 0.63836863)

109b -> 112b : 0.056092 (c= 0.23683823)

109b -> 113b : 0.407515 (c= 0.63836863)

STATE 5: E= 0.135104 au 3.676 eV 29651.9 cm**-1

109a -> 112a : 0.058160 (c= 0.24116452)

109a -> 113a : 0.419299 (c= -0.64753266)

109b -> 112b : 0.058160 (c= 0.24116452)

109b -> 113b : 0.419299 (c= 0.64753266)

STATE 6: E= 0.140445 au 3.822 eV 30824.1 cm**-1

104a -> 114a : 0.010668 (c= 0.10328689)

106a -> 115a : 0.049352 (c= -0.22215414)

108a -> 114a : 0.123448 (c= -0.35135114)

109a -> 114a : 0.254355 (c= -0.50433598)

104b -> 114b : 0.010668 (c= -0.10328689)

106b -> 115b : 0.049352 (c= -0.22215414)

108b -> 114b : 0.123448 (c= 0.35135114)

109b -> 114b : 0.254355 (c= -0.50433598)

STATE 7: E= 0.143249 au 3.898 eV 31439.4 cm**-1

101a -> 111a : 0.014168 (c= 0.11902960)

102a -> 112a : 0.088506 (c= -0.29749927)

102a -> 113a : 0.011635 (c= -0.10786483)

103a -> 110a : 0.053299 (c= -0.23086528)

103a -> 111a : 0.280148 (c= -0.52929025)

101b -> 111b : 0.014168 (c= -0.11902960)

102b -> 112b : 0.088506 (c= -0.29749927)

102b -> 113b : 0.011635 (c= 0.10786483)

103b -> 110b : 0.053299 (c= -0.23086528)

103b -> 111b : 0.280148 (c= -0.52929025)

STATE 8: E= 0.150890 au 4.106 eV 33116.4 cm**-1

```
  99a -> 110a  :  0.041279 (c=  0.20317314)
 100a -> 113a  :  0.051420 (c=  0.22676066)
 101a -> 113a  :  0.034823 (c=  0.18660910)
 104a -> 110a  :  0.012211 (c= -0.11050548)
 105a -> 113a  :  0.050061 (c= -0.22374274)
 107a -> 112a  :  0.019840 (c= -0.14085368)
 107a -> 113a  :  0.146012 (c=  0.38211481)
 108a -> 110a  :  0.017149 (c=  0.13095518)
 108a -> 114a  :  0.012812 (c= -0.11319056)
 109a -> 113a  :  0.012808 (c= -0.11317054)
  99b -> 110b  :  0.041279 (c= -0.20317314)
 100b -> 113b  :  0.051420 (c= -0.22676066)
 101b -> 113b  :  0.034823 (c= -0.18660910)
 104b -> 110b  :  0.012211 (c=  0.11050548)
 105b -> 113b  :  0.050061 (c=  0.22374274)
 107b -> 112b  :  0.019840 (c=  0.14085368)
 107b -> 113b  :  0.146012 (c=  0.38211481)
 108b -> 110b  :  0.017149 (c= -0.13095519)
 108b -> 114b  :  0.012812 (c=  0.11319056)
 109b -> 113b  :  0.012808 (c= -0.11317054)
```

STATE 9: E= 0.152050 au 4.137 eV 33371.1 cm**-1

```
 106a -> 113a  :  0.011242 (c=  0.10602713)
 106a -> 115a  :  0.079720 (c=  0.28234759)
 107a -> 113a  :  0.013495 (c=  0.11616709)
 108a -> 114a  :  0.138579 (c=  0.37226244)
 109a -> 114a  :  0.163979 (c= -0.40494265)
 109a -> 130a  :  0.011170 (c=  0.10568836)
 106b -> 113b  :  0.011242 (c= -0.10602713)
 106b -> 115b  :  0.079720 (c=  0.28234759)
 107b -> 113b  :  0.013495 (c=  0.11616709)
 108b -> 114b  :  0.138579 (c= -0.37226245)
```

109b -> 114b : 0.163979 (c= -0.40494264)

109b -> 130b : 0.011170 (c= -0.10568836)

STATE 10: E= 0.156396 au 4.256 eV 34325.0 cm**-1

105a -> 110a : 0.012048 (c= 0.10976478)

107a -> 110a : 0.388284 (c= 0.62312423)

107a -> 111a : 0.025706 (c= -0.16033192)

108a -> 110a : 0.034559 (c= 0.18590115)

105b -> 110b : 0.012048 (c= 0.10976478)

107b -> 110b : 0.388284 (c= -0.62312423)

107b -> 111b : 0.025706 (c= 0.16033192)

108b -> 110b : 0.034559 (c= 0.18590115)

References

[1] Y. Moon, B. Park, I. Kim, G. Kang, S. Shin, D. Kang, M.-H. Baik, S. Hong, *Nat. Commun.* **2019**, *10*, 4117.

[2] Y. Okimoto, S. Sakaguchi, Y. Ishii, *J. Am. Chem. Soc.* **2002**, *124*, 1590.

[3] N. G. Commelly, W. E. Geiger, *Chem. Rev.* **1996**, 96, 877.

[4] M. A. Cismesia, T. P. Yoon, *Chem. Sci.* **2015**, *6*, 5426.

[5] H. J. Kuhn, S. E. Braslavsky, R. Schmidt, *Pure Appl. Chem.* **2004**, *76*, 2105.

[6] J. N. Damas, W. D. Bowman, E. F. Zalewski, R. A. Velapoldi, *J. Phy. Chem.* **1981**, *85*, 2766.

[7] P. S. Fier, *J. Am. Chem. Soc.* **2017**, *139*, 9499

[8] H. Huang, J. Cai, L. Tang, Z. Wang, F. Li, G-J. Deng, *J. Org. Chem.* **2016**, *81*, 1499.

[9] E. M. Simmons, J. F. Hartwig, *Angew. Chem. Int. Ed.* **2012**, *51*, 3066.

[10] R. G. Parr, W. Yang, Density Functional Theory of Atoms and Molecules, Oxford University Press, New York, **1989**.

[11] A. D. Bochevarov, E. Harder, T. F. Hughes, J. R. Greenwood, D. A. Braden, D. M. Philipp, D. Rinaldo, M. D. Halls, J. Zhang, R. A. Friesner, *Int. J. Quantum Chem.* **2013**, *113*, 2110.

[12] A. D. Becke, *J. Chem. Phys.* **1993**, *98*, 1372.

[13] B. Marten, K. Kim, C. Cortis, R. A. Friesner, R. B. Murphy, M. N. Ringnalda, D. Stikoff, B. Honig, *J. Phys. Chem.* **1996**, *100*, 11775.

[14] M. Friedrichs, R. Zhou, S. R. Edinger, R. A. Friesner, *J. Phys. Chem.* **1999**, *103*, 3057.

[15] S. R. Edinger, C. Cortis, P. S. Shenkin, R. A. Friesner, *J. Phys. Chem.* **1997**, *101*, 1190.

[16] Rashin, A. A. Honig, B. *J. Phys. Chem.* **1985**, *89*, 5588.

[17] F. Neese, *Wiley Interdiscip. Rev.: Comput. Mol. Sci.* **2002**, *2*, 73.

[18] Y. Zhao, D. G. Truhlar, *J. Phys. Chem. A* **2005**, *109*, 5656.

[19] Weigend, F. *Phys. Chem. Chem. Phys.* **2006**, *8*, 1057.

[20] Weigend, F. *Phys. Chem. Chem. Phys.* 2005, *7*, 3297.

[21] L. Huang, C. Zhu, L. Yi, H. Yue, R. Kancherla, M. Rueping, *Angew. Chem. Int. Ed.* **2020**, *59*, 457.

VII. Compound Characterizations

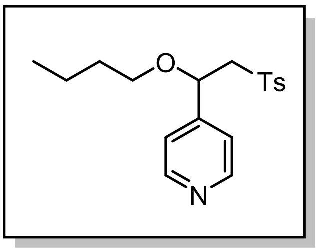

4-(1-butoxy-2-tosylethyl)pyridine (4a). Yield 89% (29.6 mg). White solid (mp: 94–97 °C). ^{1}H NMR (400 MHz, $CDCl_3$) δ 8.71 – 8.46 (m, 2H), 7.79 (d, *J* = 8.0 Hz, 2H), 7.34 (d, *J* = 8.0 Hz, 2H), 7.21 (d, *J* = 4.9 Hz, 2H), 4.82 (dd, *J* = 9.1, 2.8 Hz, 1H), 3.59 (dd, *J* = 14.7, 9.1 Hz, 1H), 3.29 – 3.08 (m, 3H), 2.45 (s, 3H), 1.41 – 1.20 (m, 2H), 1.24 – 1.06 (m, 2H), 0.80 (t, *J* = 7.3 Hz, 3H). ^{13}C NMR (100 MHz, $CDCl_3$) δ 150.3, 149.0, 144.8, 137.5, 129.8, 128.2, 121.4, 75.7, 69.8, 63.2, 31.6, 21.7, 19.2, 13.9. HRMS (EI) m/z calcd. for $C_{18}H_{23}NO_3S$ $[M]^+$: 333.1398, found : 333.1398.

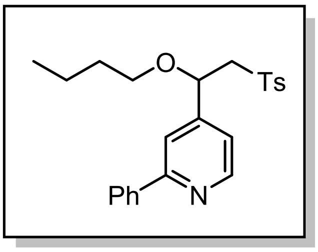

4-(1-butoxy-2-tosylethyl)-2-phenylpyridine (4b). Yield 95% (38.8 mg). White solid (mp: 44–47 °C). ^{1}H NMR (400 MHz, $CDCl_3$) δ 8.64 (d, *J* = 5.0 Hz, 1H), 7.99 – 7.91 (m, 2H), 7.80 (d, *J* = 8.1 Hz, 2H), 7.61 (s, 1H), 7.52 – 7.37 (m, 3H), 7.33 (d, *J* = 8.1 Hz, 2H), 7.14 (d, *J* = 5.0, 1.6 Hz, 1H), 4.89 (dd, *J* = 9.0, 3.0 Hz, 1H), 3.65 (dd, *J* = 14.8, 9.0 Hz, 1H), 3.30 (dd, *J* = 14.8, 3.0 Hz, 1H), 3.30 – 3.21 (m, 2H), 2.43 (s, 3H), 1.42 – 1.25 (m, 2H), 1.29 – 1.12 (m, 2H), 0.82 (t, *J* = 7.3 Hz, 3H). ^{13}C NMR (100 MHz, $CDCl_3$) δ 158.3, 150.3, 149.7, 144.8, 138.9, 137.5, 129.8, 129.4, 128.9, 128.2, 127.0, 119.8, 118.1, 76.0, 69.8, 63.3, 31.6, 21.7, 19.2, 14.0. HRMS (EI) m/z calcd. for $C_{24}H_{27}NO_3S$ $[M]^+$: 409.1711, found : 409.1710.

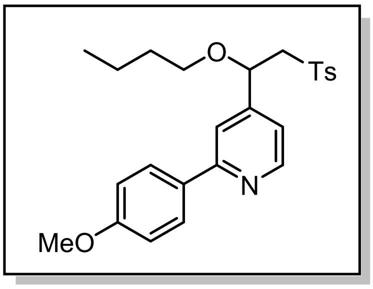

4-(1-butoxy-2-tosylethyl)-2-(4-methoxyphenyl)pyridine (4c). Yield 91% (40.0 mg). White solid (mp: 125–128 °C). ^{1}H NMR (600 MHz, $CDCl_3$) δ 8.58 (d, *J* = 5.0 Hz, 1H), 7.91 (d, *J* = 8.6 Hz, 2H), 7.79 (d, *J* = 8.0 Hz, 2H), 7.54 (s, 1H), 7.32 (d, *J* = 8.0 Hz, 2H), 7.06 (dd, *J* = 5.1, 1.5 Hz, 1H), 6.98 (d, *J* = 8.6 Hz, 2H), 4.86 (dd, *J* = 9.2, 2.8 Hz, 1H), 3.85 (s, 3H), 3.64 (dd, *J* = 14.8, 9.2 Hz, 1H), 3.29 (dd, *J* = 14.8, 2.8 Hz, 1H), 3.24 (t, *J* = 6.6 Hz, 2H), 2.42 (s, 3H), 1.37 – 1.26 (m, 2H), 1.25 – 1.12 (m, 2H), 0.81 (t, *J* = 7.4 Hz, 3H). ^{13}C NMR (150 MHz, $CDCl_3$) δ 160.8, 157.9, 150.2, 149.5, 144.7, 137.5, 131.5, 129.8, 128.3, 128.1, 119.0, 117.3, 114.3, 76.0, 69.7, 63.3, 55.5, 31.6, 21.7, 19.2. HRMS (EI) m/z calcd. for $C_{25}H_{29}NO_4S$ $[M]^+$: 439.1817, found : 439.1819.

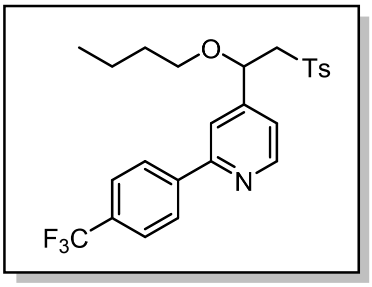

4-(1-butoxy-2-tosylethyl)-2-(4-(trifluoromethyl)phenyl)pyridine (4d). Yield 90% (43.0 mg). White solid (mp: 44–47 °C).. ^{1}H NMR (400 MHz, $CDCl_3$) δ 8.68 (dd, *J* = 5.0, 0.8 Hz, 1H), 8.08 (d, *J* = 7.8 Hz, 1H), 7.80 (d, *J* = 8.3 Hz, 2H), 7.76 – 7.69 (m, 1H), 7.69 – 7.63 (m, 1H), 7.34 (d, *J* = 7.7 Hz, 1H), 7.21 (dd, *J* = 5.1, 1.5 Hz, 1H), 4.92 (dd, *J* = 8.9, 3.0 Hz, 1H), 3.66 (dd, *J* = 14.7, 8.9 Hz, 1H), 3.35 – 3.21 (m, 3H), 2.43 (s, 3H), 1.43 – 1.25 (m, 2H), 1.29 – 1.12 (m, 2H), 0.82 (t, *J* = 7.3 Hz, 3H). ^{13}C NMR (100 MHz, $CDCl_3$) δ 156.7, 150.6, 150.1, 144.9, 142.3 (d, *J* = 1.6 Hz), 137.43, 131.2 (q, *J* = 32.4 Hz), 129.9, 128.2, 127.4, 125.9 (q, *J* = 3.8 Hz), 124.2 (q, *J* = 272.1 Hz), 120.7 , 118.4, 75.9, 69.9, 63.3, 31.6, 21.8, 19.2, 14.0. ^{19}F NMR (376 MHz, $CDCl_3$) δ -62.6. HRMS (EI) m/z calcd. for $C_{25}H_{26}F_3NO_3S$ $[M]^+$: 477.1586, found : 477.1588.

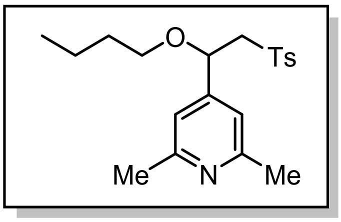

4-(1-butoxy-2-tosylethyl)-2,6-dimethylpyridine (4e). Yield 60% (21.6 mg). Colorless gum. ^{1}H NMR (600 MHz, $CDCl_3$) δ 7.78 (d, *J* = 8.0 Hz, 2H), 7.33 (d, *J* = 8.0 Hz, 2H), 6.86 (s, 2H), 4.74 (dd, *J* = 9.0, 2.8 Hz, 1H), 3.58 (dd, *J* = 14.8, 9.0 Hz, 1H), 3.23 – 3.19 (m, 1H), 3.18 (t, *J* = 6.6 Hz, 2H), 2.51 (s, 6H), 2.45 (s, 3H), 1.35 – 1.24 (m, 2H), 1.22 – 1.11 (m, 2H), 0.81 (t, *J* = 7.3 Hz, 3H). ^{13}C NMR (100 MHz, $CDCl_3$) δ 158.3, 144.7, 137.5, 129.8, 128.2, 118.2, 75.8, 69.7, 63.2, 31.6, 24.2, 21.8, 19.2, 13.9. HRMS (EI) m/z calcd. for $C_{20}H_{27}NO_3S$ $[M]^+$: 361.1712, found : 361.1713.

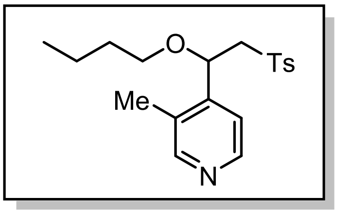

4-(1-butoxy-2-tosylethyl)-3-methylpyridine (4f). Yield 90% (31.2 mg). White solid (mp: 94–97 °C). ^{1}H NMR (600 MHz, $CDCl_3$) δ 8.41 (d, *J* = 5.0 Hz, 1H), 8.38 (s, 1H), 7.80 (d, *J* = 8.0 Hz, 2H), 7.34 (d, *J* = 8.0 Hz, 2H), 7.18 (d, *J* = 5.0 Hz, 1H), 5.00 (dd, *J* = 9.3, 2.1 Hz, 1H), 3.53 (dd, *J* = 14.9, 9.3 Hz, 1H), 3.16 – 3.10 (m, 3H), 2.45 (s, 3H), 2.31 (s, 3H), 1.34 – 1.17 (m, 2H), 1.20 – 1.08 (m, 2H), 0.79 (t, *J* = 7.3 Hz, 3H). ^{13}C NMR (100 MHz, $CDCl_3$) δ 151.7, 148.3, 146.6, 144.8, 137.4, 130.4, 129.8, 128.2, 120.1, 72.9, 69.6, 62.0, 31.6, 21.7, 19.2, 15.9, 14.0. HRMS (EI) m/z calcd. for $C_{19}H_{25}NO_3S$ $[M]^+$: 347.1556, found : 347.1555.

\

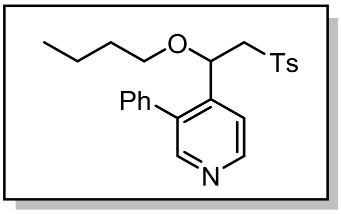

4-(1-butoxy-2-tosylethyl)-3-phenylpyridine (4g). Yield 82% (33.5 mg). White solid (mp: 127–130 °C). ^{1}H NMR (600 MHz, $CDCl_3$) δ 8.59 (d, *J* = 5.1 Hz, 1H), 8.46 (s, 1H), 7.60 (d, *J* = 8.1 Hz, 2H), 7.50 – 7.44 (m, 3H), 7.38 (d, *J* = 5.1 Hz, 1H), 7.26 (d, *J* = 8.1 Hz, 2H), 7.23 – 7.21 (m, 2H), 4.93 (dd, *J* = 9.9, 2.1 Hz, 1H), 3.46 (dd, *J* = 14.7, 9.9 Hz, 1H), 3.17 – 3.03 (m, 3H), 2.43 (s, 3H), 1.32 – 1.22 (m, 2H),

1.20 – 1.11 (m, 2H), 0.79 (t, *J* = 7.3 Hz, 3H). ^{13}C NMR (100 MHz, $CDCl_3$) δ 150.8, 149.5, 146.1, 144.5, 137.3, 136.6, 136.0, 129.7, 129.3, 129.0, 128.5, 128.1, 120.6, 72.4, 69.4, 62.1, 31.6, 21.7, 19.2, 14.0. HRMS (EI) m/z calcd. for $C_{24}H_{27}NO_3S$ $[M]^+$: 409.1712, found : 409.1714.

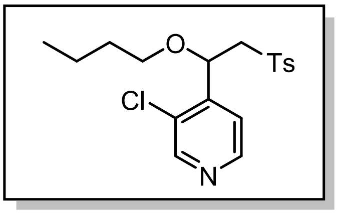

4-(1-butoxy-2-tosylethyl)-3-chloropyridine (4h). Yield 81% (29.7 mg). White solid (88–91 °C). ^{1}H NMR (600 MHz, CD_2Cl_2) δ 8.61 – 8.51 (m, 1H), 8.52 – 8.42 (m, 1H), 7.81 (d, *J* = 7.9 Hz, 2H), 7.39 (d, *J* = 7.9 Hz, 2H), 7.37 – 7.28 (m, 1H), 5.12 (dd, *J* = 9.8, 2.0 Hz, 1H), 3.46 (dd, *J* = 14.8, 9.8 Hz, 1H), 3.31 (dd, *J* = 14.8, 2.0 Hz, 1H), 3.28 – 3.16 (m, 2H), 2.46 (s, 3H), 1.38 – 1.26 (m, 2H), 1.25 – 1.15 (m, 2H), 0.83 (t, *J* = 7.3 Hz, 3H). ^{13}C NMR (100 MHz, $CDCl_3$) δ 149.9, 148.4, 146.2, 144.8, 137.6, 129.8, 128.2, 121.8, 77.2, 73.1, 70.4, 61.0, 31.6, 21.8, 19.2, 14.0. HRMS (EI) m/z calcd. for $C_{18}H_{22}ClNO_3S$ $[M]^+$: 367.1009, found : 367.1006.

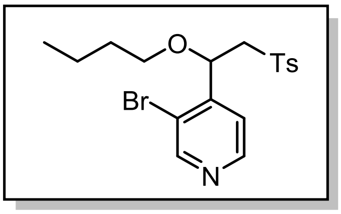

3-bromo-4-(1-butoxy-2-tosylethyl)pyridine (4i). Yield 80% (32.8 mg). White solid (88–91 °C). ^{1}H NMR (600 MHz, CD_2Cl_2) δ 8.72 – 8.59 (m, 1H), 8.56 – 8.42 (m, 1H), 7.82 (d, *J* = 7.9 Hz, 2H), 7.39 (d, *J* = 7.9 Hz, 2H), 7.33 (d, *J* = 4.8 Hz, 1H), 5.06 (d, *J* = 9.8 Hz, 1H), 3.43 (dd, *J* = 14.8, 9.8 Hz, 1H), 3.32 (dd, *J* = 14.8, 2.0 Hz, 1H), 3.28 – 3.15 (m, 2H), 2.46 (s, 3H), 1.39 – 1.26 (m, 2H), 1.26 – 1.13 (m, 2H), 0.83 (t, *J* = 7.3 Hz, 3H). ^{13}C NMR (100 MHz, $CDCl_3$) δ 152.4, 148.9, 147.9, 144.8, 137.6, 129.8, 128.2, 122.3, 75.3, 70.3, 61.0, 31.6, 21.8, 19.2, 14.0. HRMS (ESI) m/z calcd. for $C_{18}H_{23}BrNO_3S$ $[M+H]^+$: 412.0582, found : 412.0582.

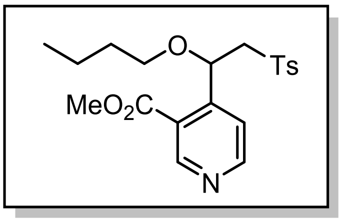

methyl 4-(1-butoxy-2-tosylethyl)nicotinate (4j). Yield 59% (23.2 mg). Colorless gum. ^1H NMR (400 MHz, $CDCl_3$) δ 9.13 (s, 1H), 8.73 (s, 1H), 7.84 (d, J = 8.3 Hz, 2H), 7.54 (d, J = 5.0 Hz, 1H), 7.35 (d, J = 8.3 Hz, 2H), 5.60 (dd, J = 9.5, 2.2 Hz, 1H), 3.99 (s, 3H), 3.52 (dd, J = 14.4, 2.3 Hz, 1H), 3.42 (dd, J = 14.4, 9.5 Hz, 1H), 3.31 – 3.20 (m, 1H), 3.20 – 3.09 (m, 1H), 2.45 (s, 3H), 1.40 – 1.24 (m, 2H), 1.28 – 1.12 (m, 2H), 0.82 (t, J = 7.3 Hz, 3H). ^{13}C NMR (151 MHz, $CDCl_3$) δ 165.9, 153.5, 152.1, 150.9, 144.5, 138.2, 129.7, 128.2, 124.4, 121.1, 73.5, 70.2, 62.4, 52.9, 31.6, 21.7, 19.2, 14.0. HRMS (EI) m/z calcd. for $C_{20}H_{25}NO_5S$ $[M]^+$: 391.1453, found : 391.1450.

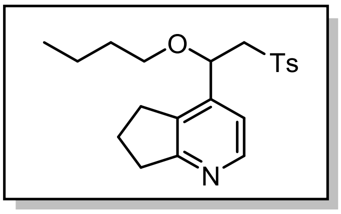

4-(1-butoxy-2-tosylethyl)-6,7-dihydro-5H-cyclopenta[b]pyridine (4k). Yield 81% (30.4 mg). White solid (mp: 105–108 °C). ^1H NMR (600 MHz, $CDCl_3$) δ 8.39 – 8.22 (m, 1H), 7.77 (d, J = 8.0 Hz, 2H), 7.33 (d, J = 8.0 Hz, 2H), 6.98 (d, J = 5.1 Hz, 1H), 4.85 (dd, J = 9.1, 2.7 Hz, 1H), 3.57 (dd, J = 14.8, 9.1 Hz, 1H), 3.19 – 3.12 (m, 3H), 3.01 (t, J = 7.8 Hz, 2H), 2.91 (t, J = 7.5 Hz, 2H), 2.44 (s, 3H), 2.18 – 2.07 (m, 2H), 1.34 – 1.20 (m, 2H), 1.20 – 1.07 (m, 2H), 0.79 (t, J = 7.3 Hz, 3H). ^{13}C NMR (100 MHz, $CDCl_3$) δ 166.4, 147.9, 144.7, 144.7, 137.4, 134.7, 129.8, 128.1, 118.1, 74.1, 69.7, 61.8, 34.0, 31.6, 29.0, 23.0, 21.7, 19.2, 14.0. HRMS (EI) m/z calcd. for $C_{21}H_{28}NO_3S$ $[M+H]^+$: 374.1790, found : 374.1793.

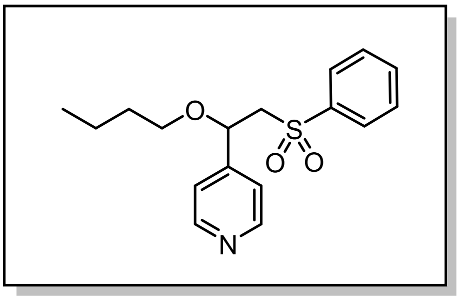

4-(1-butoxy-2-(phenylsulfonyl)ethyl)pyridine (4l). Yield 75% (24.0 mg). Yellow solid (74–77 ^{o}C). ^{1}H NMR (600 MHz, CD_2Cl_2) δ 8.64 – 8.50 (m, 2H), 7.91 (d, *J* = 7.8 Hz, 2H), 7.68 (t, *J* = 7.5 Hz, 1H), 7.58 (t, *J* = 7.7 Hz, 2H), 7.27 (d, *J* = 4.9 Hz, 2H), 4.83 (dd, *J* = 9.2, 2.8 Hz, 1H), 3.63 (dd, *J* = 14.9, 9.2 Hz, 1H), 3.27 (dd, *J* = 14.8, 2.8 Hz, 1H), 3.24 – 3.13 (m, 2H), 1.33 – 1.22 (m, 2H), 1.25 – 1.07 (m, 2H), 0.79 (t, *J* = 7.3 Hz, 3H). ^{13}C NMR (100 MHz, CD_2Cl_2) δ = 150.0, 149.8, 140.8, 134.0, 129.5, 128.3, 122.1, 76.0, 70.0, 63.2, 31.8, 19.4, 13.9. HRMS (EI) m/z calcd. for $C_{17}H_{22}NO_3S$ $[M+H]^+$: 320.1320, found : 320.1321.

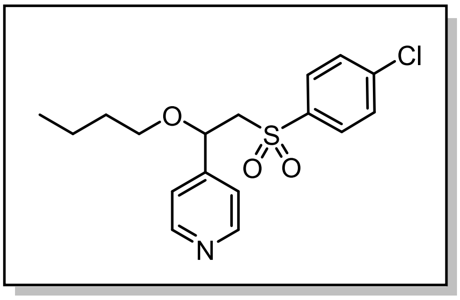

4-(1-butoxy-2-((4-chlorophenyl)sulfonyl)ethyl)pyridine (4m). Yield 85% (29.9 mg). White solid (mp: 104–107 ^{o}C). ^{1}H NMR (400 MHz, $CDCl_3$) δ 8.98 – 8.31 (m, 2H), 7.94 – 7.79 (m, 2H), 7.58 – 7.44 (m, 2H), 7.32 – 7.12 (m, 2H), 4.84 (dd, *J* = 9.5, 2.6 Hz, 1H), 3.59 (dd, *J* = 14.8, 9.5 Hz, 1H), 3.28 – 3.22 (m, 1H), 3.22 – 3.13 (m, 2H), 1.36 – 1.21 (m, 2H), 1.20 – 1.04 (m, 2H), 0.80 (t, *J* = 7.3 Hz, 3H). ^{13}C NMR (100 MHz, $CDCl_3$) δ 150.0, 149.1, 140.6, 139.0, 129.7, 129.5, 121.7, 75.7, 69.8, 63.1, 31.5, 19.2, 13.9. HRMS (EI) m/z calcd. for $C_{17}H_{21}ClNO_3S$ $[M+H]^+$: 354.0931, found : 354.0932.

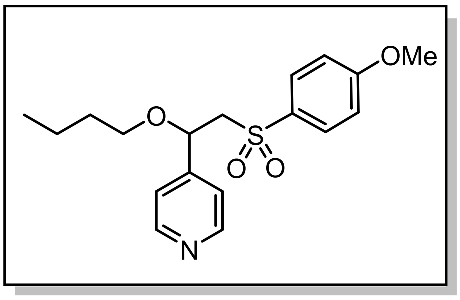

4-(1-butoxy-2-((4-methoxyphenyl)sulfonyl)ethyl)pyridine (4n). Yield 90% (31.3 mg). White solid (mp: 95–98 ^{o}C). ^{1}H NMR (600 MHz, $CDCl_3$) δ 8.58 (d, J = 5.1 Hz, 2H), 7.84 (d, J = 8.8 Hz, 2H), 7.20 (d, J = 5.1 Hz, 2H), 7.00 (d, J = 8.8 Hz, 2H), 4.83 (dd, J = 9.1, 2.8 Hz, 1H), 3.89 (s, 3H), 3.58 (dd, J = 14.8, 9.1 Hz, 1H), 3.28 – 3.15 (m, 3H), 1.40 – 1.28 (m, 2H), 1.19 (dddd, J = 22.5, 15.6, 13.7, 6.7 Hz, 2H), 0.82 (t, J = 7.4 Hz, 3H). ^{13}C NMR (100 MHz, $CDCl_3$) δ = 163.9, 150.5, 148.9, 132.0, 130.4, 121.4,

114.4, 75.8, 69.8, 63.5, 55.9, 31.7, 19.2, 14.0. HRMS (ESI) m/z calcd. for $C_{18}H_{24}NO_4S$ [M+H]+ : 350.1426, found : 350.1426.

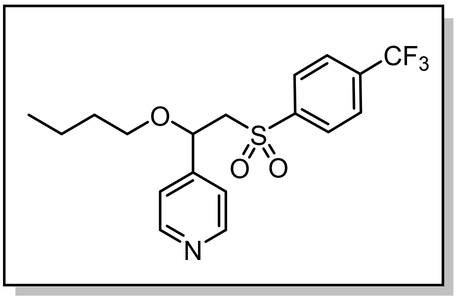

4-(1-butoxy-2-((4-(trifluoromethyl)phenyl)sulfonyl)ethyl)pyridine (4o). Yield 58% (22.5 mg). Yellow solid (mp: 114–117 °C). ^{1}H NMR (600 MHz, $CDCl_3$) δ 8.68 – 8.56 (m, 2H), 8.08 (d, *J* = 8.1 Hz, 2H), 7.84 (d, *J* = 8.2 Hz, 2H), 7.23 (d, *J* = 4.9 Hz, 2H), 4.87 (dd, *J* = 9.7, 2.4 Hz, 1H), 3.64 (dd, *J* = 15.0, 9.7 Hz, 1H), 3.27 (dd, *J* = 15.0, 2.5 Hz, 1H), 3.17 (qd, *J* = 9.0, 4.7 Hz, 2H), 1.21 (dddd, *J* = 22.3, 15.7, 12.5, 5.2 Hz, 2H), 1.16 – 1.02 (m, 2H), 0.77 (t, *J* = 7.3 Hz, 3H). ^{13}C NMR (100 MHz, $CDCl_3$) δ 150.3, 148.7, 144.1, 135.5 (q, *J* = 33.1 Hz), 128.8, 126.3 (q, *J* = 3.7 Hz), 123.3 (q, *J* = 273.1 Hz), 121.5, 75.8, 69.8, 63.0, 31.5, 19.2, 13.8. ^{19}F NMR (376 MHz, $CDCl_3$) δ -63.2. HRMS (EI) m/z calcd. for $C_{18}H_{21}F_3NO_3S$ $[M+H]^+$: 388.1194, found : 388.1195.

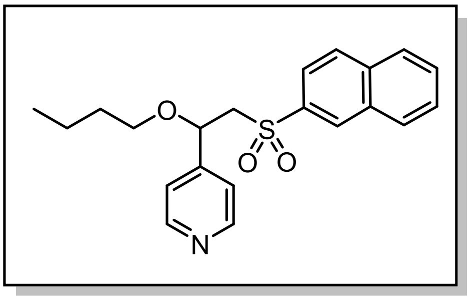

4-(1-butoxy-2-(naphthalen-2-ylsulfonyl)ethyl)pyridine (4p). Yield 90% (33.1 mg). Colorless gum. ^{1}H NMR (400 MHz, CD_2Cl_2) δ 8.56 – 8.52 (m, 2H), 8.50 (dd, *J* = 2.0, 0.8 Hz, 1H), 8.07 – 8.00 (m, 2H), 8.01 – 7.94 (m, 1H), 7.88 (dd, *J* = 8.7, 1.9 Hz, 1H), 7.68 (dddd, *J* = 18.3, 8.1, 6.9, 1.4 Hz, 2H), 7.23 – 7.16 (m, 2H), 4.84 (dd, *J* = 9.4, 2.7 Hz, 1H), 3.70 (dd, *J* = 14.8, 9.4 Hz, 1H), 3.32 (dd, *J* = 14.8, 2.7 Hz, 1H), 3.21 – 3.07 (m, 2H), 1.21 – 1.02 (m, 2H), 1.05 – 0.90 (m, 2H), 0.62 (t, *J* = 7.2 Hz, 3H). ^{13}C NMR (100 MHz, CD_2Cl_2) δ 150.7, 148.8, 137.8, 135.7, 132.5, 130.1, 129.7, 129.6, 128.3, 128.1, 123.2, 121.7, 76.2, 69.8, 63.3, 31.7, 19.3, 13.8. HRMS (EI) m/z calcd. for $C_{21}H_{24}NO_3S$ $[M+H]^+$: 370.1477, found : 370.1474.

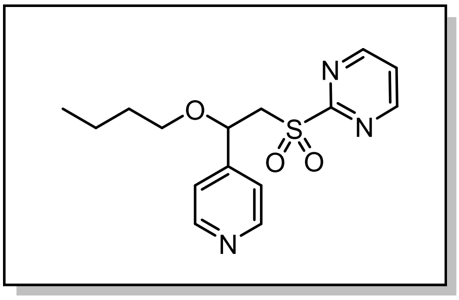

2-((2-butoxy-2-(pyridin-4-yl)ethyl)sulfonyl)pyrimidine (4q). Yield 70% (22.5 mg). Yellow gum. ^{1}H NMR (600 MHz, CD_2Cl_2) δ 8.94 (d, *J* = 4.9 Hz, 2H), 8.56 (d, *J* = 5.0 Hz, 2H), 7.56 (t, *J* = 4.9 Hz, 1H), 7.24 (d, *J* = 5.0 Hz, 2H), 4.86 (dd, *J* = 10.4, 3.0 Hz, 1H), 4.27 (dd, *J* = 15.0, 10.4 Hz, 1H), 3.43 (dd, *J* = 15.0, 3.0 Hz, 1H), 3.21 – 3.07 (m, 2H), 1.12 – 0.92 (m, 4H), 0.72 (t, *J* = 7.0 Hz, 3H). ^{13}C NMR (100 MHz, CD_2Cl_2) δ 166.5, 158.8, 150.7, 148.2, 123.9, 121.8, 76.3, 69.7, 57.8, 31.7, 19.3, 13.9. HRMS (EI) m/z calcd. for $C_{15}H_{20}N_3O_3S$ $[M+H]^+$: 322.1225, found : 322.1227.

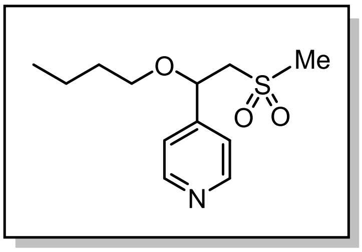

4-(1-butoxy-2-(methylsulfonyl)ethyl)pyridine (4r). Yield 78% (20.0 mg). White solid (53-56 °C). ^{1}H NMR (600 MHz, $CDCl_3$) δ 8.64 (d, *J* = 5.6 Hz, 2H), 7.26 (d, *J* = 5.6 Hz, 2H), 4.86 (dd, *J* = 10.7, 2.1 Hz, 1H), 3.45 – 3.37 (m, 2H), 3.35 (dt, *J* = 9.2, 6.8 Hz, 1H), 3.08 (s, 3H), 3.00 (d, *J* = 15.1 Hz, 1H), 1.61 – 1.53 (m, 2H), 1.42 – 1.28 (m, 2H), 0.89 (t, *J* = 7.4 Hz, 3H). ^{13}C NMR (100 MHz, $CDCl_3$) δ 150.7, 148.1, 121.3, 76.2, 69.9, 61.6, 43.6, 31.8, 19.4, 13.9. HRMS (EI) m/z calcd. for $C_{12}H_{20}NO_3S$ $[M+H]^+$: 258.1164, found : 258.1162.

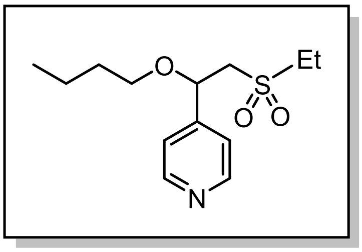

4-(1-butoxy-2-(ethylsulfonyl)ethyl)pyridine (4s). Yield 67% (18.0 mg). White solid (mp: 80–83 °C). ^{1}H NMR (600 MHz, CD_2Cl_2) δ 8.60 (d, *J* = 5.1 Hz, 2H), 7.39 – 7.21 (m, 2H), 4.83 (dd, *J* = 10.4, 2.2 Hz, 1H), 3.46 – 3.41 (m, 1H), 3.40 – 3.31 (m, 2H), 3.26 – 3.18 (m, 1H), 3.18 – 3.08 (m, 1H), 2.94 – 2.88 (m, 1H), 1.59 – 1.53 (m, 2H), 1.41 – 1.30 (m, 4H), 0.89 (t, *J* = 7.4 Hz, 3H). ^{13}C NMR (100 MHz, CD_2Cl_2) δ 150.8, 148.6, 121.7, 76.4, 70.0, 59.1, 50.0, 32.1, 19.7, 14.0, 6.8. HRMS (EI) m/z calcd. for $C_{13}H_{22}NO_3S$ $[M+H]^+$: 272.1320, found : 272.1319.

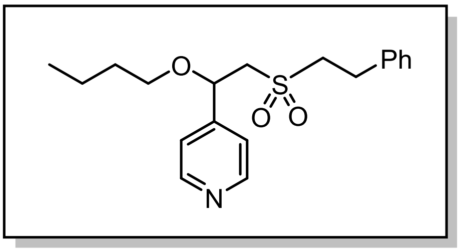

4-(1-butoxy-2-(phenethylsulfonyl)ethyl)pyridine (4t). Yield 68% (23.5 mg). White solid (mp: 90–93 °C).. ^{1}H NMR (400 MHz, CD_2Cl_2) δ 8.78 – 8.45 (m, 2H), 7.42 – 7.31 (m, 2H), 7.30 – 7.11 (m, 5H), 4.84 (dd, *J* = 10.5, 2.3 Hz, 1H), 3.56 (ddd, *J* = 13.8, 9.6, 7.3 Hz, 1H), 3.47 – 3.29 (m, 4H), 3.16 (ddd, *J* = 9.7, 6.4, 2.8 Hz, 2H), 2.92 (ddd, *J* = 15.2, 2.3, 1.4 Hz, 1H), 1.63 – 1.45 (m, 2H), 1.45 – 1.22 (m, 2H), 0.86 (t, *J* = 7.4 Hz, 3H). ^{13}C NMR (100 MHz, CD_2Cl_2) δ 150.8, 148.4, 138.4, 129.2, 128.9, 127.3, 121.7, 76.5, 70.0, 60.0, 56.9, 32.1, 28.6, 19.8, 13.9. HRMS (EI) m/z calcd. for $C_{19}H_{25}NO_3S$ $[M]^+$: 347.1555, found : 347.1558.

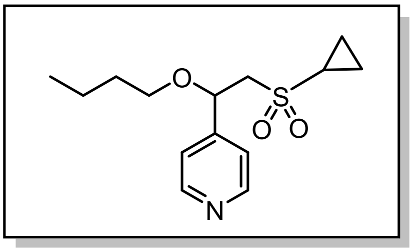

4-(1-butoxy-2-(cyclopropylsulfonyl)ethyl)pyridine (4u). Yield 73% (20.7 mg). White solid (mp: 69–72 °C). ^{1}H NMR (400 MHz, $CDCl_3$) δ 8.63 (d, *J* = 5.0 Hz, 2H), 7.31 – 7.26 (m, 2H), 4.90 (dd, *J* = 10.1, 2.5 Hz, 1H), 3.52 – 3.39 (m, 2H), 3.35 (dt, *J* = 9.1, 6.8 Hz, 1H), 3.06 (dd, *J* = 14.9, 2.5z Hz, 1H), 2.64 (tt, *J* = 8.0, 4.9 Hz, 1H), 1.63 – 1.51 (m, 2H), 1.47 – 1.27 (m, 3H), 1.19 – 1.09 (m, 1H), 1.12 – 0.99 (m, 2H), 0.88 (t, *J* = 7.4 Hz, 3H). ^{13}C NMR (100 MHz, $CDCl_3$) δ 150.6, 148.6, 121.4, 76.2, 69.9, 60.9, 31.9, 31.9, 19.4, 14.0, 5.8, 4.8. HRMS (EI) m/z calcd. for $C_{14}H_{22}NO_3S$ $[M+H]^+$: 284.1320, found : 284.1318.

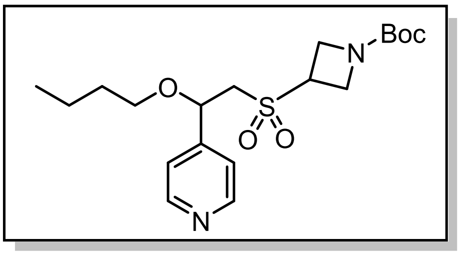

***tert*-butyl 3-((2-butoxy-2-(pyridin-4-yl)ethyl)sulfonyl)azetidine-1-carboxylate (4v).** Yield 58% (23.1 mg). White solid (mp: 95–98 °C). ^{1}H NMR (600 MHz, CD_2Cl_2) δ 8.61 (d, *J* = 5.5 Hz, 2H), 7.26 (d, *J* = 5.5 Hz, 2H), 4.81 (dd, *J* = 10.5, 2.1 Hz, 1H), 4.36 – 4.28 (m, 1H), 4.22 – 4.13 (m, 4H), 3.42 – 3.25 (m, 3H), 3.01 (dd, *J* = 15.2, 2.2 Hz, 1H), 1.63 – 1.52 (m, 2H), 1.43 (s, 9H), 1.38 – 1.22 (m, 2H), 0.89 (t, *J* = 7.4 Hz, 3H). ^{13}C NMR (100 MHz, CD_2Cl_2) δ 156.1, 150.9, 148.0, 121.7, 80.5, 76.1, 70.0, 59.1, 50.8, 32.0, 28.4, 19.6, 14.0. HRMS (EI) m/z calcd. for $C_{19}H_{30}$N2O_5S $[M]^+$: 398.1875, found : 398.1873.

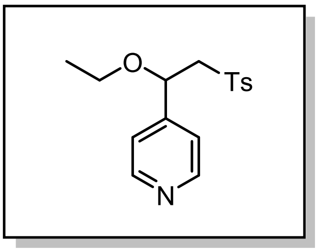

4-(1-ethoxy-2-tosylethyl)pyridine (4w). Yield 78% (23.9 mg). White solid (mp: 105–108 °C). ^{1}H NMR (400 MHz, $CDCl_3$) δ 8.90 – 8.28 (m, 2H), 7.79 (d, *J* = 8.4 Hz, 2H), 7.34 (d, *J* = 7.7 Hz, 2H), 7.26 – 7.17 (m, 2H), 4.85 (dd, *J* = 9.3, 2.8 Hz, 1H), 3.58 (dd, *J* = 14.7, 9.3 Hz, 1H), 3.34 – 3.22 (m, 2H), 3.22 (dd, *J* = 14.7, 2.8 Hz, 1H), 2.44 (s, 3H), 0.96 (t, *J* = 7.0 Hz, 3H). ^{13}C NMR (100 MHz, $CDCl_3$) δ 150.2, 149.0, 144.8, 137.4, 129.8, 128.2, 121.5, 75.6, 65.3, 63.2, 21.8, 14.9. HRMS (EI) m/z calcd. for $C_{16}H_{19}NO_3S$ $[M]^+$: 305.1086, found : 305.1088.

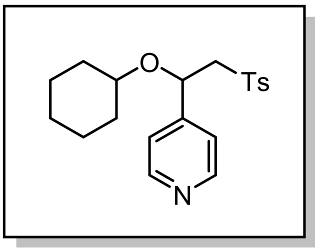

4-(1-(cyclohexyloxy)-2-tosylethyl)pyridine (4x). Yield 89% (31.9 mg). Pale-beige solid (mp: 100–103 °C). ^{1}H NMR (400 MHz, $CDCl_3$) δ 8.67 – 8.43 (m, 2H), 7.78 (d, *J* = 8.3 Hz, 2H), 7.32 (d, *J* = 8.0 Hz, 2H), 7.24 (d, *J* = 4.9 Hz, 2H), 5.07 (dd, *J* = 8.7, 3.1 Hz, 1H), 3.58 (dd, *J* = 14.6, 8.7 Hz, 1H), 3.19 (dd, *J* = 14.6, 3.1 Hz, 1H), 3.17 – 3.09 (m, 1H), 2.43 (s, 3H), 1.93 – 1.79 (m, 1H), 1.77 – 1.64 (m, 1H), 1.64 – 1.51 (m, 2H), 1.50 – 1.41 (m, 1H), 1.31 – 0.98 (m, 5H). ^{13}C NMR (100 MHz, $CDCl_3$) δ 150.3, 150.1, 144.8, 137.5, 129.8, 128.1, 121.5, 72.4, 63.5, 33.2, 31.4, 25.6, 24.2, 24.1, 21.7. HRMS (EI) m/z calcd. for $C_{20}H_{25}NO_3S$ $[M]^+$: 359.1555, found : 359.1555.

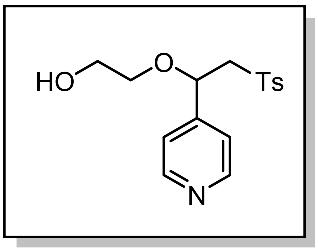

2-(1-(pyridin-4-yl)-2-tosylethoxy)ethan-1-ol (4y). Yield 83% (26.8 mg). White solid (mp: 78–81 °C). ^{1}H NMR (400 MHz, $CDCl_3$) δ 8.68 – 8.52 (m, 2H), 7.82 (d, *J* = 8.3 Hz, 2H), 7.37 (d, *J* = 8.0 Hz, 2H), 7.28 (d, *J* = 5.0 Hz, 2H), 5.01 (dd, *J* = 9.8, 2.3 Hz, 1H), 3.74 – 3.51 (m, 4H), 3.43 – 3.36 (m, 1H), 3.28 (brs, 1H), 3.22 (dd, *J* = 14.5, 2.3 Hz, 1H), 2.45 (s, 3H). ^{13}C NMR (100 MHz, $CDCl_3$) δ 149.9, 149.0, 145.3, 136.9, 130.1, 128.0, 121.5, 75.6, 71.5, 62.9, 61.3, 21.8. HRMS (EI) m/z calcd. for $C_{16}H_{19}NO_4S$ $[M]^+$: 321.1035, found : 321.1034.

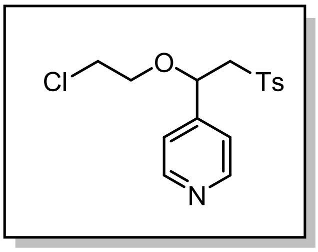

4-(1-(2-chloroethoxy)-2-tosylethyl)pyridine (4z). Yield 67% (22.6 mg). White solid (mp: 110–113 °C). ^{1}H NMR (400 MHz, $CDCl_3$) δ 8.59 (d, *J* = 4.6 Hz, 2H), 7.84 – 7.77 (m, 2H), 7.40 – 7.31 (m, 2H), 7.26 – 7.20 (m, 2H), 4.93 (dd, *J* = 9.0, 2.9 Hz, 1H), 3.63 (dd, *J* = 14.8, 9.0 Hz, 1H), 3.56 (dt, *J* = 10.2, 5.3 Hz, 1H), 3.48 (ddd, *J* = 10.2, 7.2, 4.9 Hz, 1H), 3.45 – 3.29 (m, 2H), 3.25 (dd, *J* = 14.8, 2.9 Hz, 1H), 2.45 (s, 3H). ^{13}C NMR (100 MHz, $CDCl_3$) δ 150.6, 147.8, 145.0, 137.3, 129.9, 128.1, 121.3, 76.0, 69.8, 63.1, 42.2, 21.8. HRMS (EI) m/z calcd. for $C_{16}H_{18}ClNO_3S$ $[M]^+$: 339.0696, found : 339.0698.

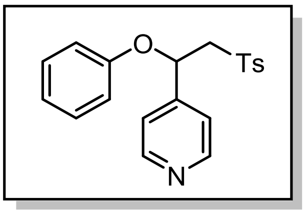

4-(1-phenoxy-2-tosylethyl)pyridine (4aa). Yield 59% (20.9 mg). White solid (mp: 130–133 °C). ^{1}H NMR (600 MHz, $CDCl_3$) δ 8.69 – 8.46 (m, 2H), 7.74 (d, *J* = 8.2 Hz, 2H), 7.28 (d, *J* = 8.2 Hz, 2H), 7.28 – 7.25 (m, 2H), 7.18 – 7.12 (m, 2H), 6.92 (t, *J* = 7.3 Hz, 1H), 6.53 (d, *J* = 8.1 Hz, 2H), 5.73 (dd, *J* = 9.4, 2.5 Hz, 1H), 3.83 (dd, *J* = 15.0, 9.4 Hz, 1H), 3.44 (dd, *J* = 15.0, 2.5 Hz, 1H), 2.44 (s, 3H). ^{13}C NMR (100 MHz, $CDCl_3$) δ 156.2, 150.5, 147.9, 145.0, 137.0, 129.9, 129.6, 128.4, 122.1, 120.9, 115.6, 73.8, 63.2, 21.8. HRMS (EI) m/z calcd. for $C_{20}H_{19}NO_3S$ $[M]^+$: 353.1086, found : 353.1089.

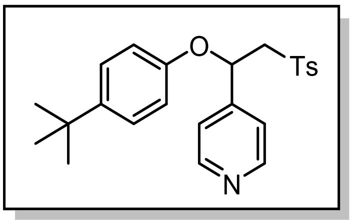

4-(1-(4-(tert-butyl)phenoxy)-2-tosylethyl)pyridine (4ab). Yield 67% (27.3 mg). White solid (mp: 173–176 °C). ^{1}H NMR (600 MHz, CD_2Cl_2) δ 8.55 (d, *J* = 5.0 Hz, 2H), 7.73 (d, *J* = 7.8 Hz, 2H), 7.32 (d, *J* = 7.9 Hz, 2H), 7.26 (d, *J* = 5.0 Hz, 2H), 7.18 (d, *J* = 8.2 Hz, 2H), 6.51 (d, *J* = 8.3 Hz, 2H), 5.68 (dd, *J* = 9.5, 2.8 Hz, 1H), 3.82 (dd, *J* = 15.0, 9.5 Hz, 1H), 3.44 (dd, *J* = 15.0, 2.8 Hz, 1H), 2.45 (s, 3H), 1.25 (s, 9H). ^{13}C NMR (100 MHz, CD_2Cl_2) δ 154.4, 150.9, 148.0, 145.4, 145.1, 137.6, 130.1, 128.6, 126.6, 121.2, 115.3, 74.2, 63.4, 34.4, 31.5, 21.8. HRMS (ESI) m/z calcd. for $C_{24}H_{28}NO_3S$ $[M+H]^+$: 410.1788, found : 410.1790.

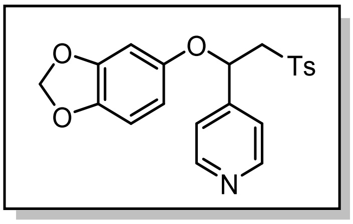

4-(1-(benzo[d][1,3]dioxol-5-yloxy)-2-tosylethyl)pyridine (4ac). Yield 70% (27.7 mg). Pale yellow solid (mp: 54–57 °C). ^{1}H NMR (400 MHz, CD_2Cl_2) δ 8.57 – 8.51 (m, 2H), 7.78 – 7.69 (m, 2H), 7.34

(d, J = 8.1 Hz, 2H), 7.26 – 7.20 (m, 2H), 6.56 (d, J = 8.5 Hz, 1H), 6.16 (d, J = 2.5 Hz, 1H), 6.04 (dd, J = 8.5, 2.5 Hz, 1H), 5.90 – 5.80 (m, 2H), 5.57 (dd, J = 9.4, 2.6 Hz, 1H), 3.81 (dd, J = 14.9, 9.4 Hz, 1H), 3.41 (dd, J = 14.9, 2.6 Hz, 1H), 2.45 (s, 3H). ^{13}C NMR (100 MHz, CD_2Cl_2) δ 152.0, 150.9, 148.6, 147.7, 145.5, 143.0, 137.5, 130.2, 128.5, 121.3, 108.1, 107.9, 101.9, 99.5, 75.4, 63.2, 21.8. HRMS (ESI) m/z calcd. for $C_{21}H_{20}NO_5S$ $[M+H]^+$: 398.1060, found : 398.1062.

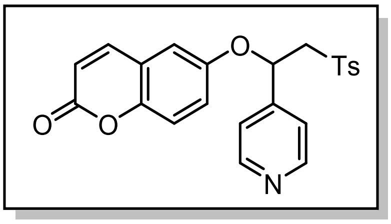

6-(1-(pyridin-4-yl)-2-tosylethoxy)-2H-chromen-2-one (4ad). Yield 40% (25.1 mg). Pale yellow solid (mp: 175–178 °C). ^{1}H NMR (600 MHz, CD_2Cl_2) δ 8.57 (d, J = 5.0 Hz, 2H), 7.73 (d, J = 7.9 Hz, 2H), 7.53 (d, J = 9.6 Hz, 1H), 7.32 (d, J = 7.9 Hz, 2H), 7.27 (d, J = 5.1 Hz, 2H), 7.15 (d, J = 9.0 Hz, 1H), 6.76 (dd, J = 9.0, 2.9 Hz, 1H), 6.67 (d, J = 3.0 Hz, 1H), 6.35 (d, J = 9.6 Hz, 1H), 5.71 (dd, J = 9.4, 2.4 Hz, 1H), 3.87 (dd, J = 15.0, 9.5 Hz, 1H), 3.46 (dd, J = 15.1, 2.4 Hz, 1H), 2.44 (s, 3H). ^{13}C NMR (100 MHz, CD_2Cl_2) δ 160.7, 153.0, 151.1, 149.6, 147.1, 145.6, 143.1, 137.4, 130.3, 128.5, 121.1, 120.9, 119.6, 118.2, 117.7, 113.1, 75.2, 63.2, 21.8. HRMS (ESI) m/z calcd. for $C_{23}H_{20}NO_5S$ $[M+H]^+$: 422.1062, found : 422.1063.

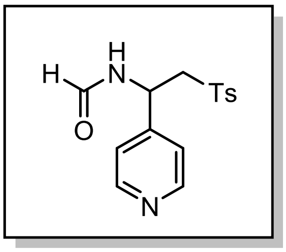

***N*-(1-(pyridin-4-yl)-2-tosylethyl)formamide (4ae).** Yield 65% (19.8 mg). Colorless gum. ^{1}H NMR (600 MHz, $CDCl_3$) δ 8.57 – 8.43 (m, 2H), 8.25 (s, 1H), 7.68 (d, J = 7.8 Hz, 2H), 7.37 – 7.29 (m, 3H), 7.13 (d, J = 4.8 Hz, 2H), 5.43 – 5.30 (m, 1H), 3.72 (dd, J = 14.8, 9.0 Hz, 1H), 3.46 (dd, J = 14.8, 3.8 Hz, 1H), 2.44 (s, 3H). ^{13}C NMR (100 MHz, $CDCl_3$) δ 161.0, 149.4, 148.7, 145.9, 135.6, 130.3, 128.1, 122.0, 59.4, 47.7, 21.8. HRMS (ESI) m/z calcd. for $C_{15}H_{17}N_2O_3S$ $[M+H]^+$: 305.0960, found : 305.0960.

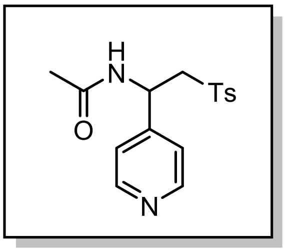

N-(1-(pyridin-4-yl)-2-tosylethyl)acetamide (4af). Yield 61% (19.4 mg). Colorless gum. ^{1}H NMR (400 MHz, $CDCl_3$) δ 8.62 – 8.36 (m, 2H), 7.71 – 7.63 (m, 2H), 7.32 (d, J = 8.1 Hz, 2H), 7.11 (d, J = 5.0 Hz, 2H), 6.89 (d, J = 6.9 Hz, 2H), 5.30 (ddd, J = 8.8, 6.9, 3.9 Hz, 1H), 3.67 (dd, J = 14.8, 8.8 Hz, 1H), 3.46 (dd, J = 14.8, 3.9 Hz, 1H), 2.44 (s, 3H), 2.05 (s, 3H). ^{13}C NMR (100 MHz, $CDCl_3$) δ 170.0, 150.3, 148.0, 145.7, 135.8, 130.3, 128.1, 121.4, 59.5, 48.9, 23.3, 21.8. HRMS (ESI) m/z calcd. for $C_{16}H_{19}N_2O_3S$ $[M+H]^+$: 319.1115, found : 319.1116.

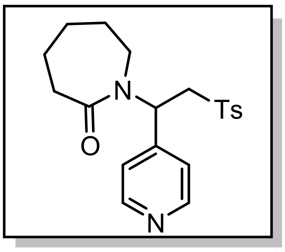

1-(1-(pyridin-4-yl)-2-tosylethyl)azepan-2-one (4ag). Yield 51% (18.9 mg). Pale-baige solid (mp: 140–143 °C). ^{1}H NMR (400 MHz, $CDCl_3$) δ 8.71 – 8.37 (m, 2H), 7.88 – 7.74 (m, 2H), 7.39 – 7.31 (m, 2H), 7.15 (d, J = 5.8 Hz, 2H), 5.88 (dd, J = 9.6, 3.9 Hz, 1H), 3.99 (dd, J = 14.7, 9.6 Hz, 1H), 3.60 (dd, J = 14.7, 4.0 Hz, 1H), 3.22 (dd, J = 6.0, 3.8 Hz, 2H), 2.50 – 2.44 (m, 2H), 2.44 (s, 3H), 1.74 – 1.52 (m, 5H), 1.40 – 1.27 (m, 1H). ^{13}C NMR (100 MHz, $CDCl_3$) δ 176.4, 150.3, 147.2, 145.3, 136.0, 130.1, 128.5, 122.3, 55.9, 53.6, 47.8, 37.7, 29.9, 28.5, 22.8, 21.8. HRMS (ESI) m/z calcd. for $C_{20}H_{25}N_2O_3S$ $[M+H]^+$: 373.1584, found : 373.1586.

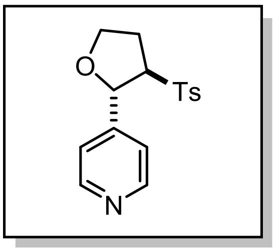

4-((2S,3R)-3-tosyltetrahydrofuran-2-yl)pyridine (4ah). Yield 86% (26.1 mg). White solid (mp: 126–129 °C). The stereochemistry was assigned to be trans as confirmed by ^{1}H NMR experiments by

comparison with literature precedent[1]. ^1H NMR (400 MHz, $CDCl_3$) δ 8.61 – 8.45 (m, 2H), 7.83 – 7.75 (m, 2H), 7.37 (d, *J* = 7.9 Hz, 2H), 7.20 (d, *J* = 4.9 Hz, 2H), 5.38 (d, *J* = 5.1 Hz, 1H), 4.17 – 4.06 (m, 1H), 4.02 (td, *J* = 8.6, 6.8 Hz, 1H), 3.59 (dd, *J* = 9.3, 5.1 Hz, 1H), 2.45 (s, 3H), 2.44 – 2.39 (m, 1H), 2.26 – 2.10 (m, 1H). ^{13}C NMR (100 MHz, $CDCl_3$) δ 150.1, 149.8, 145.7, 135.0, 130.3, 128.8, 120.7, 78.5, 71.3, 68.8, 29.0, 21.8. HRMS (EI) m/z calcd. for $C_{16}H_{17}NO_3S$ $[M]^+$: 303.0929, found : 303.0931.

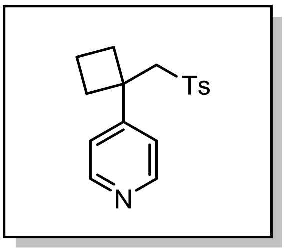

4-(1-(tosylmethyl)cyclobutyl)pyridine (4ai). Yield 60% (18.2 mg). White solid (mp: 80–83 °C). ^1H NMR (400 MHz, $CDCl_3$) δ 9.01 – 7.84 (m, 2H), 7.46 – 7.35 (m, 2H), 7.11 (d, *J* = 8.0 Hz, 2H), 7.09 – 6.97 (m, 2H), 3.71 (s, 2H), 2.60 – 2.49 (m, 2H), 2.44 (dtd, *J* = 10.9, 7.2, 2.2 Hz, 2H), 2.36 (s, 3H), 2.23 – 2.04 (m, 1H), 2.00 – 1.77 (m, 1H). ^{13}C NMR (100 MHz, $CDCl_3$) δ 154.5, 149.3, 144.5, 137.6, 129.7, 127.6, 122.1, 65.9, 44.0, 33.3, 21.7, 16.4. HRMS (ESI) m/z calcd. for $C_{17}H_{20}NO_2S$ $[M+H]^+$: 302.1214, found : 302.1215.

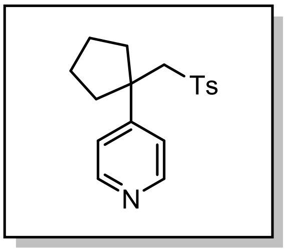

4-(1-(tosylmethyl)cyclopentyl)pyridine (4aj). Yield 40% (12.7 mg) White solid (mp: 110–113 °C). ^1H NMR (500 MHz, $CDCl_3$) δ 8.91 – 7.98 (m, 2H), 7.40 (d, *J* = 8.0 Hz, 2H), 7.23 – 7.05 (m, 4H), 3.57 (s, 2H), 2.38 (s, 3H), 2.17 – 2.05 (m, 4H), 1.85 – 1.73 (m, 2H), 1.69 – 1.56 (m, 2H). ^{13}C NMR (125 MHz, $CDCl_3$) δ 154.1, 148.6, 144.6, 137.6, 129.8, 127.6, 123.0, 65.9, 49.6, 38.1, 22.6, 21.7. HRMS (ESI) m/z calcd. for $C_{18}H_{22}NO_2S$ $[M+H]^+$: 316.1371, found : 316.1372.

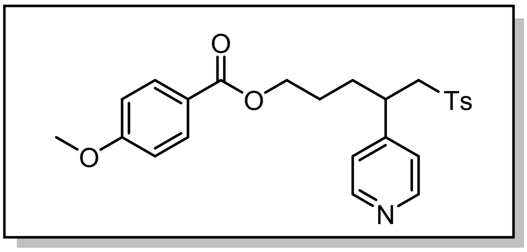

4-(pyridin-4-yl)-5-tosylpentyl 4-methoxybenzoate (4ak). Yield 33% (14.9 mg). Colorless gum. ^{1}H NMR (600 MHz, Methylene CD_2Cl_2) δ 8.50 – 8.31 (m, 2H), 7.93 (d, *J* = 8.5 Hz, 2H), 7.59 (d, *J* = 8.0 Hz, 2H), 7.25 (d, *J* = 8.0 Hz, 2H), 6.99 (d, *J* = 4.9 Hz, 2H), 6.93 (d, *J* = 8.7 Hz, 3H), 4.16 (t, *J* = 6.5 Hz, 2H), 3.85 (s, 3H), 3.43 (qd, *J* = 14.5, 6.6 Hz, 2H), 3.25 – 3.15 (m, 1H), 2.40 (s, 3H), 2.04 – 1.95 (m, 1H), 1.78 – 1.71 (m, 1H), 1.65 – 1.56 (m, 1H), 1.51 – 1.43 (m, 1H). ^{13}C NMR (150 MHz, CD_2Cl_2) δ 166.3, 163.8, 150.9, 150.4, 145.4, 137.0, 131.8, 130.2, 128.2, 123.3, 123.1, 114.0, 64.2, 61.7, 55.9, 40.4, 32.9, 26.6, 21.7.HRMS (ESI) m/z calcd. for $C_{25}H_{28}NO_5S$ $[M+H]^+$: 454.1688, found : 454.1688.

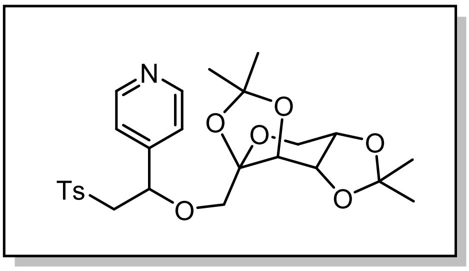

4-(1-(((3aR,5aS,8aS,8bR)-2,2,7,7-tetramethyltetrahydro-3a*H*-bis([1,3]dioxolo)[4,5-b:4',5'-d]pyran-3a-yl)methoxy)-2-tosylethyl)pyridine (4al). Diastereomeric mixture (4:1). Yield 73% (37.9 mg). Colorless gum. ^{1}H NMR (600 MHz, CD_2Cl_2, major isomer) δ 8.66 – 8.54 (m, 2H), 7.81 (d, *J* = 7.8 Hz, 2H), 7.40 (d, *J* = 7.9 Hz, 2H), 7.23 (d, *J* = 4.9 Hz, 2H), 4.79 (dd, *J* = 9.1, 2.7 Hz, 1H), 4.55 (dd, *J* = 7.8, 2.7 Hz, 1H), 4.18 (d, *J* = 7.9 Hz, 1H), 4.07 (d, *J* = 2.6 Hz, 1H), 3.84 – 3.78 (m, 1H), 3.60 (dd, *J* = 14.7, 9.6 Hz, 1H), 3.56 (d, *J* = 12.9 Hz, 1H), 3.36 – 3.29 (m, 2H), 3.29 – 3.23 (m, 1H), 2.47 (s, 3H), 1.50 (s, 3H), 1.42 (s, 3H), 1.38 (s, 3H), 1.36 (s, 3H). ^{13}C NMR (100 MHz, CD_2Cl_2, major isomer) δ 150.8, 147.9, 145.4, 137.2, 130.6, 128.4, 109.2, 109.0, 102.2, 76.0, 70.5, 70.4, 69.7, 63.3, 61.4, 26.7, 26.2, 25.6, 24.3, 21.8. HRMS (ESI) m/z calcd. for $C_{26}H_{34}NO_8S$ $[M+H]^+$: 520.2005, found : 520.2003.

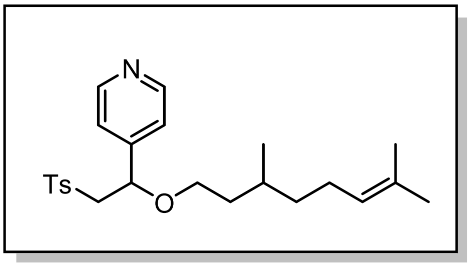

4-(1-((3,7-dimethyloct-6-en-1-yl)oxy)-2-tosylethyl)pyridine (4am). Diastereomeric mixture (1:1). Yield 72% (29.7 mg). Colorless gum. ^{1}H NMR (600 MHz, $CDCl_3$) δ 8.57 (d, *J* = 5.4 Hz, 2H), 7.79 (d, *J* = 8.0 Hz, 2H), 7.33 (d, *J* = 7.8 Hz, 2H), 7.19 (d, *J* = 5.0 Hz, 2H), 5.07 – 4.98 (m, 1H), 4.85 – 4.77 (m, 1H), 3.64 – 3.52 (m, 1H), 3.28 – 3.12 (m, 3H), 2.44 (s, 3H), 1.98 – 1.80 (m, 2H), 1.67 (s, 3H), 1.57 (s, 3H), 1.40 – 1.26 (m, 2H), 1.22 – 1.10 (m, 1H), 1.09 – 0.97 (m, 1H), 0.78 – 0.71 (m, 3H). ^{13}C NMR (100 MHz, $CDCl_3$) δ = 150.5, 148.8, 148.7, 144.8, 137.4, 131.4, 129.8, 128.2, 124.7, 124.6, 121.3, 75.9, 68.3, 63.2, 37.4, 36.9, 36.5, 36.3, 29.4, 29.3, 25.9, 25.5, 25.4, 21.8, 19.7, 19.3, 17.8. HRMS (ESI) m/z calcd. for $C_{24}H_{34}NO_3S$ $[M+H]^+$: 416.2259, found : 416.2260.

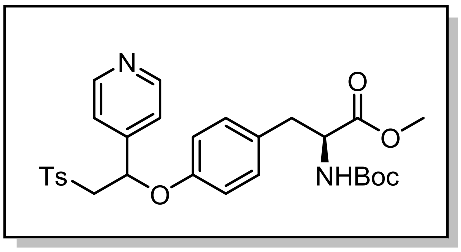

methyl (2S)-2-((tert-butoxycarbonyl)amino)-3-(4-(1-(pyridin-4-yl)-2-tosylethoxy)phenyl)propanoate (4an). Diastereomeric mixture (1:1) Yield 73% (40.7 mg). Pale-yellow solid (mp: 79–82 °C). ^{1}H NMR (600 MHz, $CDCl_3$) δ 8.71 – 8.45 (m, 2H), 7.71 (d, *J* = 7.9 Hz, 2H), 7.28 (d, *J* = 7.9 Hz, 2H), 7.24 (d, *J* = 4.8 Hz, 2H), 6.90 (d, *J* = 8.1 Hz, 2H), 6.45 (d, *J* = 8.1 Hz, 2H), 5.68 (dd, *J* = 9.3, 2.4 Hz, 1H), 4.95 (d, *J* = 8.3 Hz, 1H), 4.49 (d, *J* = 7.1 Hz, 1H), 3.80 (dd, *J* = 15.0, 9.3 Hz, 1H), 3.67 (s, 3H), 3.41 (dd, *J* = 15.0, 2.4 Hz, 1H), 2.99 (dd, *J* = 14.1, 5.7 Hz, 1H), 2.90 (dd, *J* = 14.1, 6.4 Hz, 1H), 2.43 (s, 3H), 1.38 (s, 9H). ^{13}C NMR (101 MHz, $CDCl_3$) δ 172.3, 155.3, 155.1, 150.5, 147.8, 145.0, 137.0, 137.0, 130.4, 129.9, 129.7, 128.3, 120.9, 115.7, 80.1, 73.9, 63.2, 54.5, 52.3, 37.6, 28.4, 21.8. HRMS (EI) m/z calcd. for $C_{29}H_{35}N_2O_7S$ $[M+H]^+$: 555.2165, found : 555.2168.

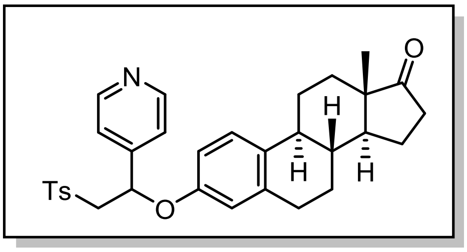

(8R,9S,13S,14S)-13-methyl-3-(1-(pyridin-4-yl)-2-tosylethoxy)-6,7,8,9,11,12,13,14,15,16-decahydro-17H-cyclopenta[a]phenanthren-17-one (4ao). Diastereomeric mixture (1:1) Yield 74% (39.3 mg). White-beige solid (mp: 79–82 ^{o}C). ^{1}H NMR (600 MHz, $CDCl_3$) δ 8.72 – 8.49 (m, 2H), 7.74 (d, *J* = 7.9 Hz, 2H), 7.35 (d, *J* = 4.7 Hz, 2H), 7.30 (d, *J* = 7.9 Hz, 2H), 7.05 (d, *J* = 8.6 Hz, 1H), 6.38 – 6.30 (m, 1H), 6.29 – 6.24 (m, 1H), 5.77 – 5.70 (m, 1H), 3.80 (dd, *J* = 14.9, 9.2 Hz, 1H), 3.43 (dd, *J* = 14.9, 2.7 Hz, 1H), 2.84 – 2.67 (m, 2H), 2.52 – 2.46 (m, 1H), 2.45 (s, 3H), 2.34 – 2.27 (m, 1H), 2.22 – 2.14 (m, 1H), 2.12 (dt, *J* = 18.7, 9.0 Hz, 1H), 2.06 – 1.99 (m, 1H), 1.99 – 1.89 (m, 2H), 1.64 – 1.56 (m, 1H), 1.54 – 1.42 (m, 4H), 1.42 – 1.33 (m, 1H), 0.88 (s, 3H). ^{13}C NMR (100 MHz, $CDCl_3$) δ 220.9, 154.1, 149.5, 149.3, 145.0, 138.0, 137.1, 133.7, 129.9, 128.4, 126.5, 121.5, 115.7, 115.6, 113.2, 113.0, 73.6, 63.0, 50.5, 48.0, 44.0, 38.3, 38.2, 35.9, 31.6, 29.7, 26.5, 25.9, 21.8, 21.7, 13.9. HRMS (EI) m/z calcd. for $C_{32}H_{35}NO_4S$ $[M]^+$: 529.2287, found : 529.2284.

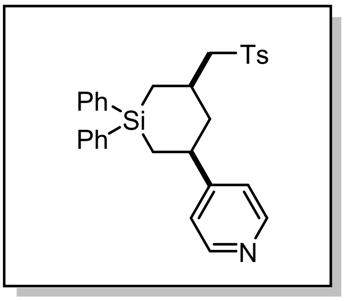

4-(1,1-diphenyl-5-(tosylmethyl)silinan-3-yl)pyridine (4ap). Yield 81% (40.4 mg). Colorless oil. The stereochemistry was assigned to be cis as confirmed by ^{1}H NMR, 2D NMR experiments by comparison with literature precedent.[19] White solid. ^{1}H NMR (500 MHz, $CDCl_3$) δ 8.50 (s, 2H), 7.83 – 7.71 (m, 2H), 7.65 – 7.59 (m, 2H), 7.52 – 7.43 (m, 2H), 7.42 (ddd, *J* = 8.0, 4.2, 1.8 Hz, 2H), 7.39 – 7.32 (m, 1H), 7.31 (dt, *J* = 6.6, 2.9 Hz, 3H), 7.14 – 7.08 (m, 2H), 3.13 (dd, *J* = 6.0, 1.7 Hz, 2H), 2.81 (ddt, *J* = 14.4, 12.3, 2.6 Hz, 1H), 2.50 – 2.35 (m, 4H), 2.00 (dt, *J* = 13.1, 2.3 Hz, 1H), 1.89 – 1.79 (m, 1H), 1.58 (dd, *J* = 14.7, 2.3 Hz, 1H), 1.42 (dt, *J* = 13.3, 11.9 Hz, 1H), 1.21 (t, *J* = 14.0 Hz, 1H), 0.92 (dd, *J* = 14.6, 13.1 Hz, 1H). ^{13}C NMR (100 MHz, $CDCl_3$) δ 157.6, 149.8, 144.7, 137.1, 135.5, 134.7, 134.3, 133.3, 130.0, 130.0, 129.9, 128.5, 128.1, 127.9, 121.9, 65.7, 43.1, 41.0, 32.0, 21.7, 18.5, 18.4. HRMS (ESI) m/z calcd. for $C_{30}H_{32}NO_2SSi$ $[M+H]^+$: 498.1923, found : 498.1923.

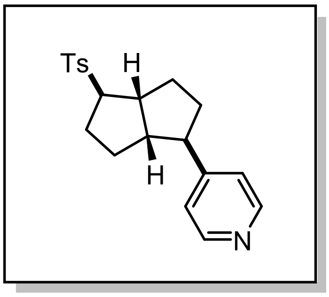

4-((3aR,6aS)-4-tosyloctahydropentalen-1-yl)pyridine (4aq). Yield 63% (21.4 mg). Colorless oil. The stereochemistry was assigned to be cis as confirmed by ^{1}H NMR, 1D-NOE, HSQC experiments by comparison with literature precedent.[21] ^{1}H NMR (400 MHz, $CDCl_3$) δ 8.85 – 8.22 (m, 2H), 7.77 (dd, *J* = 8.3, 2.7 Hz, 2H), 7.34 (dd, *J* = 8.3, 2.8 Hz, 2H), 7.08 (d, *J* = 4.9 Hz, 2H), 3.21 (q, *J* = 6.7 Hz, 1H), 3.15 – 3.00 (m, 1H), 2.73 – 2.57 (m, 2H), 2.43 (d, *J* = 3.4 Hz, 3H), 2.19 – 1.98 (m, 4H), 1.99 – 1.86 (m, 1H), 1.69 (dtd, *J* = 12.4, 9.6, 6.5 Hz, 1H), 1.51 – 1.35 (m, 1H), 1.27 (dqd, *J* = 13.2, 6.7, 3.1 Hz, 1H). ^{13}C NMR (100 MHz, $CDCl_3$) δ 153.5, 149.7, 144.7, 135.8, 129.9, 129.8, 128.6, 122.8, 72.0, 52.0, 51.8, 45.2, 35.8, 32.8, 31.2, 29.1, 21.7. HRMS (ESI) m/z calcd. for $C_{20}H_{24}NO_2S$ $[M+H]^+$: 342.1528, found : 342.1528.

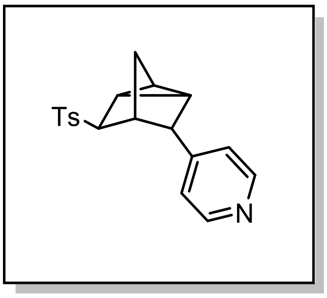

4-(5-tosyltricyclo[2.2.1.02,6]heptan-3-yl)pyridine (4ar). Yield 60% (19.6 mg). Yellowish gum. Diasteromeric ratio 4:1. ^{1}H NMR (400 MHz, $CDCl_3$, major isomer) δ 8.77 – 8.25 (m, 2H), 7.85 – 7.75 (m, 2H), 7.37 (d, *J* = 8.0 Hz, 2H), 7.21 – 7.09 (m, 2H), 3.26 (t, *J* = 1.1 Hz, 1H), 2.85 (s, 1H), 2.46 (s, 3H), 2.40 – 2.34 (m, 1H), 2.22 (dt, *J* = 11.6, 1.6 Hz, 1H), 1.78 – 1.66 (m, 4H), 1.15 (dt, *J* = 11.5, 1.3 Hz, 1H). ^{13}C NMR (100 MHz, $CDCl_3$, major isomer) δ 149.6, 148.8, 144.9, 136.9, 130.1, 128.2, 123.6, 70.8, 50.0, 38.8, 32.1, 26.4, 21.8, 15.7, 14.2, 12.0. HRMS (ESI) m/z calcd. for $C_{19}H_{20}NO_2S$ $[M+H]^+$: 326.1215, found : 326.1214.

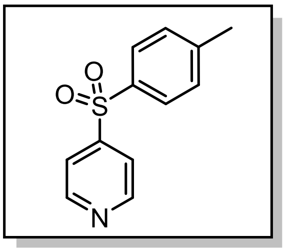

4-tosylpyridine (5a). Yield 80% (18.6 mg). White solid (mp: 131–133 °C). ^{1}H NMR (600 MHz, $CDCl_3$) δ 8.83 – 8.78 (m, 2H), 7.84 (d, *J* = 8.4 Hz, 2H), 7.77 – 7.71 (m, 2H), 7.35 (d, *J* = 8.1 Hz, 2H), 2.42 (s, 3H). ^{13}C NMR (150 MHz, $CDCl_3$) δ 151.3, 151.3, 150.2, 145.6, 136.8, 130.4, 128.3, 120.6, 21.8. HRMS (EI) m/z calcd. for $C_{32}H_{35}NO_4S$ $[M]^+$: 529.2287, found : 529.2284.

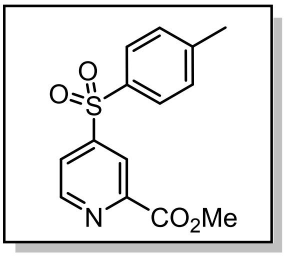

methyl 4-tosylpicolinate (5b). Yield 89% (0.063 mmol scale, 16.4mg). White solid (mp: 126–129 °C). ^{1}H NMR (600 MHz, $CDCl_3$) δ 8.92 (d, *J* = 5.0 Hz, 1H), 8.51 (s, 1H), 7.94 (dd, *J* = 5.0, 1.8 Hz, 1H), 7.86 (d, *J* = 8.1 Hz, 2H), 7.35 (d, *J* = 8.1 Hz, 3H), 4.02 (s, 3H), 2.42 (s, 3H). ^{13}C NMR (100 MHz, $CDCl_3$) δ 164.3, 151.9, 151.3, 149.7, 145.9, 136.3, 130.6, 128.5, 123.7, 122.2, 53.5, 21.8. HRMS (EI) m/z calcd. for $C_{32}H_{35}NO_4S$ $[M]^+$: 529.2287, found : 529.2284.

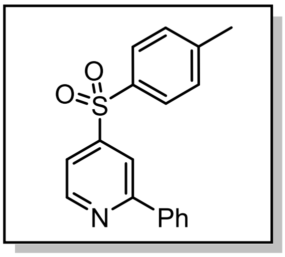

2-phenyl-4-tosylpyridine (5c). Yield 98% (30.4 mg). White solid (mp: 110–113 °C). ^{1}H NMR (600 MHz, $CDCl_3$) δ 8.85 (d, *J* = 5.1 Hz, 1H), 8.18 (t, *J* = 1.1 Hz, 1H), 8.07 – 7.96 (m, 2H), 7.95 – 7.79 (m, 2H), 7.65 (dd, *J* = 5.1, 1.6 Hz, 1H), 7.57 – 7.43 (m, 3H), 7.35 (d, *J* = 8.1 Hz, 2H), 2.42 (s, 3H). ^{13}C NMR (100 MHz, $CDCl_3$) δ 159.4, 151.1, 151.1, 145.5, 137.9, 137.0, 130.4, 130.2, 129.1, 128.3, 127.2, 118.8, 117.3, 21.8. HRMS (EI) m/z calcd. for $C_{18}H_{15}NO_2S$ $[M]^+$: 309.0824, found : 309.0821.

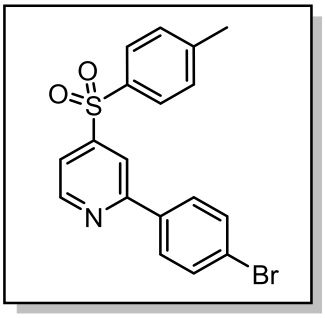

2-(4-bromophenyl)-4-tosylpyridine (5d). Yield 91% (35.5 mg). White solid (mp: 168–171 °C). ^{1}H NMR (400 MHz, $CDCl_3$) δ = 8.83 (dd, *J*=5.0, 0.8, 1H), 8.15 (dd, *J*=1.7, 0.8, 1H), 7.92 – 7.85 (m, 4H), 7.66 (dd, *J*=5.1, 1.6, 1H), 7.64 – 7.60 (m, 2H), 7.38 – 7.33 (m, 2H), 2.42 (s, 3H). ^{13}C NMR (100 MHz, $CDCl_3$) δ = 158.1, 151.3, 151.2, 145.6, 136.8, 136.7, 132.3, 130.5, 128.7, 128.3, 124.8, 119.2, 117.0, 21.8. HRMS (ESI) m/z calcd. for $C_{18}H_{15}NO_2S$ $[M+H]^+$: 388.0007, found : 338.0005.

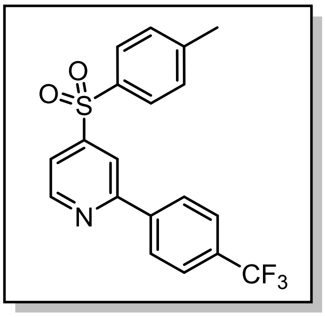

4-tosyl-2-(4-(trifluoromethyl)phenyl)pyridine (5e). Yield 97% (36.6 mg). White solid (mp: 167–170 °C). ^{1}H NMR (600 MHz, $CDCl_3$) δ 8.88 (d, *J* = 5.0 Hz, 1H), 8.22 (s, 1H), 8.14 (d, *J* = 8.0 Hz, 2H), 7.89 (d, *J* = 8.1 Hz, 2H), 7.75 (d, *J* = 8.1 Hz, 2H), 7.71 (dd, *J* = 5.0, 1.6 Hz, 1H), 7.36 (d, *J* = 7.9 Hz, 2H), 2.43 (s, 3H). ^{13}C NMR (100 MHz, $CDCl_3$) δ 157.8, 151.5, 151.4, 145.7, 141.1, 136.8, 131.9 (q, *J* = 32.6 Hz), 130.5, 128.4, 127.6, 126.1 (q, *J* = 3.8 Hz), 124.1 (q, *J* = 272.2 Hz), 119.8, 117.6, 21.8. ^{19}F NMR (376 MHz, $CDCl_3$) δ -62.7. HRMS (EI) m/z calcd. for $C_{19}H_{14}F_3NO_2S$ $[M]^+$: 377.0697, found : 377.0699.

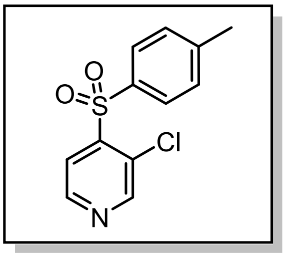

3-chloro-4-tosylpyridine (5f). Yield 59% (15.8 mg). White solid (mp: 117–120 ^{o}C). ^{1}H NMR (600 MHz, $CDCl_3$) δ 8.74 (d, *J* = 5.0 Hz, 1H), 8.67 (s, 1H), 8.11 (d, *J* = 5.0 Hz, 1H), 7.86 (d, *J* = 8.0 Hz, 2H), 7.35 (d, *J* = 8.0 Hz, 2H), 2.44 (s, 3H). ^{13}C NMR (100 MHz, $CDCl_3$) δ 152.2, 149.1, 146.5, 145.8, 135.6, 130.0, 129.2, 129.1, 123.0, 21.9. HRMS (EI) m/z calcd. for $C_{12}H_{10}ClNO_2S$ $[M]^+$: $C_{12}H_{10}ClNO_2S$, found : 267.0120.

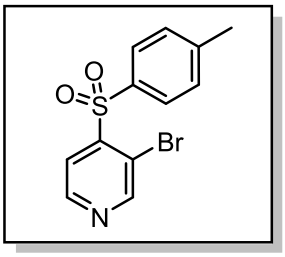

3-bromo-4-tosylpyridine (5g). Yield 70% (21.7 mg). White solid (mp: 72–75 ^{o}C). ^{1}H NMR (400 MHz, $CDCl_3$) δ 8.82 (s, 1H), 8.78 (d, *J* = 5.1 Hz, 1H), 8.15 (d, *J* = 5.0 Hz, 1H), 7.90 – 7.81 (m, 2H), 7.34 (d, *J* = 8.1 Hz, 2H), 2.44 (s, 3H). ^{13}C NMR (100 MHz, $CDCl_3$) δ 154.9, 149.6, 148.1, 145.8, 135.3, 129.9, 129.3, 123.7, 21.9. HRMS (ESI) m/z calcd. for $C_{12}H_{11}BrNO_2S$ $[M+H]^+$: 311.9696, found : 311.9694.

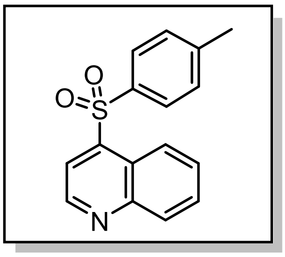

4-tosylquinoline (5h). Yield 91% (25.7 mg). Whtie solid (mp: 102–105 ^{o}C). ^{1}H NMR (600 MHz, $CDCl_3$) δ 9.11 (d, *J* = 4.4 Hz, 1H), 8.69 – 8.62 (m, 1H), 8.19 (dd, *J* = 8.5, 1.3 Hz, 1H), 8.14 (d, *J* = 4.4 Hz, 1H), 7.93 – 7.85 (m, 2H), 7.77 (ddd, *J* = 8.4, 6.9, 1.3 Hz, 1H), 7.65 (ddd, *J* = 8.4, 6.9, 1.3 Hz, 1H), 7.31 (d, *J* = 8.1 Hz, 2H), 2.38 (s, 3H). ^{13}C NMR (100 MHz, $CDCl_3$) δ 149.8, 149.6, 145.3, 145.3, 137.3, 130.7, 130.5, 130.2, 128.9, 128.2, 124.4, 122.3, 121.2, 21.8. HRMS (EI) m/z calcd. for $C_{16}H_{13}NO_2S$

[M]$^+$: 283.0667, found : 309.0821.

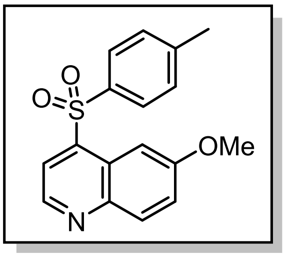

6-methoxy-4-tosylquinoline (5i). Yield 85% (0.030 mmol scale, 8.0 mg). Pale-yellow solid (mp: 149–152 ^{o}C). ^{1}H NMR (600 MHz, $CDCl_3$) δ = 8.95 (d, *J*=4.5, 1H), 8.13 (d, *J*=4.5, 1H), 8.06 (d, *J*=9.2, 1H), 7.89 – 7.85 (m, 3H), 7.39 (dd, *J*=9.2, 2.7, 1H), 7.31 (d, *J*=8.0, 2H), 3.93 (s, 3H), 2.40 (s, 3H). ^{13}C NMR (100 MHz, $CDCl_3$) δ = 159.3, 146.9, 146.0, 145.2, 143.2, 137.4, 132.1, 130.1, 128.0, 123.7, 123.5, 121.4, 102.1, 55.8, 21.8. HRMS (ESI) m/z calcd. for $C_{17}H_{16}NO_3S$ [M+H]$^+$: 314.0851, found : 314.0852.

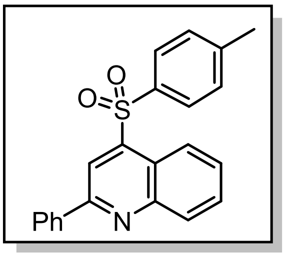

2-phenyl-4-tosylquinoline (5j). Yield 99% (35.5 mg). White solid (mp: 181–184 ^{o}C). ^{1}H NMR (500 MHz, $CDCl_3$) δ = 8.71 (s, 1H), 8.61 (d, *J*=8.5, 1H), 8.30 – 8.20 (m, 3H), 7.92 (d, *J*=8.1, 2H), 7.76 (ddd, *J*=8.3, 6.9, 1.3, 1H), 7.65 – 7.50 (m, 4H), 7.31 (d, *J*=8.1, 2H), 2.38 (s, 3H). ^{13}C NMR (125 MHz, $CDCl_3$) δ = 156.9, 149.7, 146.0, 145.1, 138.3, 137.6, 130.9, 130.6, 130.4, 130.2, 129.2, 128.4, 128.1, 127.7, 124.2, 121.2, 119.4, 21.8. HRMS (ESI) m/z calcd. for $C_{22}H_{18}NO_2S$ [M+H]$^+$: 360.1057, found : 360.1058.

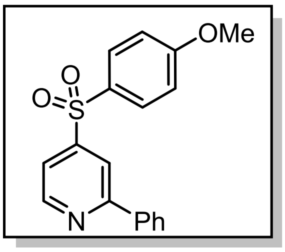

4-((4-methoxyphenyl)sulfonyl)-2-phenylpyridine (5k). Yield 96% (31.3 mg). White solid (mp: 100–103 °C). ^1H NMR (600 MHz, $CDCl_3$) δ 8.84 (d, *J* = 5.1 Hz, 1H), 8.17 (s, 1H), 8.01 (d, *J* = 7.5 Hz, 2H), 7.95 – 7.91 (m, 2H), 7.63 (d, *J* = 5.1 Hz, 1H), 7.49 (dt, *J* = 13.1, 7.8 Hz, 3H), 7.01 (dd, *J* = 8.8, 1.9 Hz, 2H), 3.86 (s, 3H). ^{13}C NMR (150 MHz, $CDCl_3$) δ 164.2, 159.4, 151.5, 151.1, 151.1, 137.9, 131.3, 130.5, 130.1, 129.1, 127.2, 118.7, 118.7, 117.2, 117.1, 115.0, 55.9. HRMS (ESI) m/z calcd. for $C_{17}H_{16}NO_3S$ $[M+H]^+$: 326.0851, found : 326.0851.

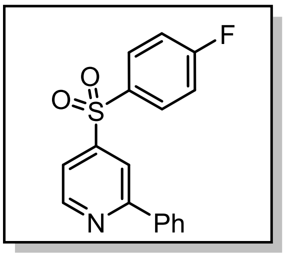

4-((4-fluorophenyl)sulfonyl)-2-phenylpyridine (5l). Yield 85% (26.6 mg). Pale-yellow solid (mp: 117–120 °C). ^1H NMR (600 MHz, $CDCl_3$) δ 8.87 (d, *J* = 5.0 Hz, 1H), 8.21 – 8.15 (m, 1H), 8.02 (td, *J* = 7.3, 3.7 Hz, 4H), 7.65 (dd, *J* = 5.1, 1.6 Hz, 1H), 7.56 – 7.42 (m, 3H), 7.23 (t, *J* = 8.4 Hz, 2H). ^{13}C NMR (100 MHz, $CDCl_3$) δ 166.1 (d, *J* = 257.8 Hz), 159.6, 151.2, 150.6, 137.7, 136.1 (d, *J* = 3.2 Hz), 131.2 (d, *J* = 9.7 Hz), 130.3, 129.2, 127.2, 118.8, 117.3, 117.2 (d, *J* = 22.8 Hz). ^{19}F NMR (376 MHz, $CDCl_3$) δ -102.3. HRMS (EI) m/z calcd. for $C_{17}H_{12}FNO_2S$ $[M]^+$: 313.0573, found : 313.0573.

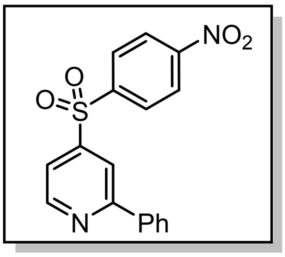

4-((4-nitrophenyl)sulfonyl)-2-phenylpyridine (5m). Yield 55% (18.7 mg). White solid (mp: 135–138 °C). ^1H NMR (500 MHz, $CDCl_3$) δ = 8.92 (dd, *J*=5.0, 0.8, 1H), 8.42 – 8.36 (m, 2H), 8.22 – 8.17 (m, 3H), 8.05 – 7.99 (m, 2H), 7.68 (dd, *J*=5.1, 1.7, 1H), 7.55 – 7.46 (m, 3H). ^{13}C NMR (125 MHz, $CDCl_3$) δ = 159.9, 151.5, 151.0, 149.3, 145.8, 137.5, 130.5, 129.6, 129.3, 127.3, 125.0, 118.9, 117.5. HRMS (ESI) m/z calcd. for $C_{17}H_{13}N_2O_4S$ $[M+H]^+$: 314.0596, found : 341.0595.

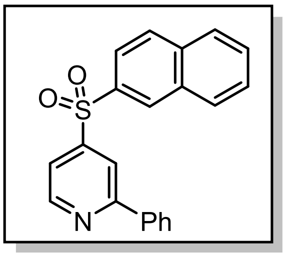

4-(naphthalen-2-ylsulfonyl)-2-phenylpyridine (5n). Yield 99% (34.4 mg). White solid (mp: 105–108 °C). ^{1}H NMR (600 MHz, $CDCl_3$) δ 8.86 (d, *J* = 5.1 Hz, 1H), 8.67 – 8.60 (m, 1H), 8.26 (s, 1H), 8.01 (d, *J* = 7.7 Hz, 3H), 7.98 (d, *J* = 8.7 Hz, 1H), 7.92 – 7.86 (m, 2H), 7.72 (dd, *J* = 5.0, 1.5 Hz, 1H), 7.65 (dt, *J* = 21.8, 7.2 Hz, 2H), 7.48 (dt, *J* = 12.7, 6.8 Hz, 3H). ^{13}C NMR (100 MHz, $CDCl_3$) δ 159.5, 151.1, 150.8, 137.8, 136.7, 135.5, 132.3, 130.2, 130.2, 130.1, 129.8, 129.6, 129.1, 128.1, 128.1, 127.3, 122.7, 119.0, 117.4. HRMS (EI) m/z calcd. for $C_{21}H_{15}NO_2S$ $[M]^+$: 345.0824, found : 345.0821.

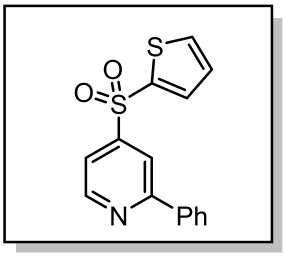

2-phenyl-4-(thiophen-2-ylsulfonyl)pyridine (5o). Yield 50% (15.1 mg). Colorless oil. ^{1}H NMR (600 MHz, $CDCl_3$) δ 8.88 (d, *J* = 5.1 Hz, 1H), 8.23 (d, *J* = 1.5 Hz, 1H), 8.06 – 7.98 (m, 2H), 7.80z (d, *J* = 3.8 Hz, 1H), 7.77 – 7.68 (m, 2H), 7.50 (dt, *J* = 13.0, 6.9 Hz, 3H), 7.15 (t, *J* = 4.4 Hz, 1H). ^{13}C NMR (100 MHz, $CDCl_3$) δ 159.5, 151.2, 151.1, 141.0, 137.8, 135.5, 134.9, 130.3, 129.2, 128.5, 127.3, 118.5, 117.0. HRMS (EI) m/z calcd. for $C_{15}H_{11}NO_2S_2$ $[M]^+$: 301.0231, found : 301.0233.

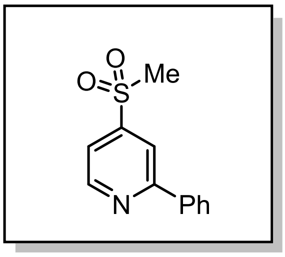

4-(methylsulfonyl)-2-phenylpyridine (5p). Yield 99% (23.2 mg). White solid (mp: 69–72 °C). ^{1}H NMR (400 MHz, $CDCl_3$) δ = 8.96 (dd, *J*=5.0, 0.9, 1H), 8.22 (dd, *J*=1.7, 0.8, 1H), 8.11 – 7.99 (m, 2H), 7.71 (dd, *J*=5.0, 1.6, 1H), 7.59 – 7.38 (m, 3H), 3.13 (s, 3H). ^{13}C NMR (100 MHz, $CDCl_3$) δ = 159.7,

151.4, 149.4, 137.7, 130.4, 129.2, 127.3, 118.6, 117.3, 44.1. HRMS (EI) m/z calcd. for $C_{12}H_{12}NO_2S$ $[M]^+$: 234.0590, found : 234.0589.

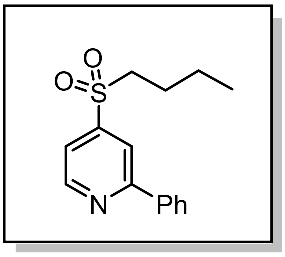

4-(butylsulfonyl)-2-phenylpyridine (5q). Yield 84% (23.1 mg). Pale-yellow solid (mp: 78–81 °C). ^{1}H NMR (400 MHz, $CDCl_3$) δ 8.95 (dd, J = 5.0, 0.9 Hz, 1H), 8.18 (dd, J = 1.7, 0.9 Hz, 1H), 8.12 – 8.02 (m, 2H), 7.67 (dd, J = 5.0, 1.6 Hz, 1H), 7.59 – 7.39 (m, 3H), 3.22 – 3.09 (m, 2H), 1.81 – 1.64 (m, 2H), 1.42 (h, J = 7.4 Hz, 2H), 0.91 (t, J = 7.3 Hz, 3H). ^{13}C NMR (100 MHz, $CDCl_3$) δ 159.5, 151.2, 148.2, 137.7, 130.3, 129.2, 127.2, 119.3, 117.9, 55.7, 24.5, 21.7, 13.6. HRMS (EI) m/z calcd. for $C_{15}H_{17}NO_2S$ $[M]^+$: 275.0980, found : 275.0978.

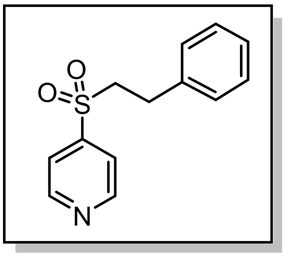

4-(phenethylsulfonyl)pyridine (5r). Yield 86% (21.3 mg). Yellow solid (mp: 87–90 °C). ^{1}H NMR (500 MHz, $CDCl_3$) δ = 8.99 – 8.80 (m, 2H), 7.78 – 7.74 (m, 2H), 7.31 – 7.18 (m, 3H), 7.13 – 7.08 (m, 2H), 3.44 – 3.37 (m, 2H), 3.11 – 3.03 (m, 2H).^{13}C NMR (125 MHz, $CDCl_3$) δ = 151.5, 147.2, 136.9, 129.0, 128.4, 127.3, 121.3, 57.2, 28.6. HRMS (ESI) m/z calcd. for $C_{13}H_{13}NO_2S$ $[M]^+$: 247.0667, found : 247.0666.

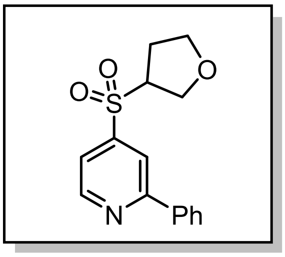

2-phenyl-4-((tetrahydrofuran-3-yl)sulfonyl)pyridine (5s). Yield 97% (28.2 mg). Pale-yellow solid

(mp: 94–97 °C). ^{1}H NMR (400 MHz, $CDCl_3$) δ = 8.99 (dd, *J*=5.0, 0.8, 1H), 8.21 (dd, *J*=1.6, 0.8, 1H), 8.11 – 8.06 (m, 2H), 7.70 (dd, *J*=5.0, 1.6, 1H), 7.59 – 7.49 (m, 3H), 4.27 (dd, *J*=10.2, 5.4, 1H), 4.05 – 3.93 (m, 2H), 3.93 – 3.80 (m, 2H), 2.51 – 2.40 (m, 1H), 2.24 (dddd, *J*=13.5, 9.5, 7.4, 6.2, 1H). ^{13}C NMR (125 MHz, $CDCl_3$) δ = 159.7, 151.4, 147.5, 137.5, 130.5, 129.2, 127.3, 119.6, 118.2, 68.5, 67.5, 63.7, 27.7. HRMS (ESI) m/z calcd. for $C_{15}H_{16}NO_3S$ $[M+H]^+$: 290.0853, found : 290.0851.

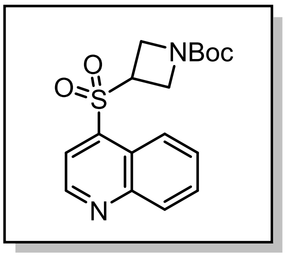

***tert*-butyl 3-(quinolin-4-ylsulfonyl)azetidine-1-carboxylate (5t).** Yield 81% (0.072 mmol scale, 20.3 mg). Yellow gum. ^{1}H NMR (500 MHz, $CDCl_3$) δ = 9.16 (d, *J*=4.4, 1H), 8.66 (dd, *J*=8.6, 1.3, 1H), 8.29 (dd, *J*=8.4, 1.3, 1H), 8.09 (d, *J*=4.3, 1H), 7.89 (ddd, *J*=8.4, 6.9, 1.3, 1H), 7.78 (ddd, *J*=8.3, 6.9, 1.3, 1H), 4.37 – 4.29 (m, 2H), 4.21 (tt, *J*=8.4, 5.4, 1H), 4.09 – 4.02 (m, 2H), 1.43 (s, 9H). ^{13}C NMR (100 MHz, $CDCl_3$) δ = 155.8, 149.8, 149.7, 140.8, 131.2, 131.0, 129.7, 123.8, 122.9, 122.6, 80.9, 51.3, 49.5, 28.4. HRMS (ESI) m/z calcd. for $C_{17}H_{21}N_2O_4S$ $[M+H]^+$: 349.1223, found : 349.1222.

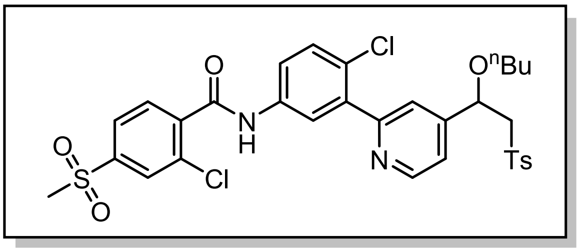

***N*-(3-(4-(1-butoxy-2-tosylethyl)pyridin-2-yl)-4-chlorophenyl)-2-chloro-4-(methylsulfonyl) benzamide (6).** Yield 98% (66.0 mg). White solid (mp: 105–108 °C). ^{1}H NMR (600 MHz, $CDCl_3$) δ 9.69 (s, 1H), 8.34 (d, *J* = 5.1 Hz, 1H), 7.92 – 7.89 (m, 1H), 7.80 (s, 1H), 7.77 (d, *J* = 8.0 Hz, 2H), 7.73 (d, *J* = 2.7 Hz, 1H), 7.65 (d, *J* = 8.0 Hz, 1H), 7.56 (s, 1H), 7.54 (d, *J* = 7.9 Hz, 1H), 7.44 (d, *J* = 8.7 Hz, 1H), 7.33 (d, *J* = 8.0 Hz, 2H), 7.11 (d, *J* = 5.1 Hz, 1H), 4.83 (dd, *J* = 9.0, 2.8 Hz, 1H), 3.58 (dd, *J* = 14.8, 9.1 Hz, 1H), 3.26 (dd, *J* = 14.8, 2.8 Hz, 1H), 3.21 (d, *J* = 6.6 Hz, 2H), 2.99 (s, 3H), 2.43 (s, 3H), 1.35 – 1.23 (m, 2H), 1.20 – 1.08 (m, 2H), 0.79 (t, *J* = 7.4 Hz, 3H). ^{13}C NMR (150 MHz, $CDCl_3$) δ 163.9, 156.4, 149.8, 149.3, 144.8, 142.7, 140.7, 138.4, 137.3, 137.1, 132.4, 131.0, 130.1, 129.8, 128.9, 128.1,

127.4, 125.8, 122.9, 122.9, 121.9, 120.6, 75.6, 70.0, 63.0, 44.4, 31.5, 21.7, 19.2, 14.0. HRMS (EI) m/z calcd. for $C_{33}H_{33}Cl_2N_2O_6S_2$ [M+H]$^+$: 675.1157, found : 675.1159.

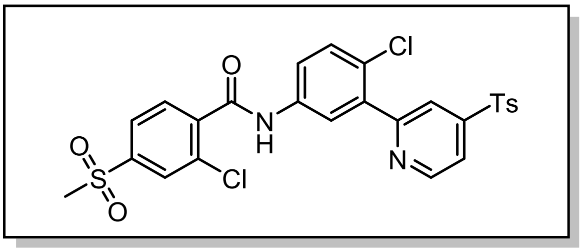

2-chloro-*N*-(4-chloro-3-(4-tosylpyridin-2-yl)phenyl)-4-(methylsulfonyl)benzamide (7). Yield 62% (35.8 mg). White solid (mp: 104–107 °C). ^{1}H NMR (600 MHz, CDCl$_3$) δ 8.83 (s, 1H), 8.80 (d, *J* = 5.1 Hz, 1H), 8.14 (s, 1H), 7.87 (d, *J* = 2.6 Hz, 1H), 7.86 – 7.82 (m, 3H), 7.79 (dd, *J* = 8.7, 2.6 Hz, 1H), 7.71 (d, *J* = 12.4 Hz, 3H), 7.46 (d, *J* = 8.7 Hz, 1H), 7.35 (d, *J* = 7.9 Hz, 2H), 3.03 (s, 3H), 2.42 (s, 3H). ^{13}C NMR (100 MHz, CDCl$_3$) δ 163.5, 157.8, 150.9, 150.4, 145.7, 142.8, 140.5, 138.0, 136.8, 136.6, 132.5, 131.2, 130.7, 130.5, 129.1, 128.3, 127.9, 126.0, 123.1, 122.3, 122.0, 119.4, 44.6, 21.8. HRMS (ESI) m/z calcd. for $C_{26}H_{21}Cl_2N_2O_5S_2$ [M+H]$^+$: 575.0270, found : 575.0269.

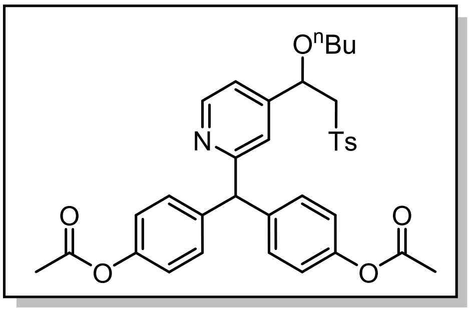

((4-(1-butoxy-2-tosylethyl)pyridin-2-yl)methylene)bis(4,1-phenylene) diacetate (8). MeOH was used as a solvent, instead of DMSO. Yield 44% (0.044 mmol scale, 11.9 mg). Pale-yellow gum. ^{1}H NMR (500 MHz, CDCl$_3$) δ 8.56 – 8.52 (m, 1H), 7.80 – 7.74 (m, 2H), 7.32 (d, *J* = 8.1 Hz, 2H), 7.19 – 7.13 (m, 4H), 7.07 – 7.03 (m, 2H), 7.03 – 6.98 (m, 4H), 5.59 (s, 1H), 4.77 (dd, *J* = 9.2, 2.7 Hz, 1H), 3.55 (dd, *J* = 14.7, 9.2 Hz, 1H), 3.22 – 3.16 (m, 2H), 3.16 – 3.08 (m, 1H), 2.44 (s, 3H), 2.28 (d, *J* = 3.2 Hz, 7H), 1.31 – 1.17 (m, 2H), 1.17 – 1.02 (m, 2H), 0.78 (t, *J* = 7.3 Hz, 3H). ^{13}C NMR (100 MHz, CDCl$_3$) δ 169.5, 163.6, 150.3, 149.7, 149.5, 144.8, 139.9, 139.8, 137.5, 130.3, 129.8, 128.2, 121.7, 121.2, 119.4, 75.7, 69.8, 63.3, 58.3, 31.6, 21.8, 21.3, 19.2, 14.0. HRMS (ESI) m/z calcd. for $C_{35}H_{38}NO_7S$ [M+H]$^+$: 616.2369, found : 616.2369.z

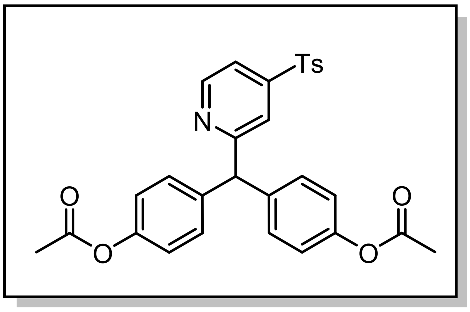

((4-tosylpyridin-2-yl)methylene)bis(4,1-phenylene) diacetate (9). Yield 53% (27.4 mg). Pale-green solid (mp: 77–80 °C). ^{1}H NMR (600 MHz, $CDCl_3$) δ 8.75 (d, *J* = 5.1 Hz, 1H), 7.77 (d, *J* = 7.9 Hz, 2H), 7.60 (s, 1H), 7.57 (dd, *J* = 5.1, 1.6 Hz, 1H), 7.32 (d, *J* = 7.9 Hz, 2H), 7.15 (d, *J* = 8.2 Hz, 4H), 7.01 (d, *J* = 8.2 Hz, 4H), 5.65 (s, 1H), 2.42 (s, 3H), 2.28 (s, 6H). ^{13}C NMR (100 MHz, $CDCl_3$) δ 169.5, 164.8, 151.1, 151.0, 149.7, 145.5, 139.1, 136.7, 130.4, 130.3, 128.3, 121.8, 120.5, 118.5, 58.1, 21.8, 21.3. HRMS (ESI) m/z calcd. for $C_{29}H_{26}NO_6S$ $[M+H]^+$: 516.1479, found : 516.1481.

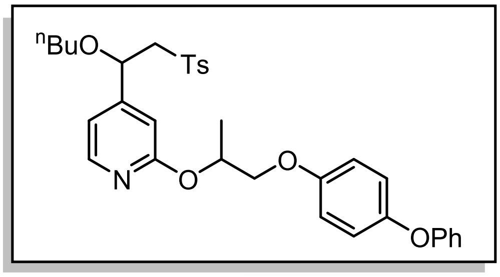

4-(1-butoxy-2-tosylethyl)-2-((1-(4-phenoxyphenoxy)propan-2-yl)oxy)pyridine (10). Diastereomeric mixture (1:1). Yield 66% (38.0 mg). Colorless oil. ^{1}H NMR (400 MHz, $CDCl_3$) δ 8.09 (d, *J* = 5.2 Hz, 1H), 7.79 (d, *J* = 8.3 Hz, 2H), 7.37 – 7.30 (m, 2H), 7.32 – 7.26 (m, 2H), 7.08 – 6.99 (m, 1H), 6.99 – 6.87 (m, 6H), 6.77 (d, *J* = 5.3 Hz, 1H), 6.64 (s, 1H), 5.61 – 5.49 (m, 1H), 4.75 (d, *J* = 9.3 Hz, 1H), 4.21 – 4.12 (m, 1H), 4.10 – 4.01 (m, 1H), 3.57 (dd, *J* = 14.7, 9.3 Hz, 1H), 3.26 – 3.14 (m, 3H), 2.44 (s, 3H), 1.50 – 1.43 (m, 3H), 1.35 – 1.23 (m, 2H), 1.24 – 1.09 (m, 2H), 0.81 (t, *J* = 7.3 Hz, 3H). ^{13}C NMR (100 MHz, $CDCl_3$) δ 163.8, 158.5, 155.2, 151.9, 150.4, 147.6, 144.7, 137.5, 129.8, 129.7, 128.2, 122.6, 120.8, 117.7, 115.9, 115.9, 114.5, 114.5, 109.2, 109.2, 75.7, 71.1, 69.9, 69.7, 63.1, 31.6, 21.7, 19.2, 17.1, 14.0. HRMS (EI) m/z calcd. for $C_{33}H_{38}NO_6S$ $[M+H]^+$: 576.2420, found : 576.2418.

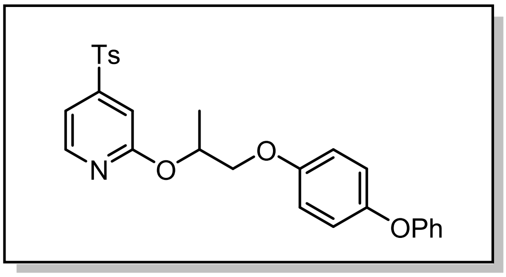

2-((1-(4-phenoxyphenoxy)propan-2-yl)oxy)-4-tosylpyridine (11). Yield 73% (34.5 mg). White solid (mp: 99–102 °C). ^{1}H NMR (400 MHz, CDCl$_3$) δ 8.28 (dd, *J* = 5.3, 0.7 Hz, 1H), 7.84 (d, *J* = 8.4 Hz, 2H), 7.35 – 7.32 (m, 2H), 7.32 – 7.25 (m, 3H), 7.21 (dd, *J* = 1.5, 0.7 Hz, 1H), 7.08 – 7.01 (m, 1H), 6.99 – 6.91 (m, 4H), 6.90 – 6.86 (m, 2H), 5.65 – 5.51 (m, 1H), 4.15 (dd, *J* = 10.0, 5.6 Hz, 1H), 4.06 (dd, *J* = 10.0, 4.5 Hz, 1H), 2.42 (s, 3H), 1.46 (d, *J* = 6.4 Hz, 3H). ^{13}C NMR (100 MHz, CDCl$_3$) δ 164.0, 158.5, 155.1, 152.9, 150.6, 148.6, 145.3, 136.9, 130.3, 129.7, 128.4, 122.6, 120.9, 117.8, 115.8, 113.4, 109.8, 70.9, 21.8, 16.9. HRMS (ESI) m/z calcd. for $C_{27}H_{26}NO_5S$ [M+H]$^+$: 476.1530, found : 476.1532.

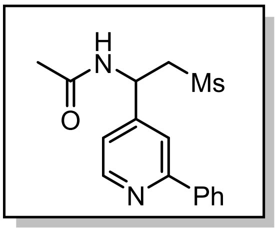

***N*-(2-(methylsulfonyl)-1-(2-phenylpyridin-4-yl)ethyl)acetamide (4ba).** Yield 72 % (0.15 mmol scale, 34.3 mg). Yellow gum. ^{1}H NMR (400 MHz, CDCl$_3$) δ = 8.70 (d, *J*=5.1, 1H), 8.02 – 7.94 (m, 2H), 7.72 (s, 1H), 7.54 – 7.39 (m, 3H), 7.22 (dd, *J*=5.0, 1.7, 1H), 7.10 (d, *J*=7.7, 1H), 5.64 (td, *J*=7.9, 4.4, 1H), 3.70 (dd, *J*=14.8, 8.1, 1H), 3.50 (dd, *J*=14.8, 4.4, 1H), 2.84 (s, 3H), 2.09 (s, 3H). ^{13}C NMR (100 MHz, CDCl$_3$) δ = 170.4, 158.6, 150.5, 148.6, 138.8, 129.6, 129.0, 127.2, 119.8, 118.5, 58.3, 48.5, 42.3, 23.4. HRMS (ESI) m/z calcd. for $C_{16}H_{19}N_2O_3S$ [M+H]$^+$: 319.1116, found : 319.1115.

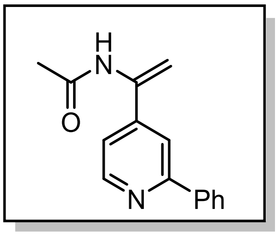

***N*-(1-(2-phenylpyridin-4-yl)vinyl)acetamide (12).** Yield 51 % (12.1 mg). Yellow gum. ^{1}H NMR (600 MHz, CDCl$_3$) δ = 8.69 (d, *J*=5.2, 1H), 7.98 (d, *J*=7.5, 2H), 7.72 (s, 1H), 7.52 – 7.46 (m, 2H), 7.46 –

7.41 (m, 1H), 7.24 (d, *J*=5.1, 1H), 6.86 (s, 1H), 5.96 (s, 1H), 5.33 (s, 1H), 2.18 (s, 3H). ^{13}C NMR (125 MHz, $CDCl_3$) δ = 169.2, 158.5, 150.3, 146.5, 139.1, 139.0, 129.4, 129.0, 127.1, 119.0, 117.5, 106.0, 24.6. HRMS (ESI) m/z calcd. for $C_{15}H_{15}N_2O$ $[M+H]^+$: 239.1184, found : 239.1184.

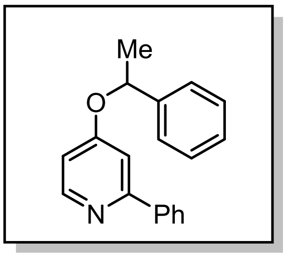

2-phenyl-4-(1-phenylethoxy)pyridine (13). Yield 61% (16.6 mg). Pale-yellow gum. ^{1}H NMR (500 MHz, $CDCl_3$) δ = 8.41 (d, *J*=5.7, 1H), 7.90 – 7.85 (m, 2H), 7.47 – 7.41 (m, 2H), 7.41 – 7.34 (m, 5H), 7.32 – 7.27 (m, 1H), 7.22 (d, *J*=2.4, 1H), 6.70 (dd, *J*=5.7, 2.4, 1H), 5.45 (q, *J*=6.4, 1H), 1.70 (d, *J*=6.4, 3H). ^{13}C NMR (125 MHz, $CDCl_3$) δ = 164.9, 159.2, 150.9, 142.1, 139.6, 129.0, 129.0, 128.8, 128.1, 127.0, 125.6, 109.8, 108.8, 76.3, 24.4. HRMS (EI) m/z calcd. for $C_{19}H_{17}NO$ $[M]^+$: 275.1310, found : 275.1307.

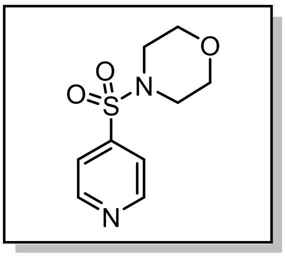

4-(pyridin-4-ylsulfonyl)morpholine (15). Yield 85% (19.5 mg). Pale-yellow solid (mp: 136–139 ^{o}C). ^{1}H NMR (600 MHz, MeOD) δ = 8.87 (d, *J*=5.0, 2H), 7.74 (d, *J*=5.3, 1H), 3.71 (t, *J*=4.7, 2H), 3.04 (t, *J*=4.8, 2H). ^{13}C NMR (100 MHz, MeOD) δ = 152.1, 145.6, 122.7, 67.2, 47.3. HRMS (ESI) m/z calcd. for $C_9H_{13}N_2O_3S$ $[M+H]^+$: 229.0648, found : 229.0647.

Appendix Ⅰ

Spectral Copies of ^{1}H-, ^{13}C-, and ^{19}F-NMR Data Obtained in this Study

4-(1-butoxy-2-tosylethyl)pyridine (4a).

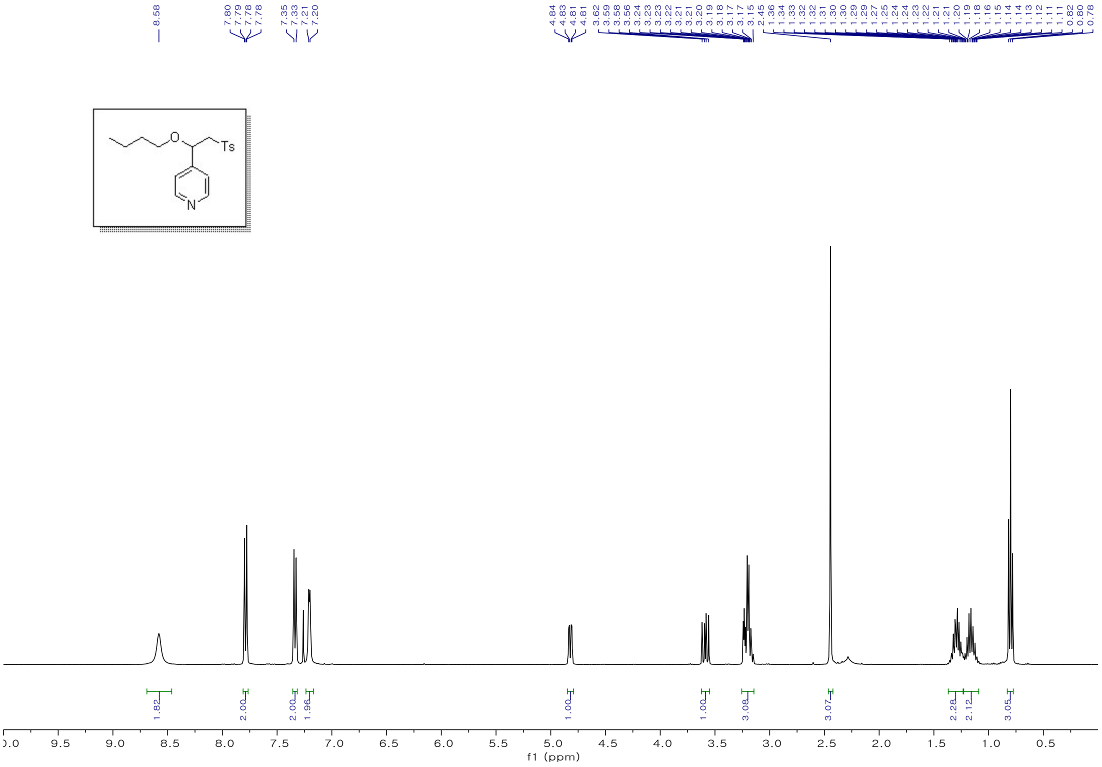

400 MHz, ^{1}H NMR in $CDCl_3$.

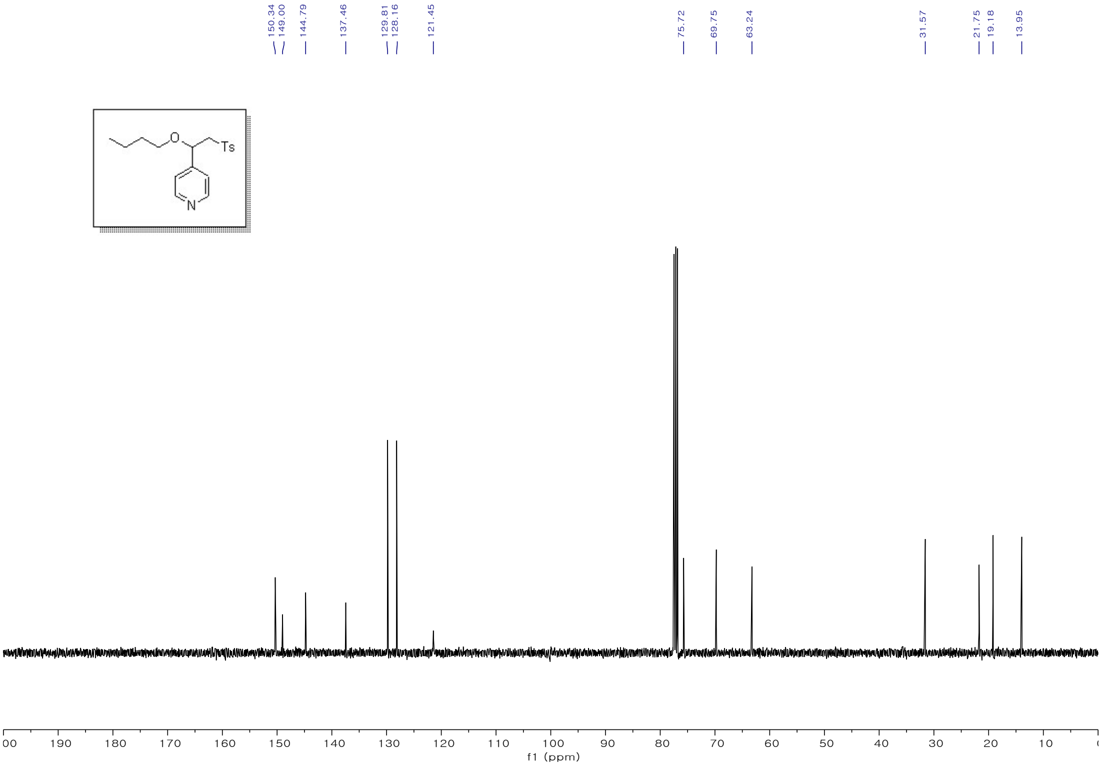

100 MHz, ^{13}C NMR in $CDCl_3$.

4-(1-butoxy-2-tosylethyl)-2-phenylpyridine (4b).

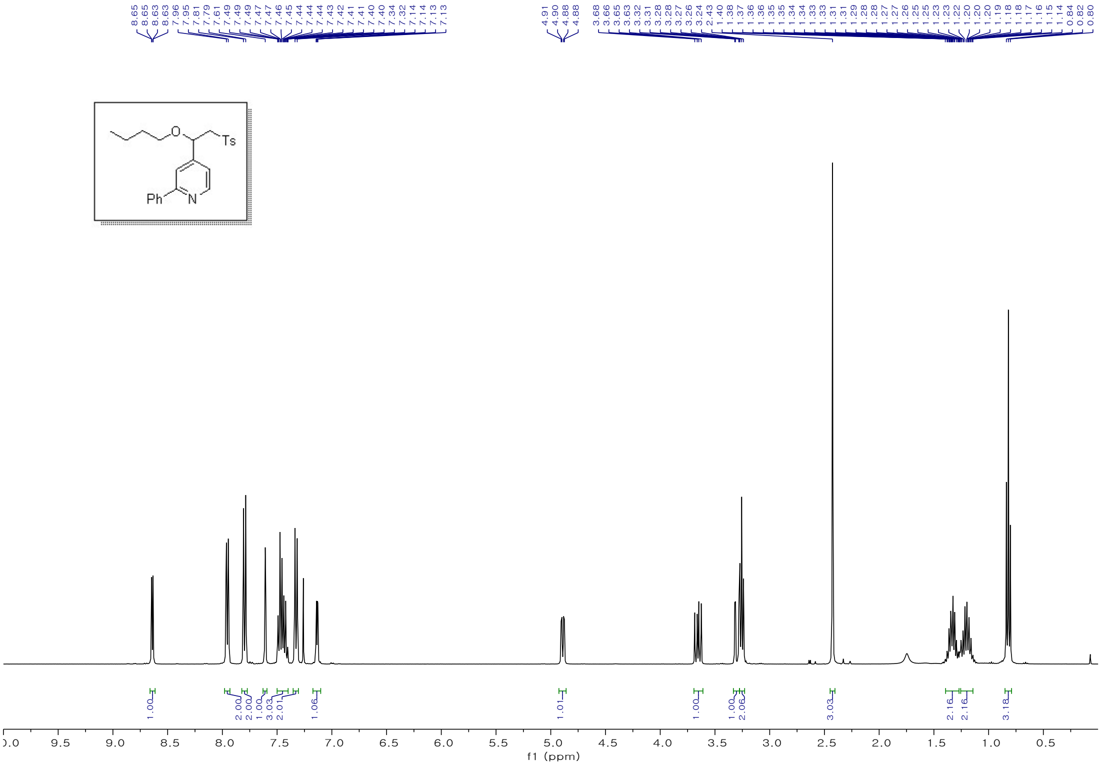

400 MHz, ^{1}H NMR in $CDCl_3$.

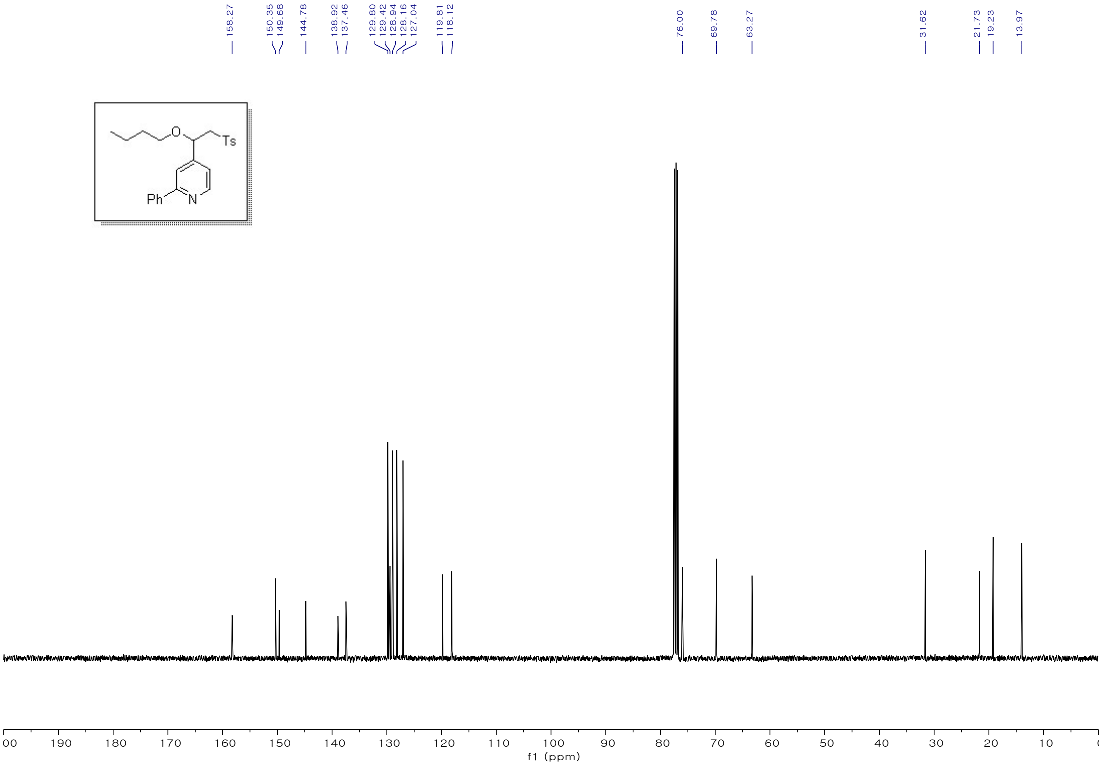

100 MHz, ^{13}C NMR in $CDCl_3$.

4-(1-butoxy-2-tosylethyl)-2-(4-methoxyphenyl)pyridine (4c).

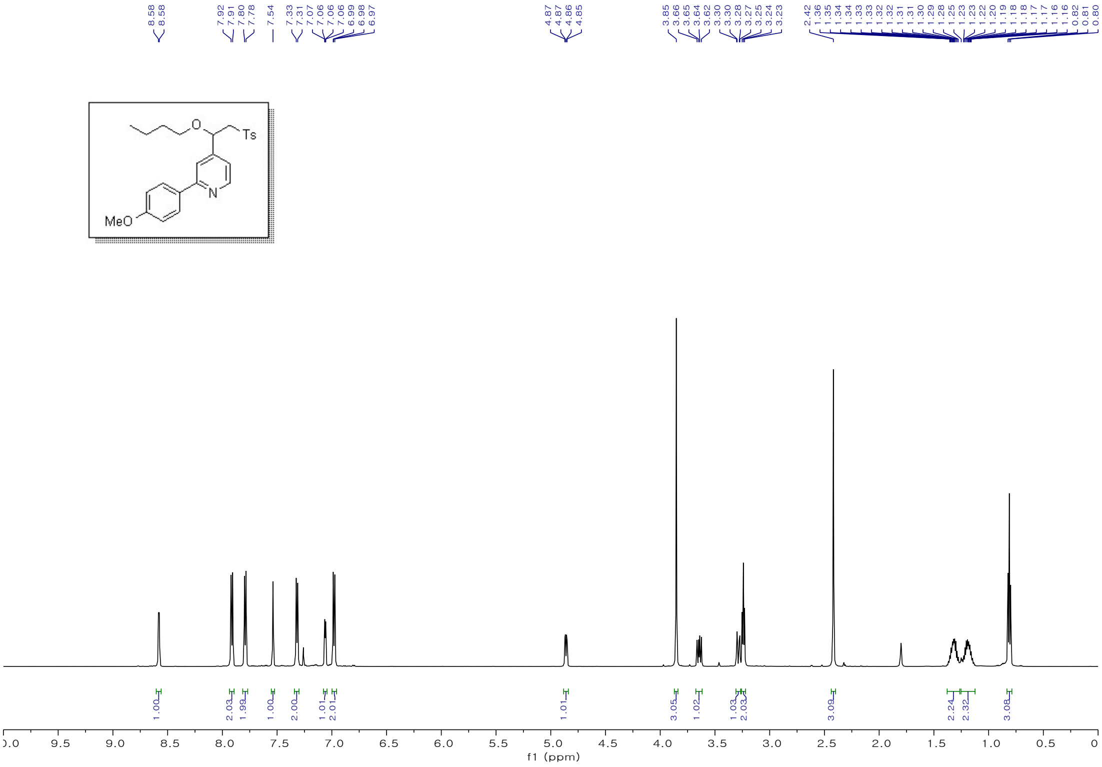

600 MHz, ^{1}H NMR in $CDCl_3$.

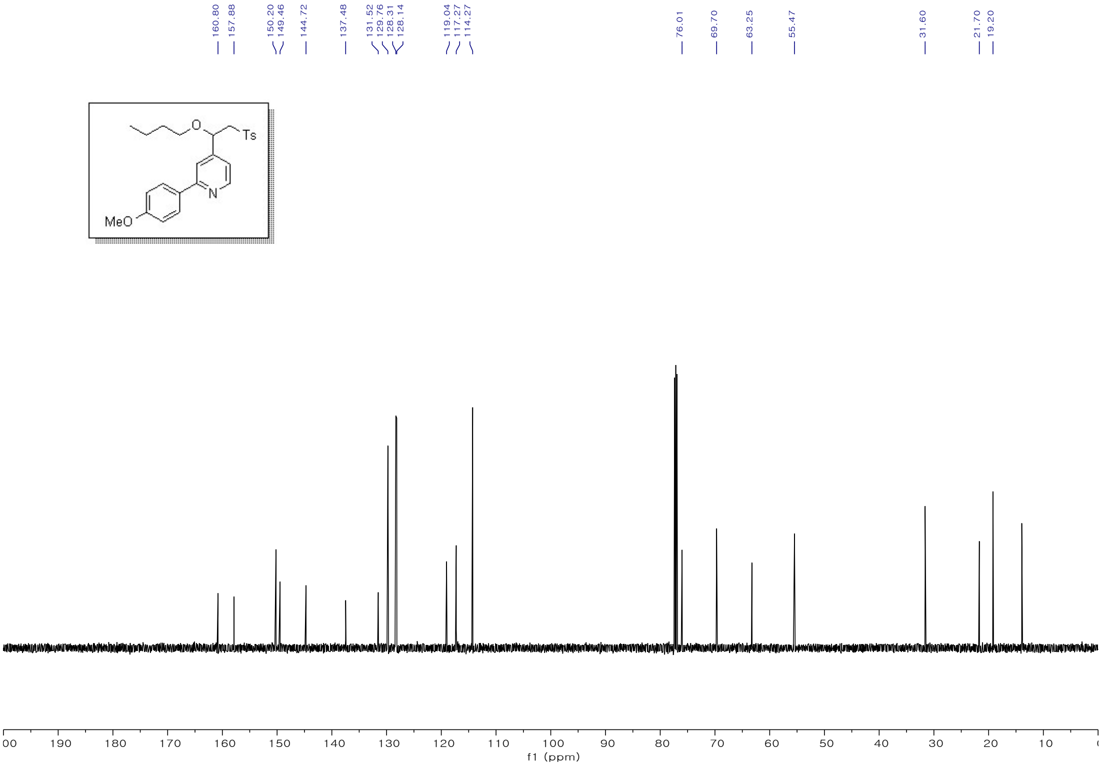

150 MHz, ^{13}C NMR in $CDCl_3$.

4-(1-butoxy-2-tosylethyl)-2-(4-(trifluoromethyl)phenyl)pyridine (4d).

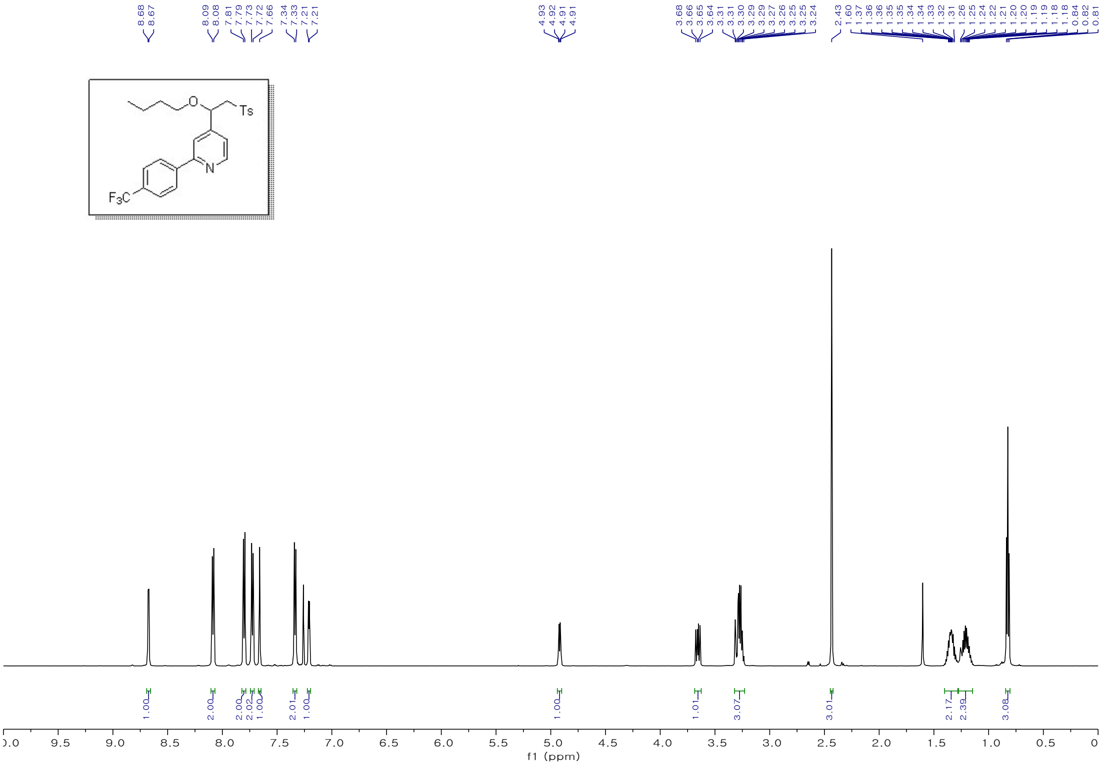

400 MHz, ^{1}H NMR in $CDCl_3$.

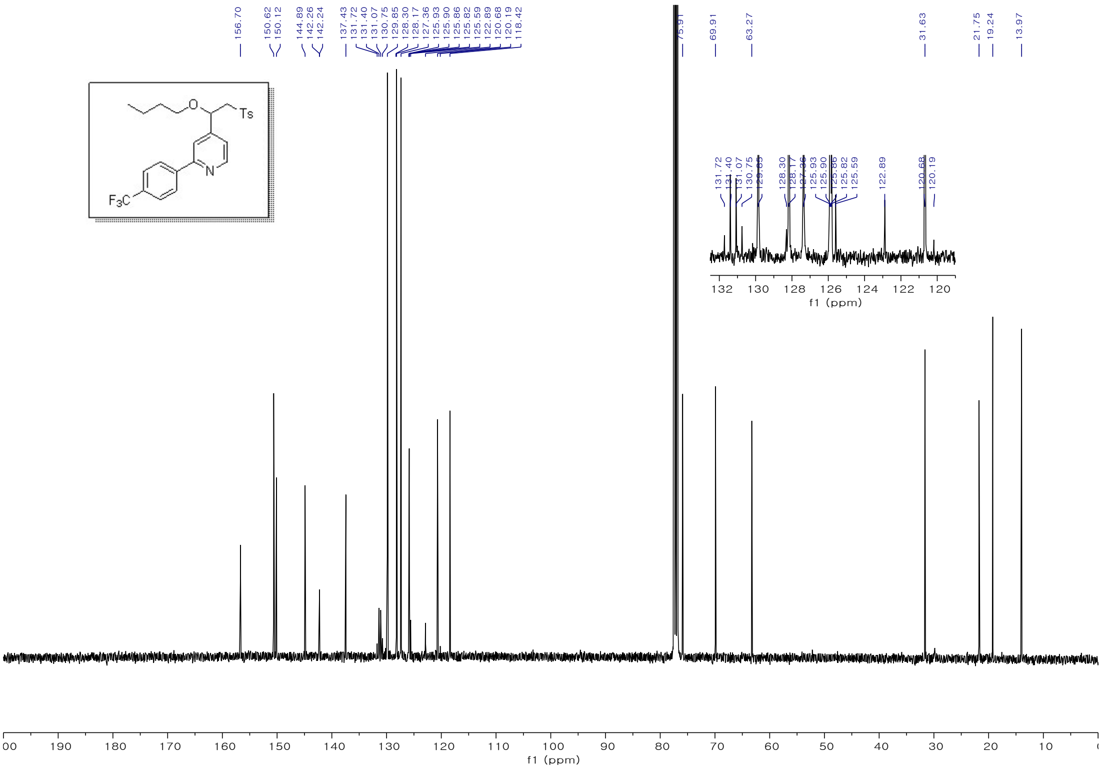

100 MHz, ^{13}C NMR in $CDCl_3$.

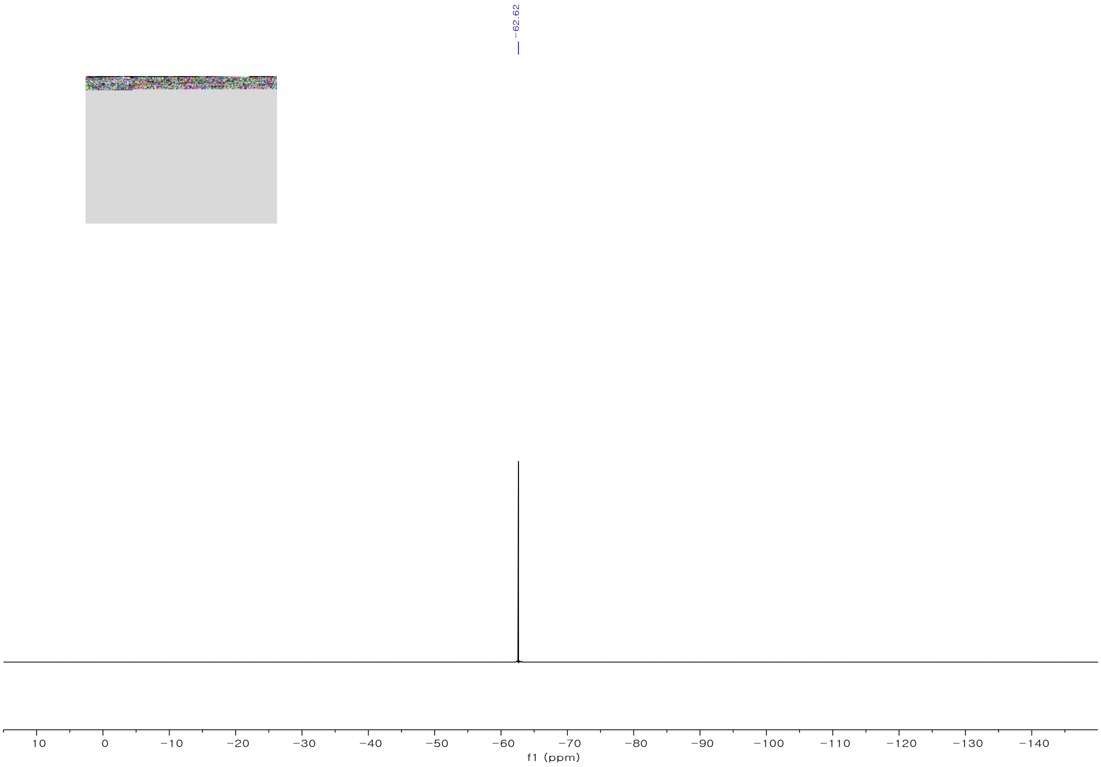

376 MHz, ^{19}F NMR in $CDCl_3$.

4-(1-butoxy-2-tosylethyl)-2,6-dimethylpyridine (4e).

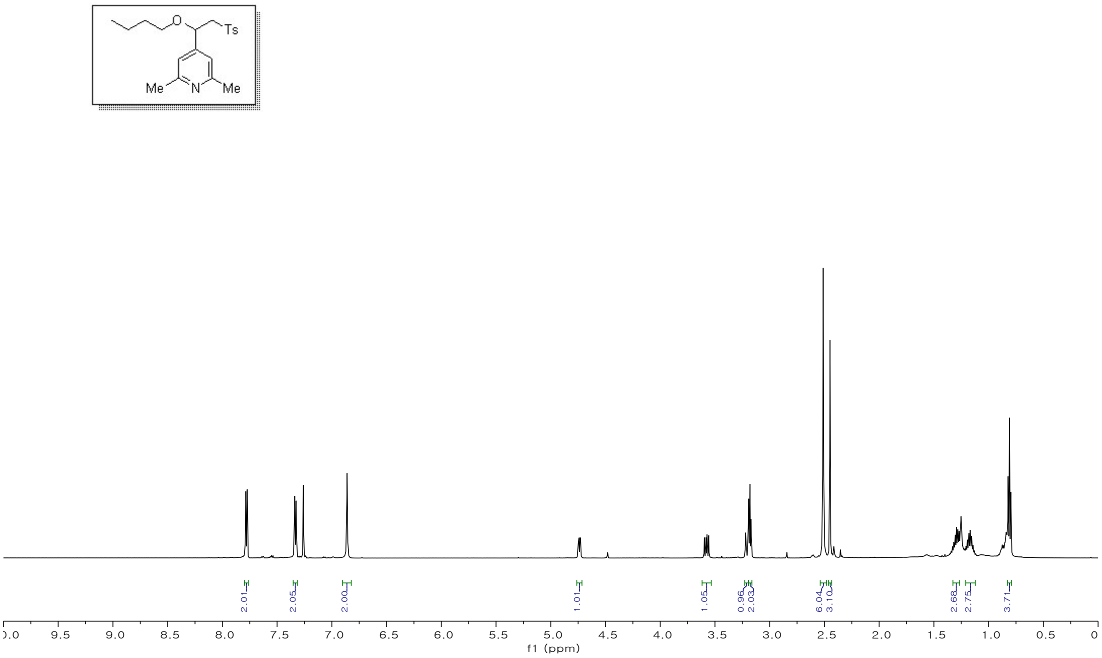

600 MHz, ^{1}H NMR in $CDCl_3$.

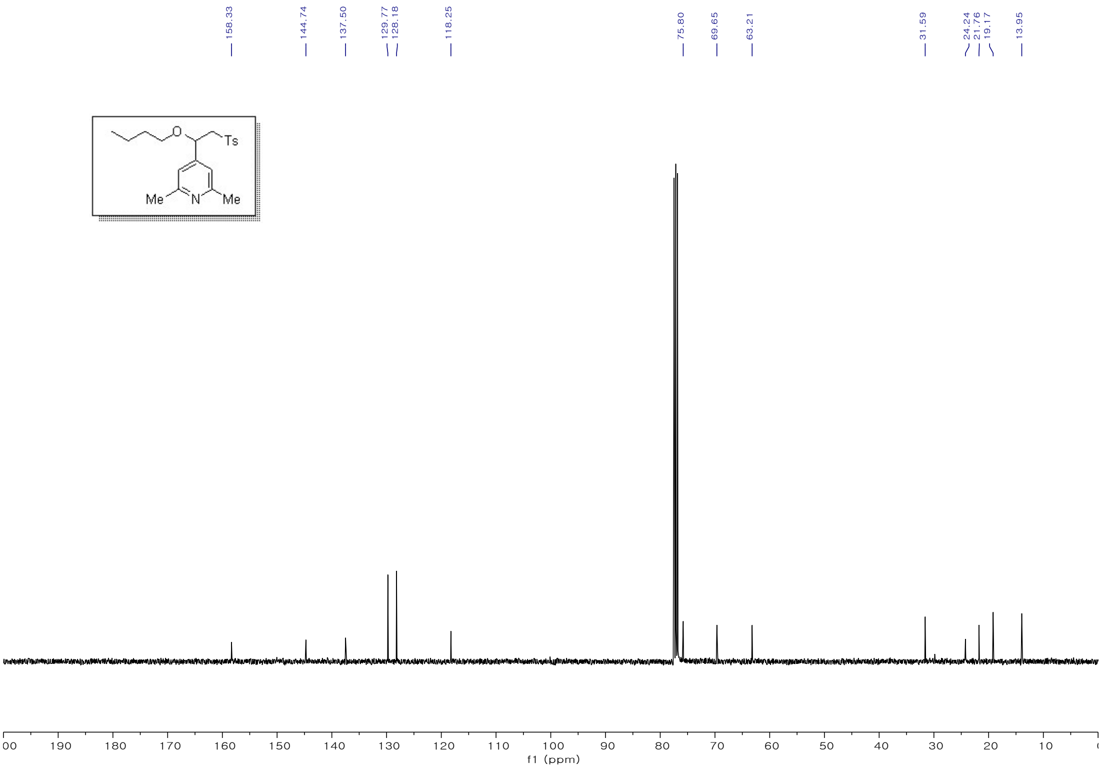

100 MHz, ^{13}C NMR in $CDCl_3$.

4-(1-butoxy-2-tosylethyl)-3-methylpyridine (4f).

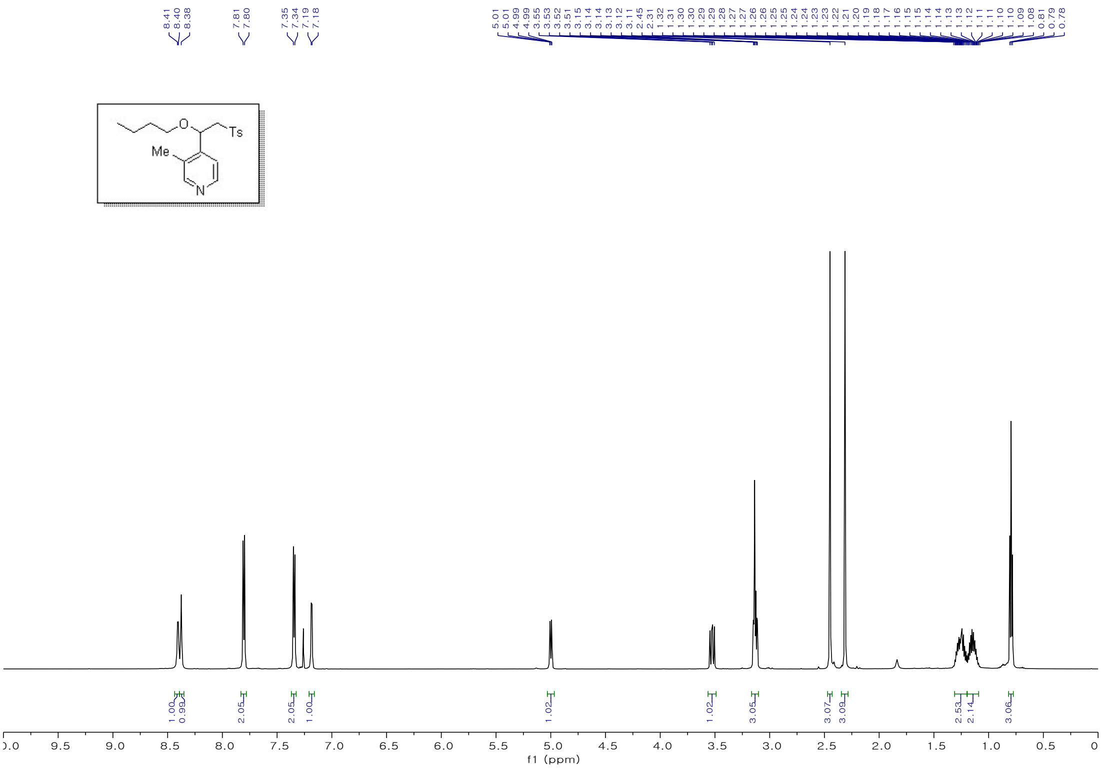

600 MHz, ^{1}H NMR in $CDCl_3$.

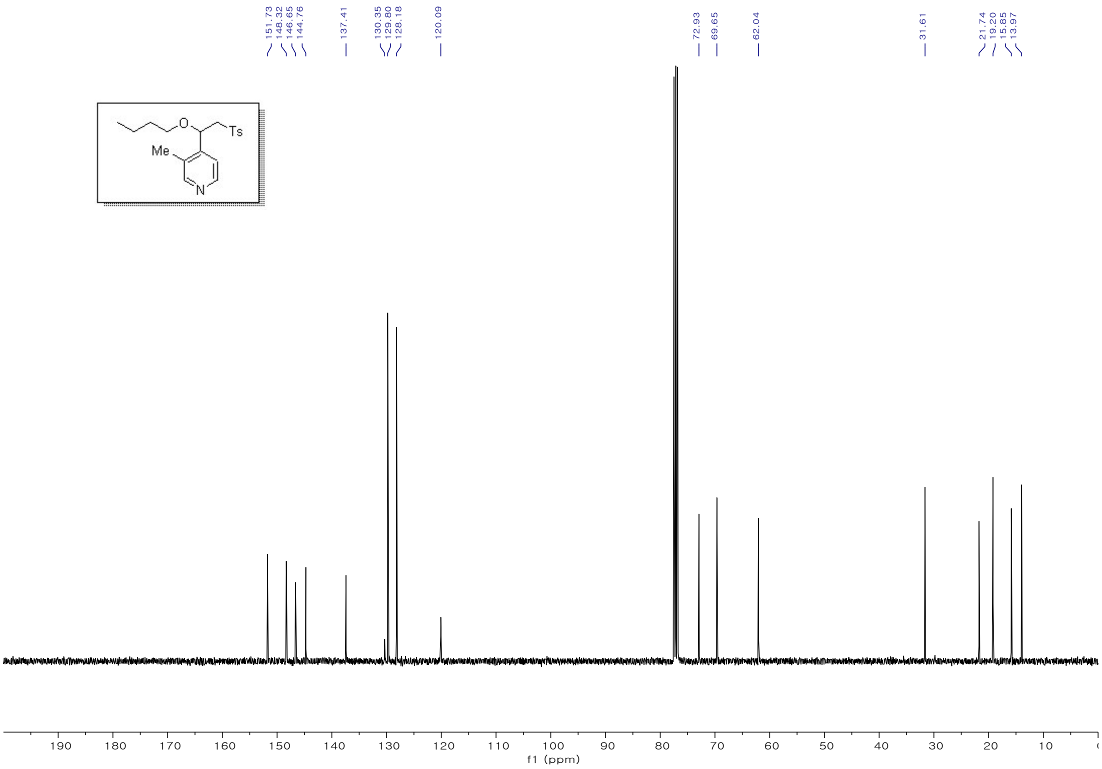

100 MHz, ^{13}C NMR in $CDCl_3$.

4-(1-butoxy-2-tosylethyl)-3-phenylpyridine (4g).

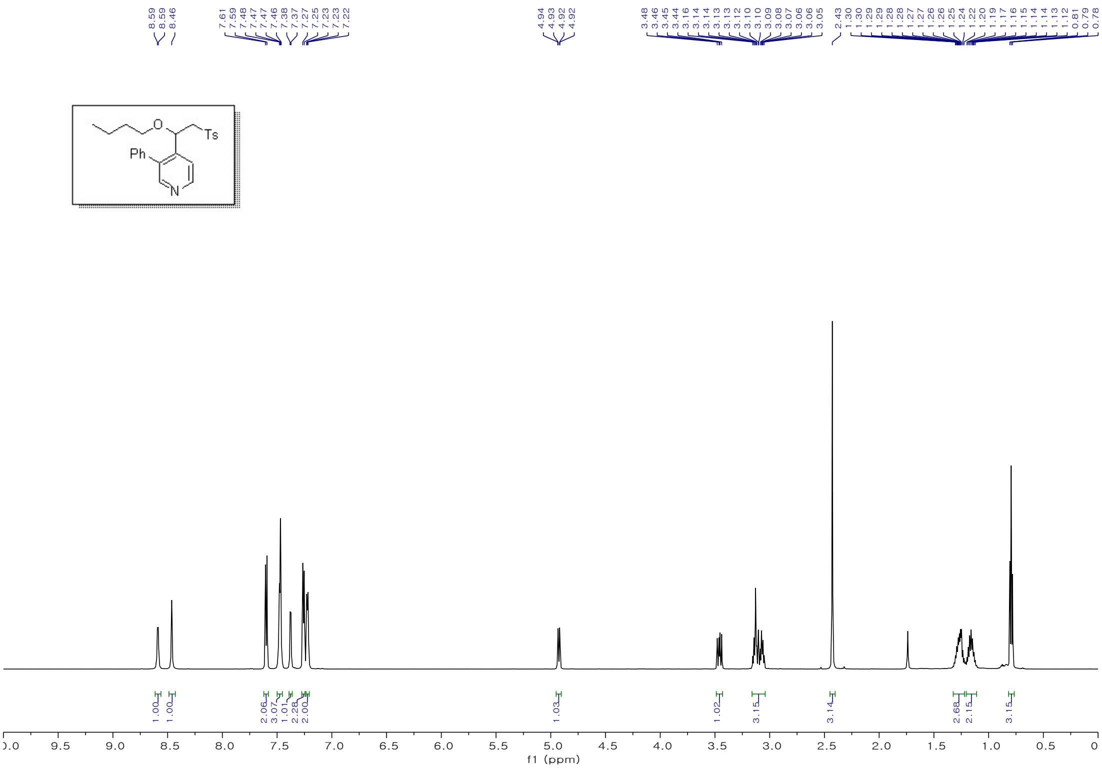

600 MHz, ^{1}H NMR in $CDCl_3$.

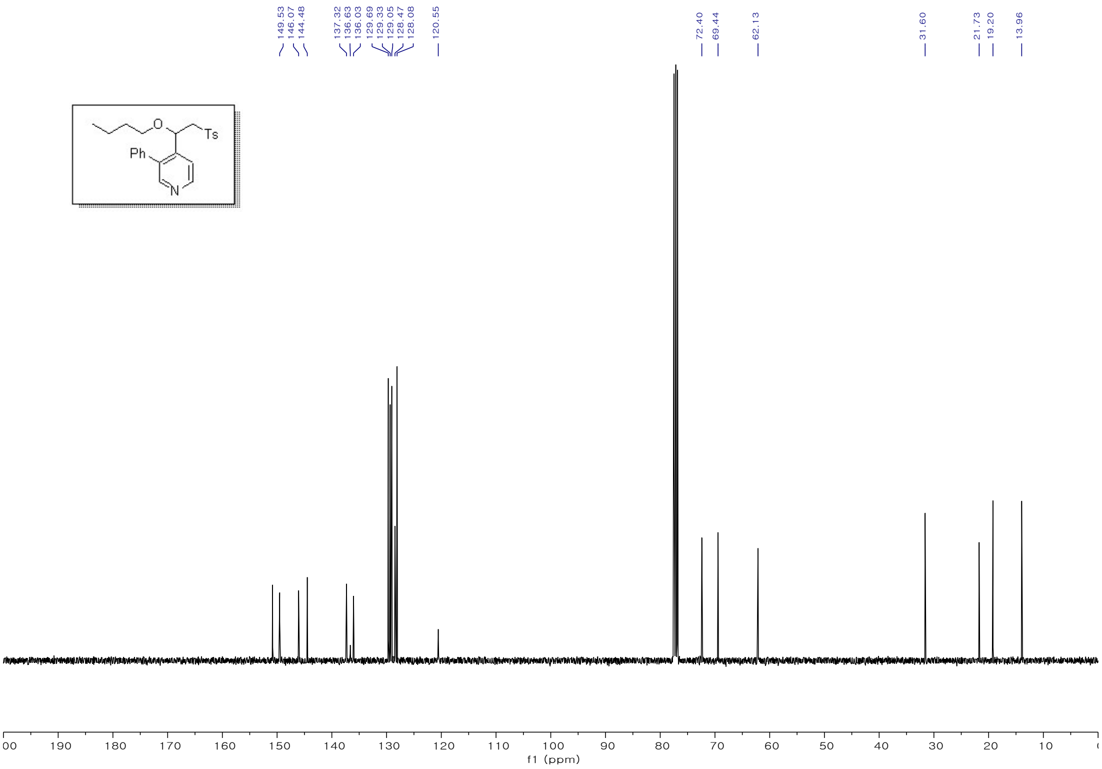

100 MHz, ^{13}C NMR in $CDCl_3$.

4-(1-butoxy-2-tosylethyl)-3-chloropyridine (4h).

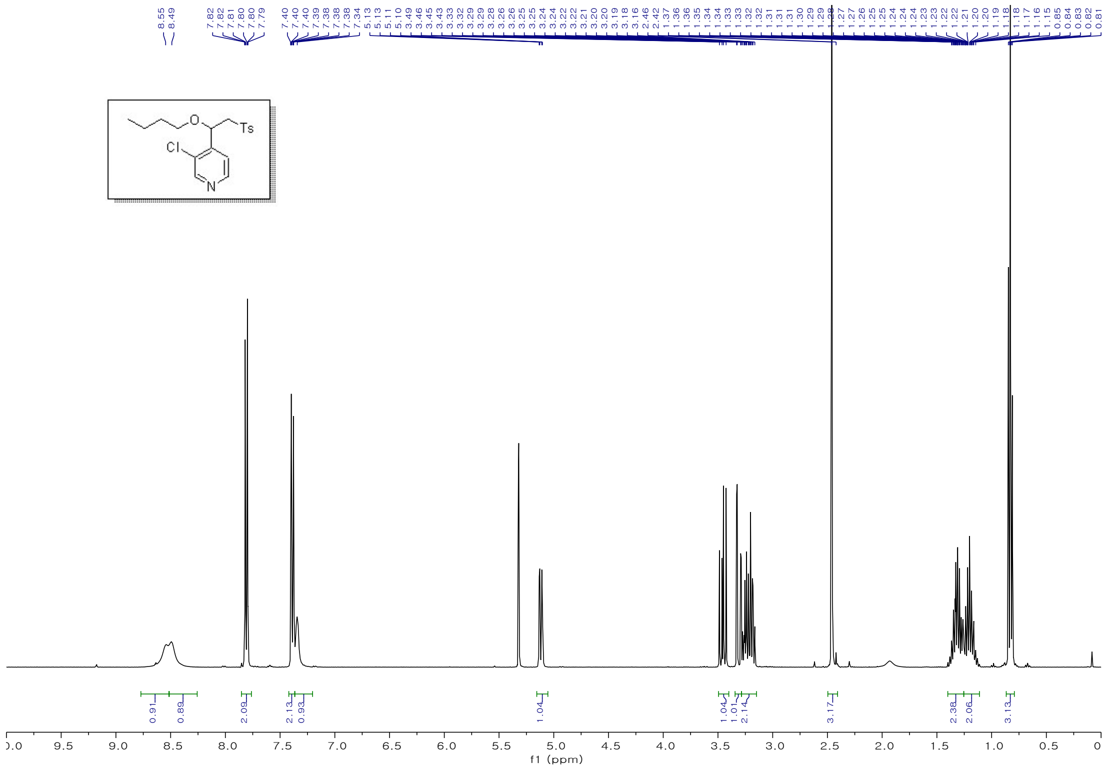

600 MHz, ^{1}H NMR in CD_2Cl_2.

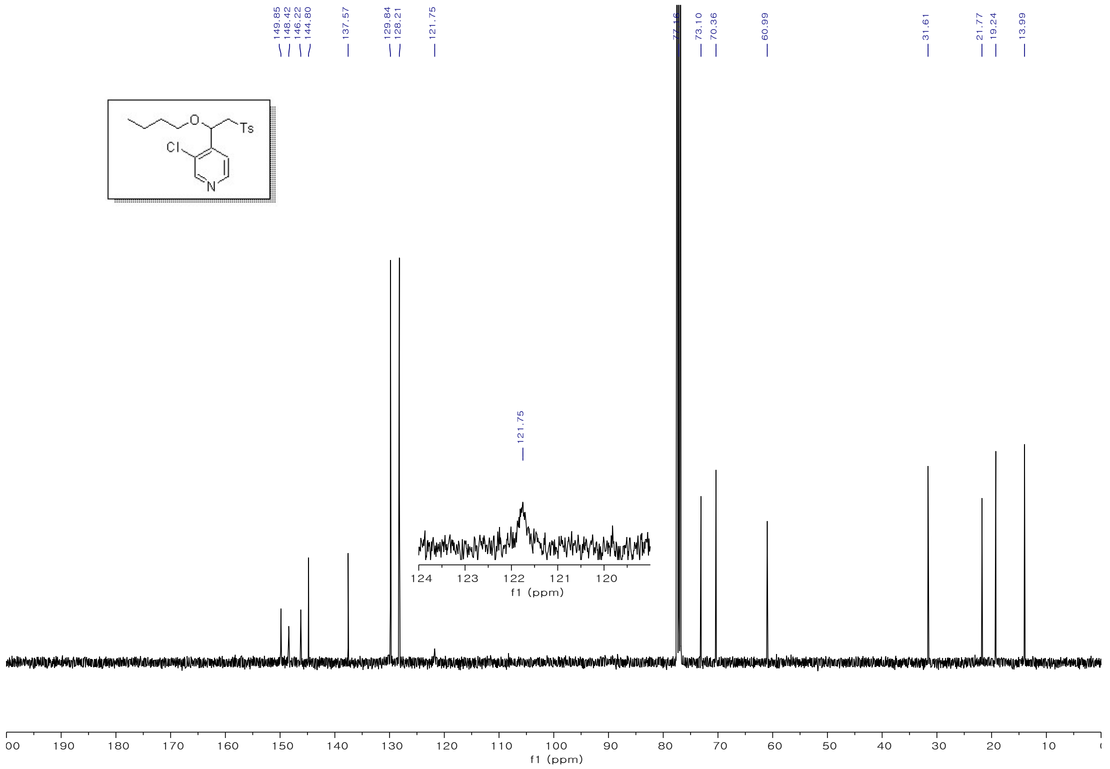

100 MHz, ^{13}C NMR in $CDCl_3$.

3-bromo-4-(1-butoxy-2-tosylethyl)pyridine (4i).

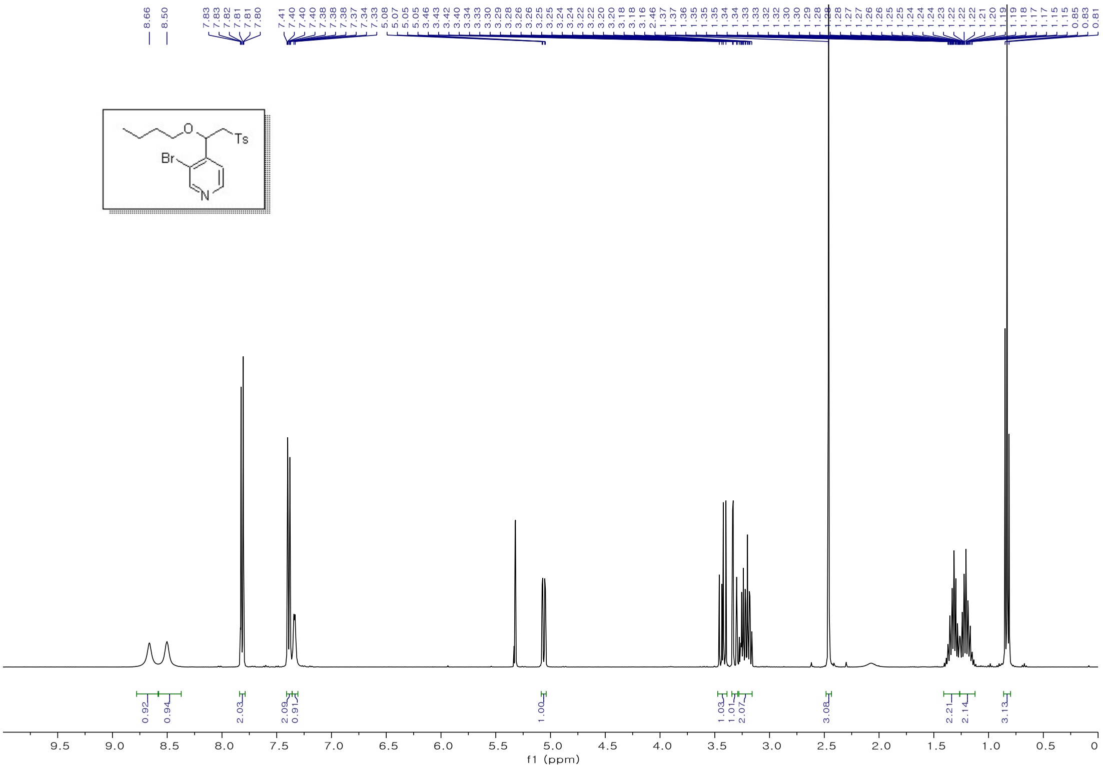

600 MHz, ^{1}H NMR in CD_2Cl_2.

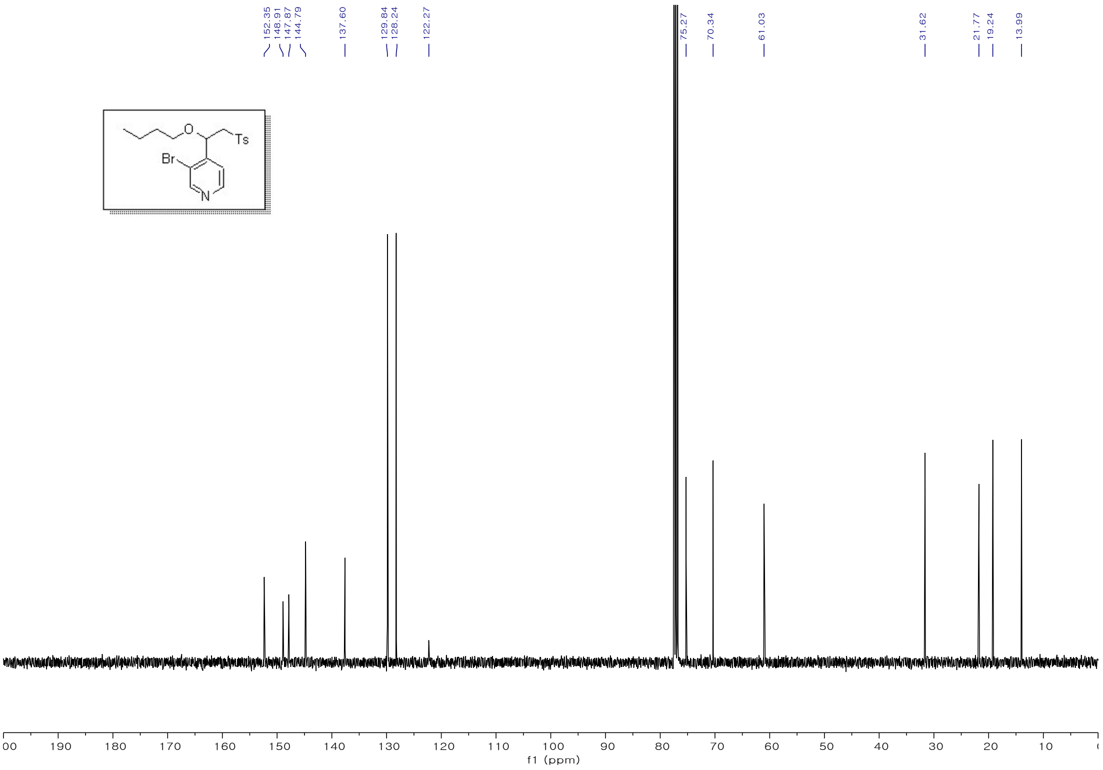

100 MHz, ^{13}C NMR in $CDCl_3$.

methyl 4-(1-butoxy-2-tosylethyl)nicotinate (4j).

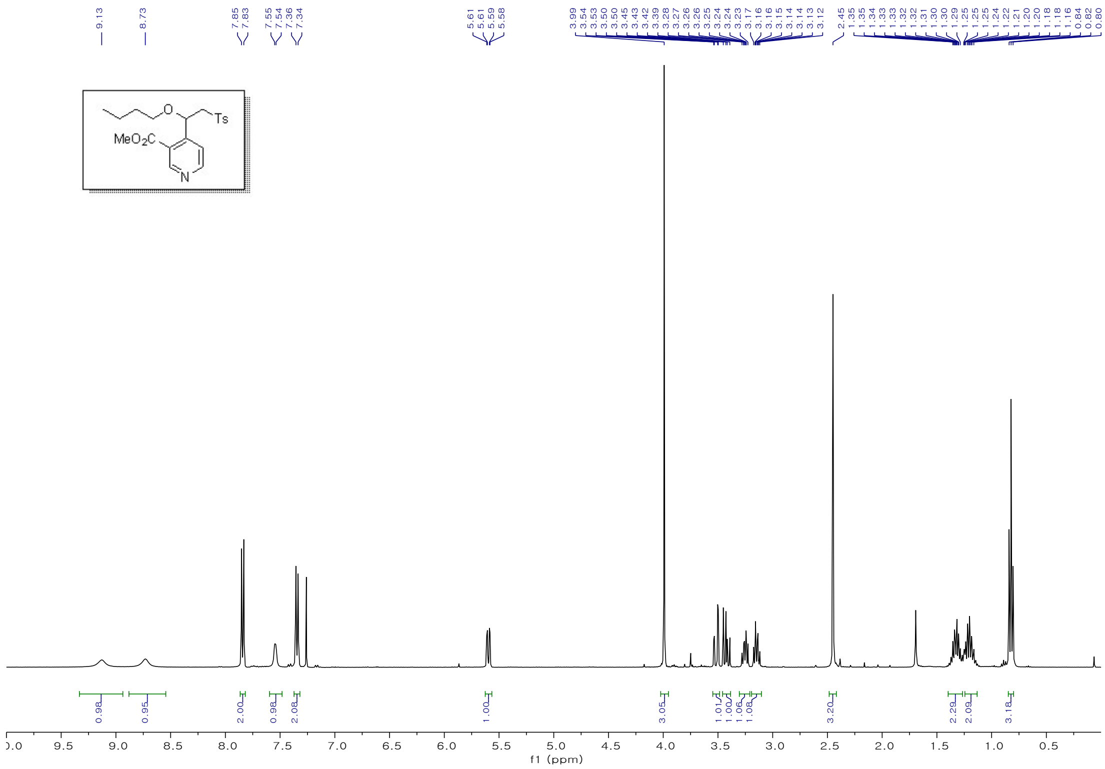

400 MHz, ^{1}H NMR in $CDCl_3$.

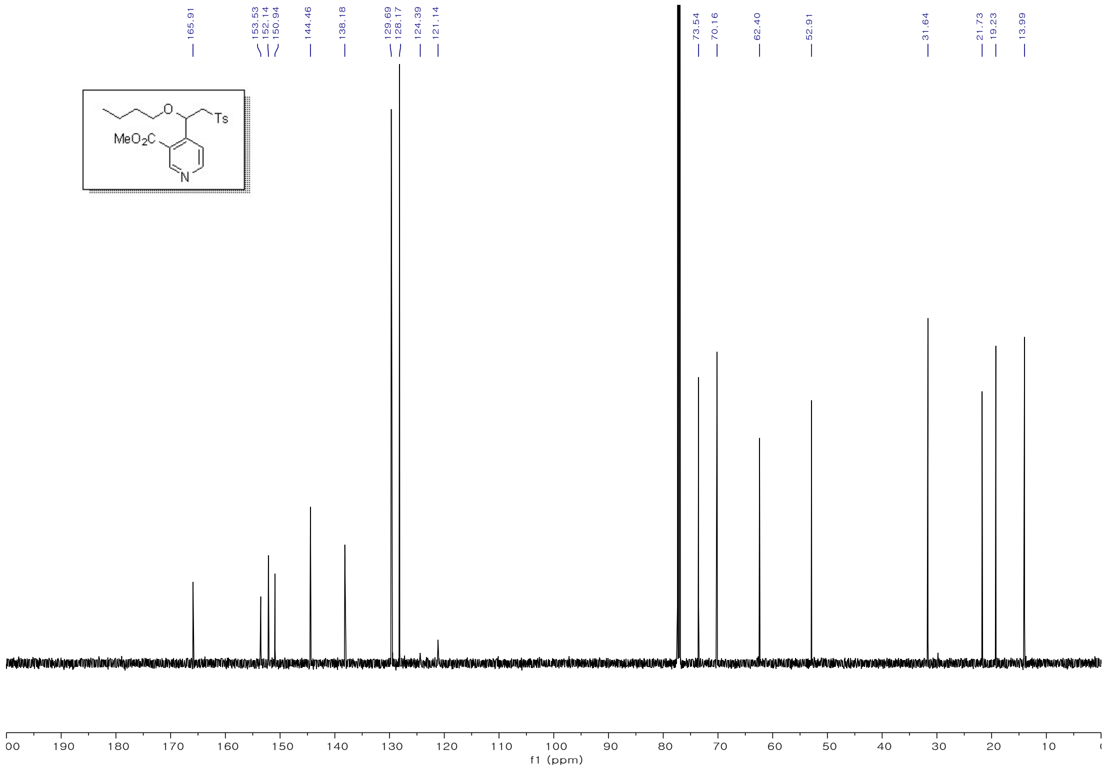

100 MHz, ^{13}C NMR in $CDCl_3$.

4-(1-butoxy-2-tosylethyl)-6,7-dihydro-5H-cyclopenta[b]pyridine (4k).

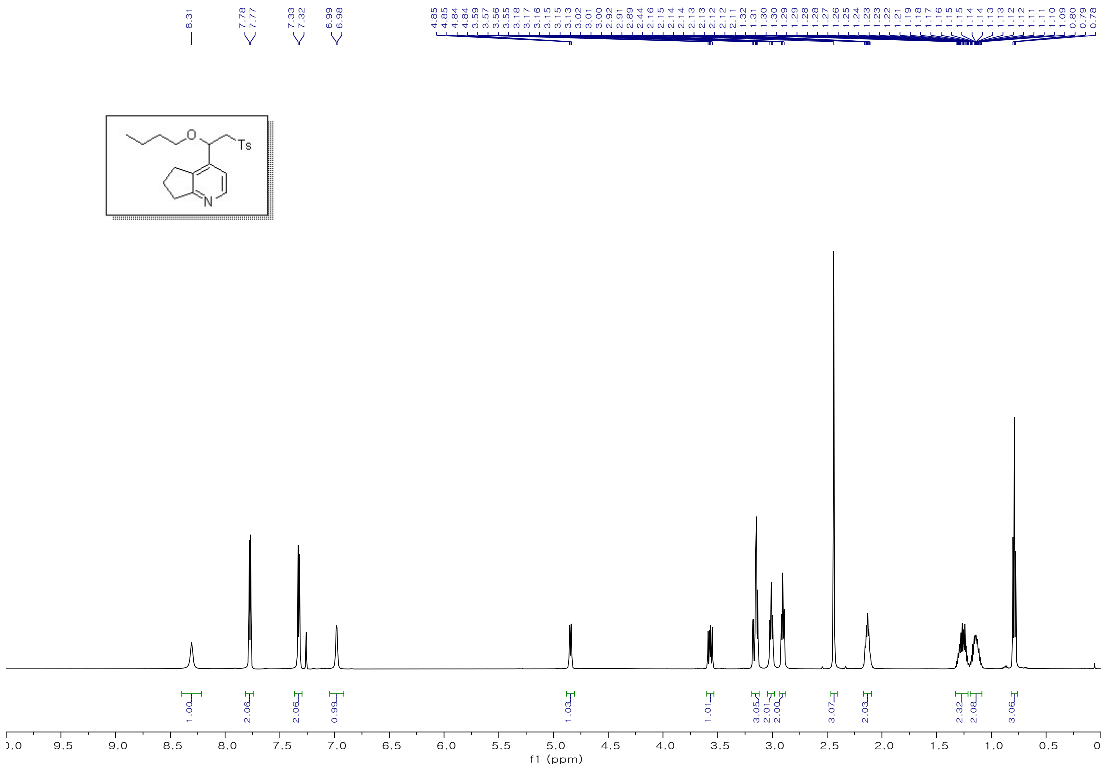

600 MHz, ^{1}H NMR in $CDCl_3$.

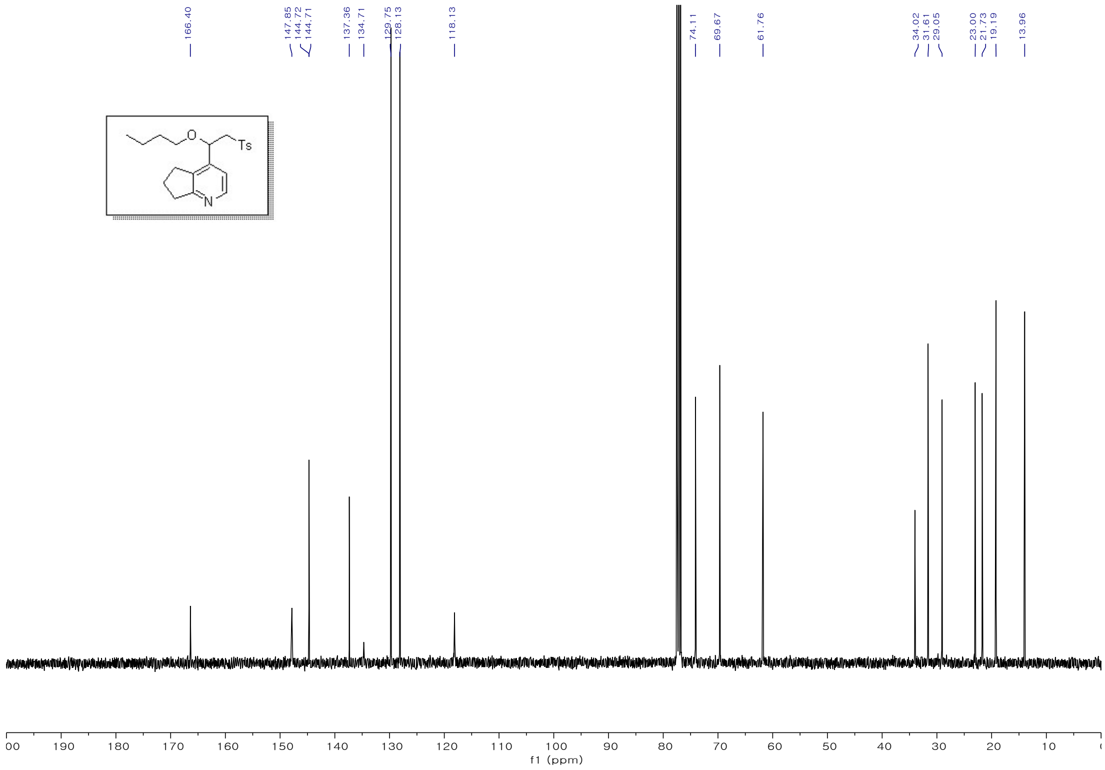

100 MHz, ^{13}C NMR in $CDCl_3$.

4-(1-butoxy-2-(phenylsulfonyl)ethyl)pyridine (4l).

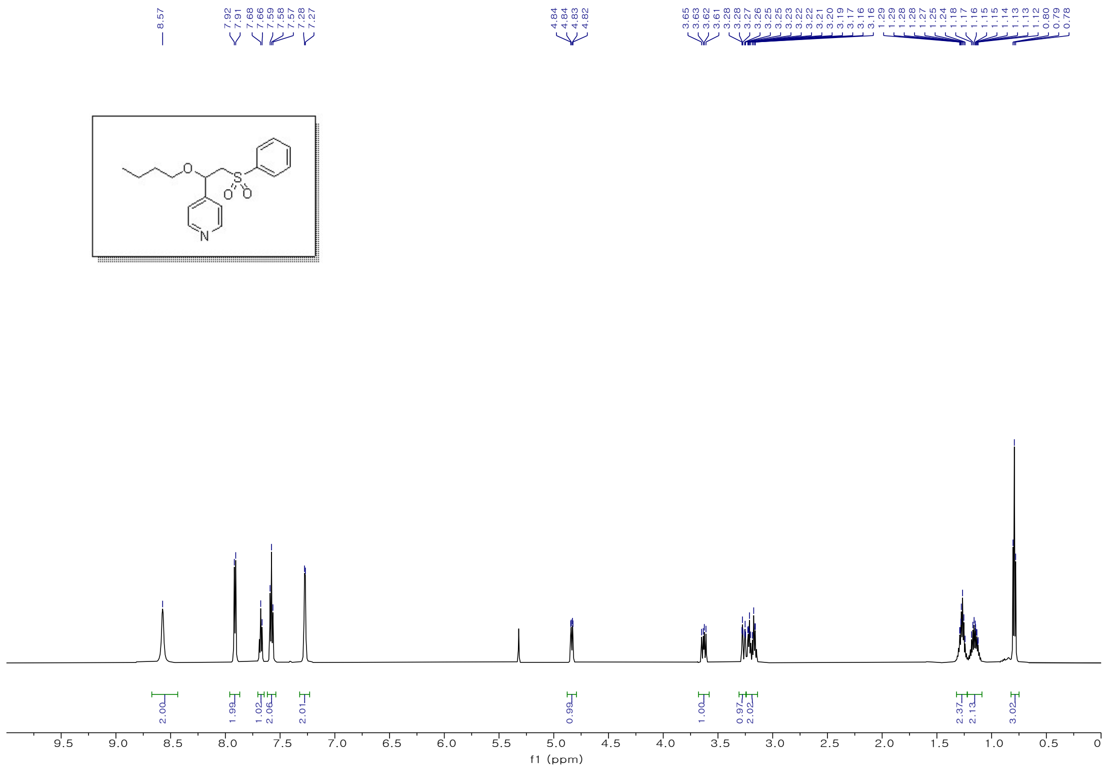

600 MHz, ^{1}H NMR in CD_2Cl_2.

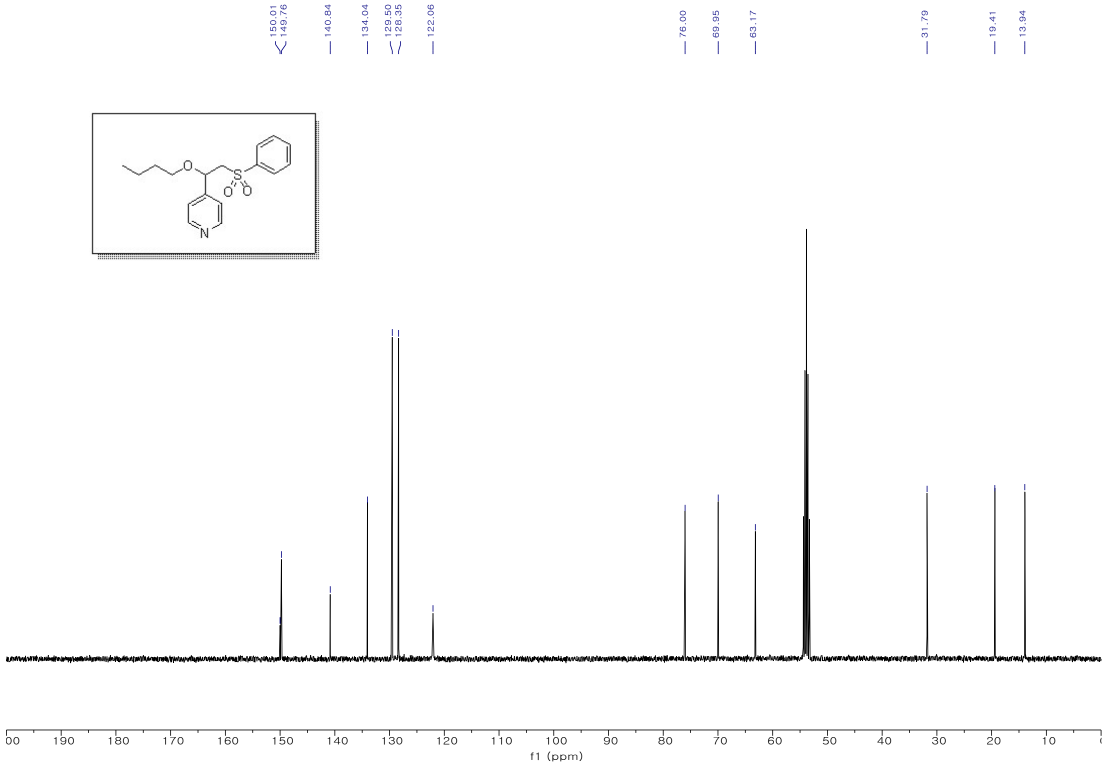

100 MHz, ^{13}C NMR in CD_2Cl_2.

4-(1-butoxy-2-((4-chlorophenyl)sulfonyl)ethyl)pyridine (4m).

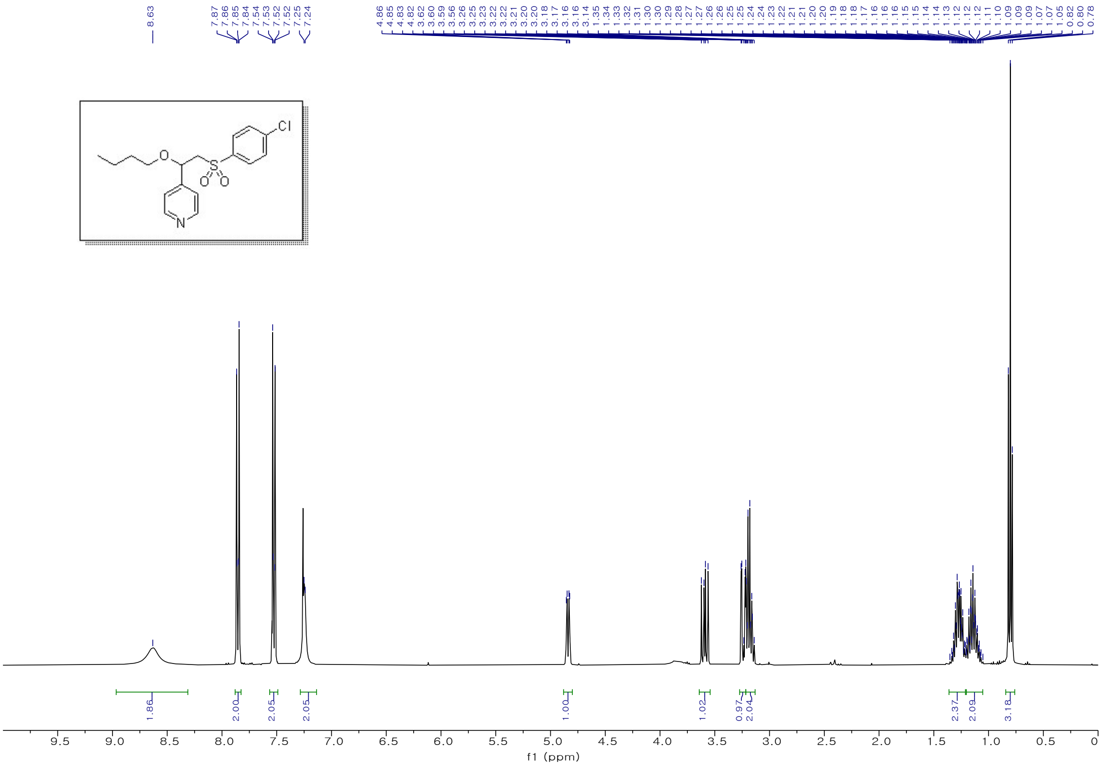

400 MHz, ^{1}H NMR in $CDCl_3$

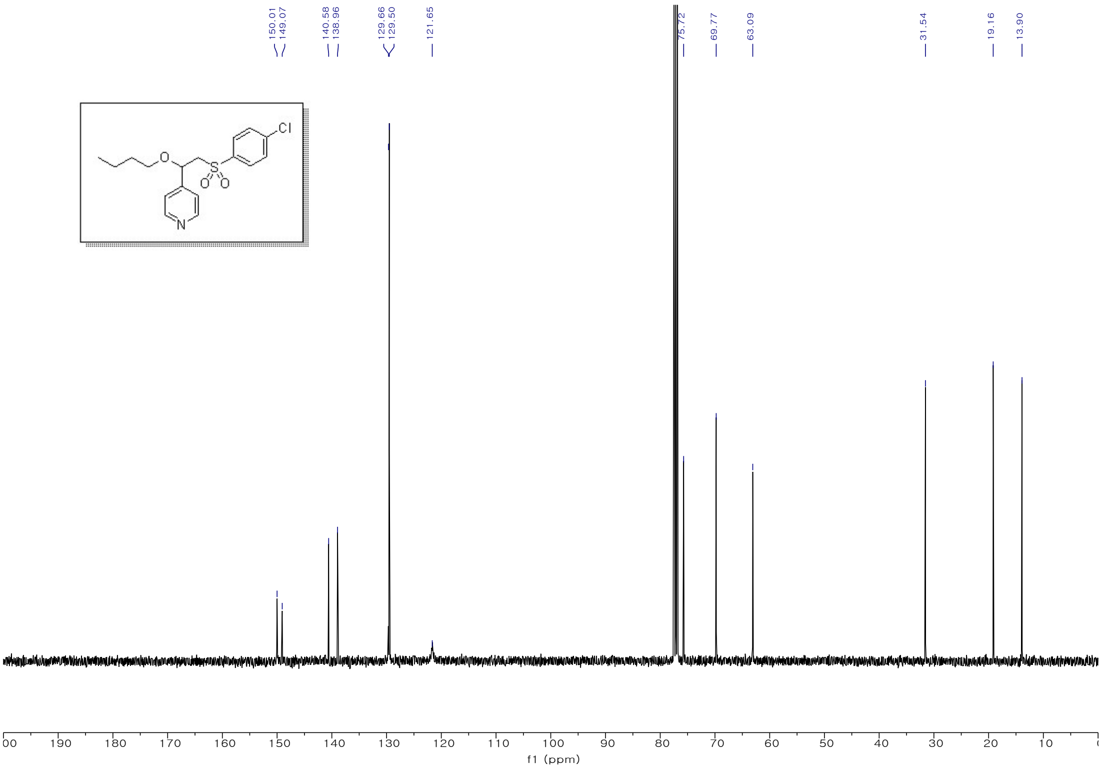

100 MHz, ^{13}C NMR in $CDCl_3$

4-(1-butoxy-2-((4-methoxyphenyl)sulfonyl)ethyl)pyridine (4n).

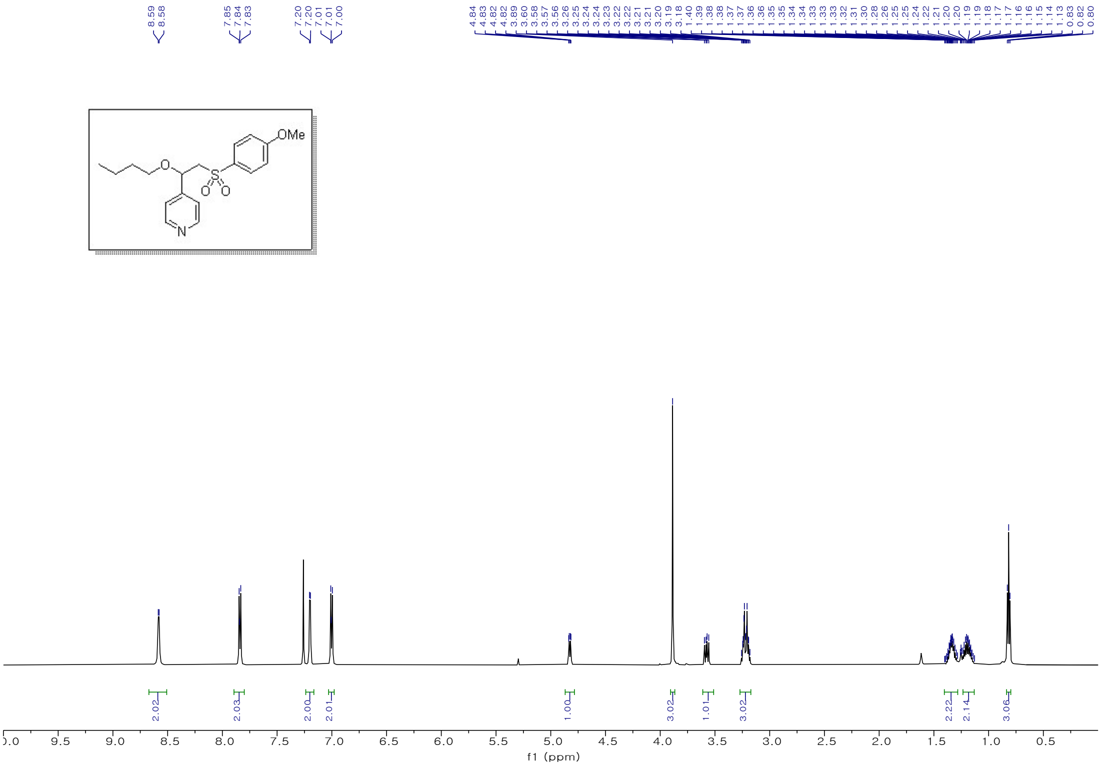

600 MHz, ^{1}H NMR in $CDCl_3$

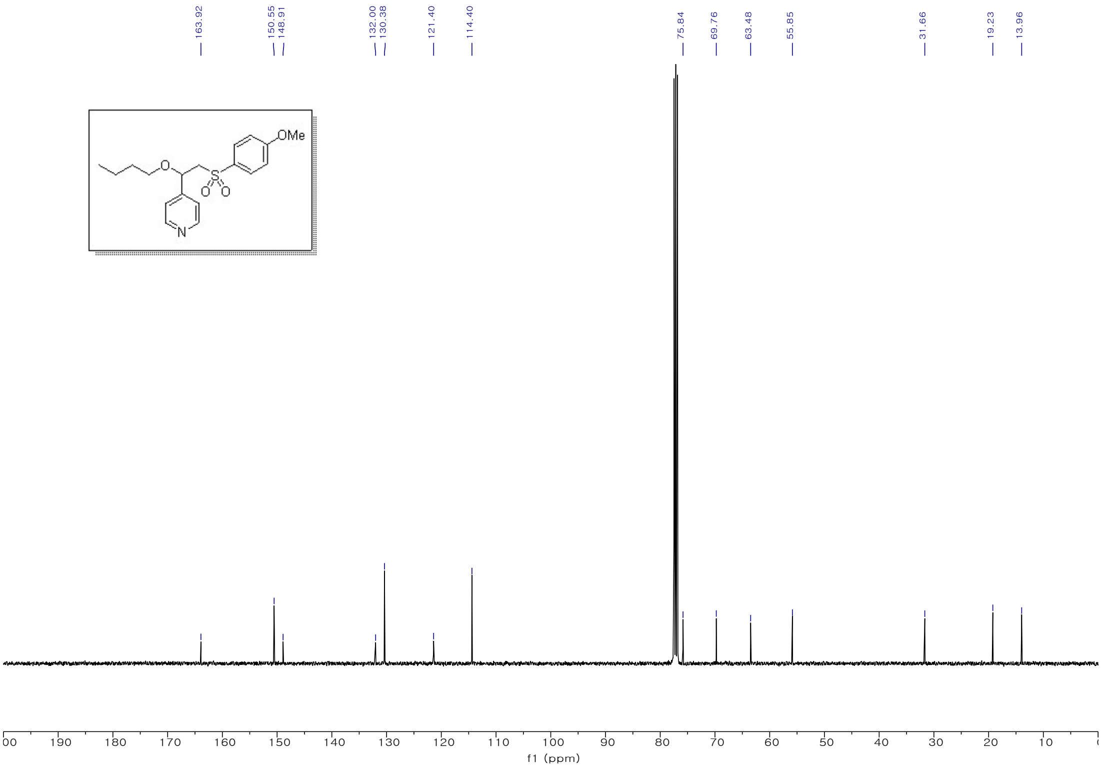

100 MHz, ^{13}C NMR in $CDCl_3$.

4-(1-butoxy-2-((4-(trifluoromethyl)phenyl)sulfonyl)ethyl)pyridine (4o).

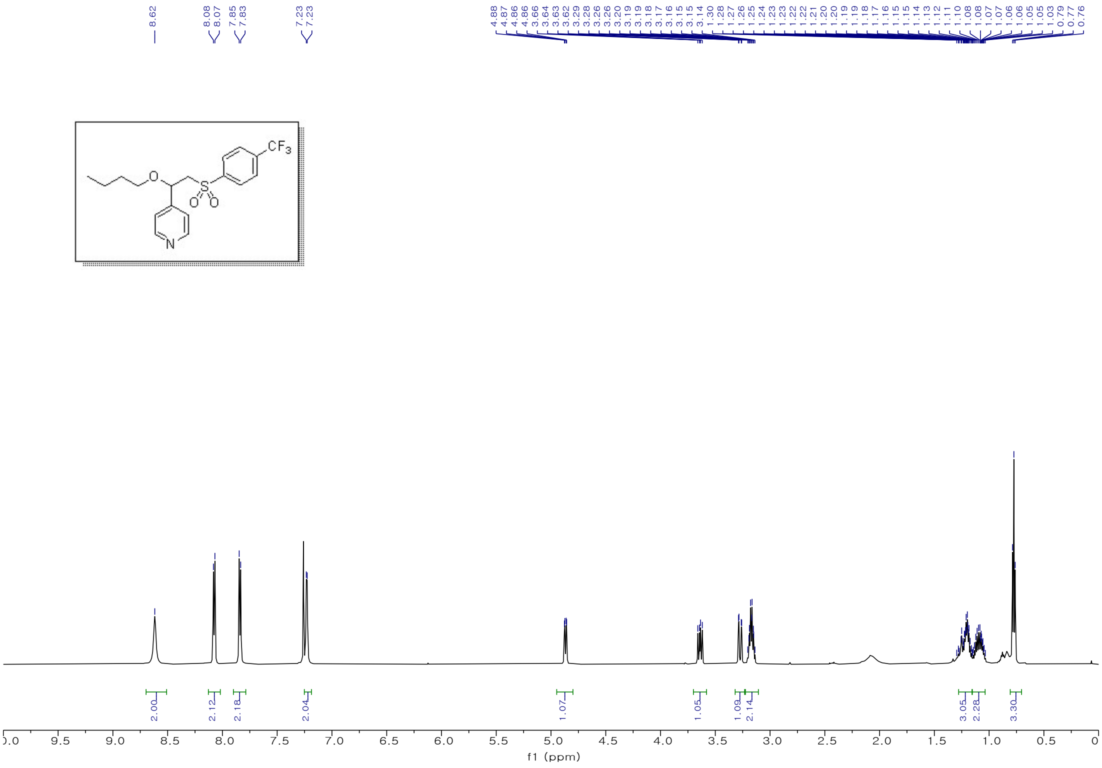

600 MHz, ^{1}H NMR in $CDCl_3$

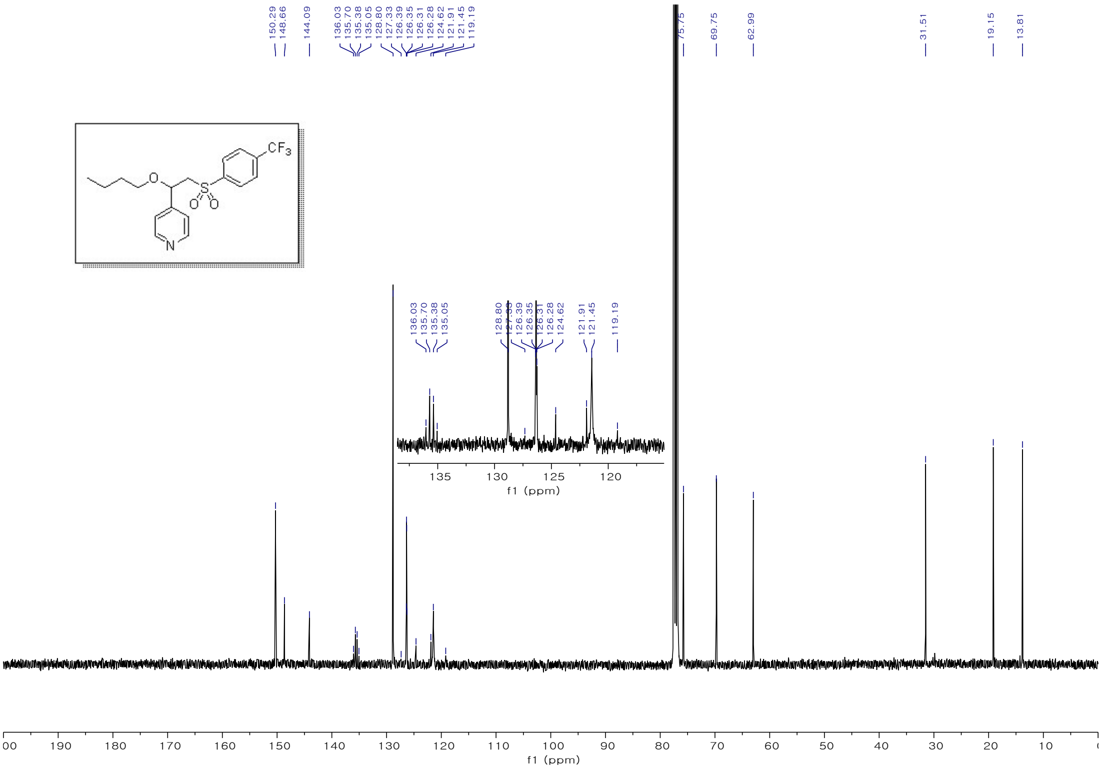

100 MHz, ^{13}C NMR in $CDCl_3$

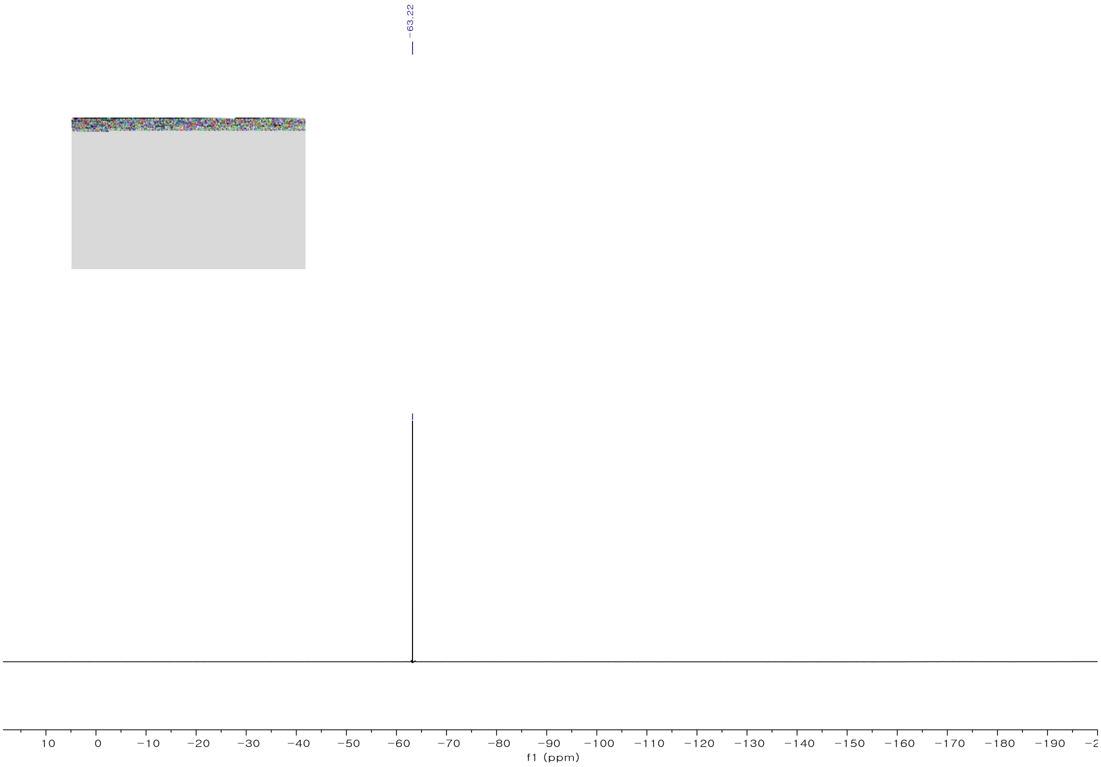

376 MHz, ^{19}F NMR in $CDCl_3$

-(1-butoxy-2-(naphthalen-2-ylsulfonyl)ethyl)pyridine (4p).

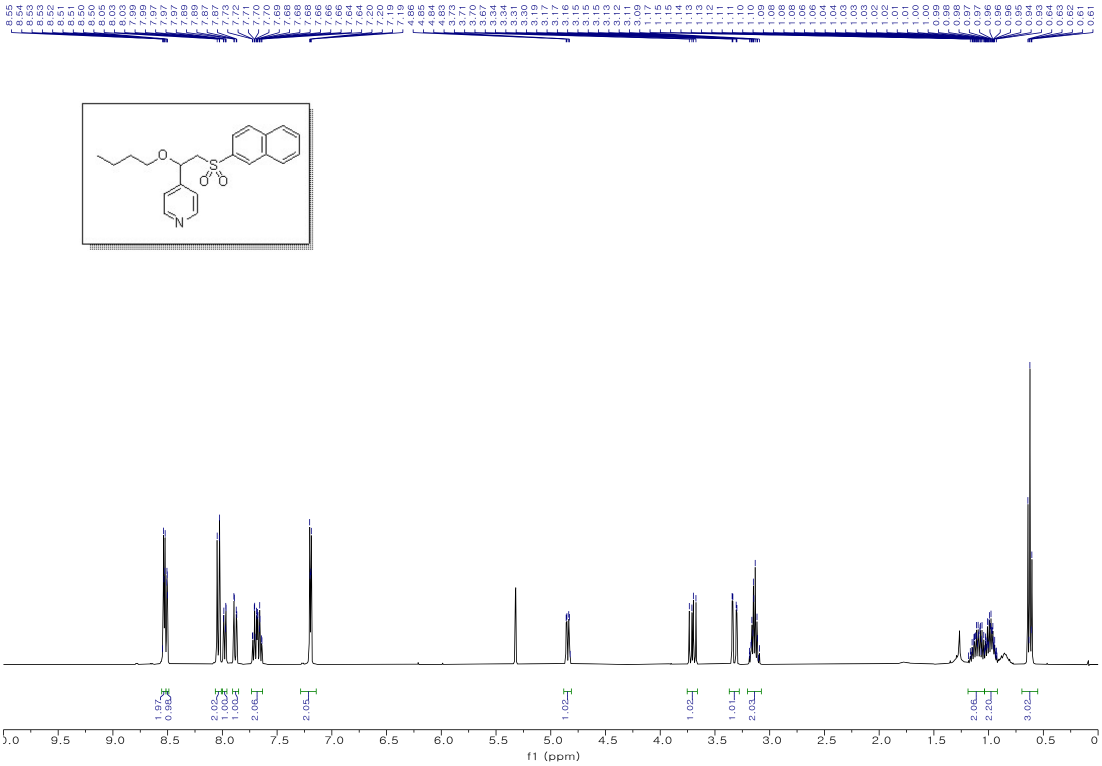

400 MHz, ^{1}H NMR in CD_2Cl_2.

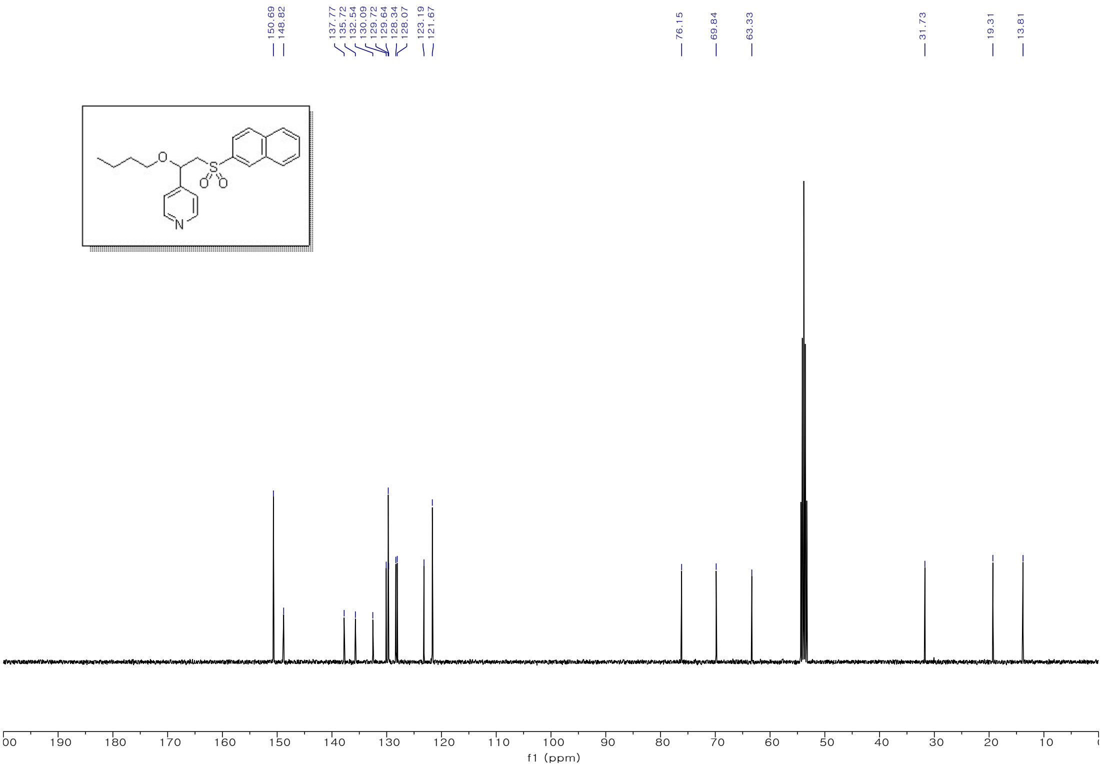

100 MHz, ^{13}C NMR in CD_2Cl_2.

2-((2-butoxy-2-(pyridin-4-yl)ethyl)sulfonyl)pyrimidine (4q).

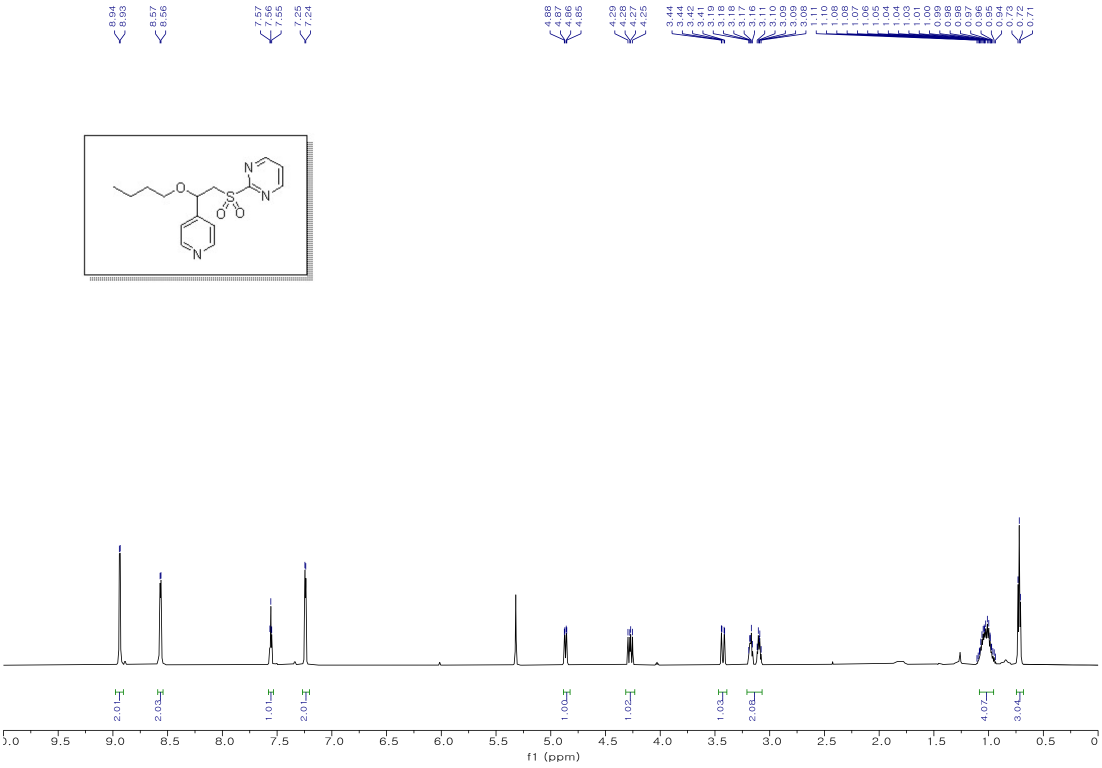

600 MHz, ^{1}H NMR in CD_2Cl_2.

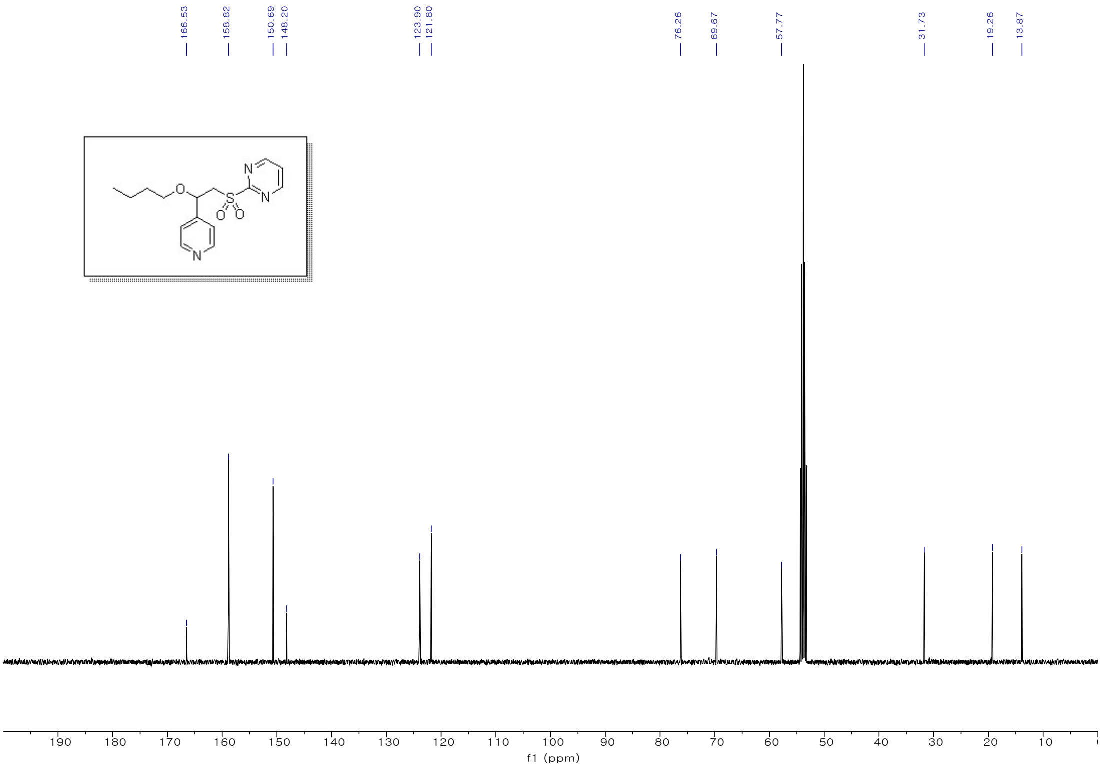

100 MHz, ^{13}C NMR in CD_2Cl_2.

4-(1-butoxy-2-(methylsulfonyl)ethyl)pyridine (4r).

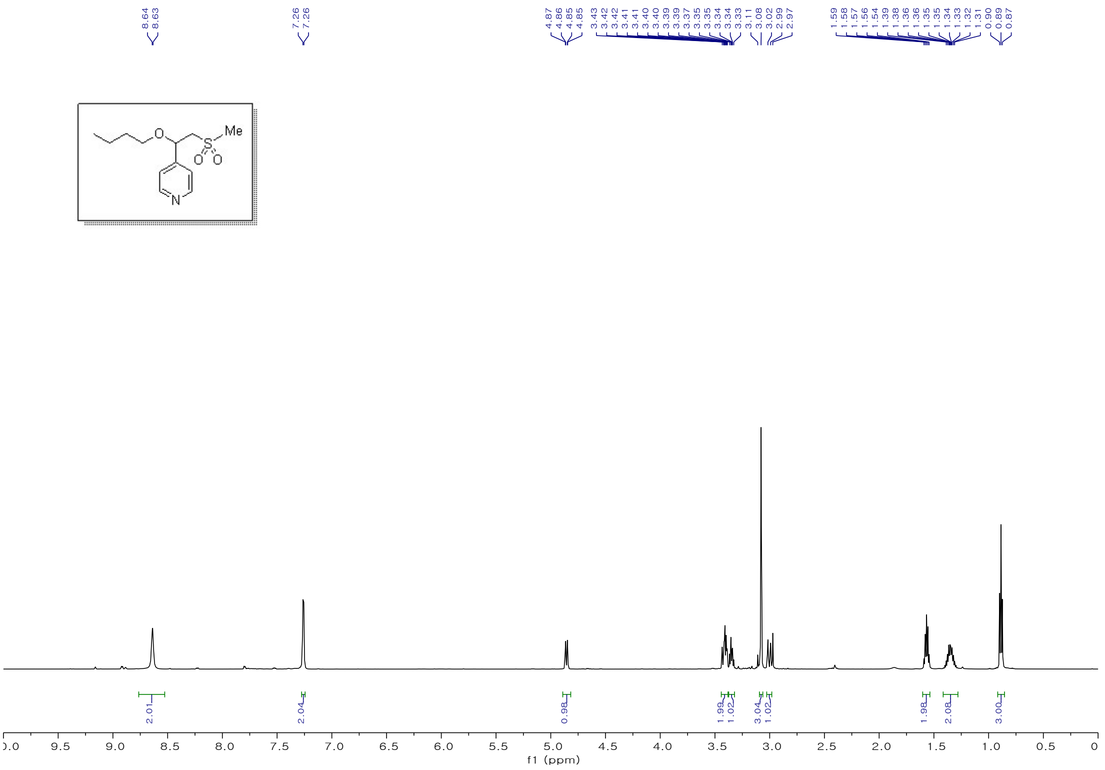

400 MHz, ^{1}H NMR in $CDCl_3$.

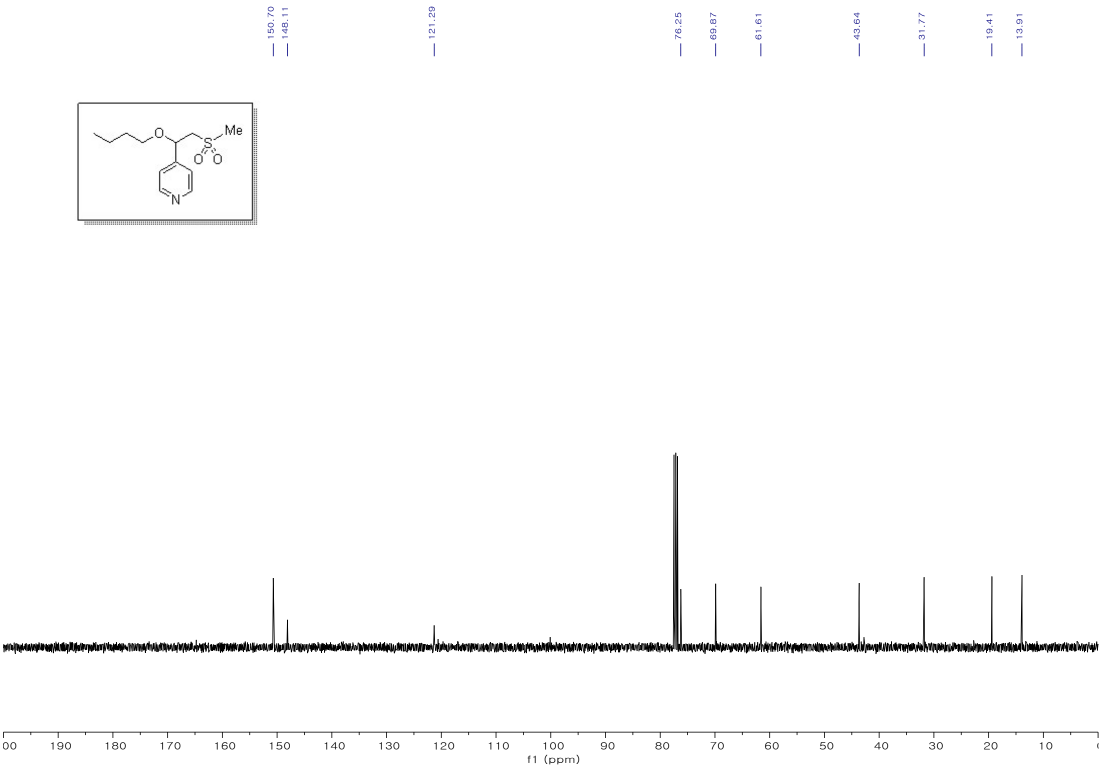

100 MHz, ^{13}C NMR in $CDCl_3$.

4-(1-butoxy-2-(ethylsulfonyl)ethyl)pyridine (4s).

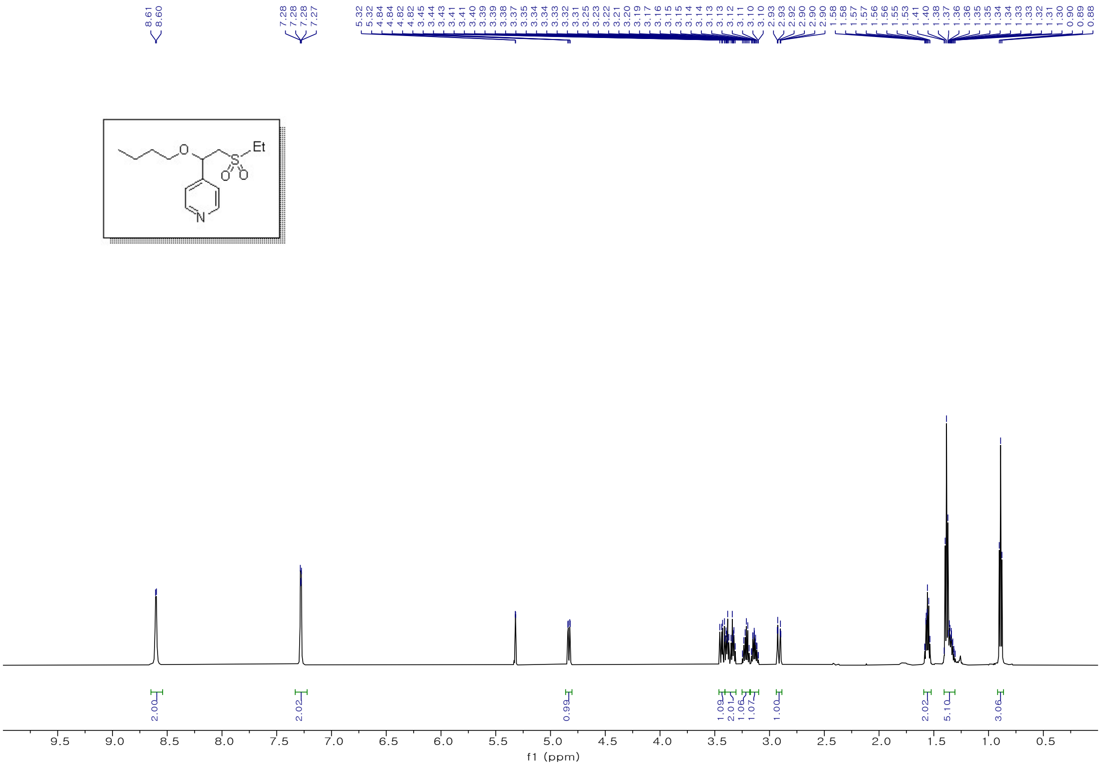

600 MHz, ^{1}H NMR in CD_2Cl_2

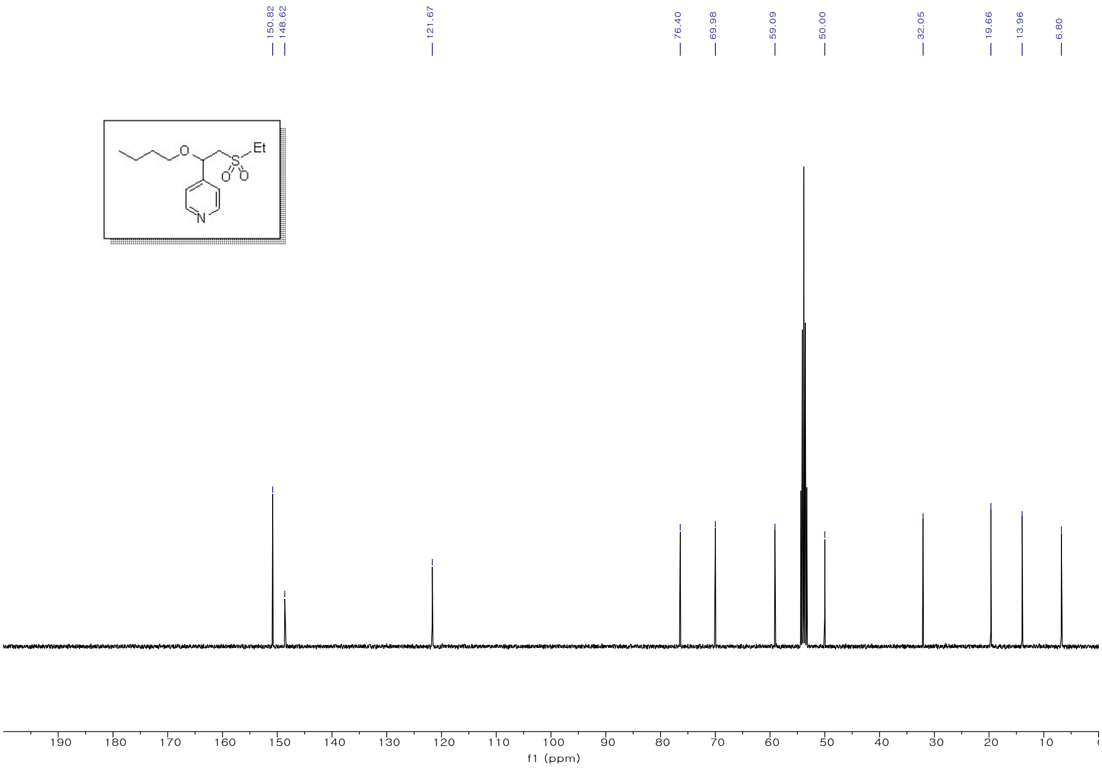

100 MHz, ^{13}C NMR in CD_2Cl_2

4-(1-butoxy-2-(phenethylsulfonyl)ethyl)pyridine (4t).

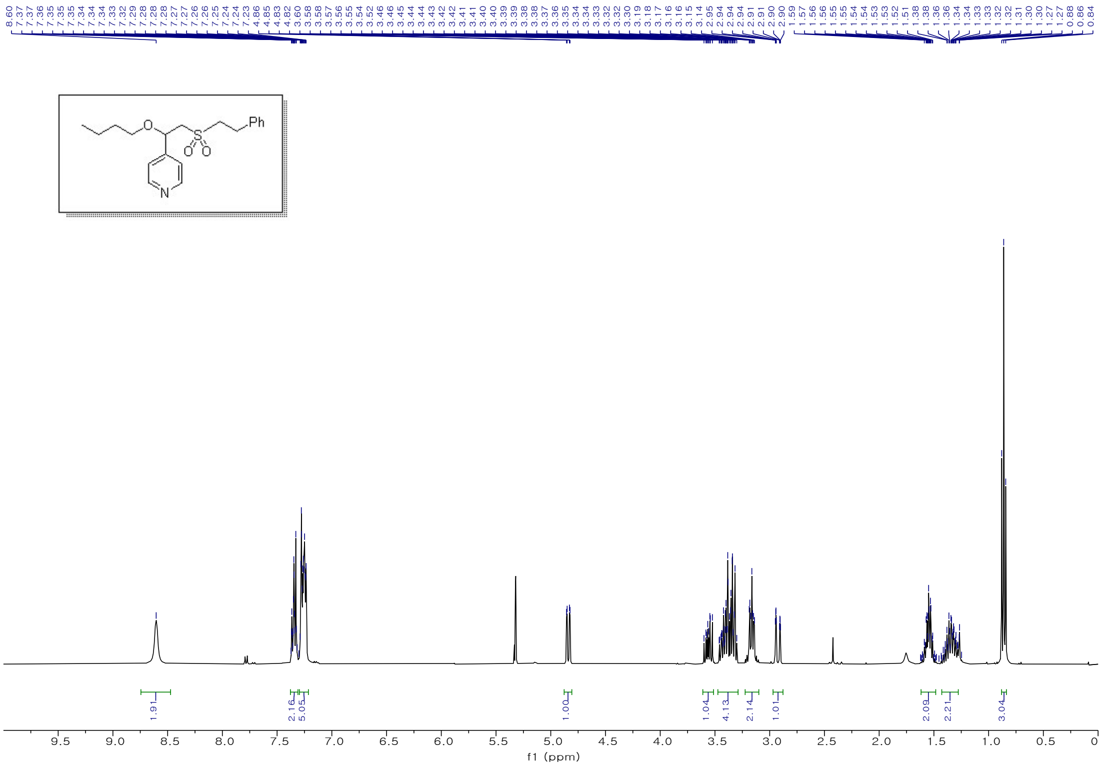

400 MHz, ^1H NMR in CD_2Cl_2

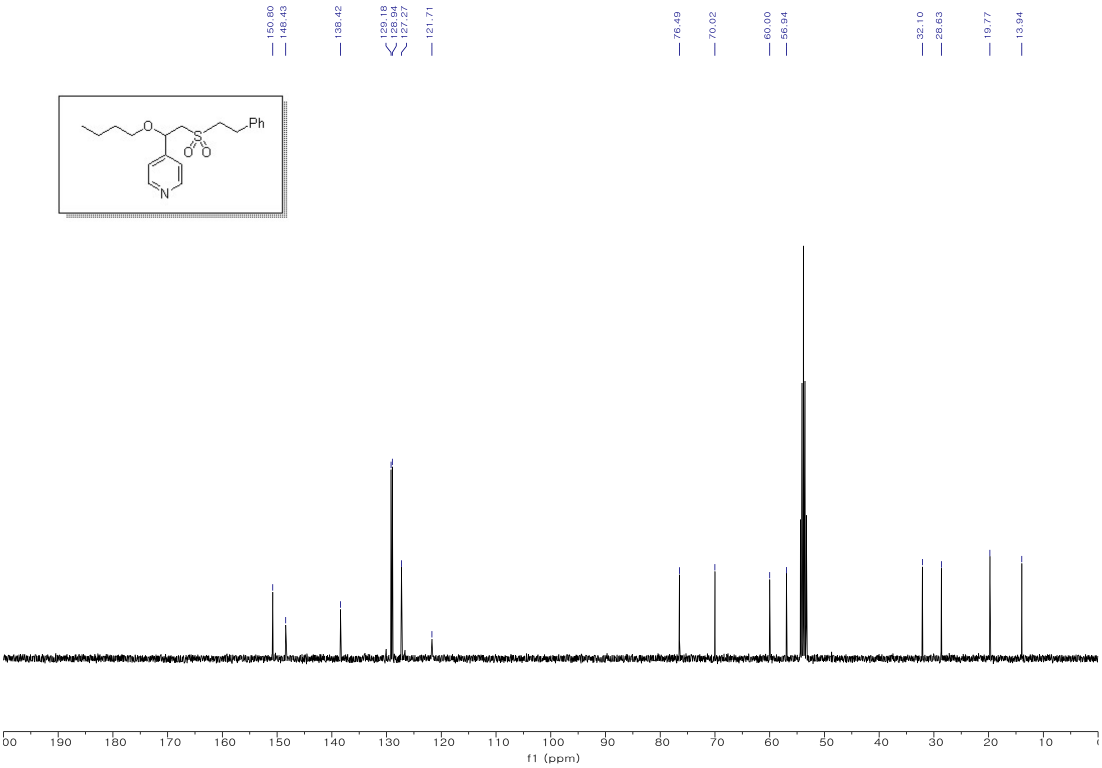

100 MHz, ^{13}C NMR in CD_2Cl_2

4-(1-butoxy-2-(cyclopropylsulfonyl)ethyl)pyridine (4u).

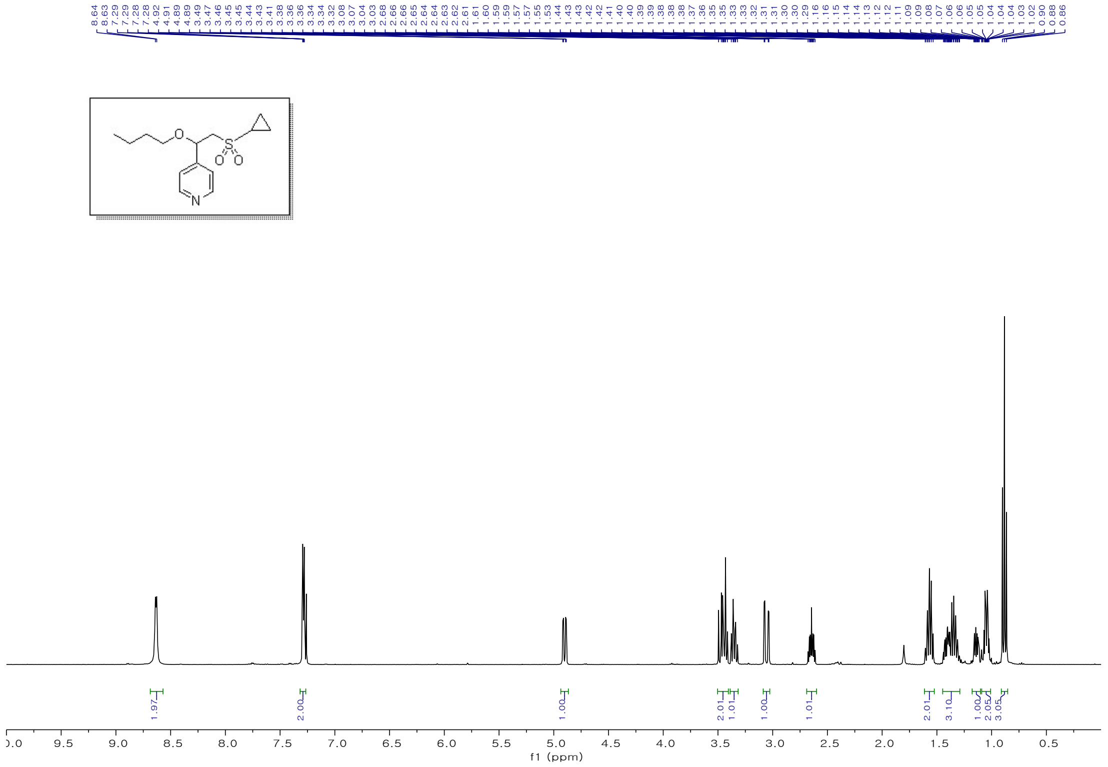

400 MHz, ^{1}H NMR in $CDCl_3$.

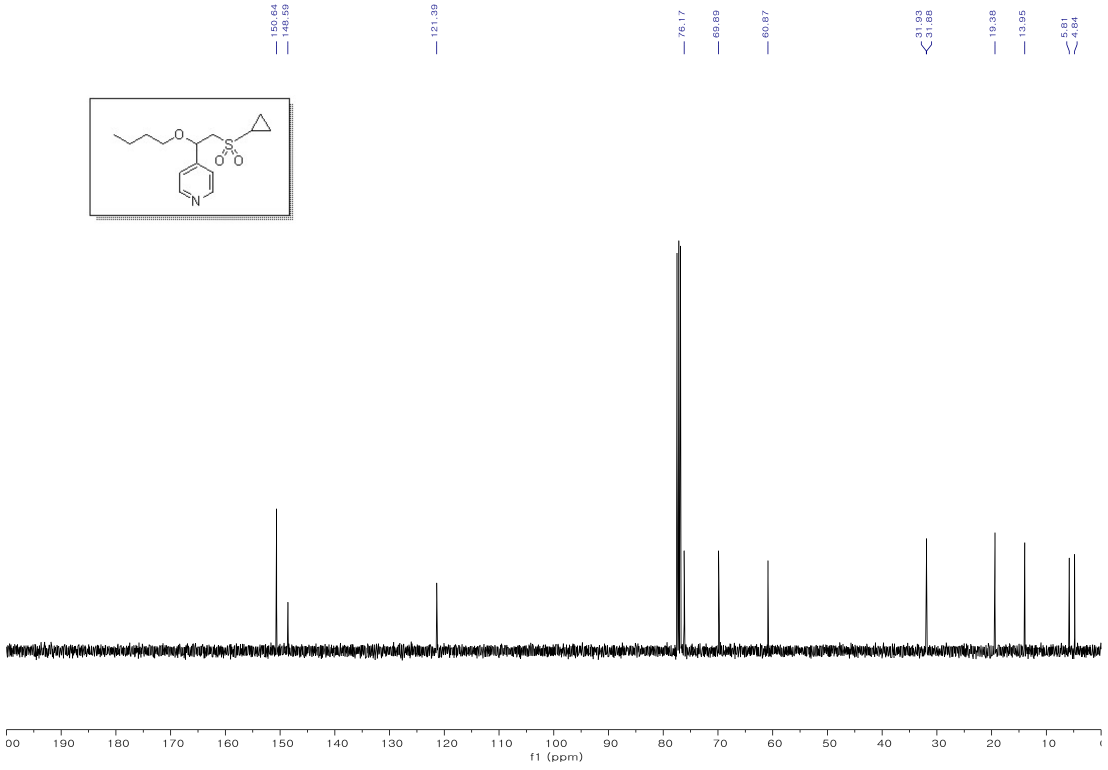

100 MHz, ^{13}C NMR in $CDCl_3$.

***tert*-butyl 3-((2-butoxy-2-(pyridin-4-yl)ethyl)sulfonyl)azetidine-1-carboxylate (4v).**

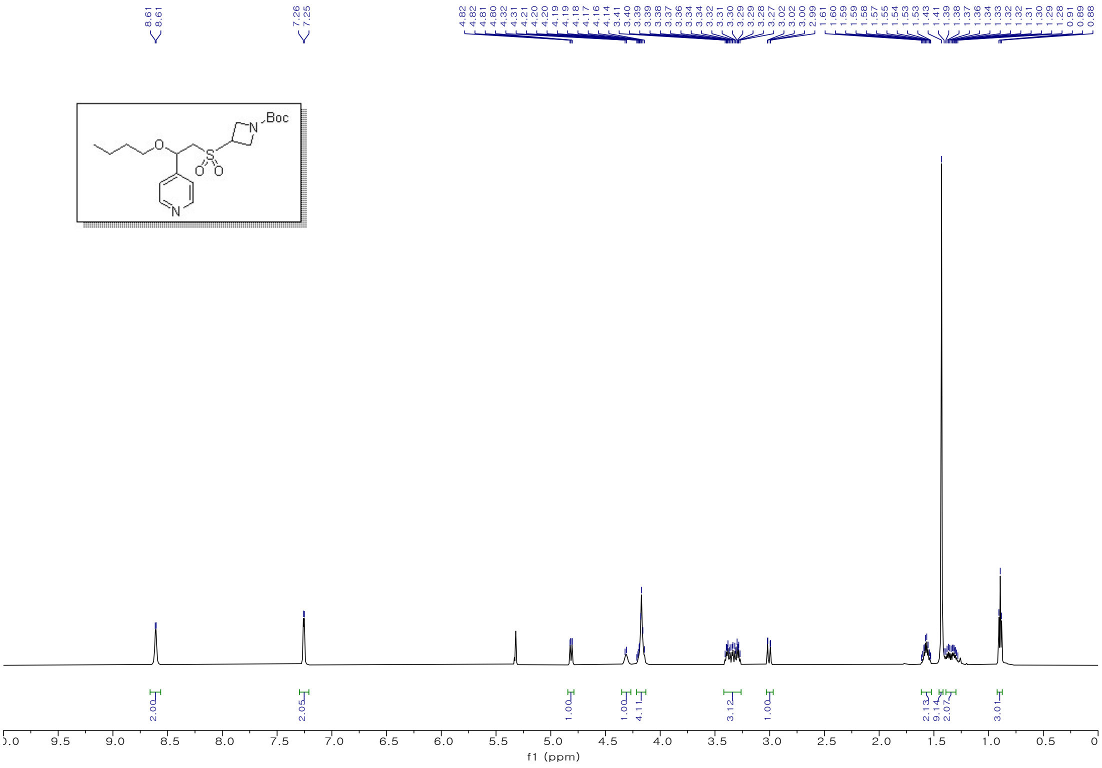

600 MHz, ^{1}H NMR in CD_2Cl_2

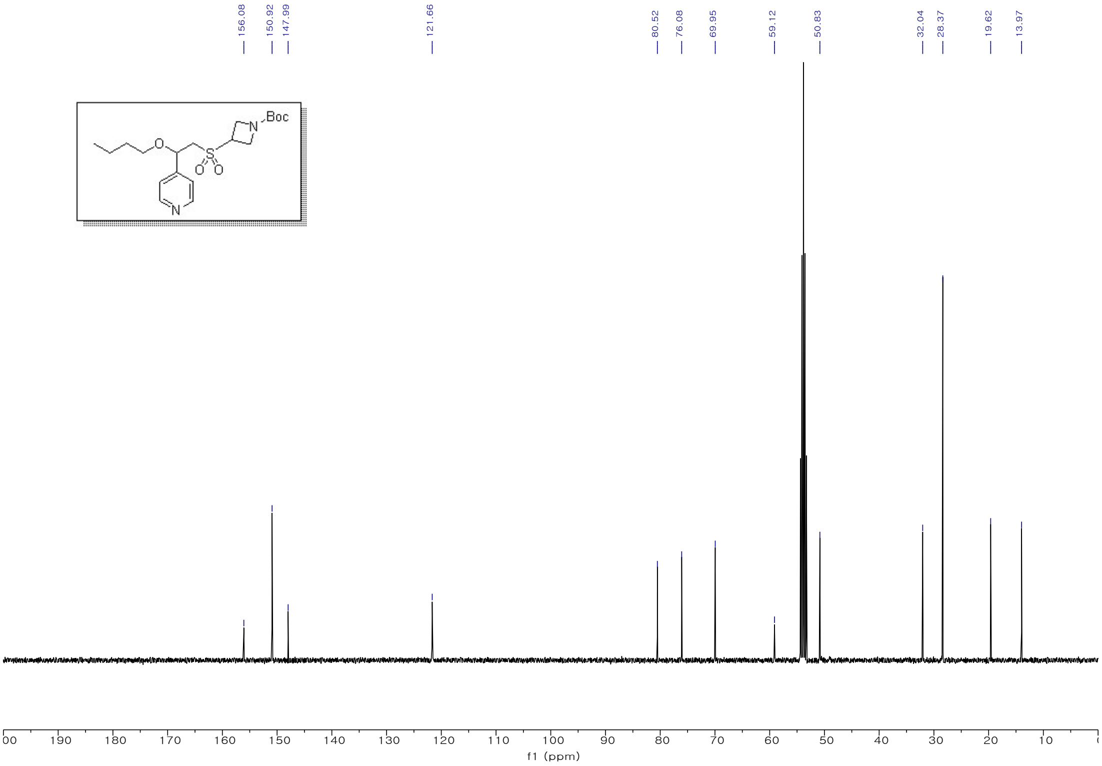

100 MHz, ^{13}C NMR in CD_2Cl_2

4-(1-ethoxy-2-tosylethyl)pyridine (4w).

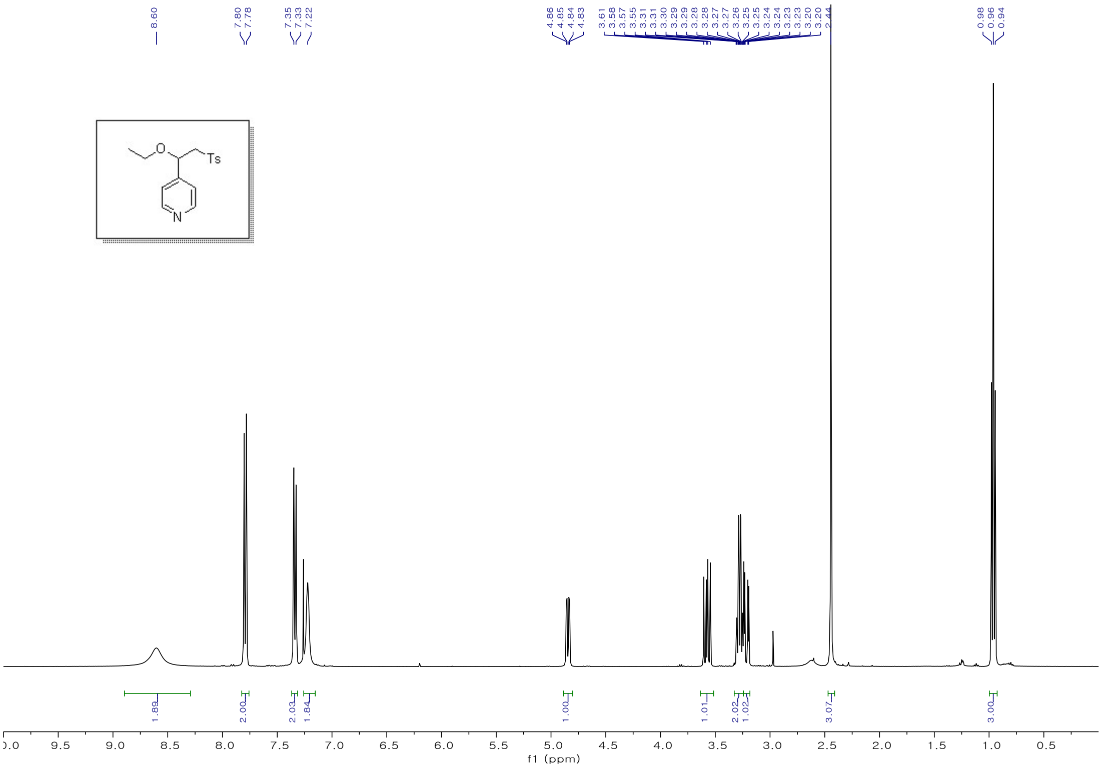

400 MHz, ^{1}H NMR in $CDCl_3$.

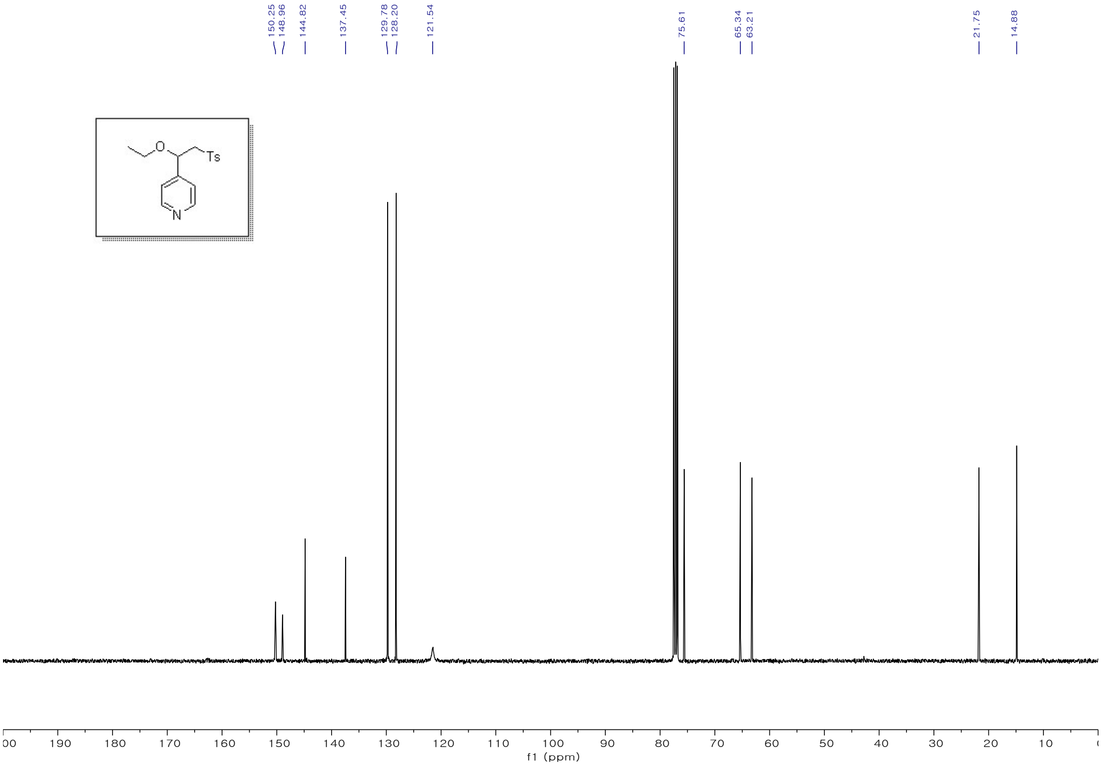

100 MHz, ^{13}C NMR in $CDCl_3$.

4-(1-(cyclohexyloxy)-2-tosylethyl)pyridine (4x).

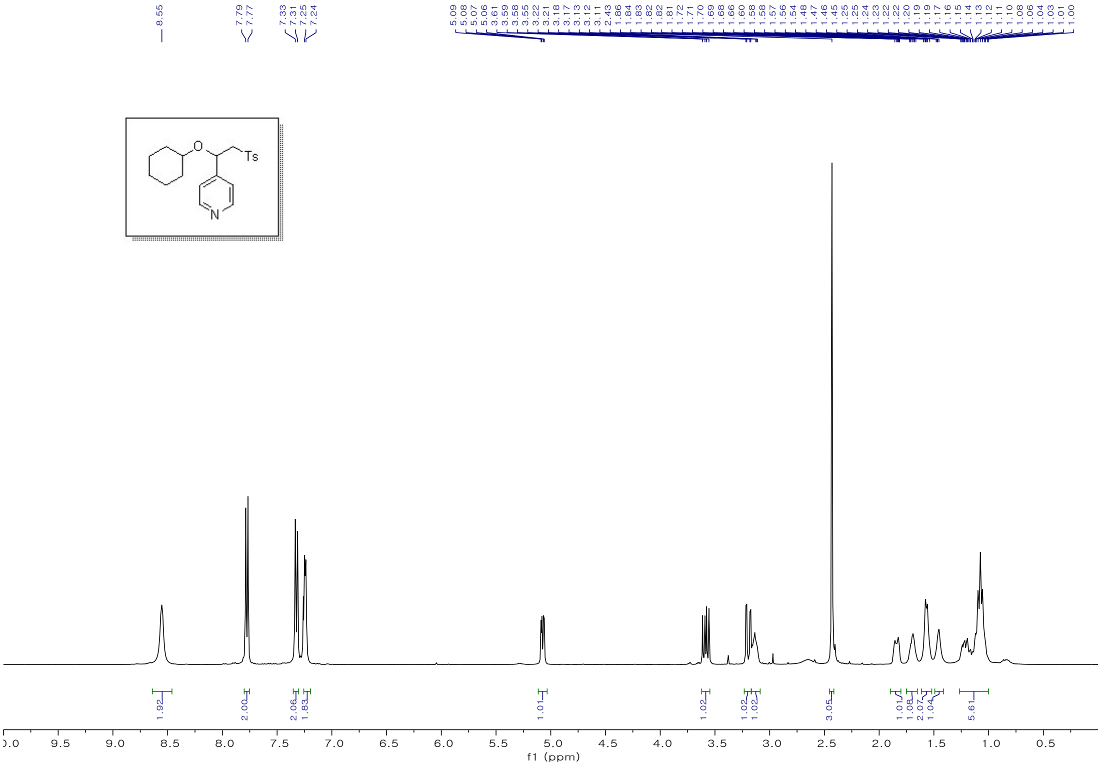

400 MHz, ^{1}H NMR in CDCl$_3$.

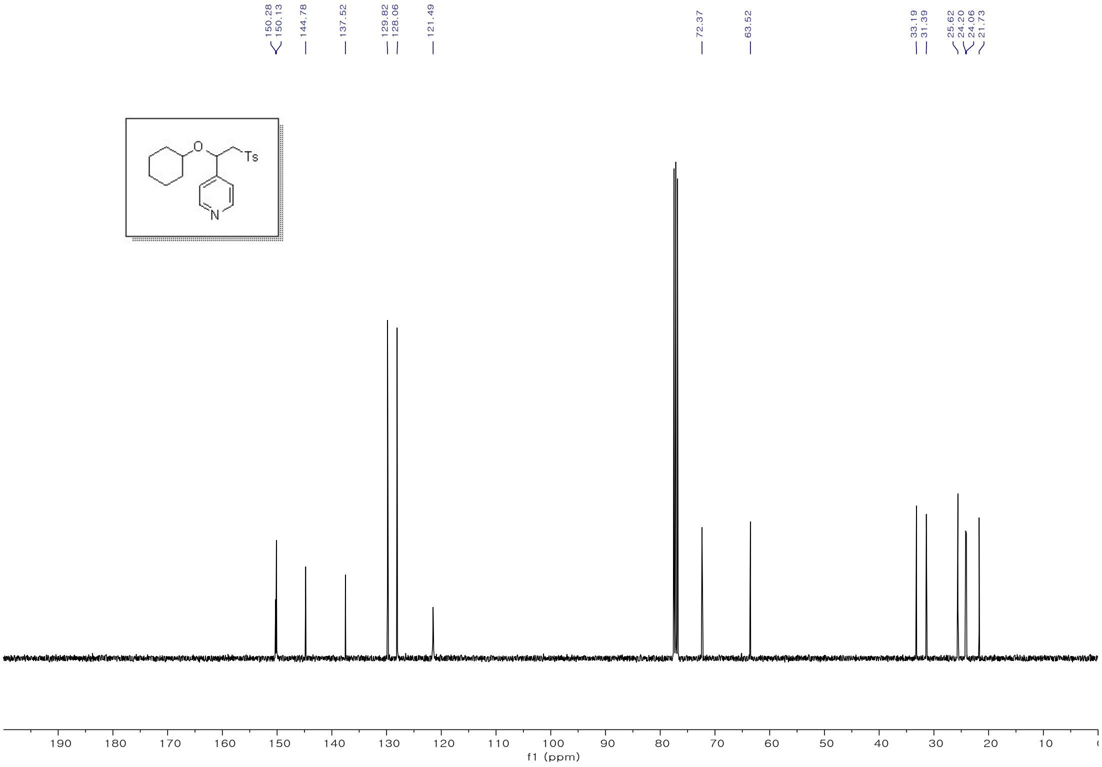

100 MHz, ^{13}C NMR in CDCl$_3$.

2-(1-(pyridin-4-yl)-2-tosylethoxy)ethan-1-ol (4y).

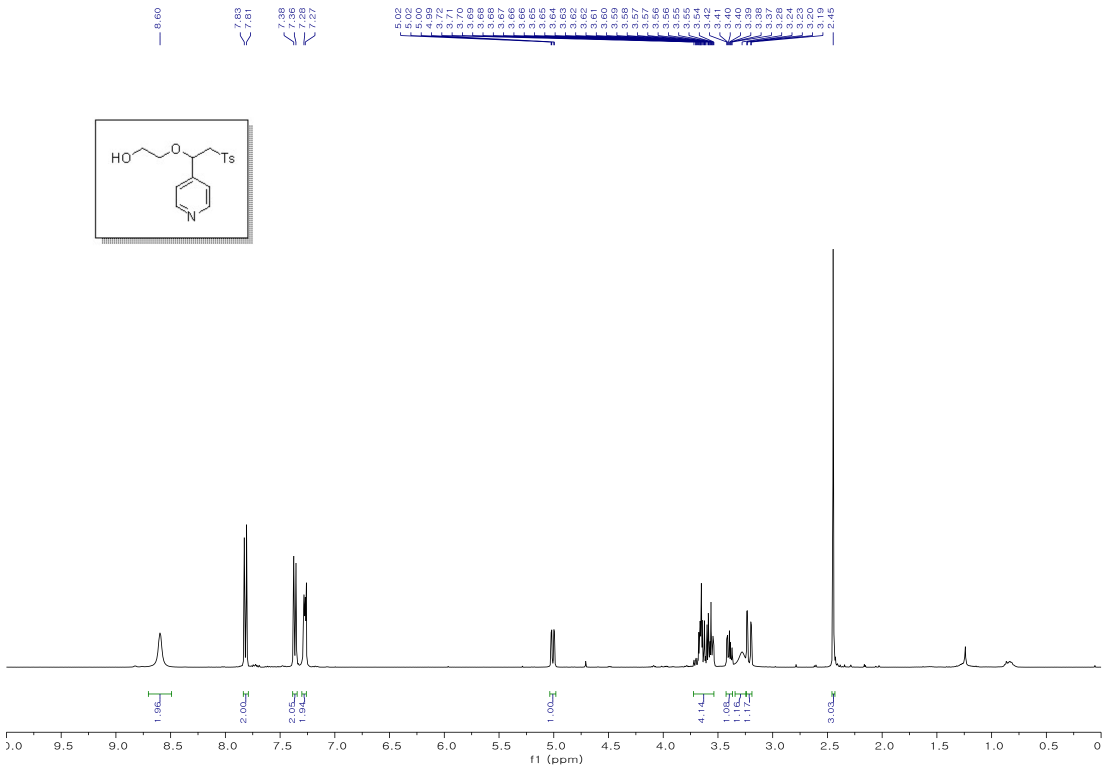

400 MHz, ^{1}H NMR in $CDCl_3$.

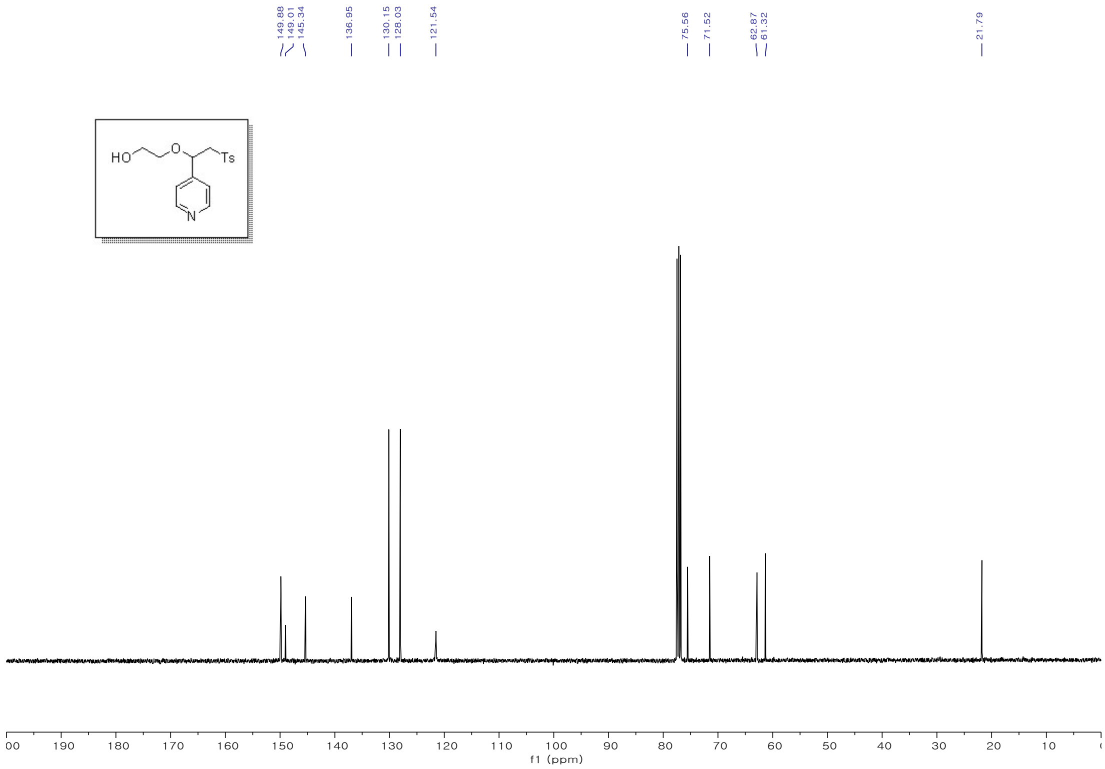

100 MHz, ^{13}C NMR in $CDCl_3$.

4-(1-(2-chloroethoxy)-2-tosylethyl)pyridine (4z).

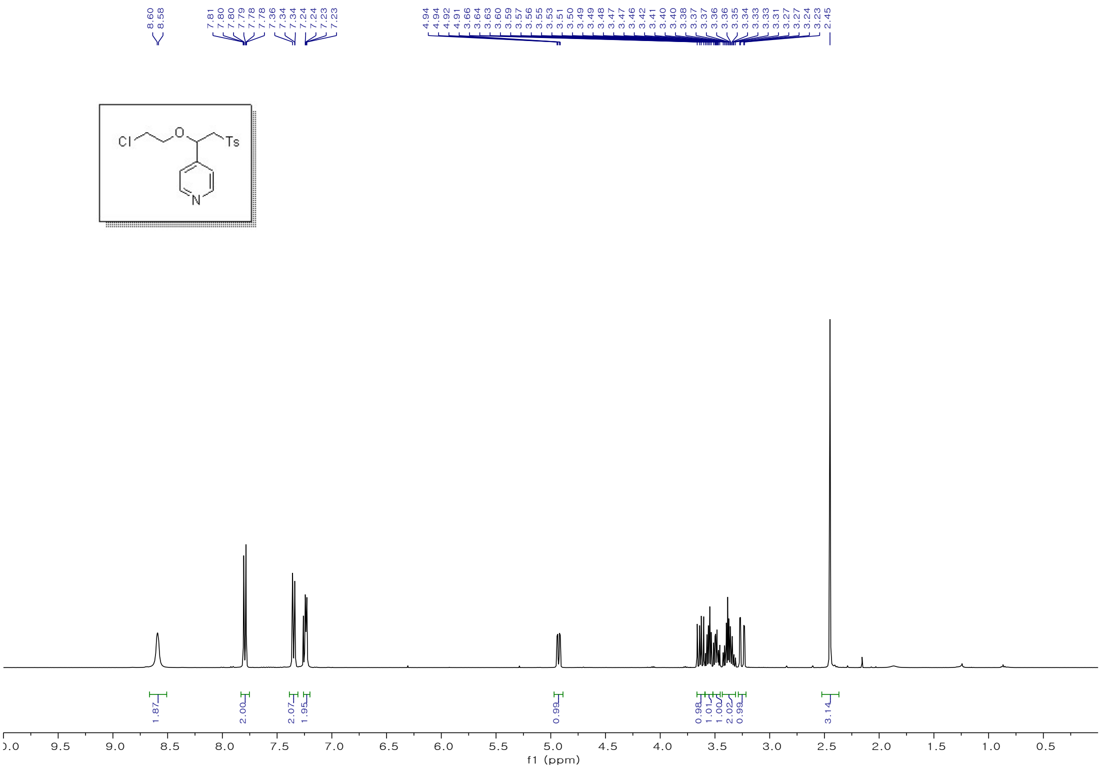

400 MHz, ^{1}H NMR in $CDCl_3$.

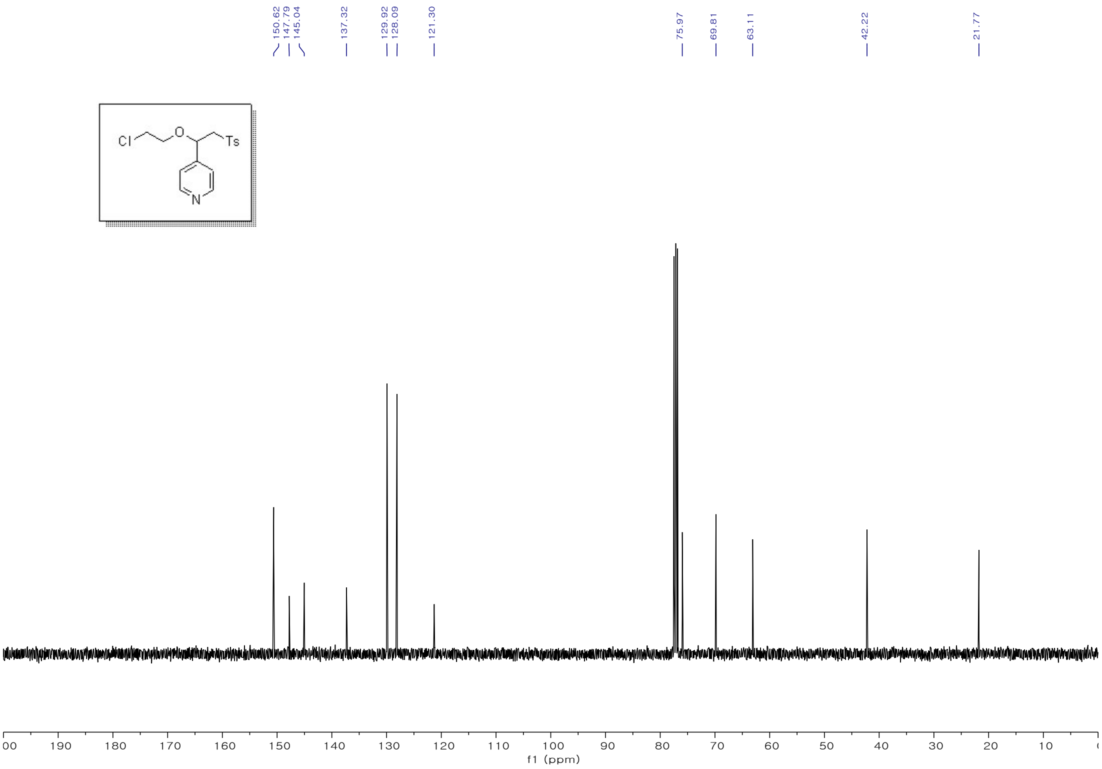

100 MHz, ^{13}C NMR in $CDCl_3$.

4-(1-phenoxy-2-tosylethyl)pyridine (4aa).

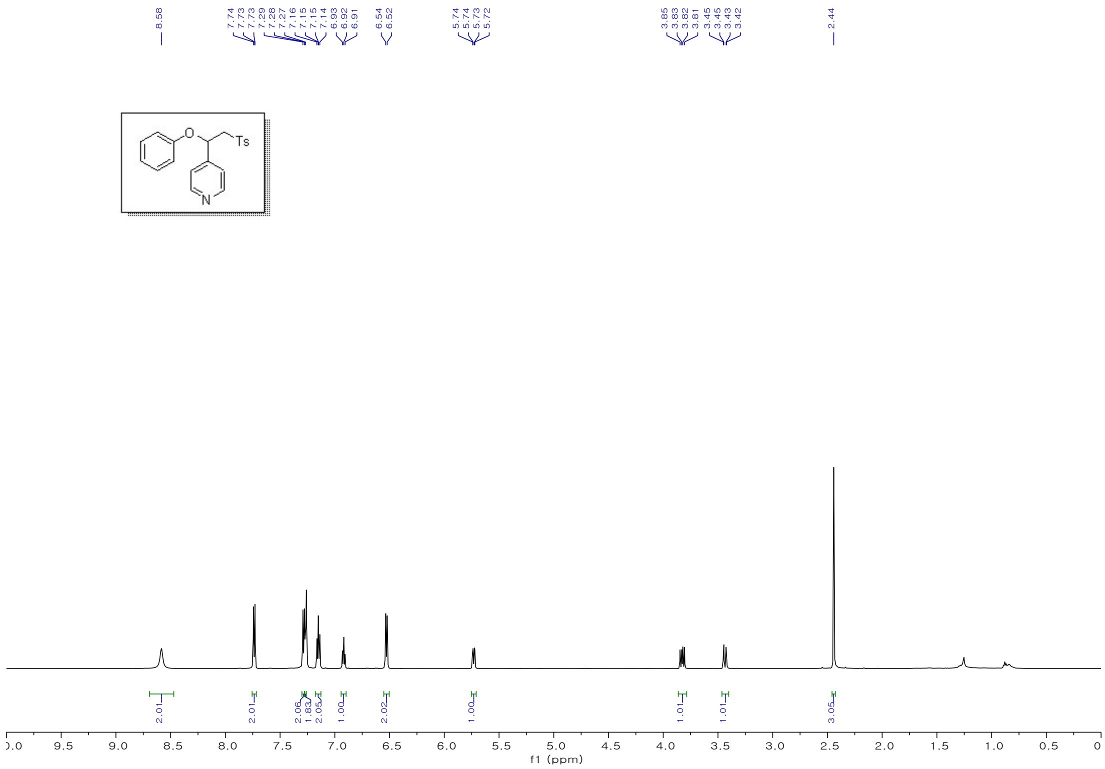

600 MHz, ^{1}H NMR in $CDCl_3$.

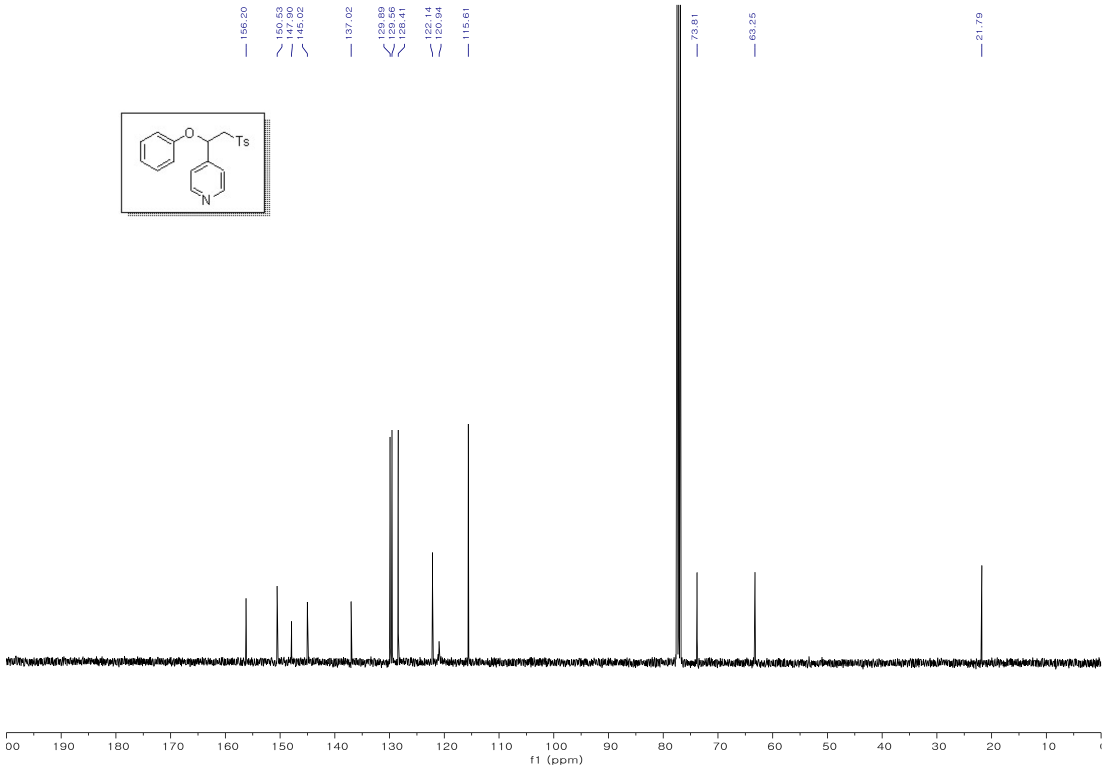

100 MHz, ^{13}C NMR in $CDCl_3$.

4-(1-(4-(tert-butyl)phenoxy)-2-tosylethyl)pyridine (4ab).

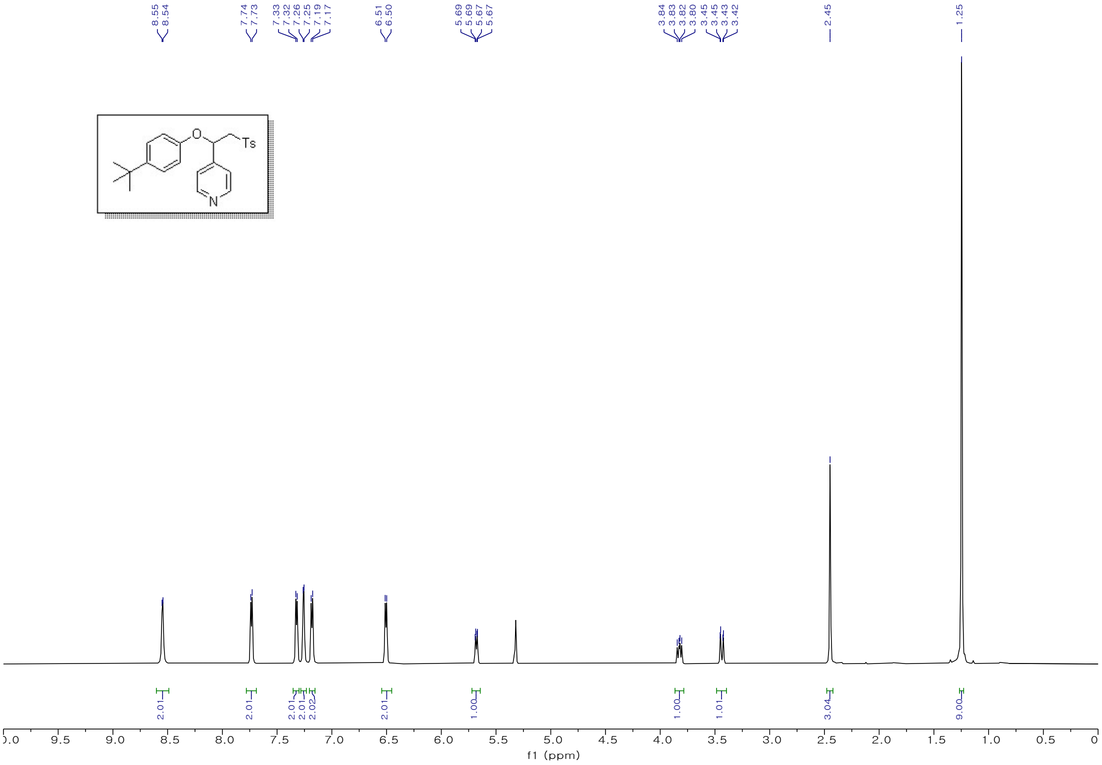

600 MHz, ^{1}H NMR in CD_2Cl_2

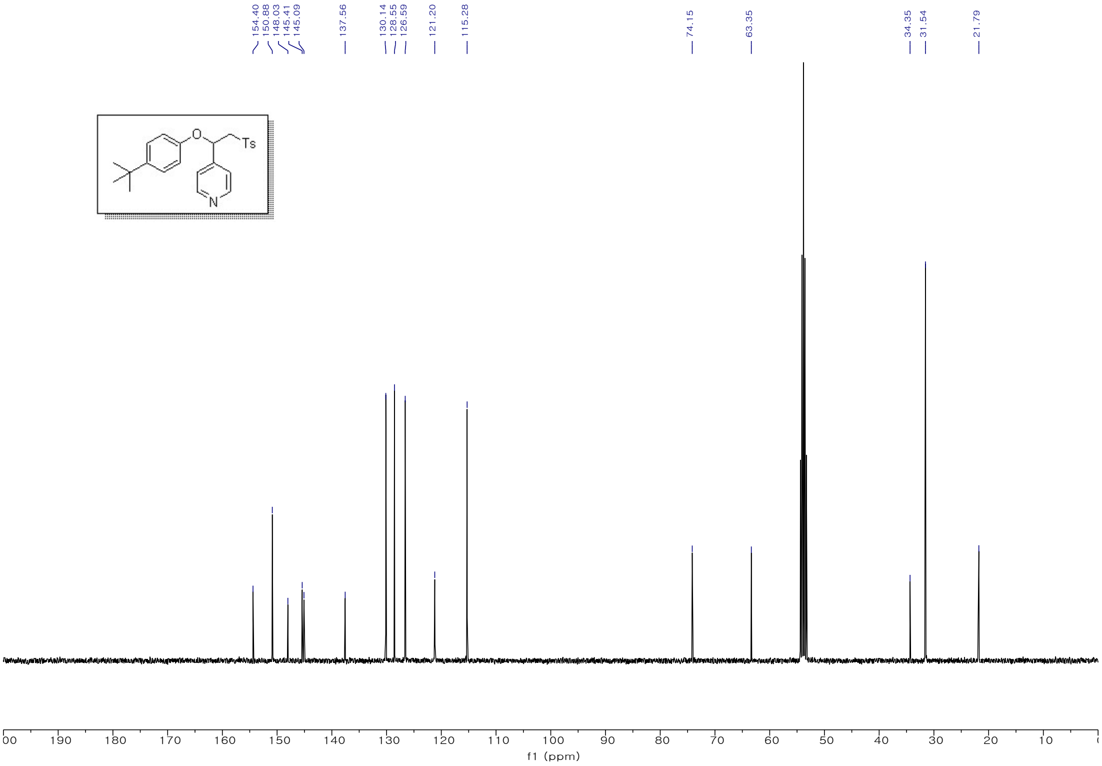

100 MHz, ^{13}C NMR in CD_2Cl_2

4-(1-(benzo[d][1,3]dioxol-5-yloxy)-2-tosylethyl)pyridine (4ac).

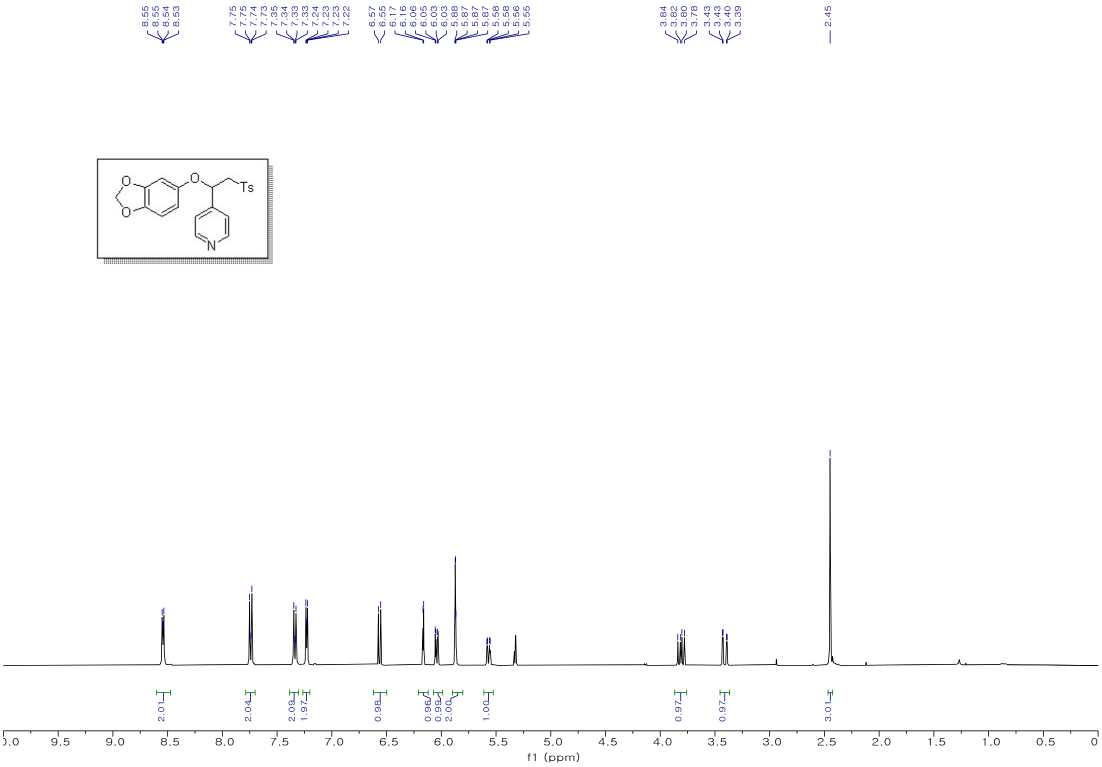

400 MHz, ^{1}H NMR in CD_2Cl_2

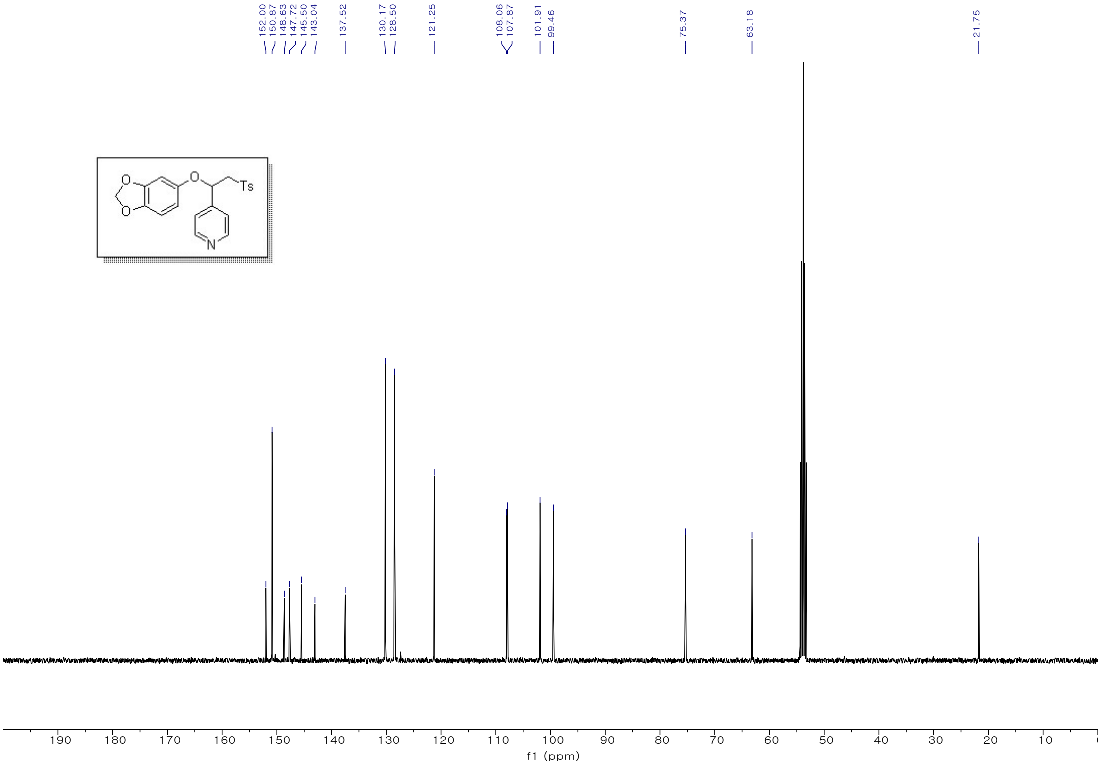

100 MHz, ^{13}C NMR in CD_2Cl_2

6-(1-(pyridin-4-yl)-2-tosylethoxy)-2H-chromen-2-one (4ad).

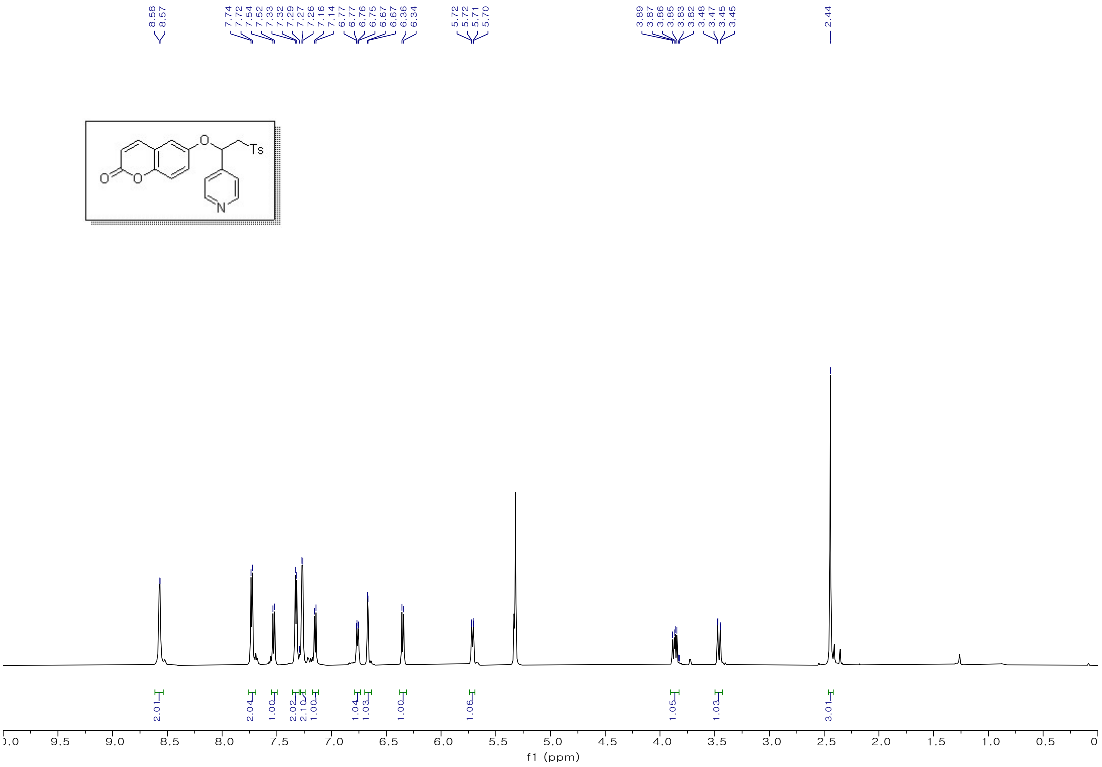

600 MHz, ^{1}H NMR in CD_2Cl_2.

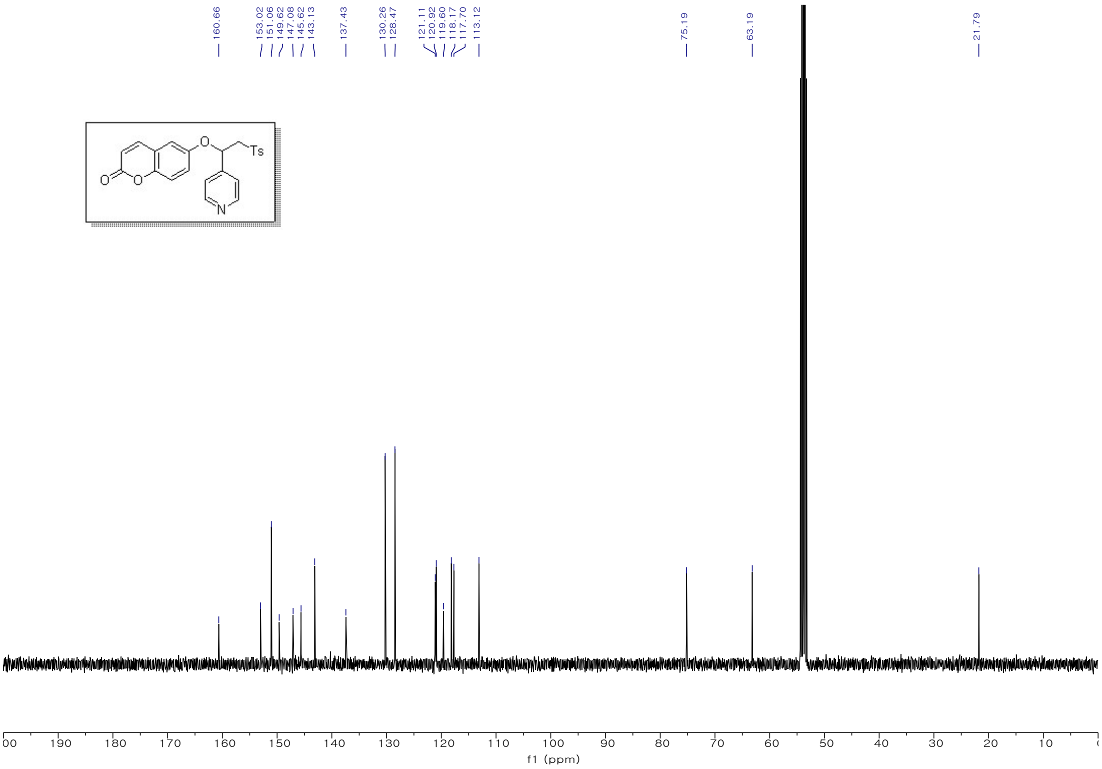

100 MHz, ^{13}C NMR in CD_2Cl_2

.*N*-(1-(pyridin-4-yl)-2-tosylethyl)formamide (4ae).

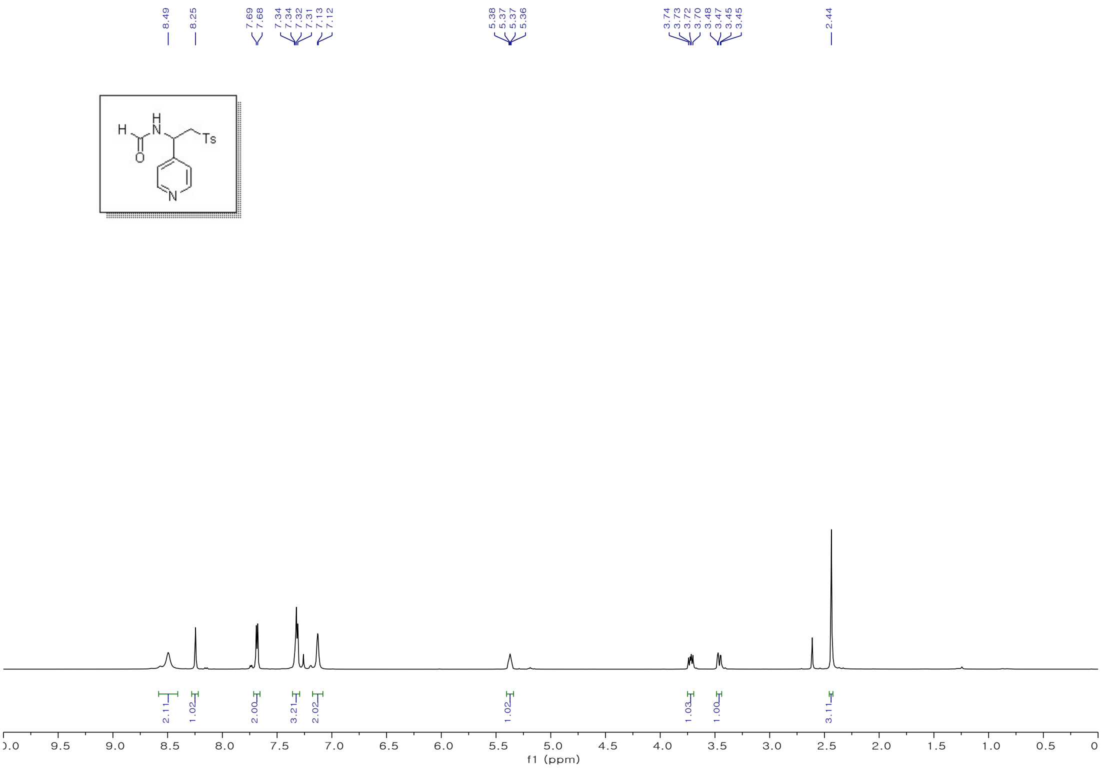

400 MHz, ^{1}H NMR in $CDCl_3$.

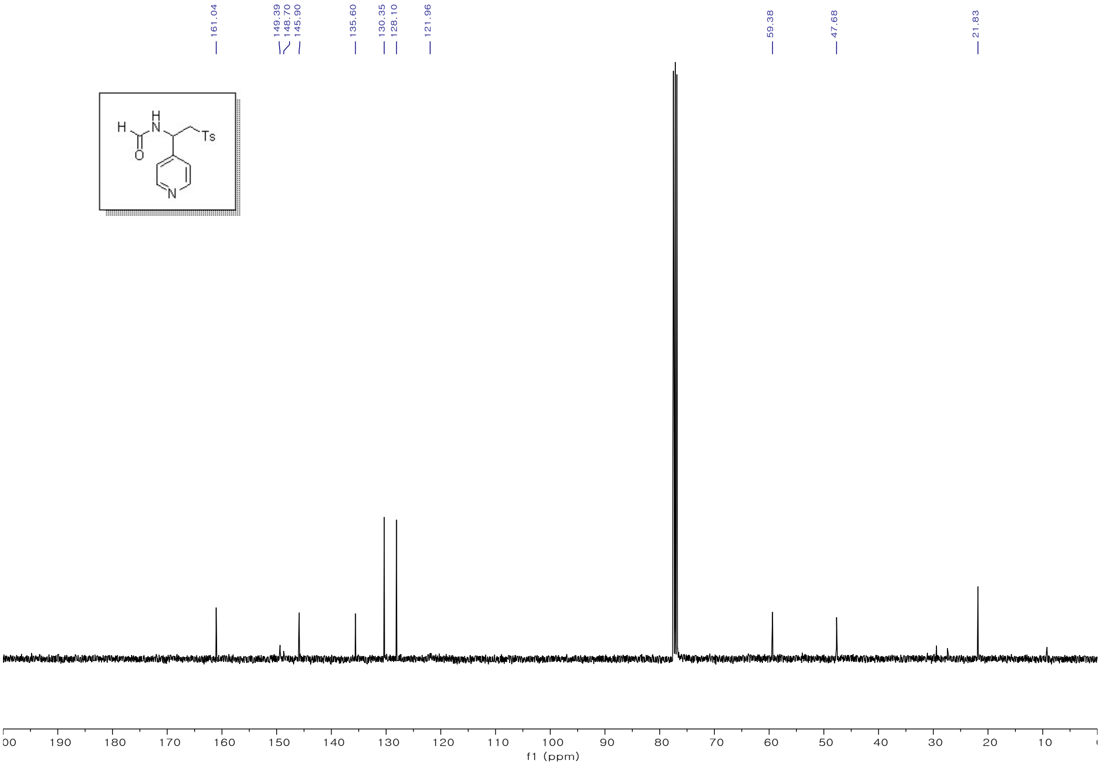

100 MHz, ^{13}C NMR in $CDCl_3$.

***N*-(1-(pyridin-4-yl)-2-tosylethyl)acetamide (4af).**

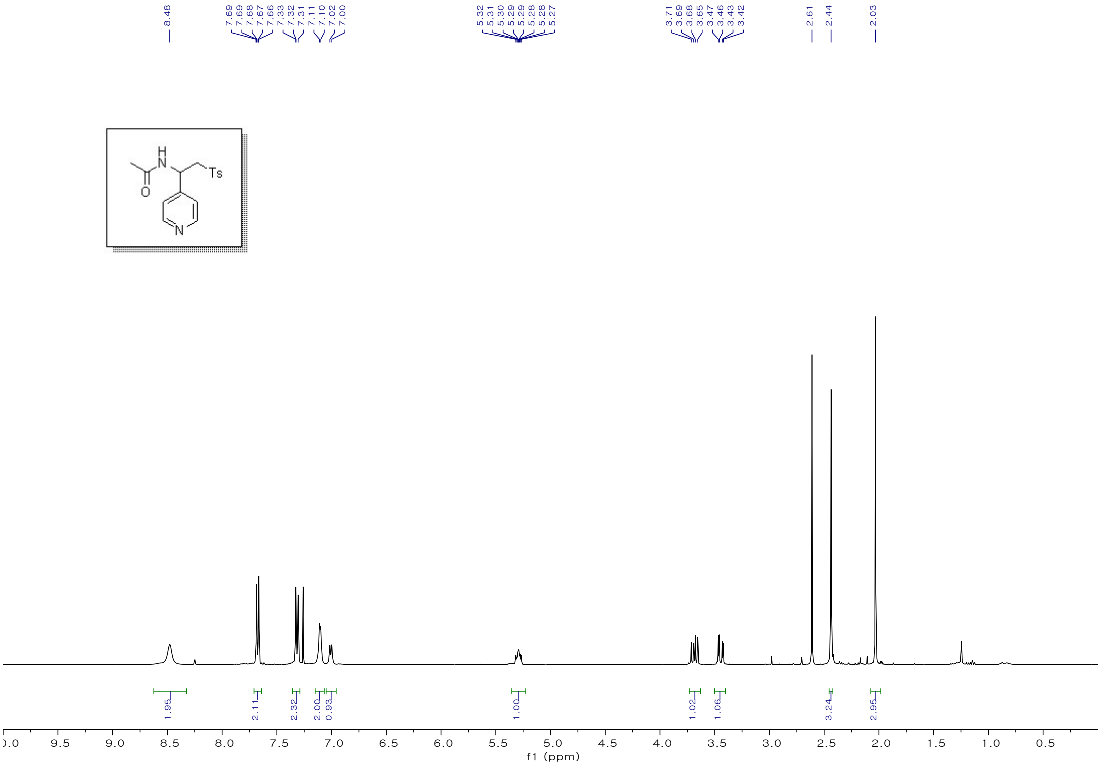

400 MHz, ^{1}H NMR in CDCl$_3$.

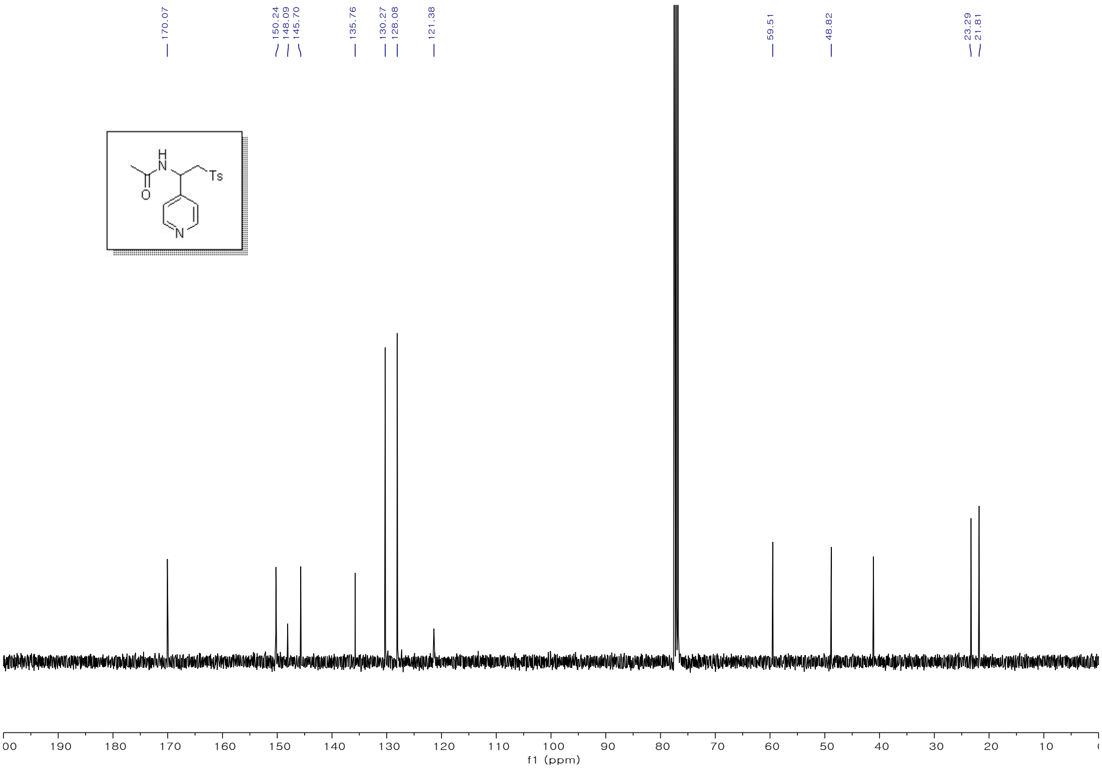

100 MHz, ^{13}C NMR in CDCl$_3$.

1-(1-(pyridin-4-yl)-2-tosylethyl)azepan-2-one (4ag).

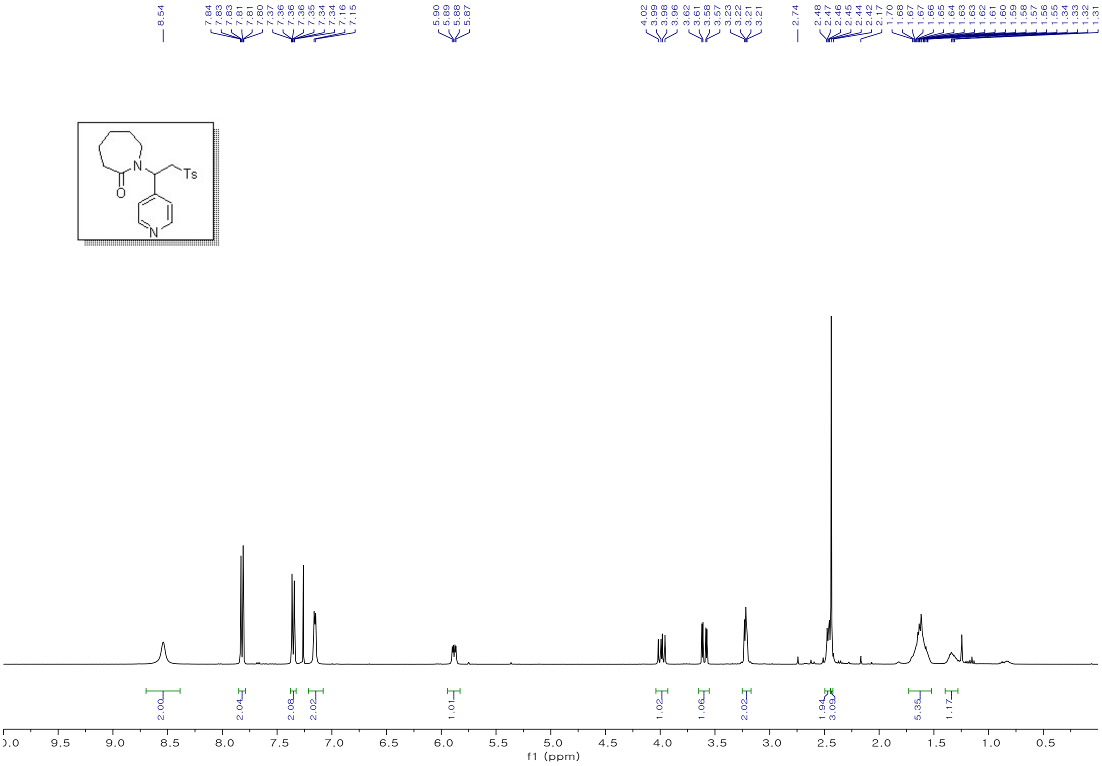

400 MHz, ^{1}H NMR in $CDCl_3$.

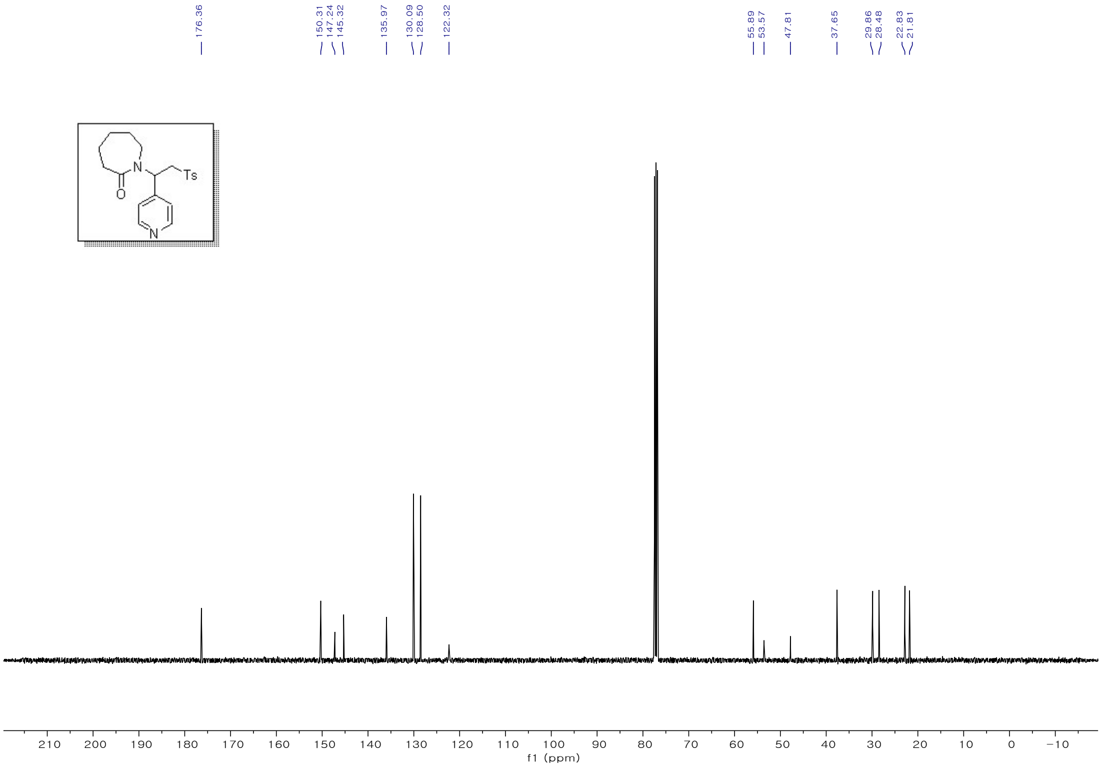

100 MHz, ^{13}C NMR in $CDCl_3$.

4-((2S,3R)-3-tosyltetrahydrofuran-2-yl)pyridine (4ah).

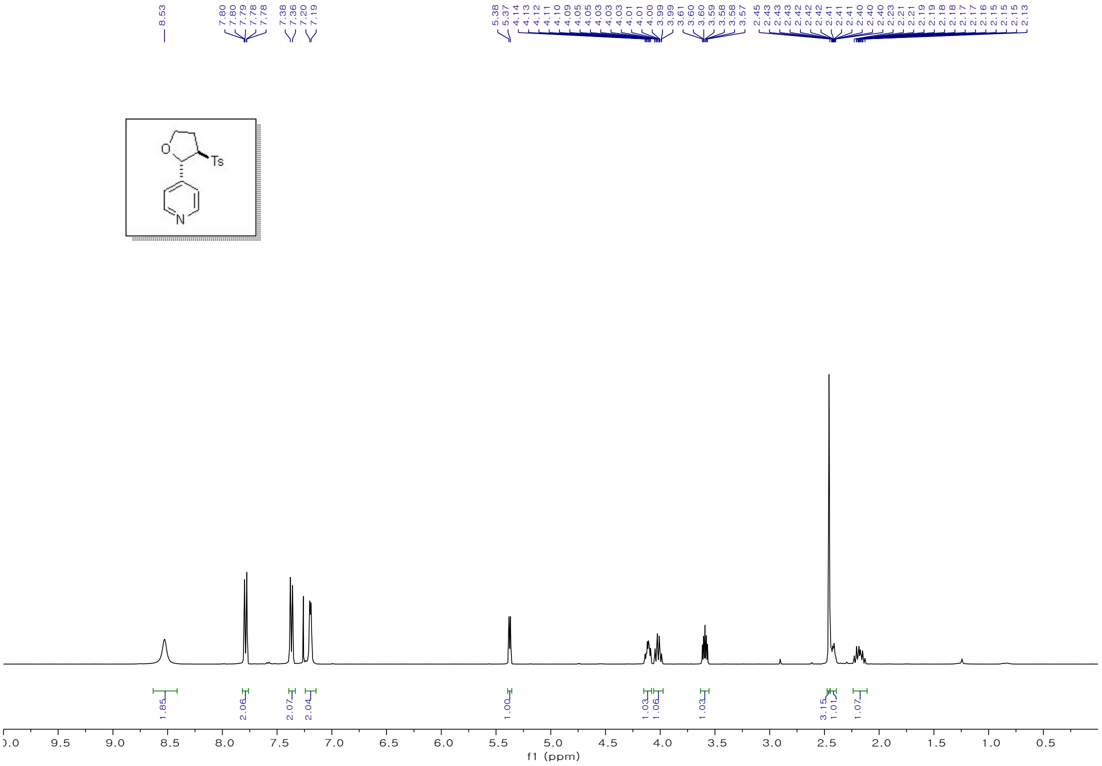

400 MHz, ^{1}H NMR in $CDCl_3$.

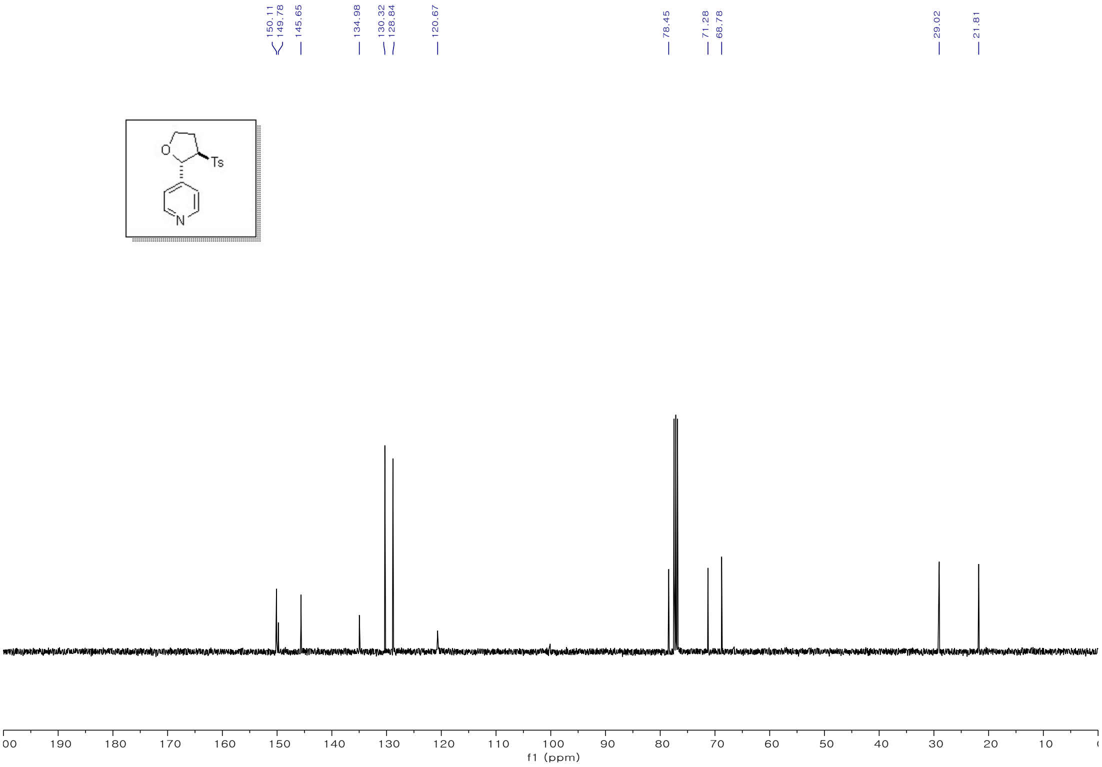

100 MHz, ^{13}C NMR in $CDCl_3$.

4-(1-(tosylmethyl)cyclobutyl)pyridine (4ai).

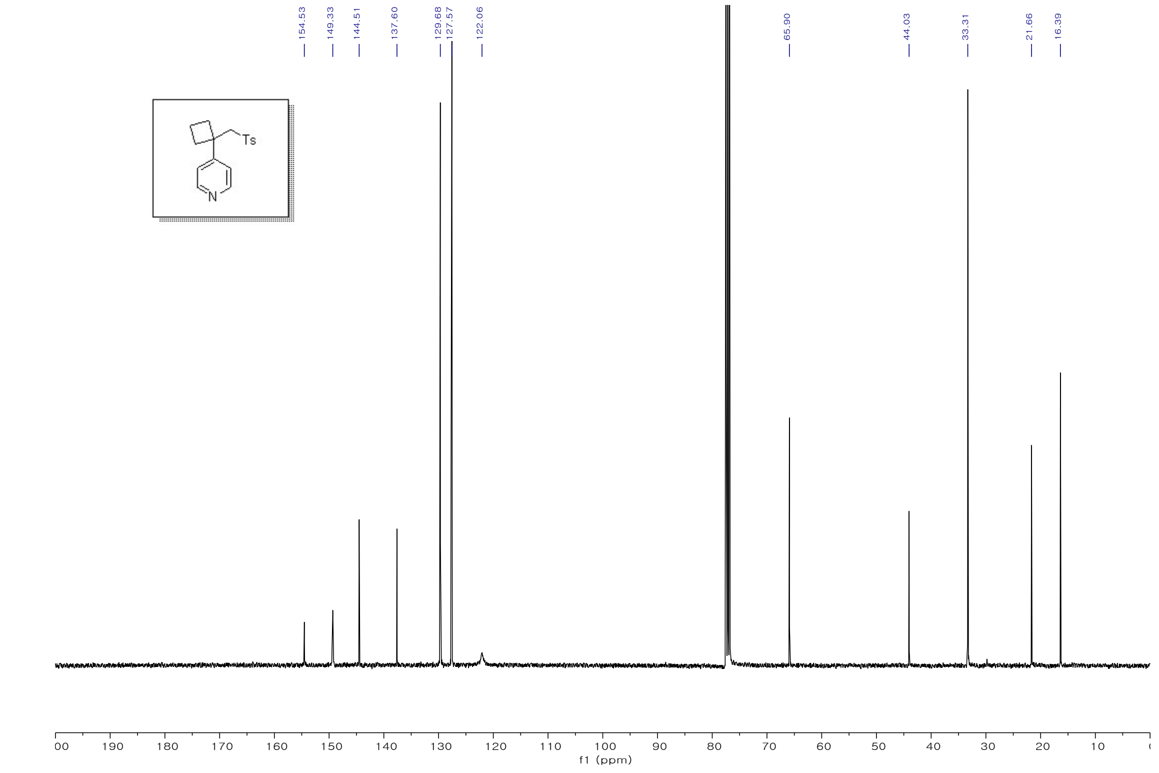

400 MHz, ^{1}H NMR in $CDCl_3$.

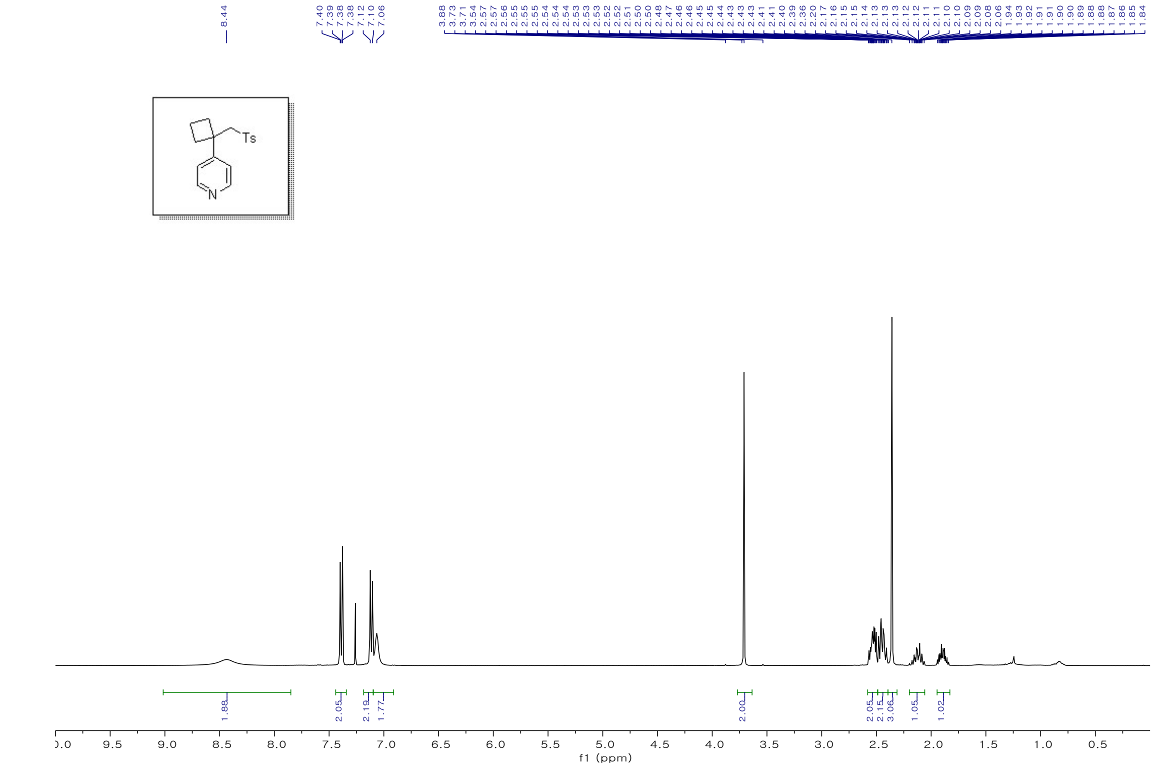

100 MHz, ^{13}C NMR in $CDCl_3$.

4-(1-(tosylmethyl)cyclopentyl)pyridine (4aj).

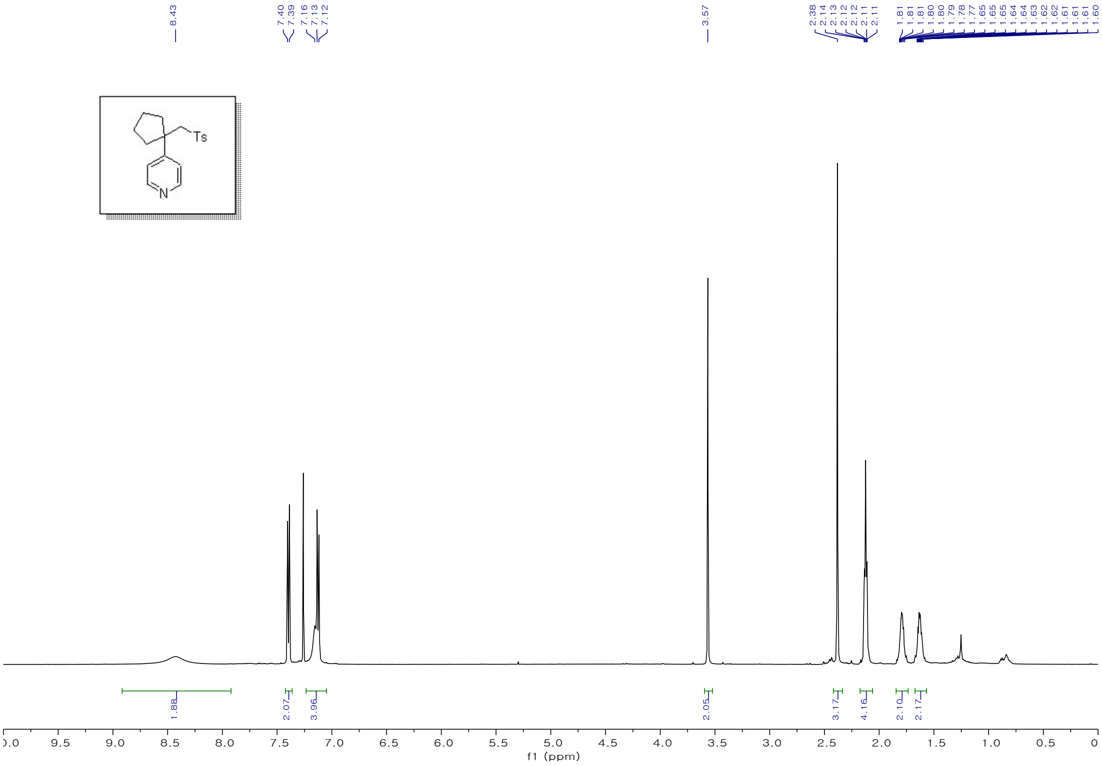

500 MHz, ¹H NMR in CDCl₃.

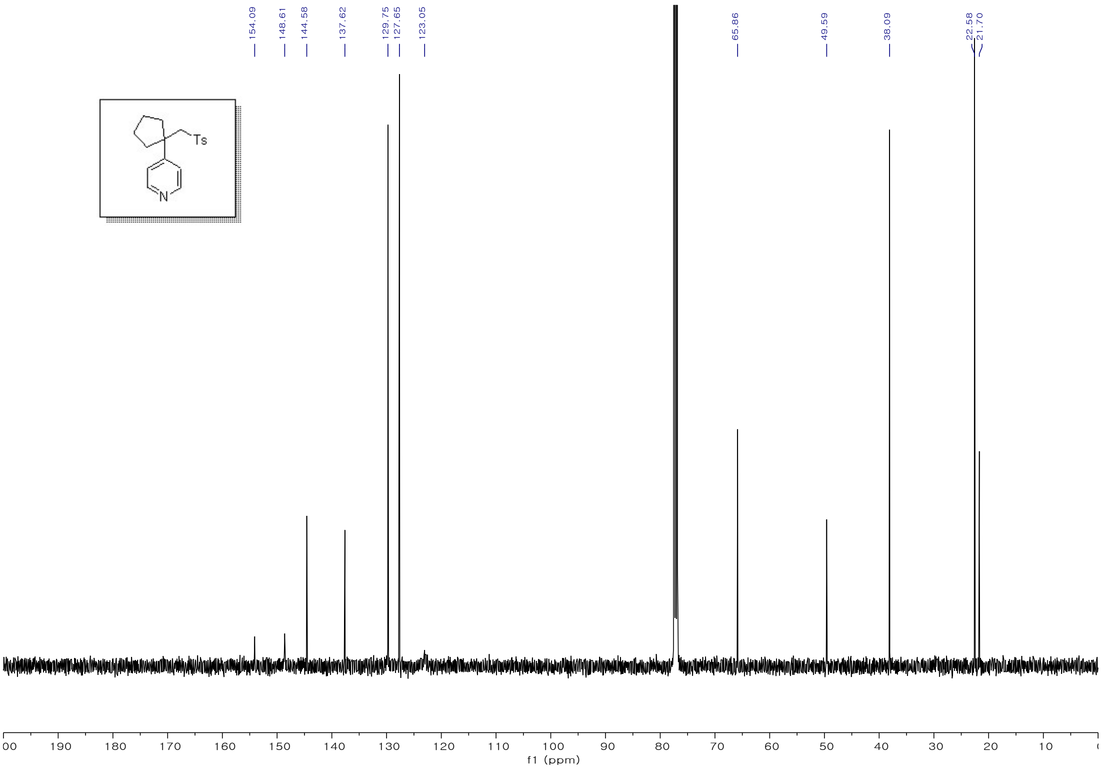

125 MHz, ¹³C NMR in CDCl₃.

4-(pyridin-4-yl)-5-tosylpentyl 4-methoxybenzoate (4ak).

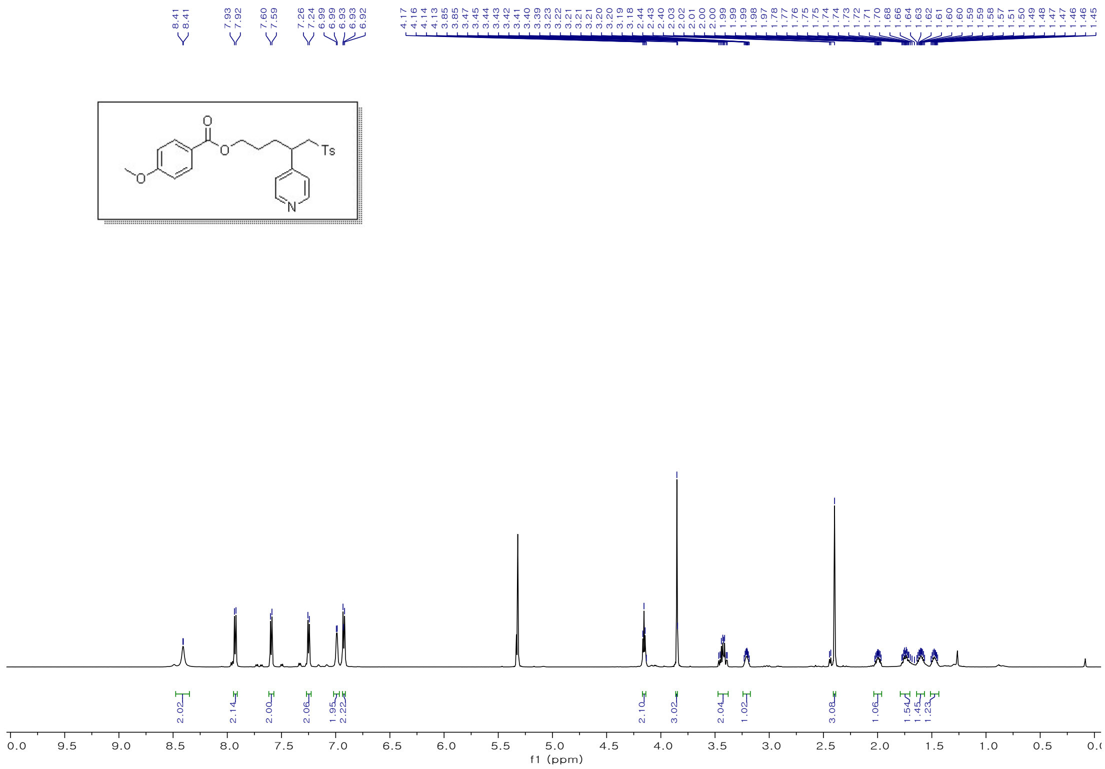

600 MHz, ^{1}H NMR in CD_2Cl_2.

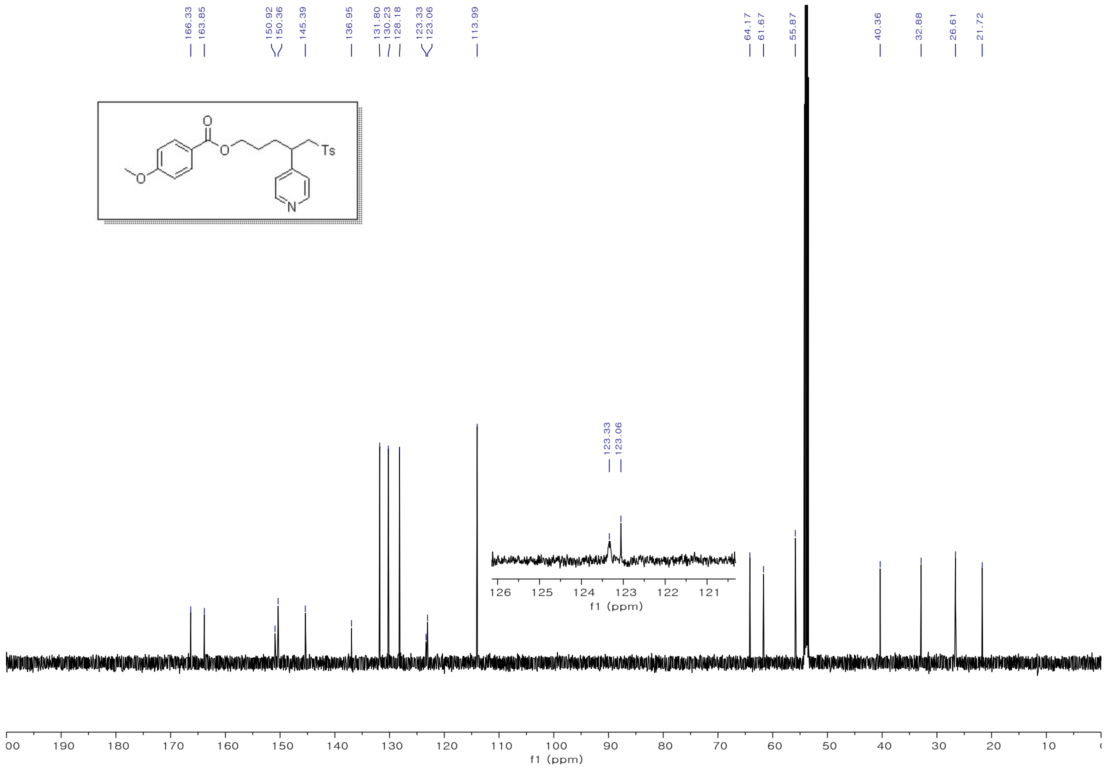

150 MHz, ^{13}C NMR in CD_2Cl_2.

4-(1-(((3aR,5aS,8aS,8bR)-2,2,7,7-tetramethyltetrahydro-3a*H*-bis([1,3]dioxolo)[4,5-b:4',5'-d]pyran-3a-yl)methoxy)-2-tosylethyl)pyridine (4al).

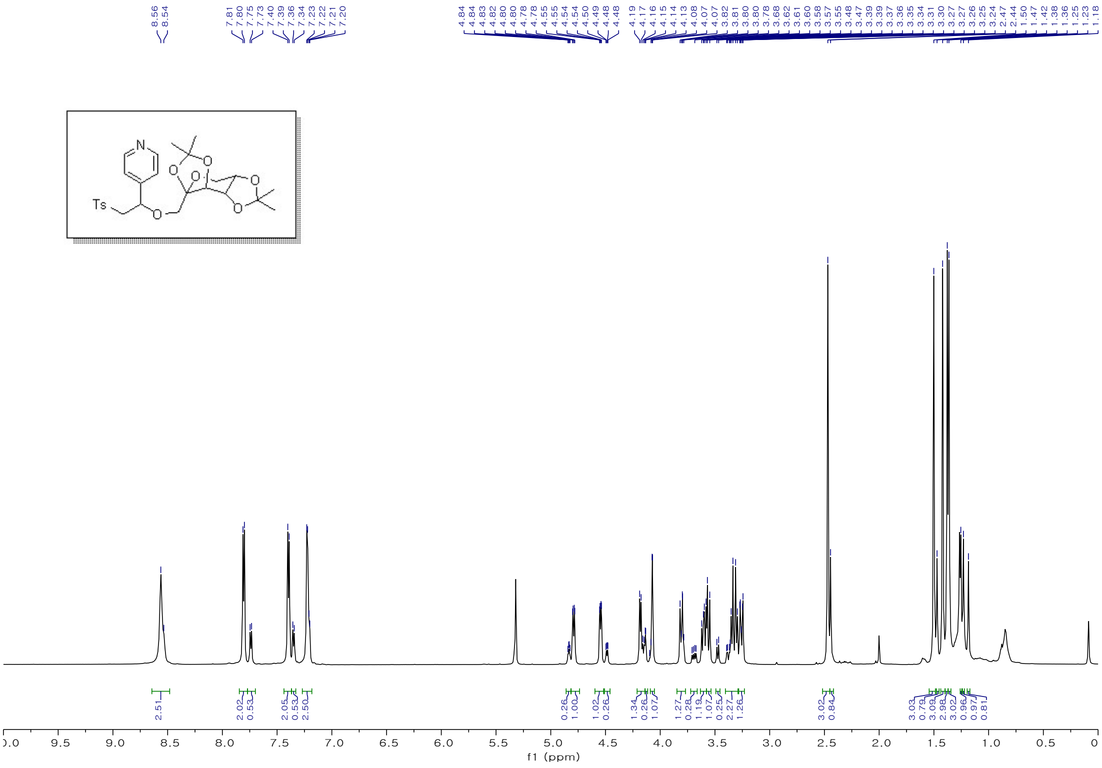

600 MHz, ^{1}H NMR in CD_2Cl_2.

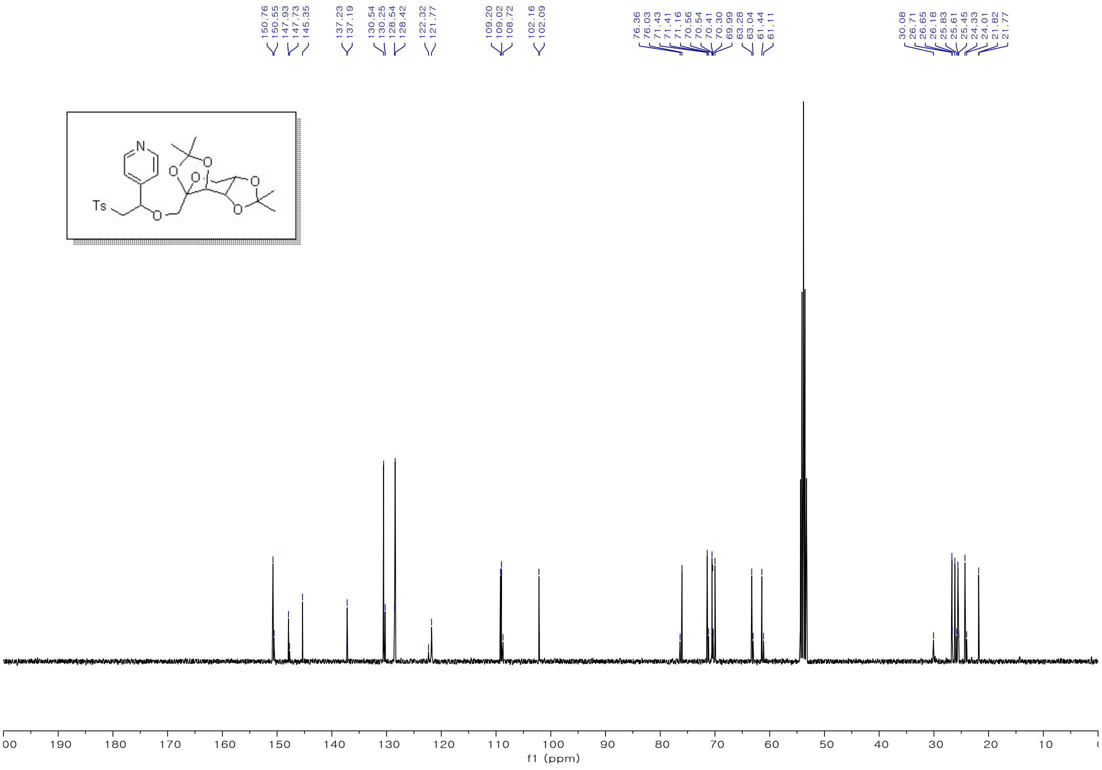

100 MHz, ^{13}C NMR in CD_2Cl_2.

4-(1-((3,7-dimethyloct-6-en-1-yl)oxy)-2-tosylethyl)pyridine (4am).

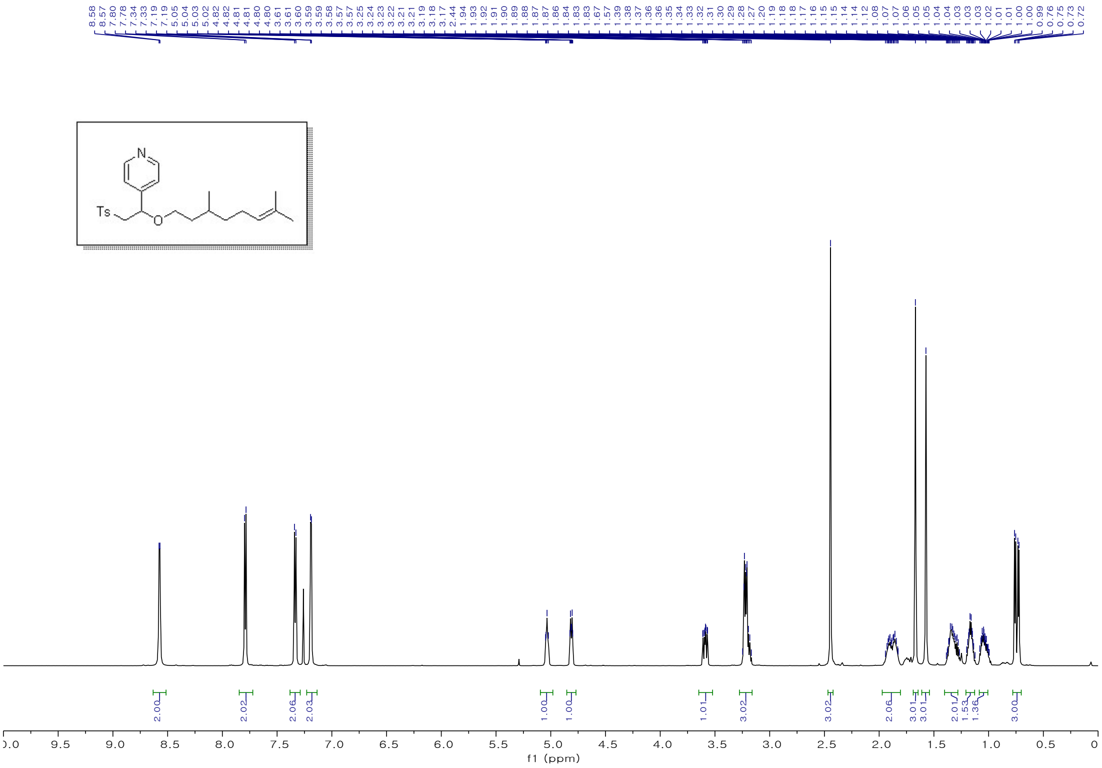

600 MHz, ^{1}H NMR in $CDCl_3$

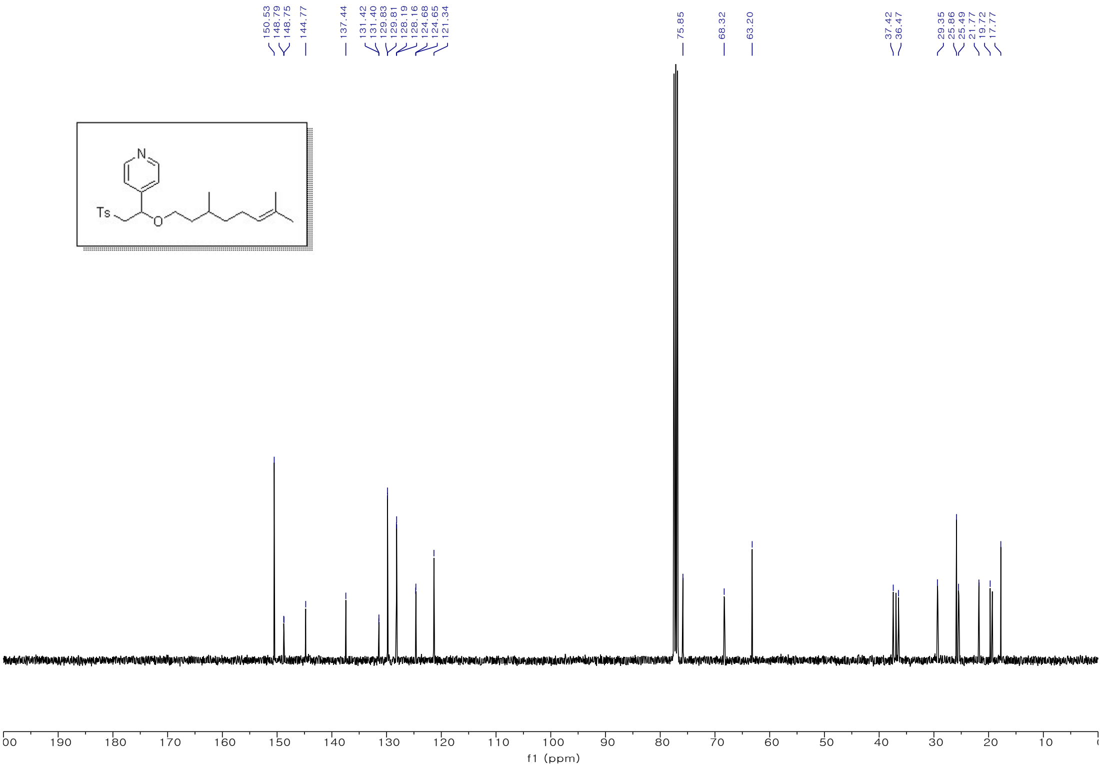

100 MHz, ^{13}C NMR in $CDCl_3$

methyl(2S)-2-((tert-butoxycarbonyl)amino)-3-(4-(1-(pyridin-4-yl)-2-tosylethoxy)phenyl)propanoate (4an).

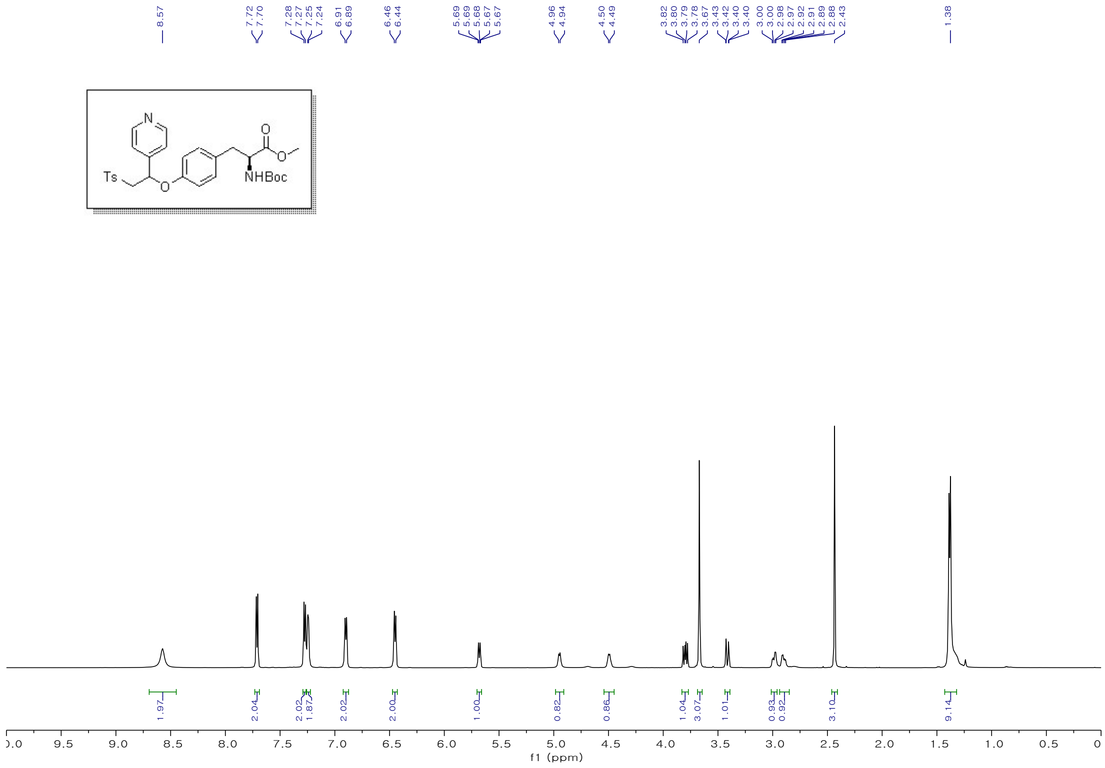

600 MHz, ^{1}H NMR in $CDCl_3$.

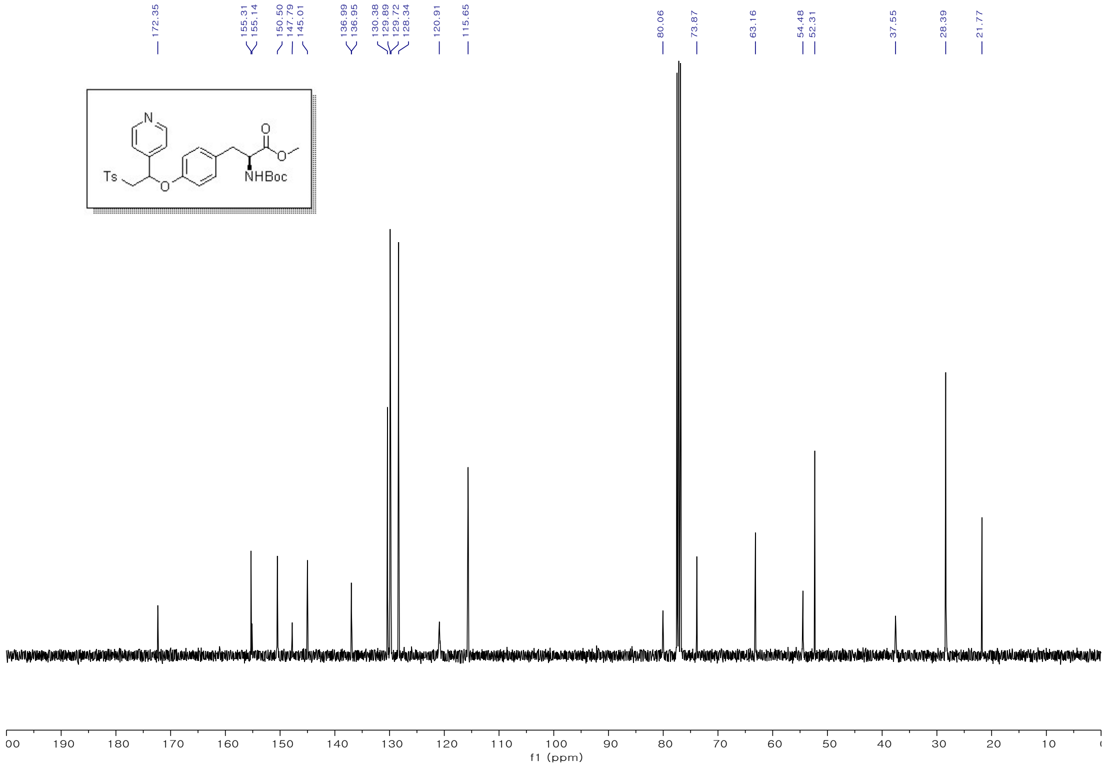

100 MHz, ^{13}C NMR in $CDCl_3$.

(8R,9S,13S,14S)-13-methyl-3-(1-(pyridin-4-yl)-2-tosylethoxy)-6,7,8,9,11,12,13,14,15,16-decahydro-17H-cyclopenta[a]phenanthren-17-one (4ao).

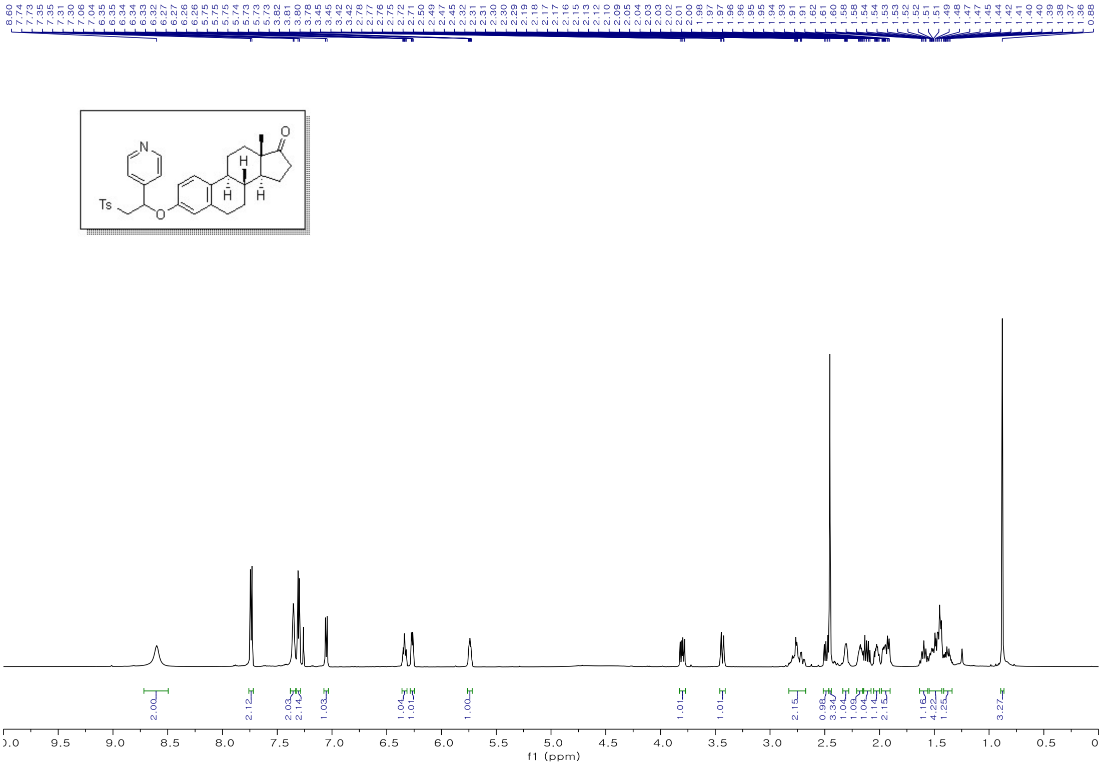

600 MHz, ^{1}H NMR in $CDCl_3$.

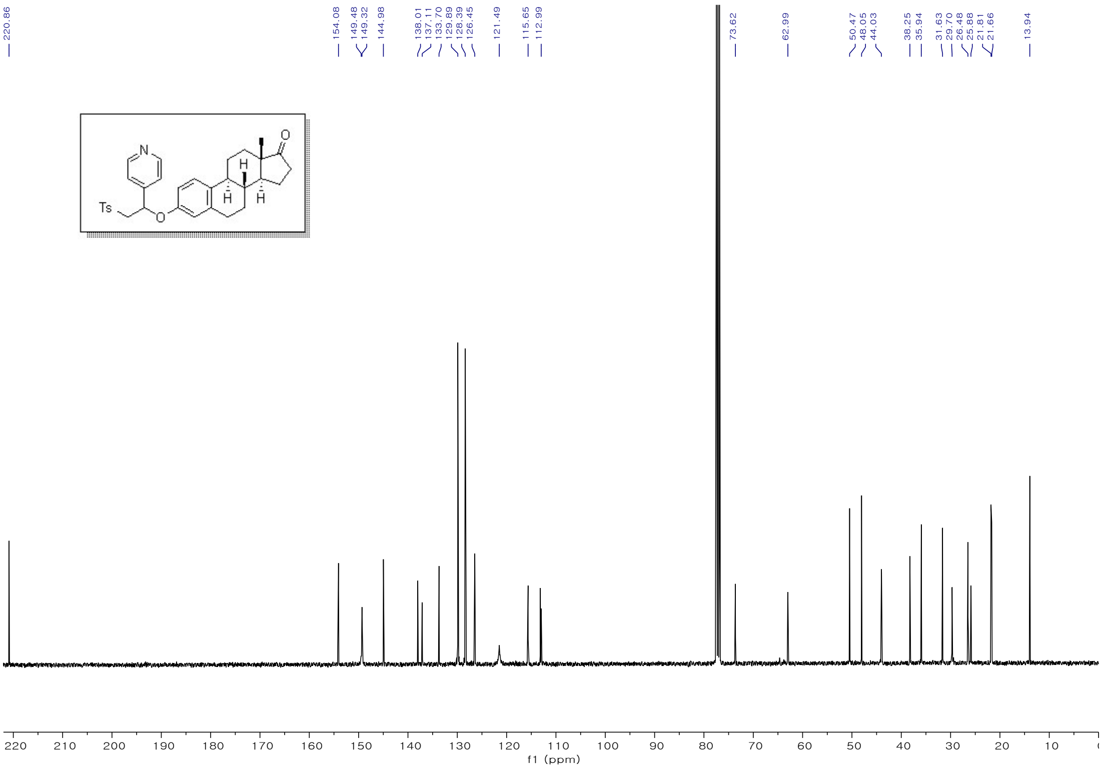

100 MHz, ^{13}C NMR in $CDCl_3$.

4-(1,1-diphenyl-5-(tosylmethyl)silinan-3-yl)pyridine (4ap).

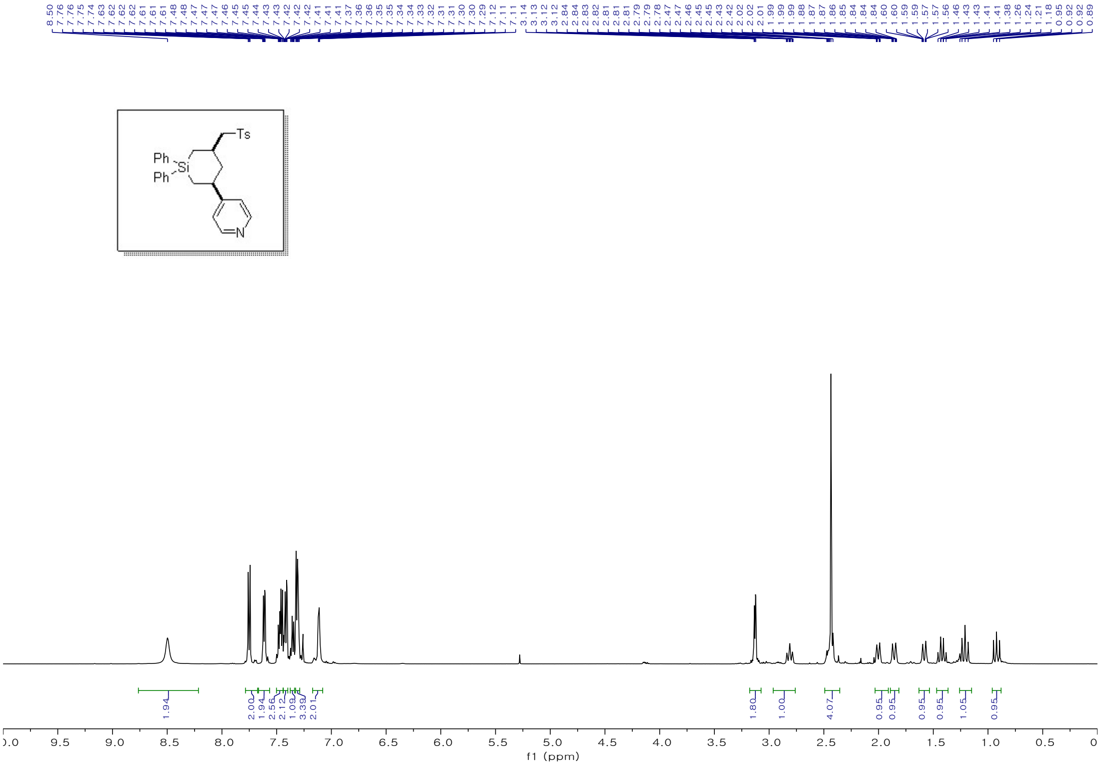

400 MHz, ^{1}H NMR in $CDCl_3$.

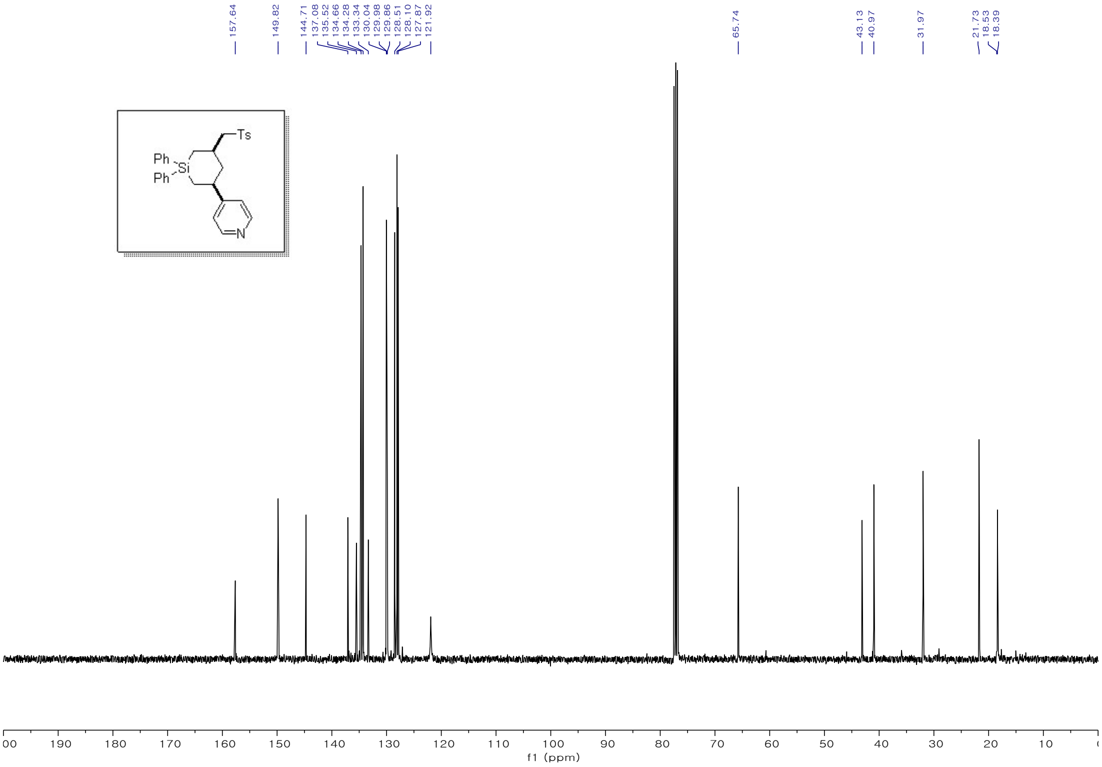

100 MHz, ^{13}C NMR in $CDCl_3$.

4-((3aR,6aS)-4-tosyloctahydropentalen-1-yl)pyridine (4aq).

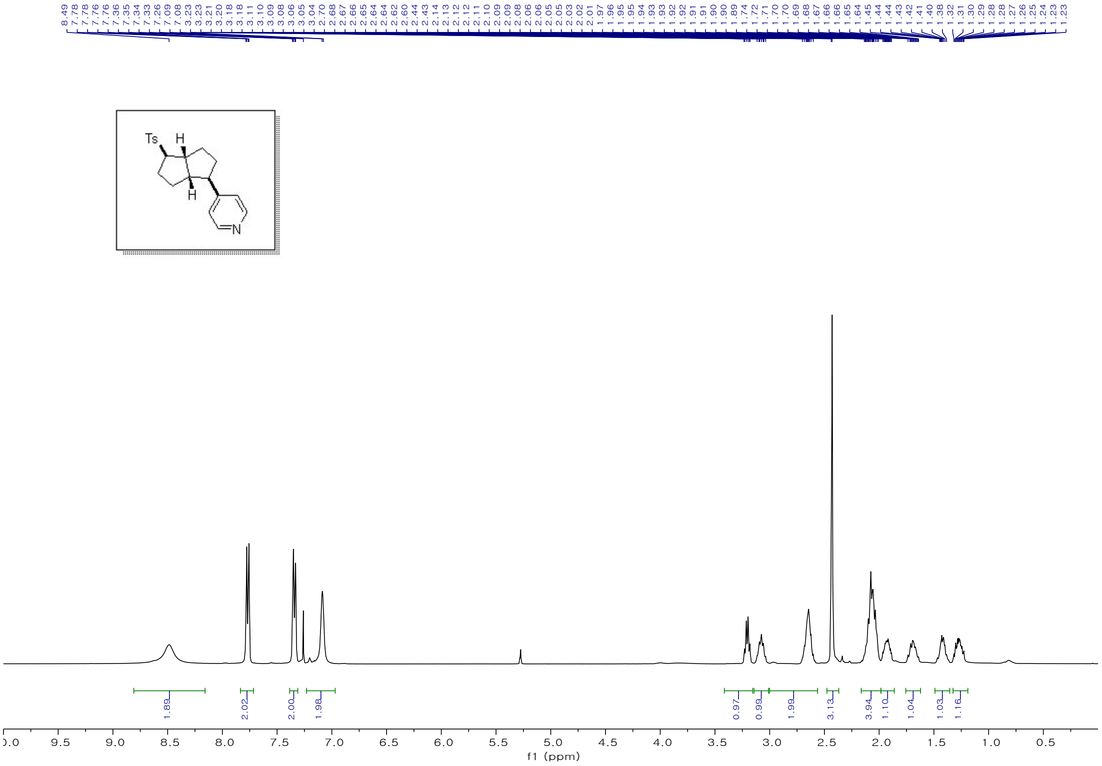

400 MHz, ^{1}H NMR in $CDCl_3$.

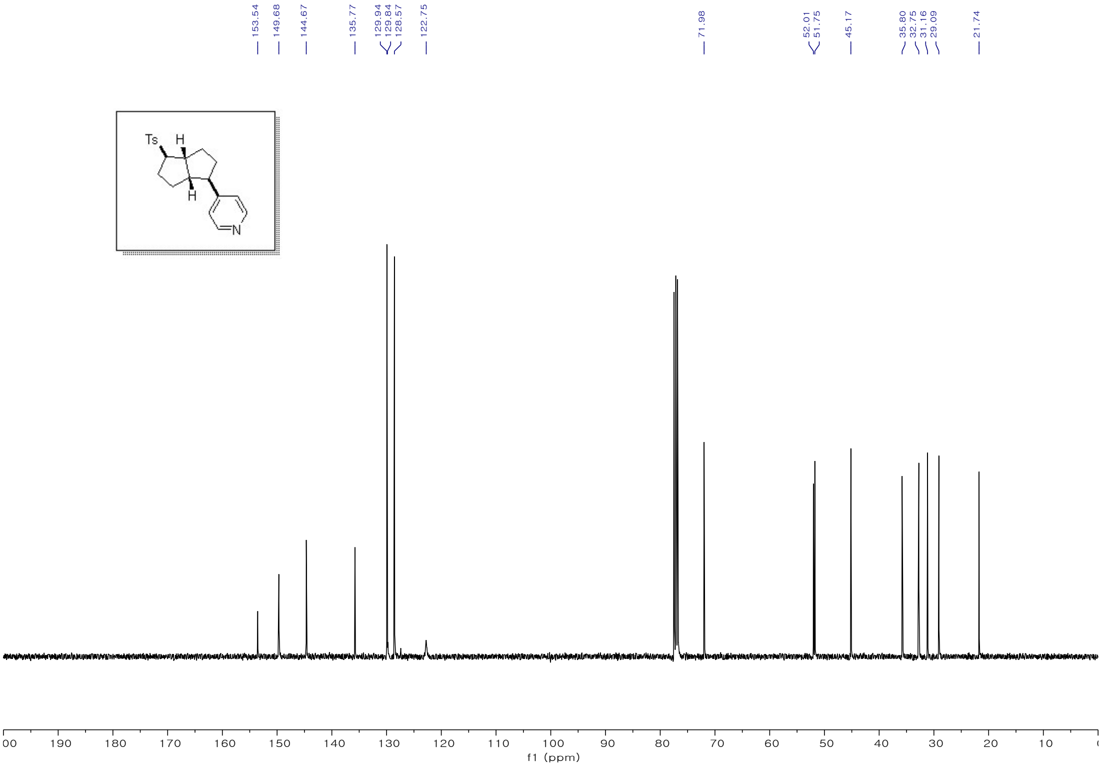

100 MHz, ^{13}C NMR in $CDCl_3$.

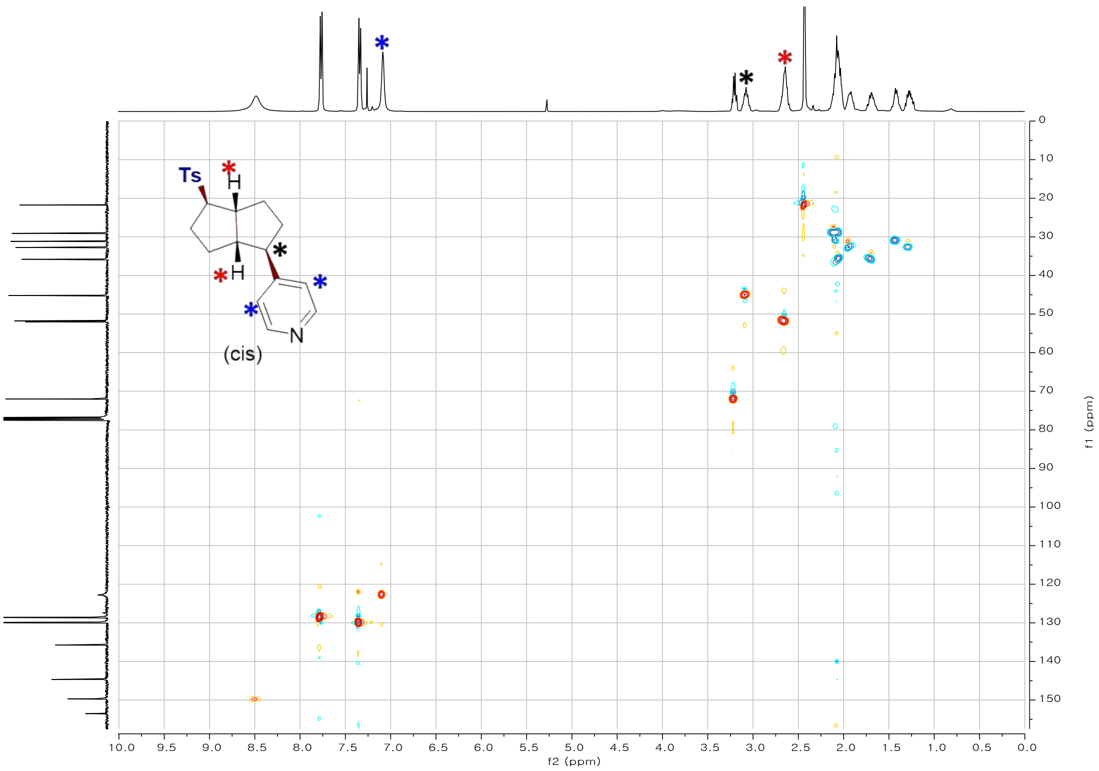

HSQC

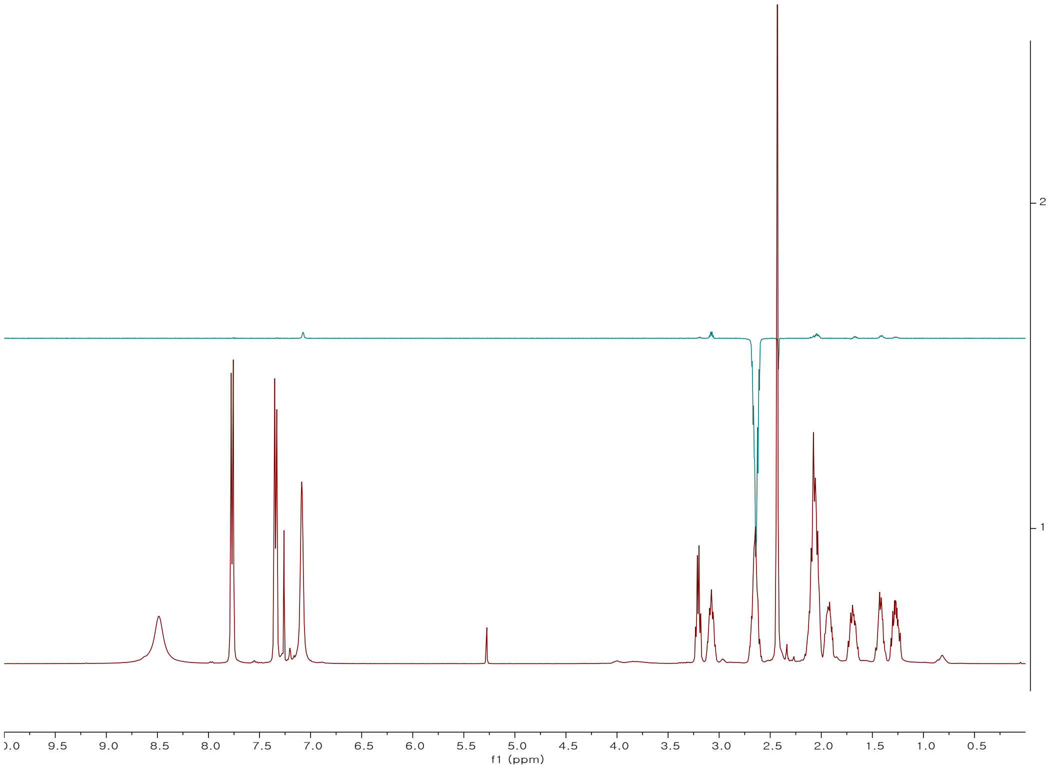

600 MHz, NOESY 1D in $CDCl_3$

4-(5-tosyltricyclo[2.2.1.02,6]heptan-3-yl)pyridine (4ar).

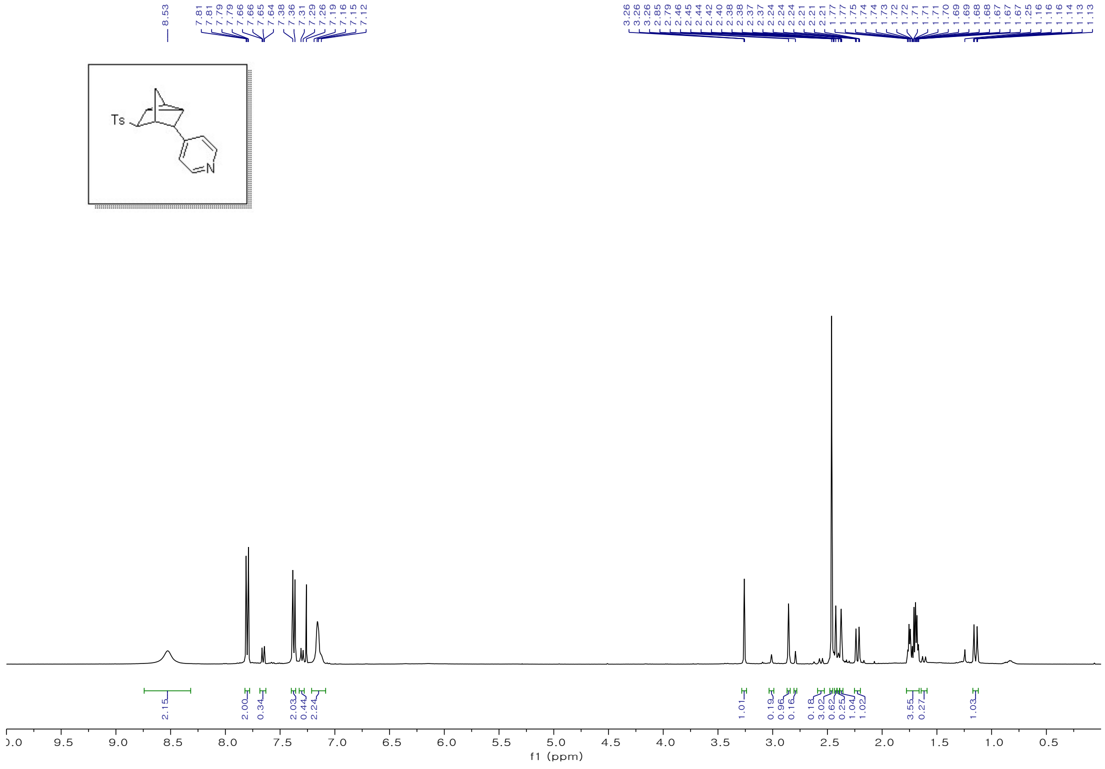

400 MHz, ^{1}H NMR in $CDCl_3$.

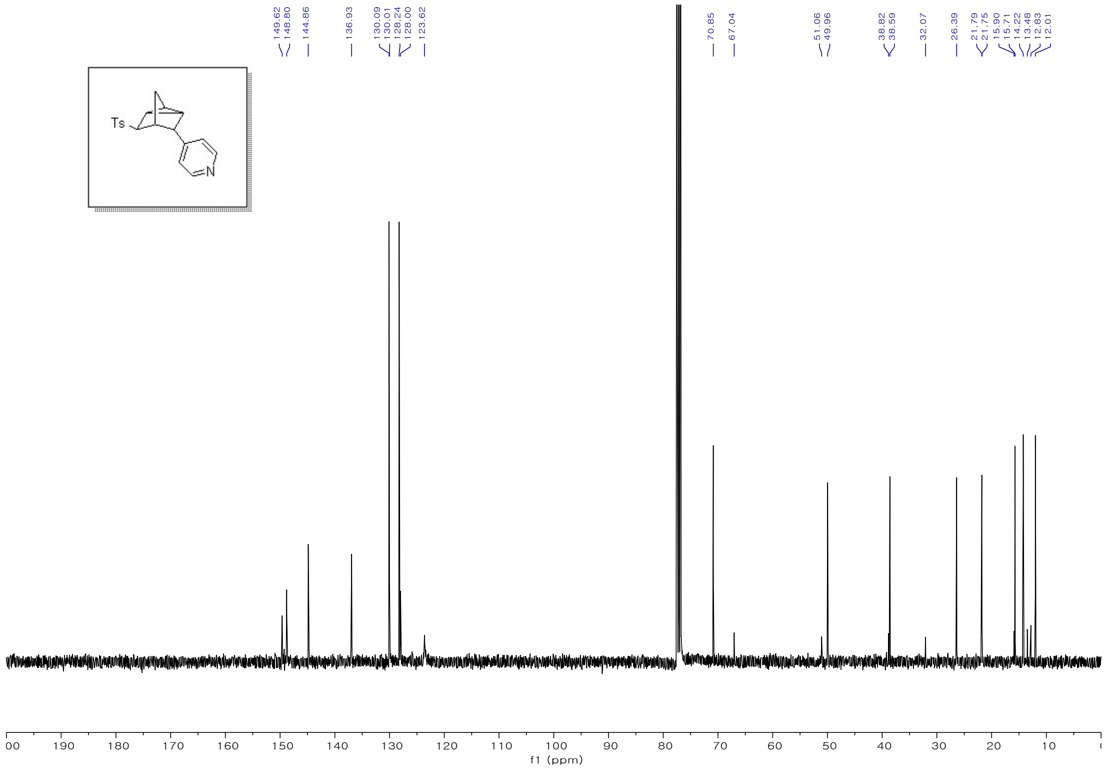

100 MHz, ^{13}C NMR in $CDCl_3$.

4-tosylpyridine (5a).

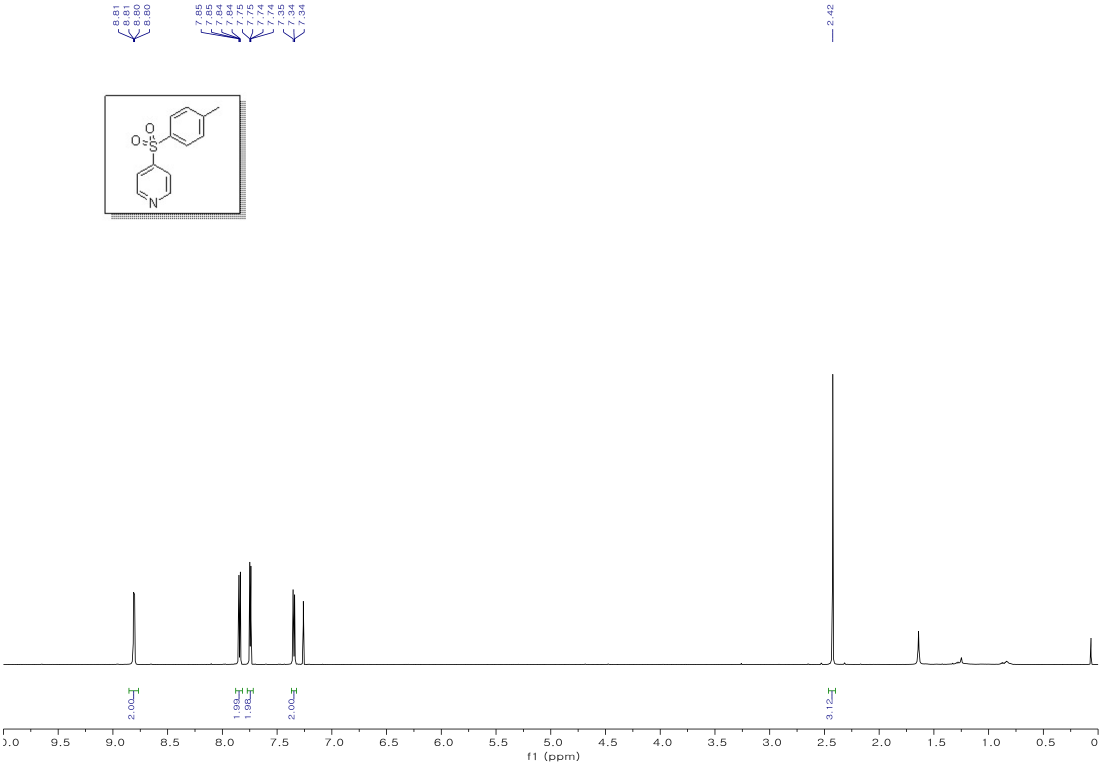

600 MHz, ^{1}H NMR in $CDCl_3$.

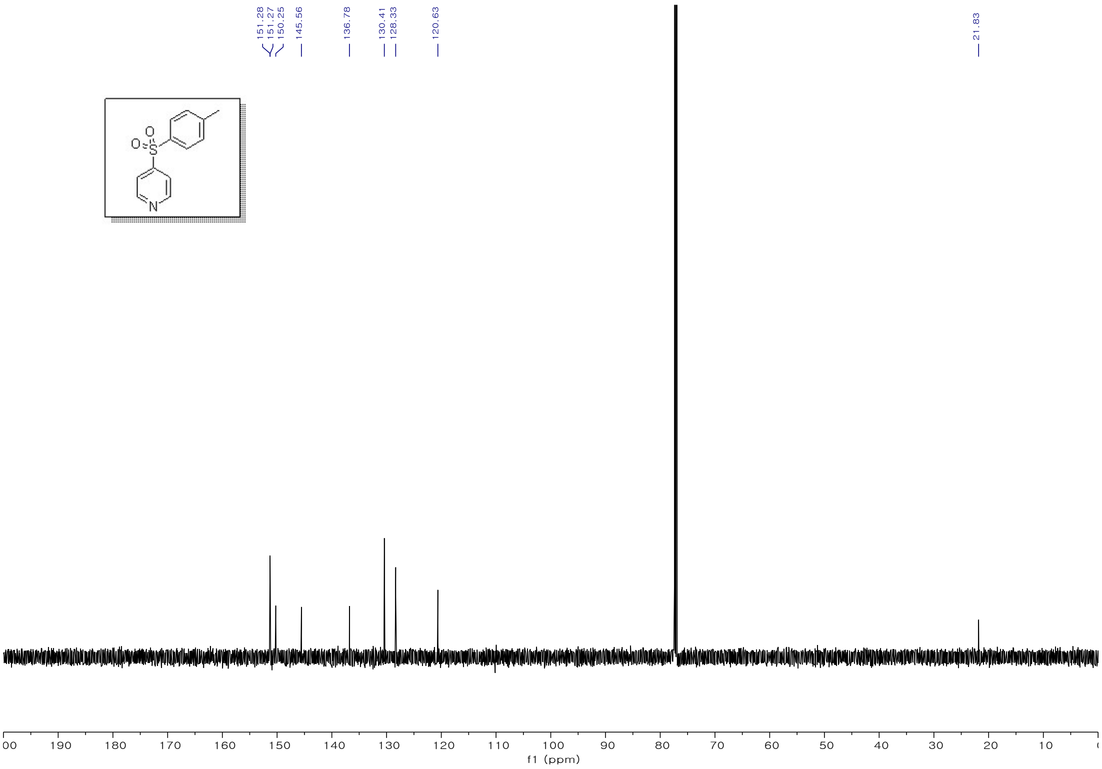

150 MHz, ^{13}C NMR in $CDCl_3$.

methyl 4-tosylpicolinate (5b).

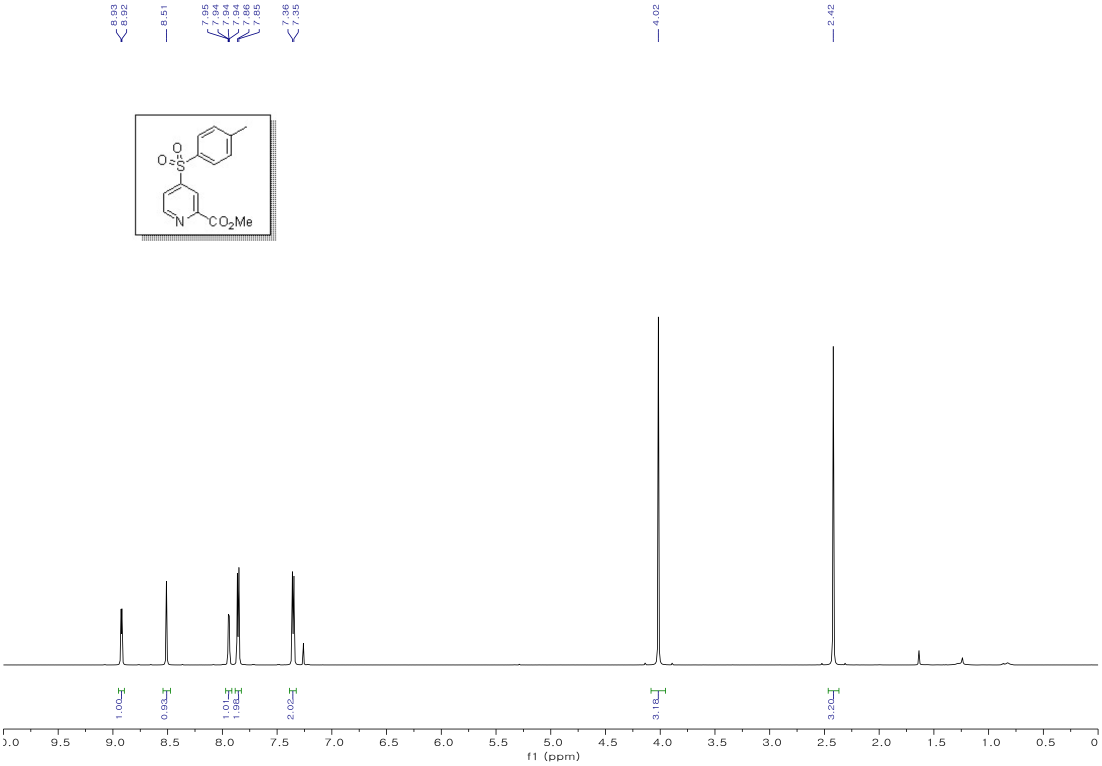

600 MHz, ^{1}H NMR in $CDCl_3$.

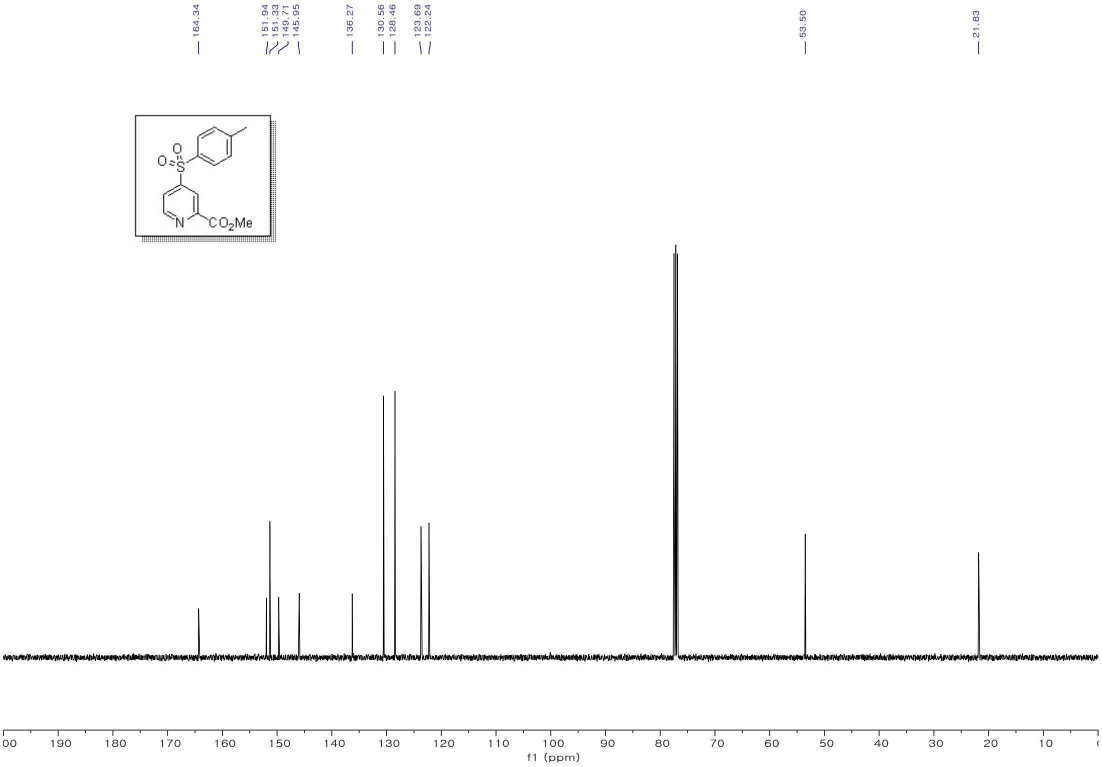

100 MHz, ^{13}C NMR in $CDCl_3$.

2-phenyl-4-tosylpyridine (5c).

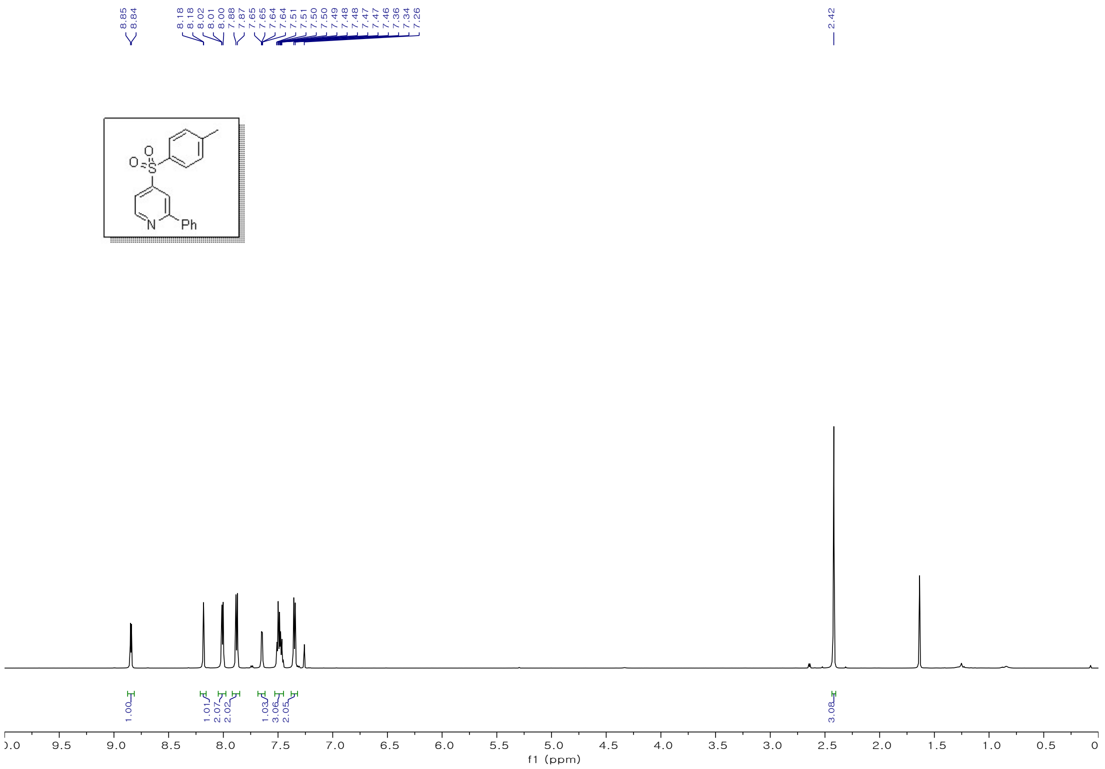

600 MHz, ^{1}H NMR in $CDCl_3$.

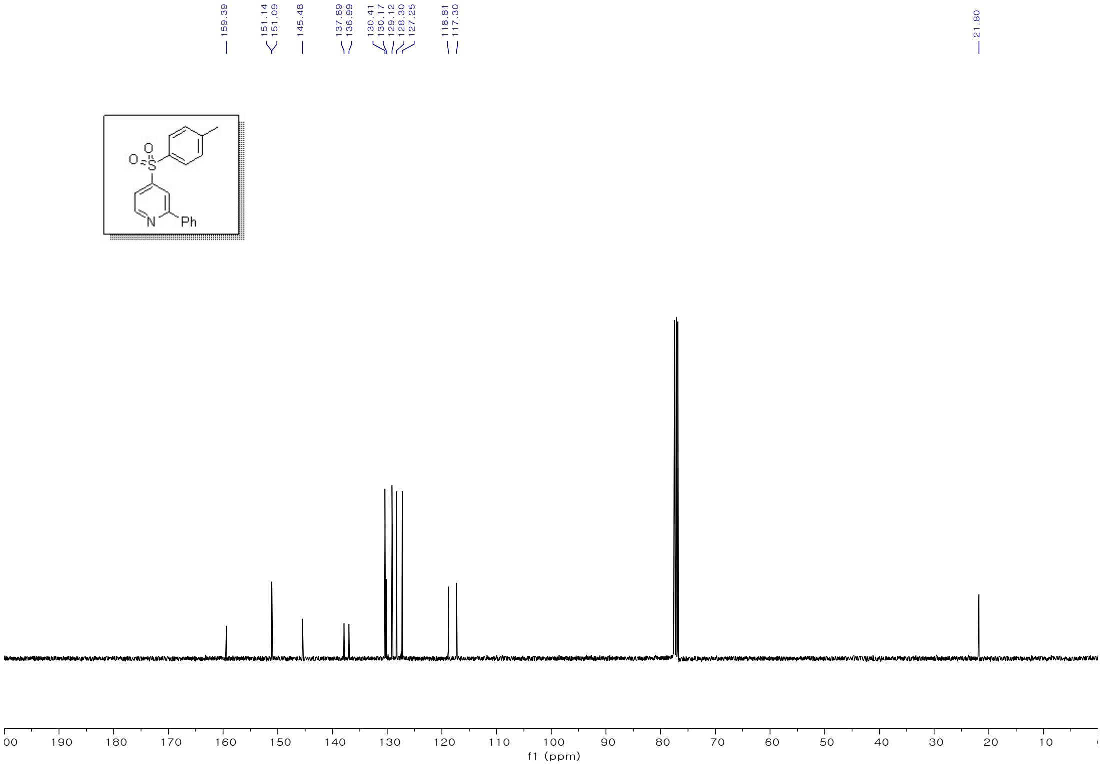

100 MHz, ^{13}C NMR in $CDCl_3$.

2-(4-bromophenyl)-4-tosylpyridine (5d).

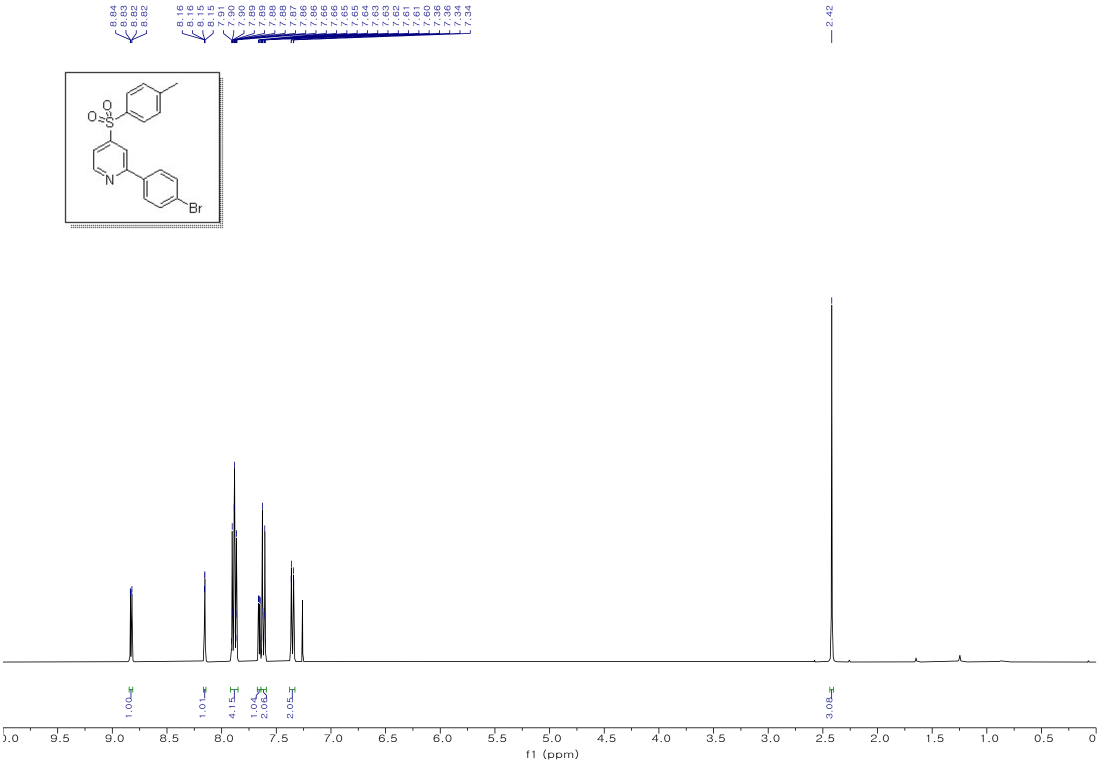

400 MHz, ^{1}H NMR in $CDCl_3$.

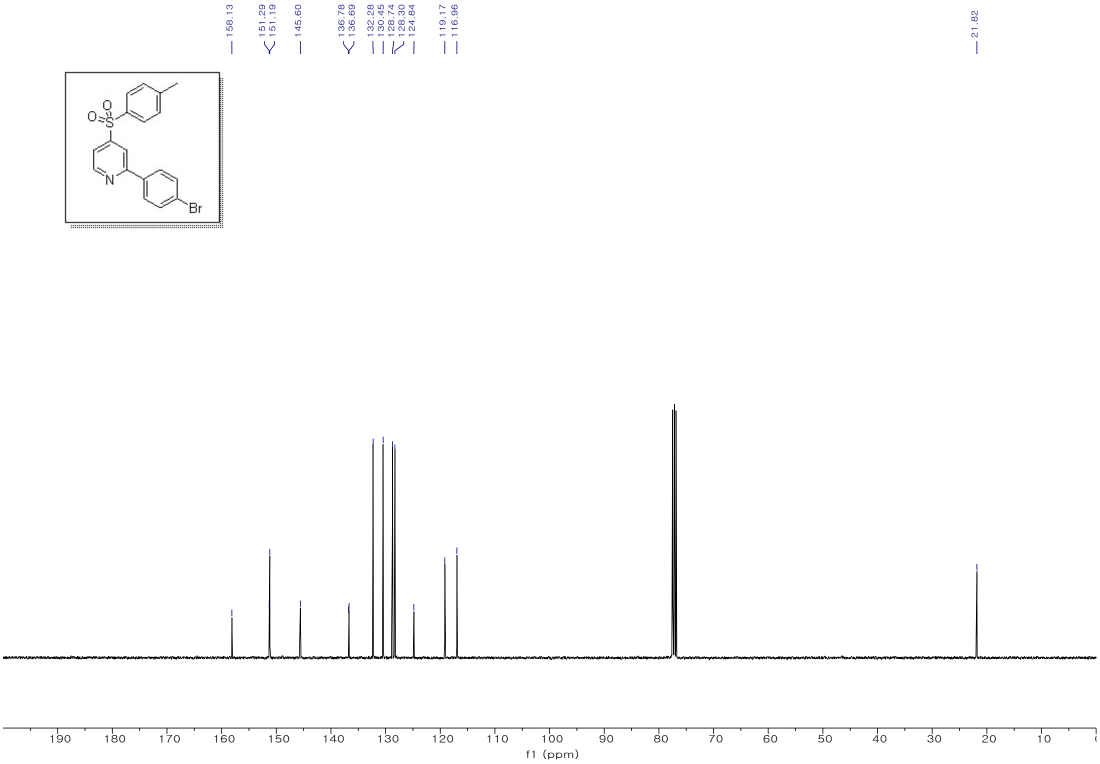

100 MHz, ^{13}C NMR in $CDCl_3$.

4-tosyl-2-(4-(trifluoromethyl)phenyl)pyridine (5e).

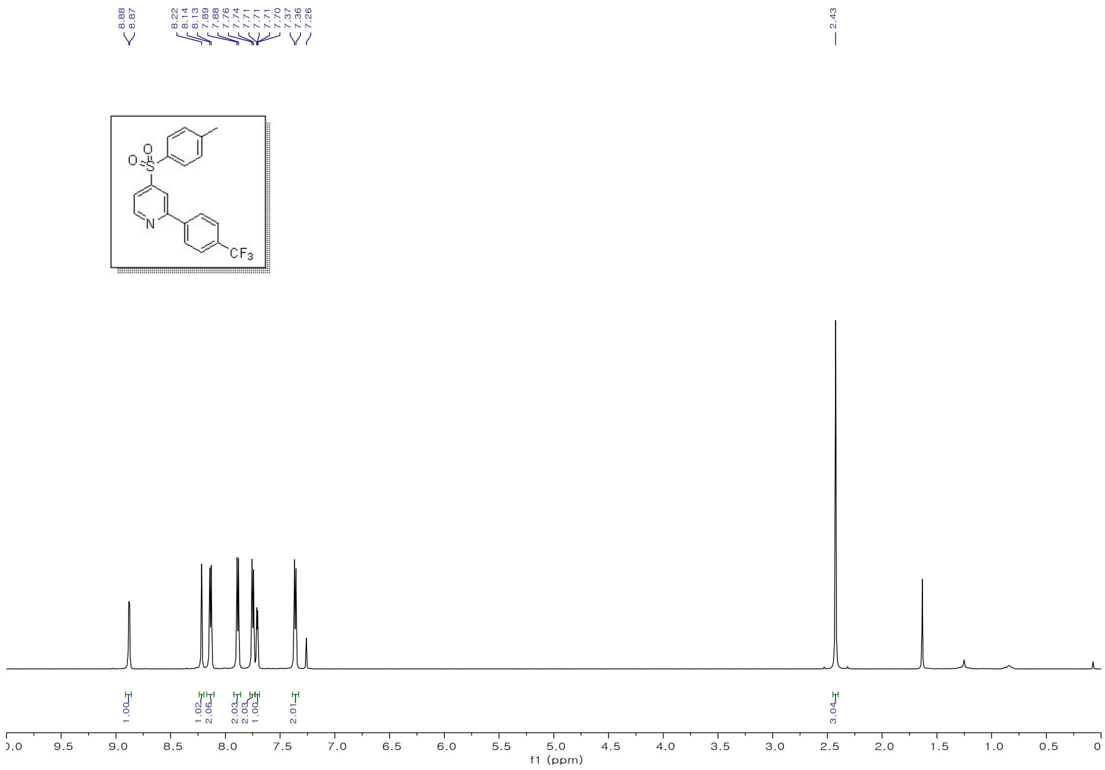

600 MHz, ^{1}H NMR in $CDCl_3$.

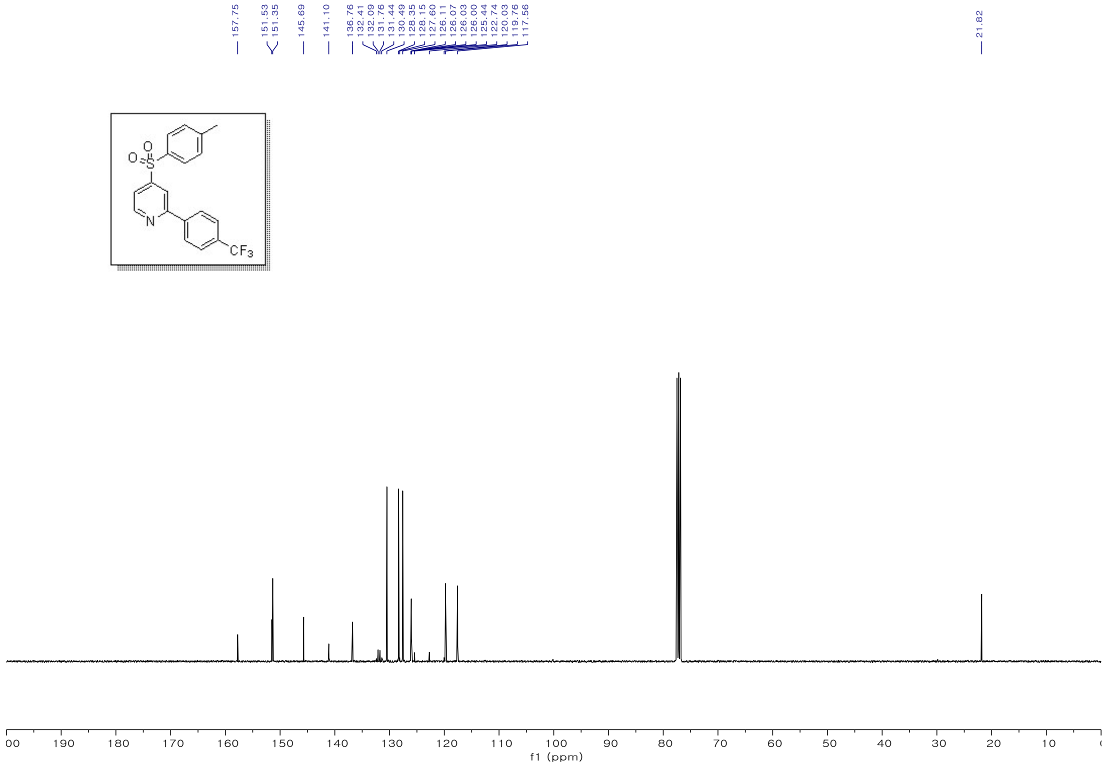

100 MHz, ^{13}C NMR in $CDCl_3$.

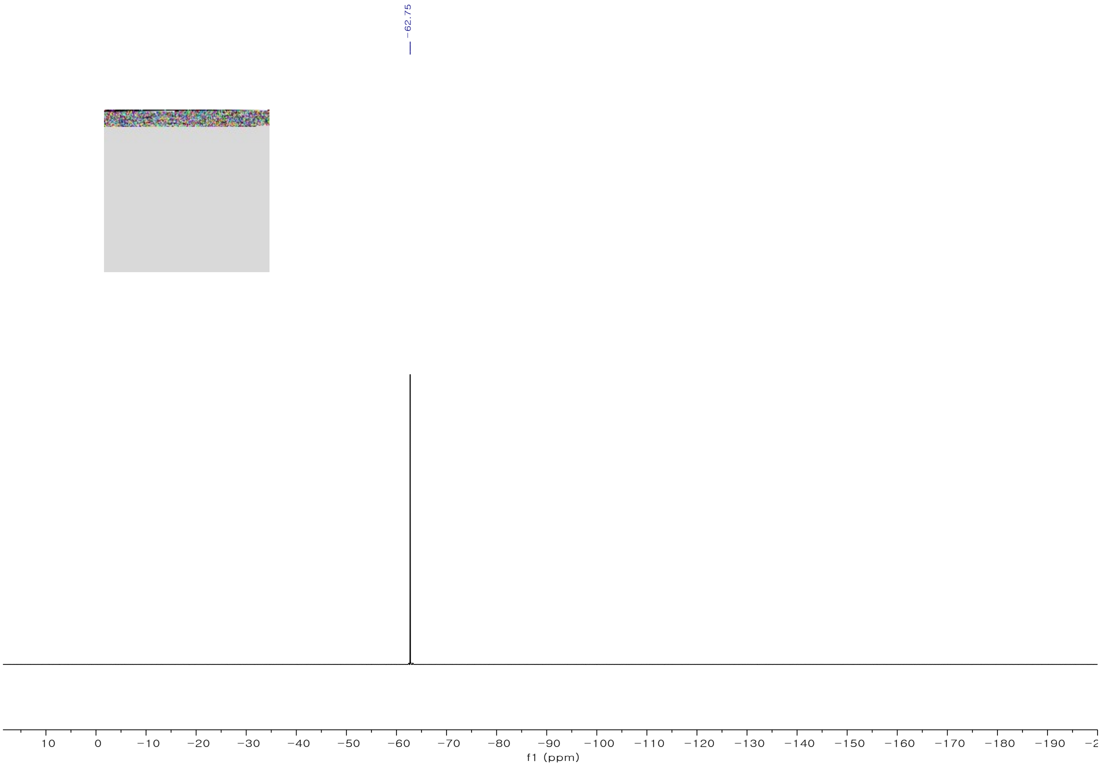

376 MHz, ^{19}F NMR in $CDCl_3$.

3-chloro-4-tosylpyridine (5f).

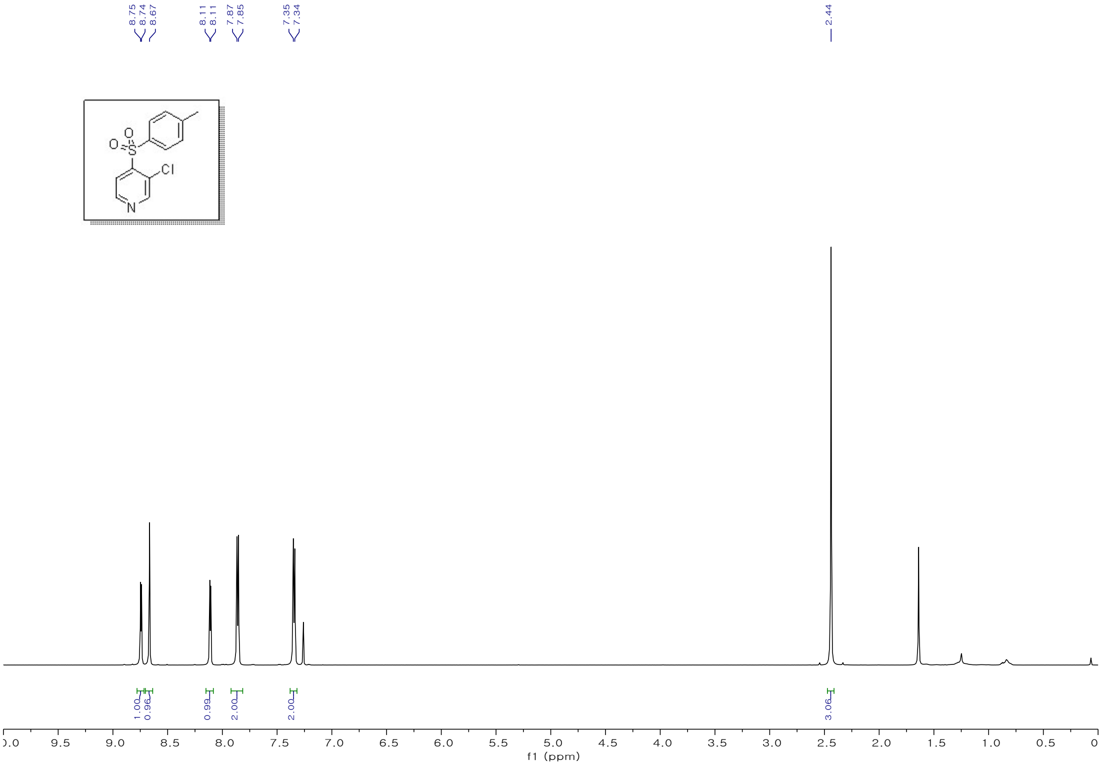

600 MHz, ^{1}H NMR in $CDCl_3$.

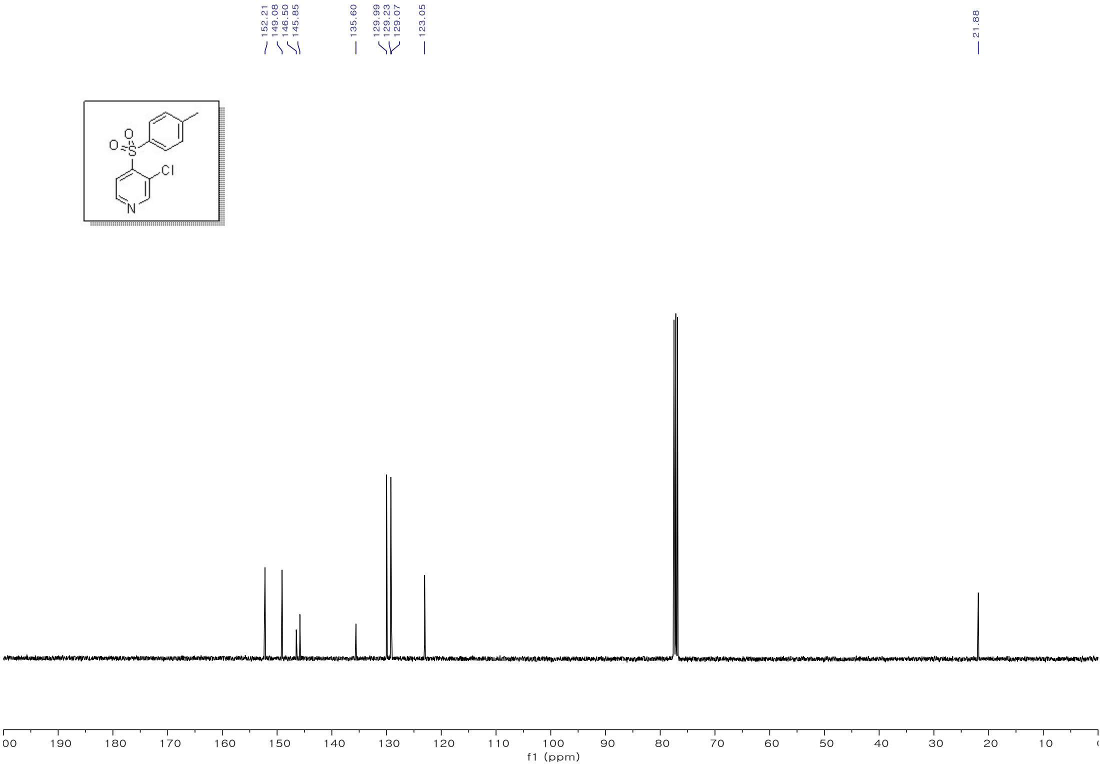

100 MHz, ^{13}C NMR in $CDCl_3$.

3-bromo-4-tosylpyridine (5g).

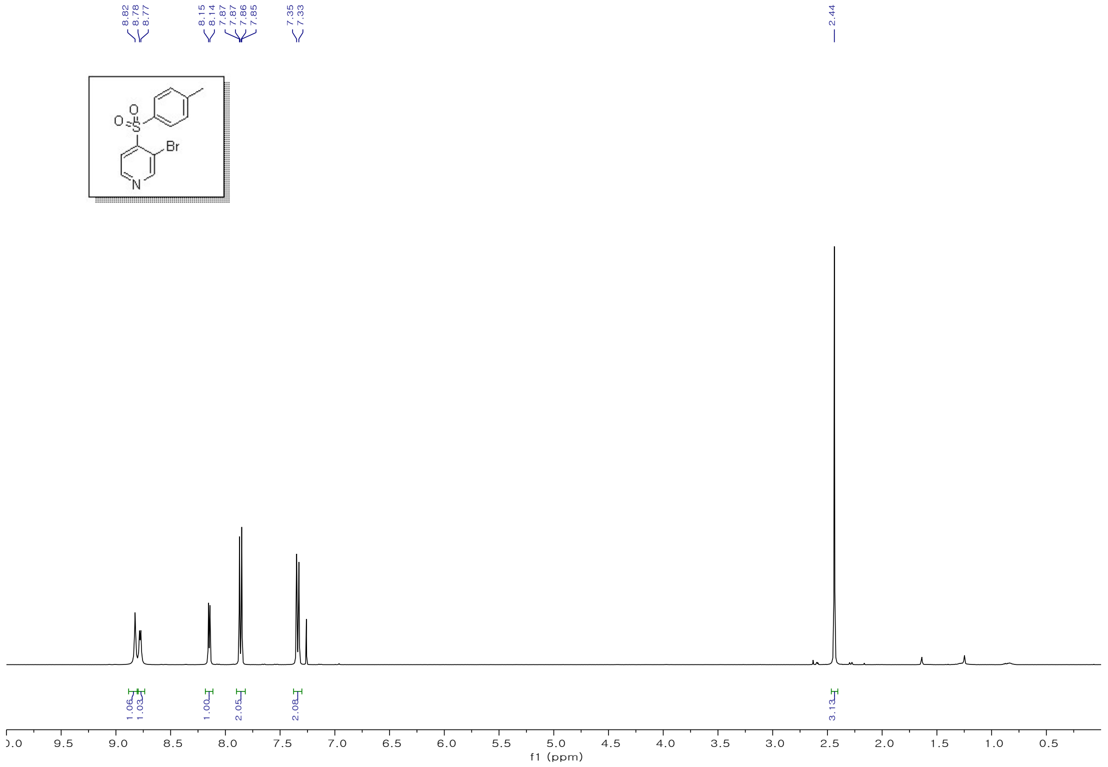

400 MHz, ^{1}H NMR in $CDCl_3$.

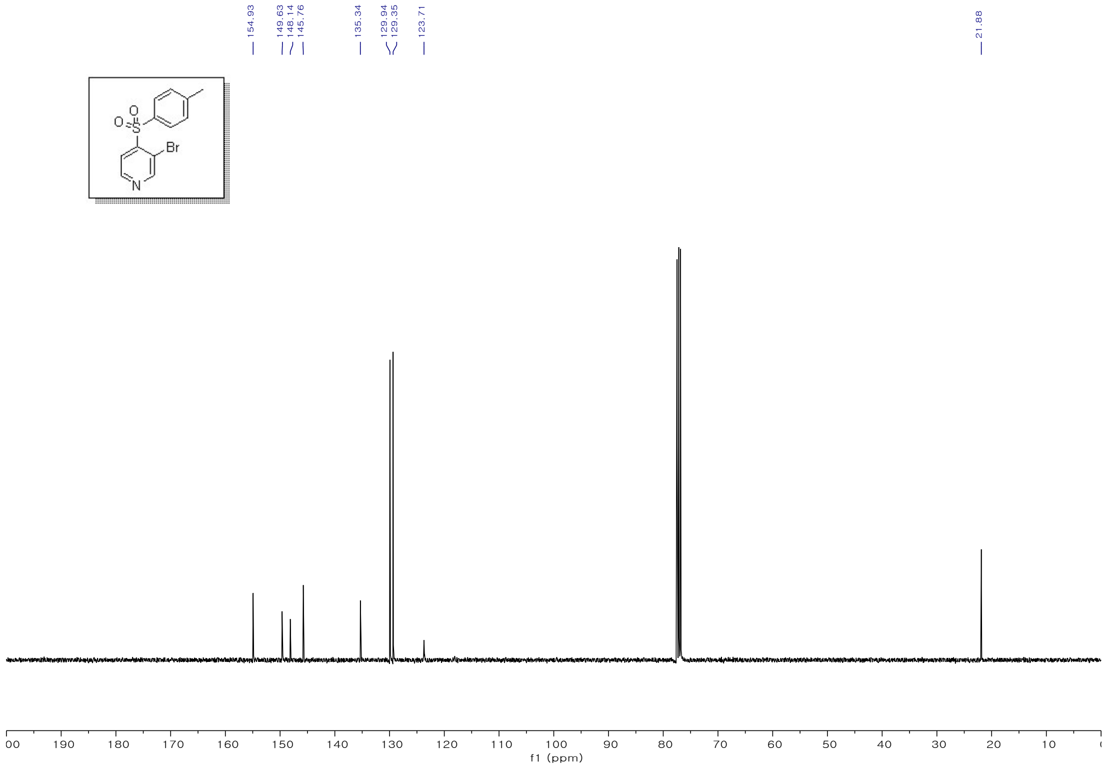

100 MHz, ^{13}C NMR in $CDCl_3$.

4-tosylquinoline (5h).

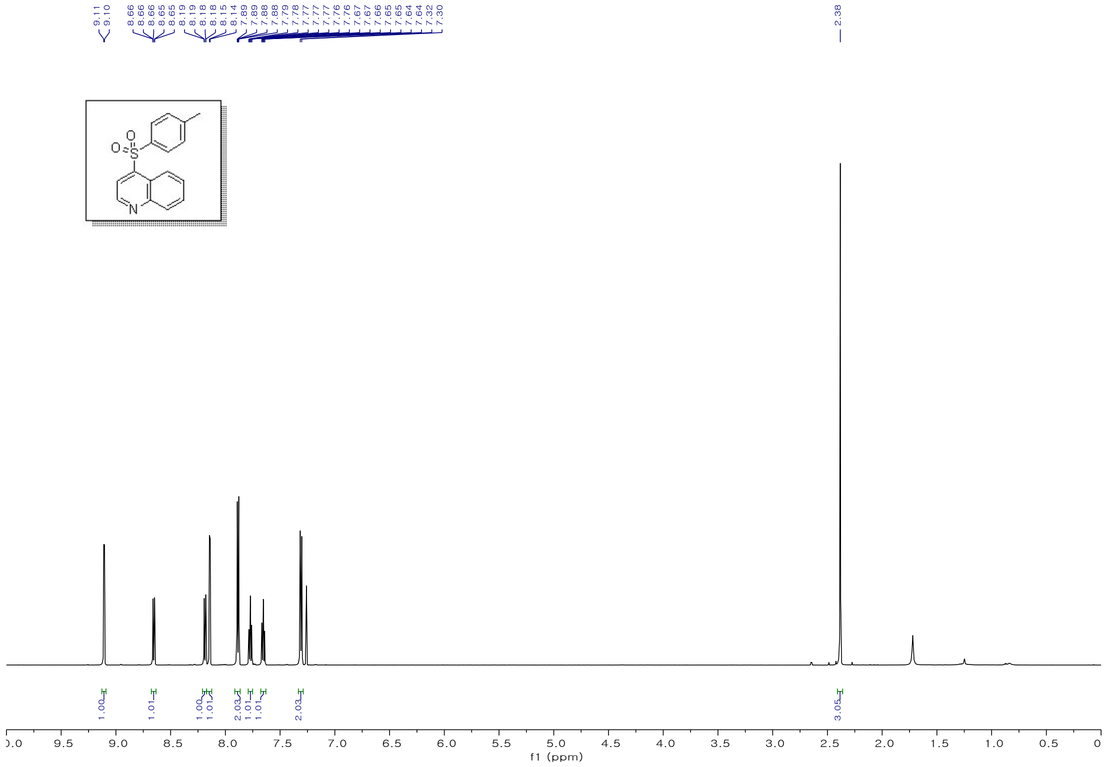

600 MHz, ^{1}H NMR in $CDCl_3$.

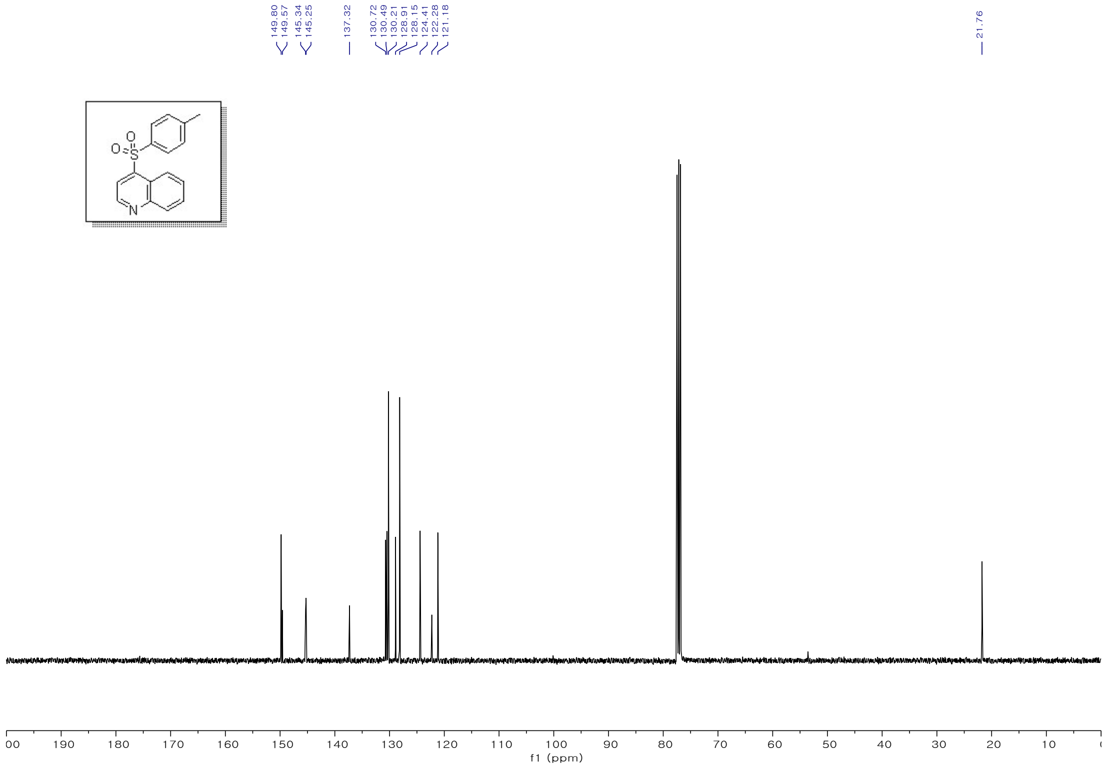

100 MHz, ^{13}C NMR in $CDCl_3$.

6-methoxy-4-tosylquinoline (5i).

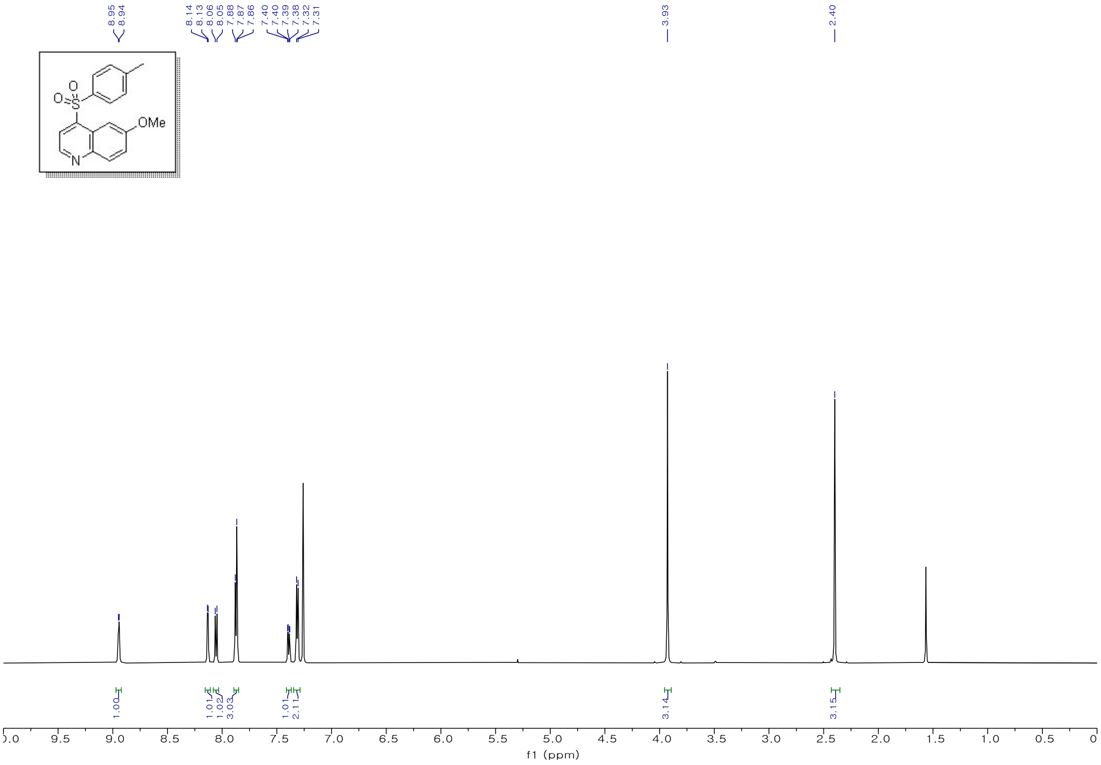

600 MHz, ^{1}H NMR in $CDCl_3$.

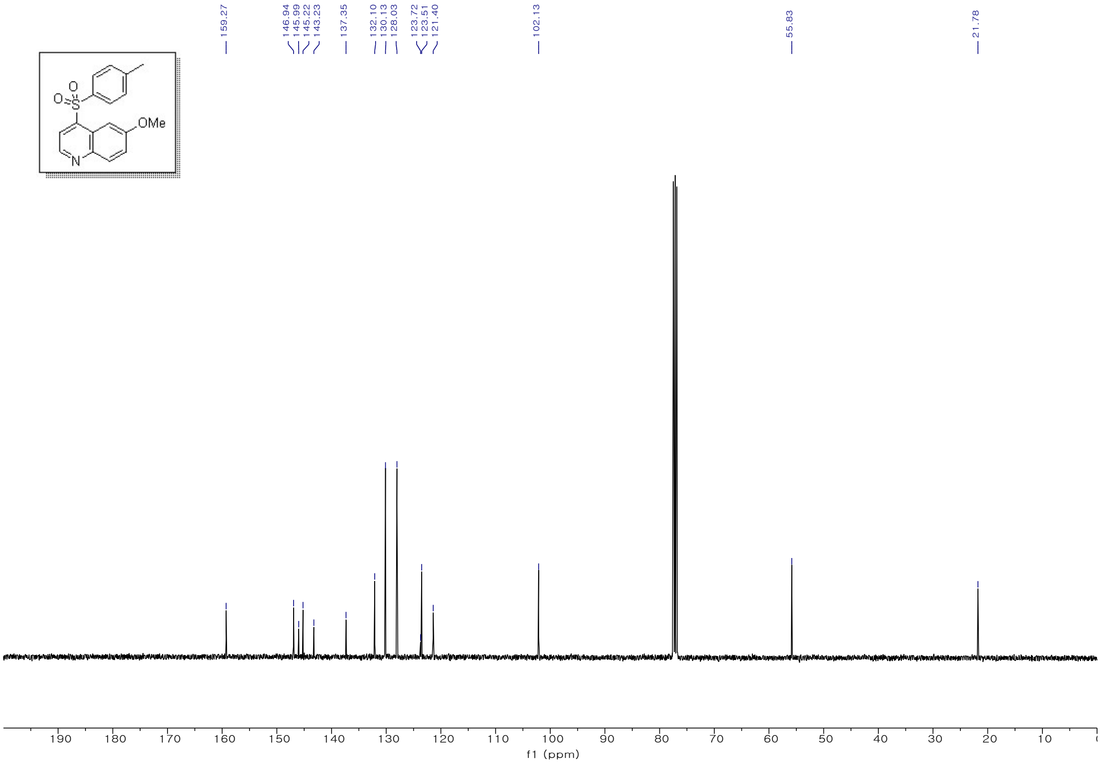

100 MHz, ^{13}C NMR in $CDCl_3$.

2-phenyl-4-tosylquinoline (5j).

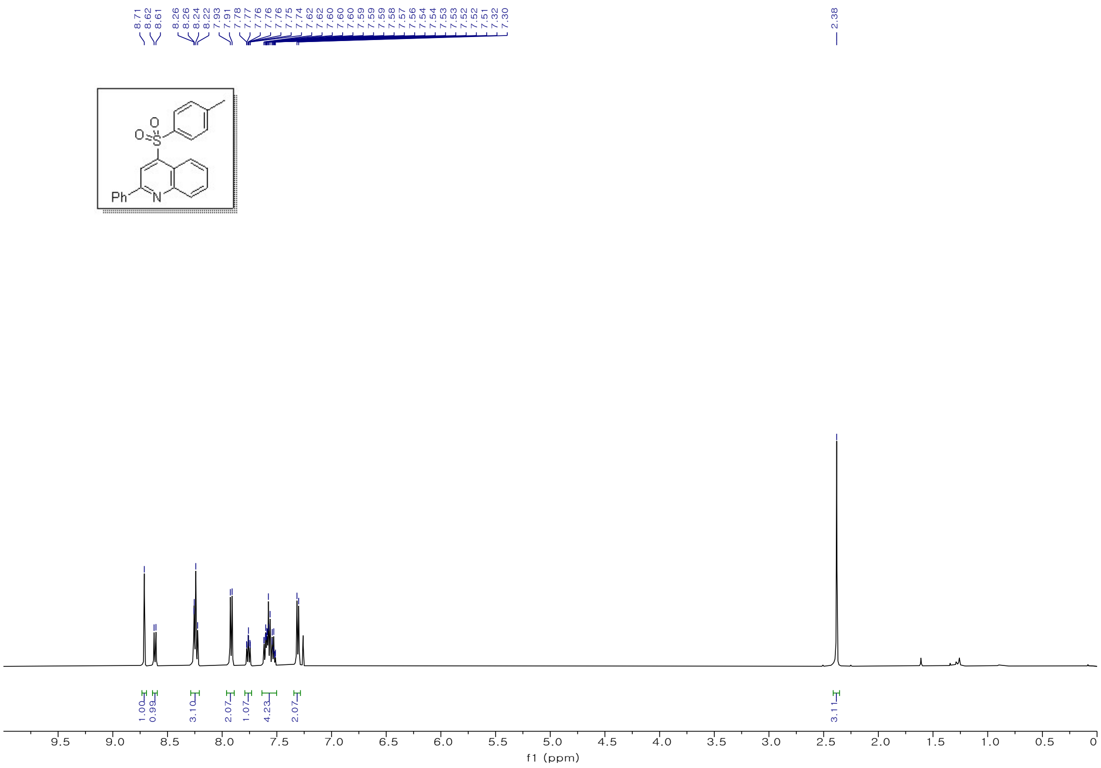

600 MHz, ^{1}H NMR in $CDCl_3$.

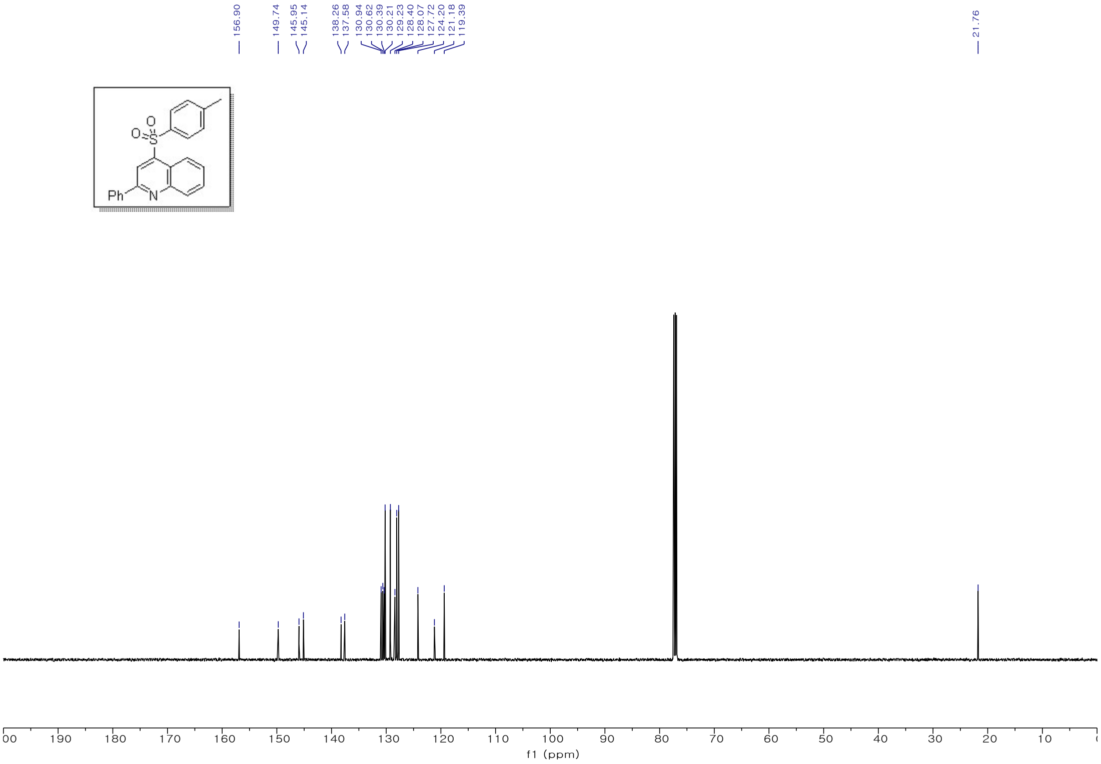

100 MHz, ^{13}C NMR in $CDCl_3$

4-((4-methoxyphenyl)sulfonyl)-2-phenylpyridine4 (5k).

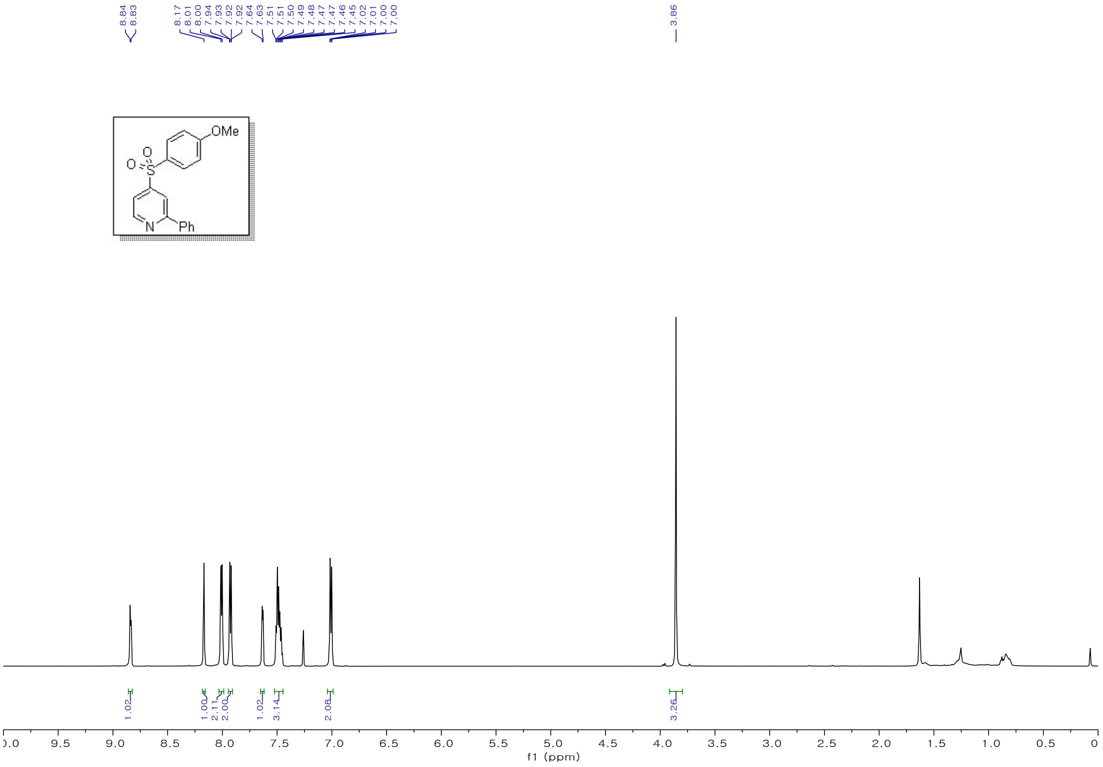

600 MHz, ^{1}H NMR in $CDCl_3$.

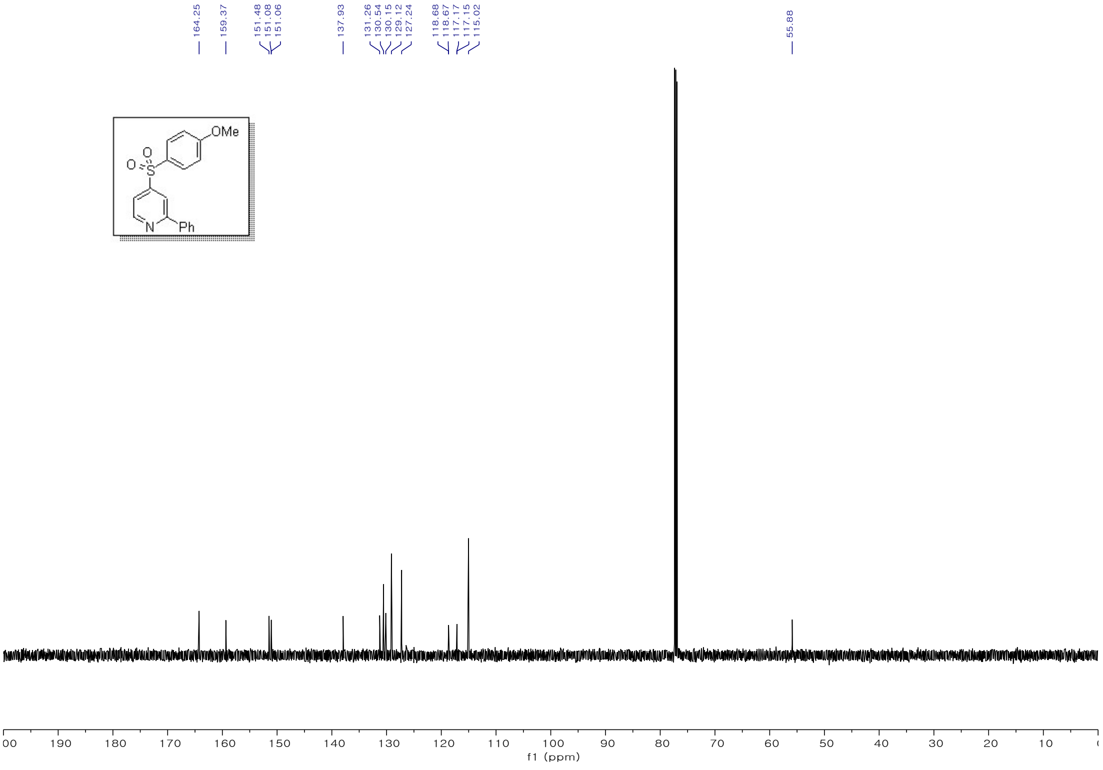

150 MHz, ^{13}C NMR in $CDCl_3$.

-((4-fluorophenyl)sulfonyl)-2-phenylpyridine (5l).

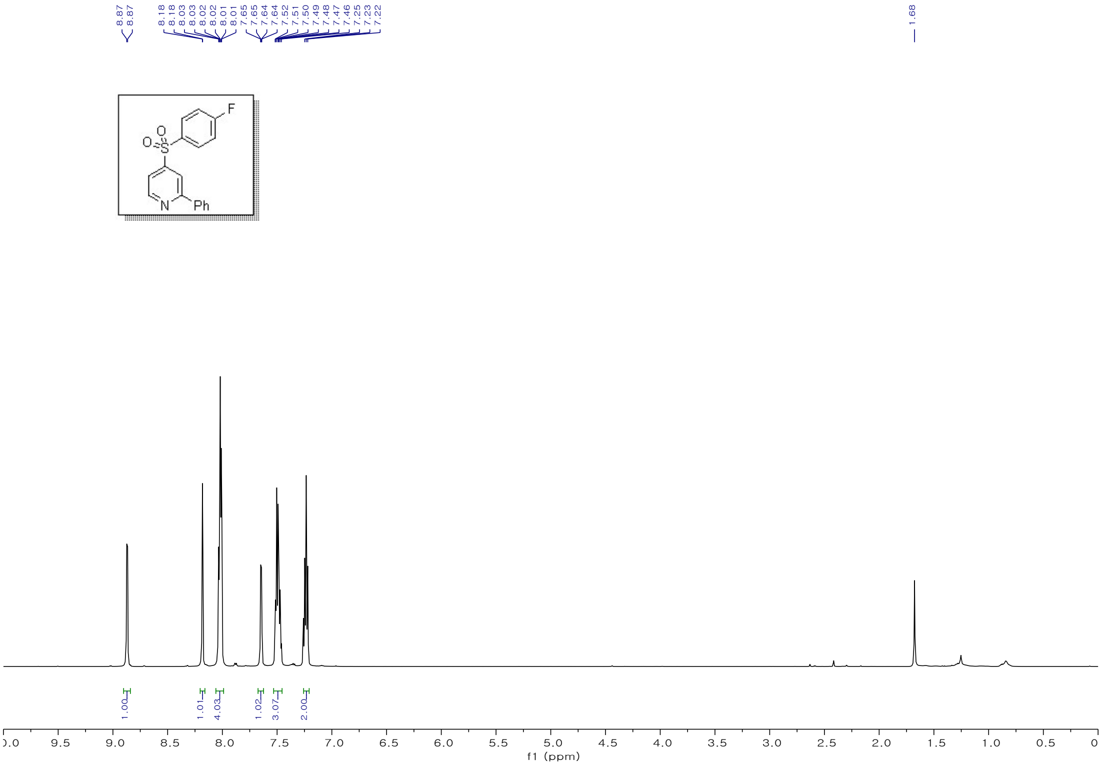

600 MHz, ^{1}H NMR in $CDCl_3$.

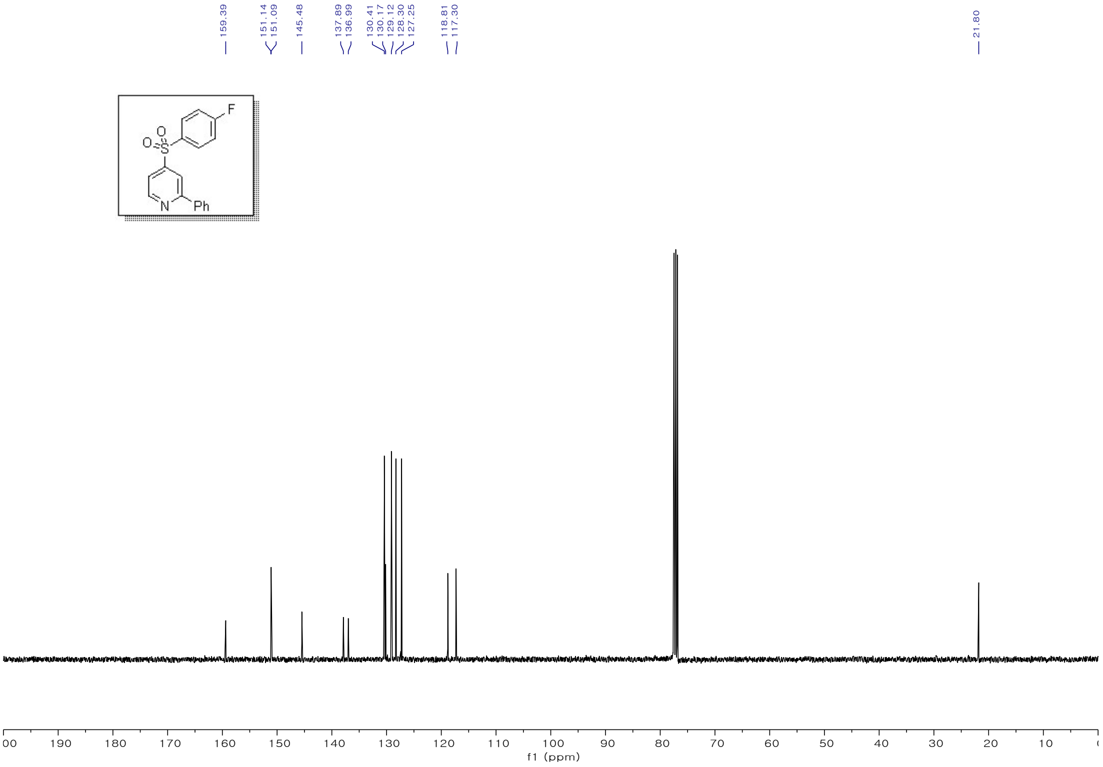

100 MHz, ^{13}C NMR in $CDCl_3$.

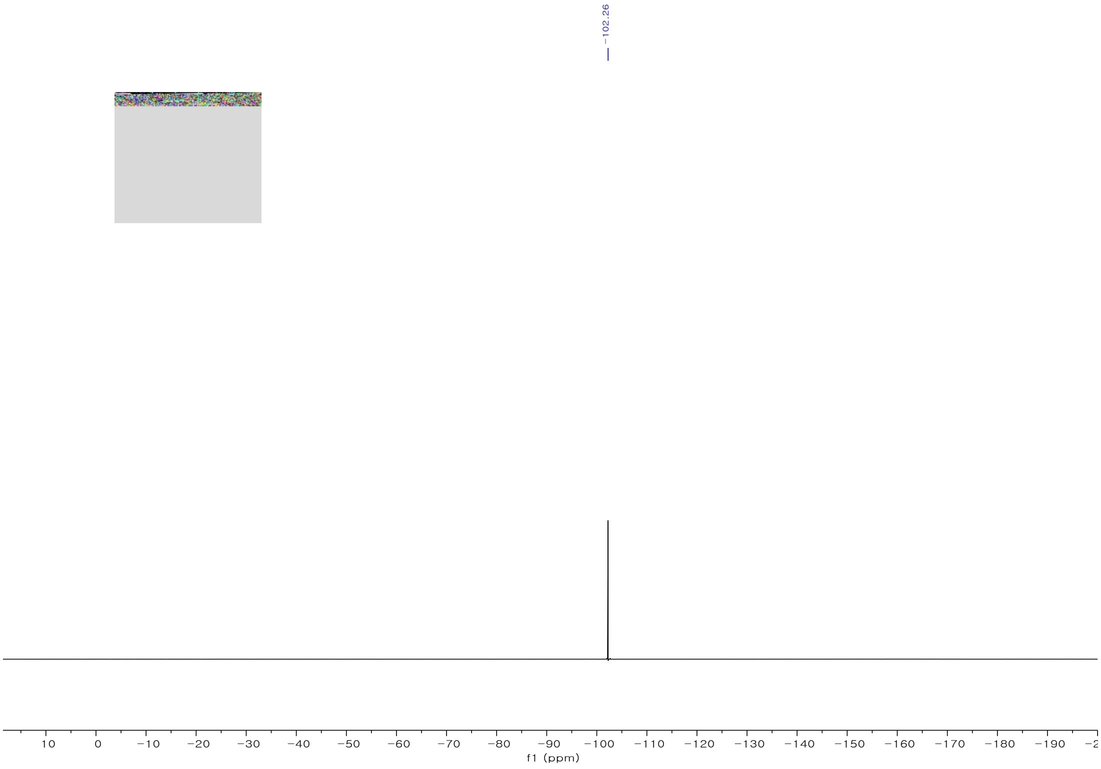

376 MHz, ^{19}F NMR in $CDCl_3$.

4-((4-nitrophenyl)sulfonyl)-2-phenylpyridine (5m).

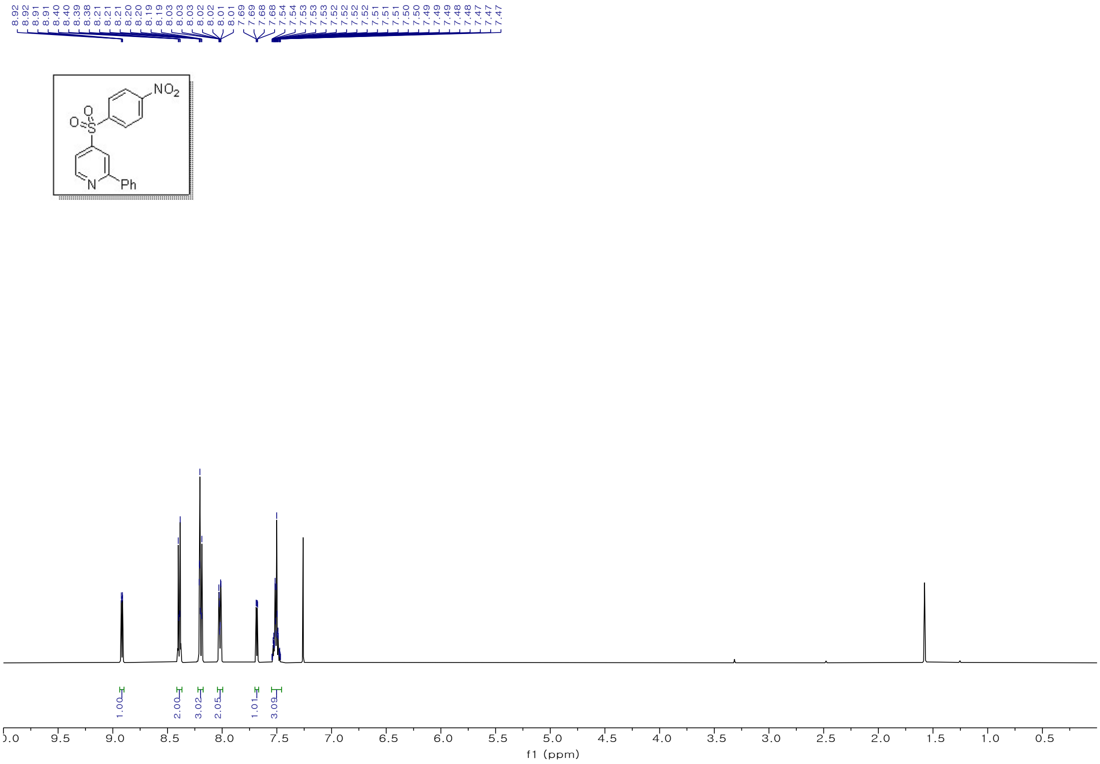

500 MHz, ^{1}H NMR in $CDCl_3$.

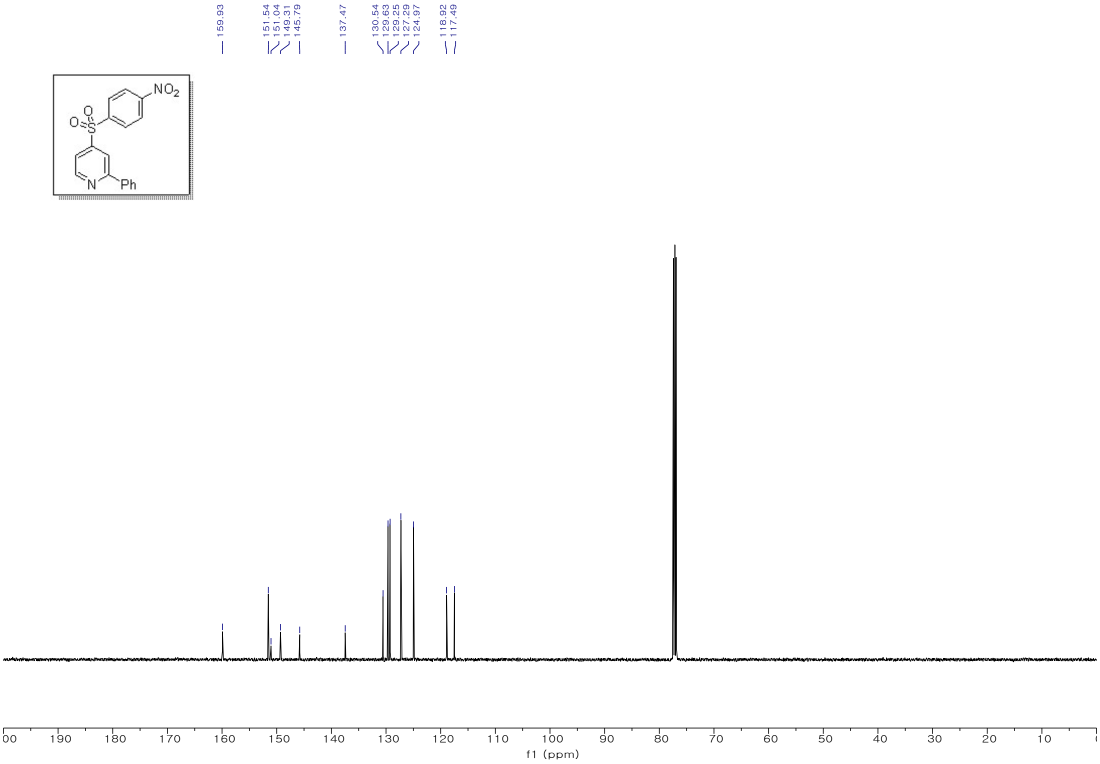

125 MHz, ^{13}C NMR in $CDCl_3$.

4-(naphthalen-2-ylsulfonyl)-2-phenylpyridine (5n).

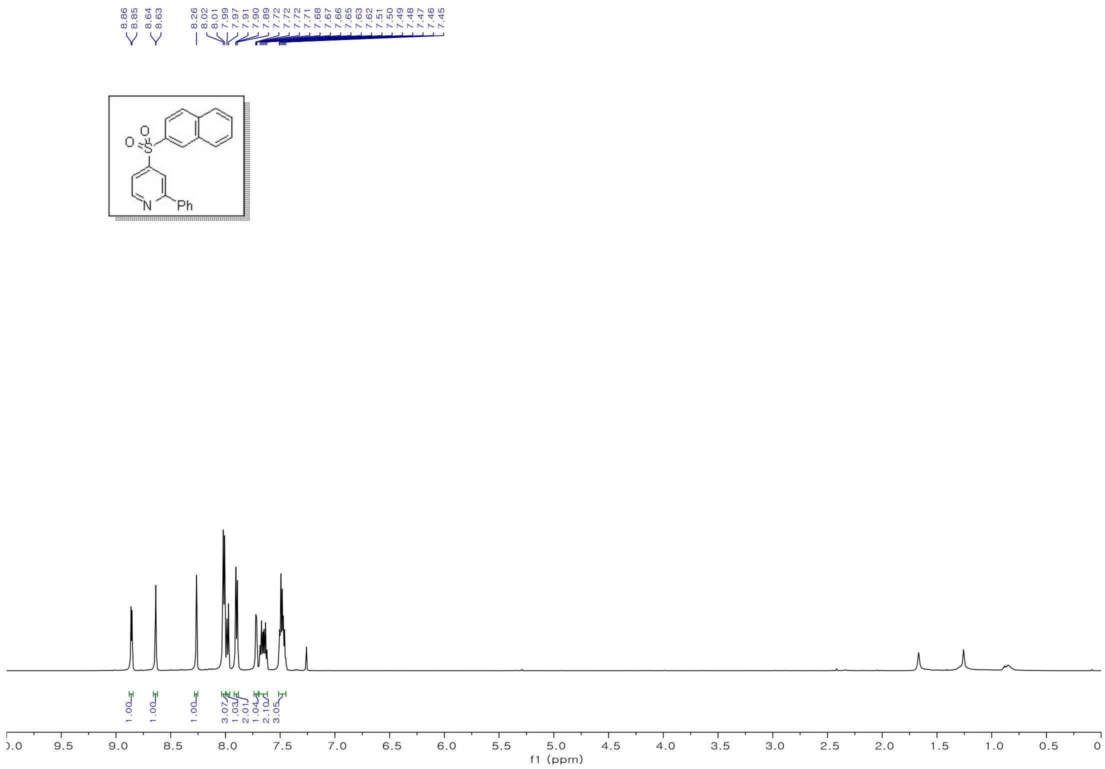

600 MHz, ^{1}H NMR in $CDCl_3$.

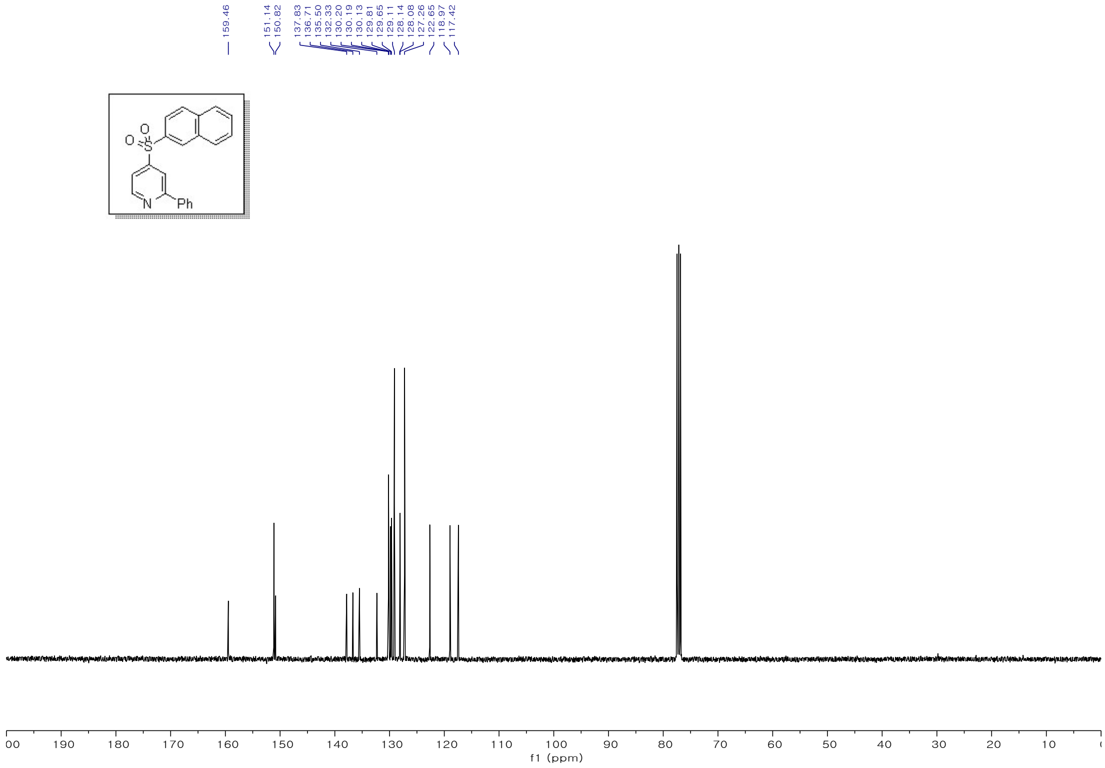

100 MHz, ^{13}C NMR in $CDCl_3$.

2-phenyl-4-(thiophen-2-ylsulfonyl)pyridine (5o).

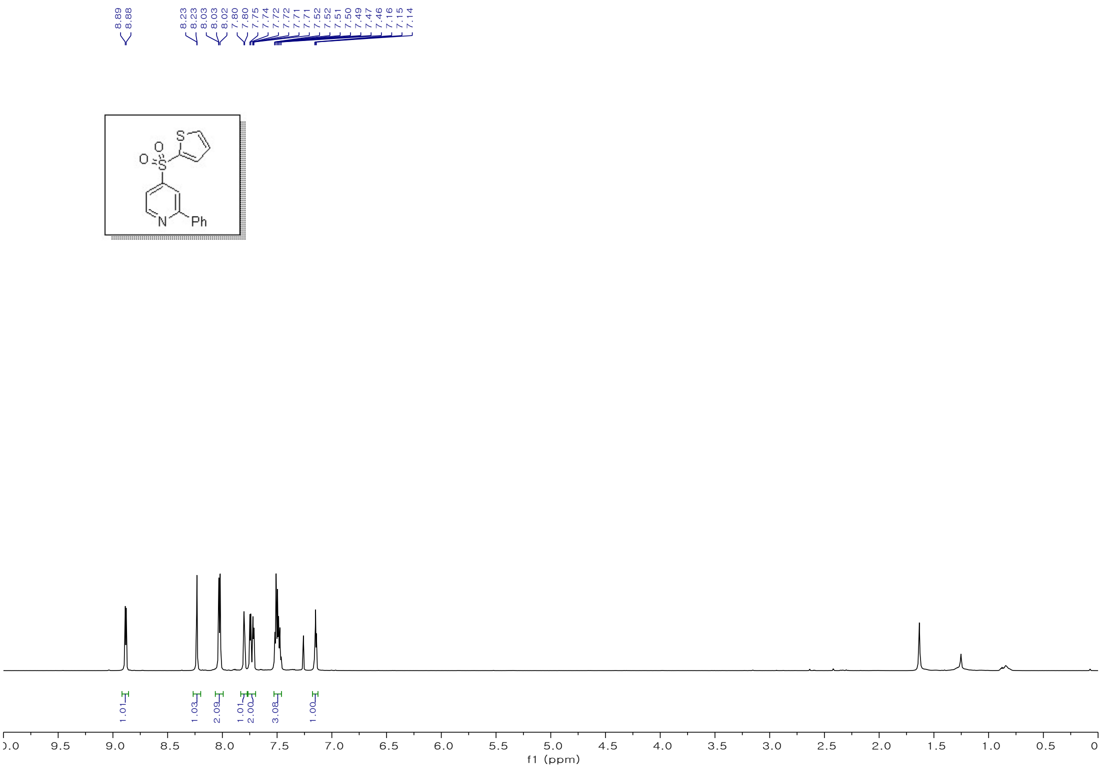

600 MHz, ^{1}H NMR in $CDCl_3$.

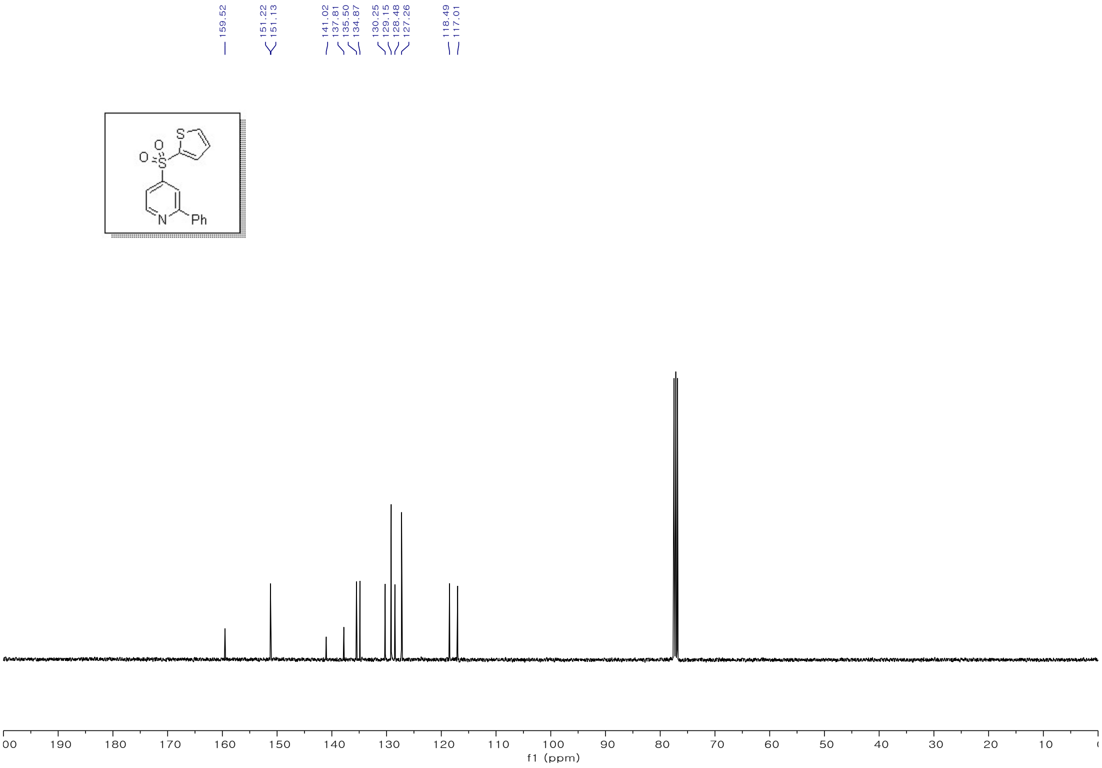

100 MHz, ^{13}C NMR in $CDCl_3$.

4-(methylsulfonyl)-2-phenylpyridine (5p)

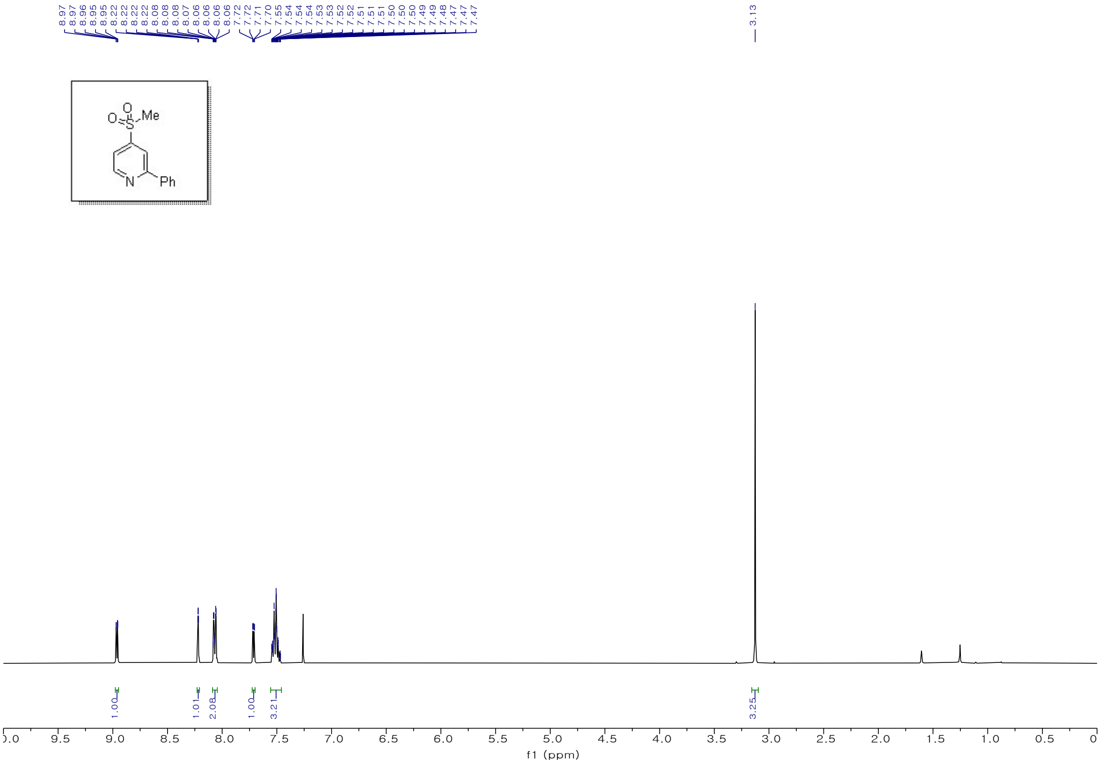

400 MHz, ^{1}H NMR in $CDCl_3$.

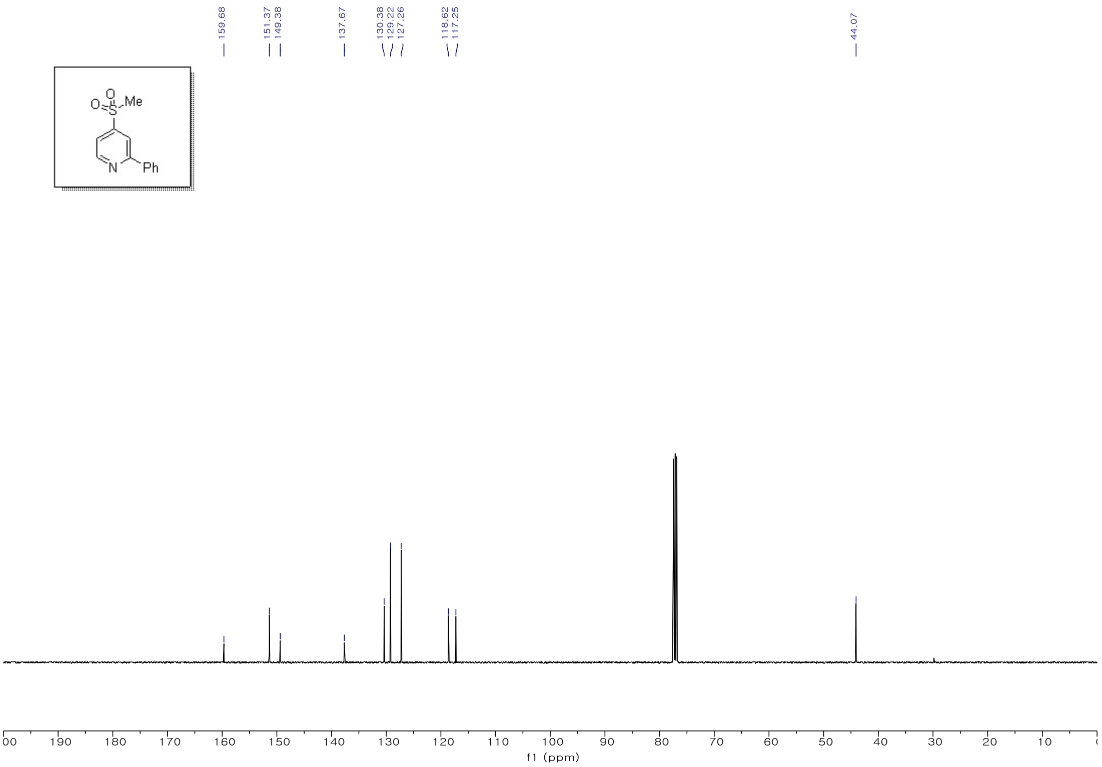

100 MHz, ^{13}C NMR in $CDCl_3$

4-(butylsulfonyl)-2-phenylpyridine (5q).

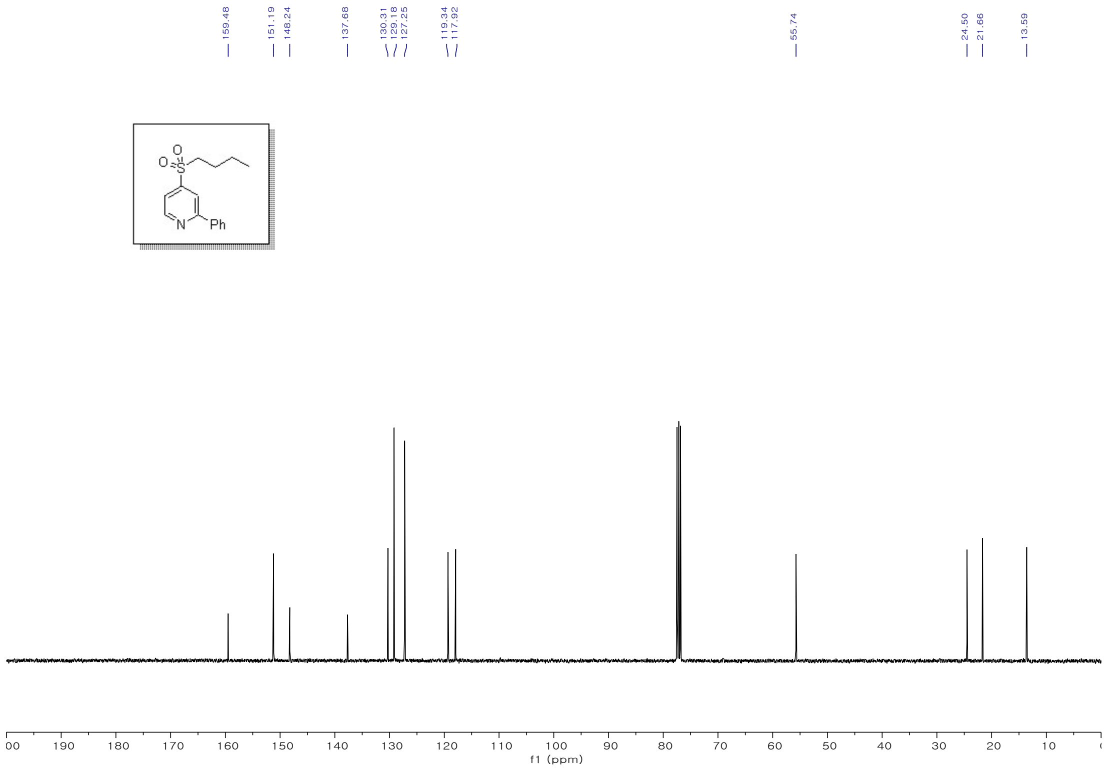

400 MHz, ^{1}H NMR in $CDCl_3$.

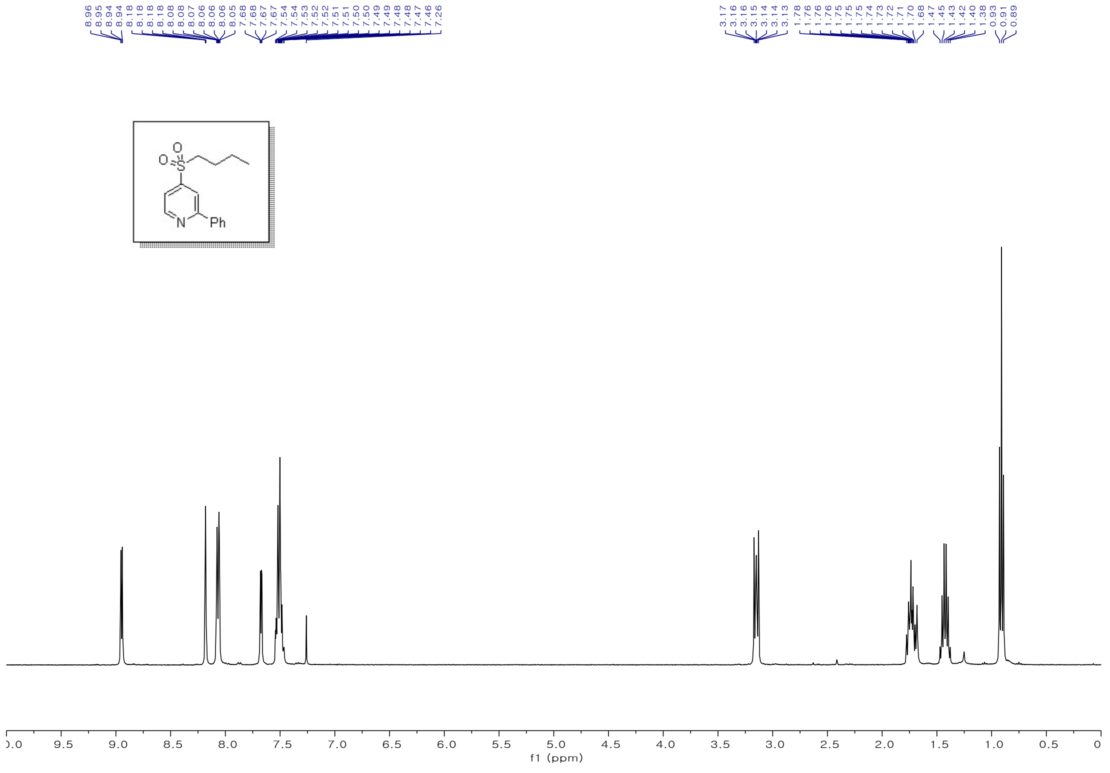

100 MHz, ^{13}C NMR in $CDCl_3$.

4-(phenethylsulfonyl)pyridine (5r)

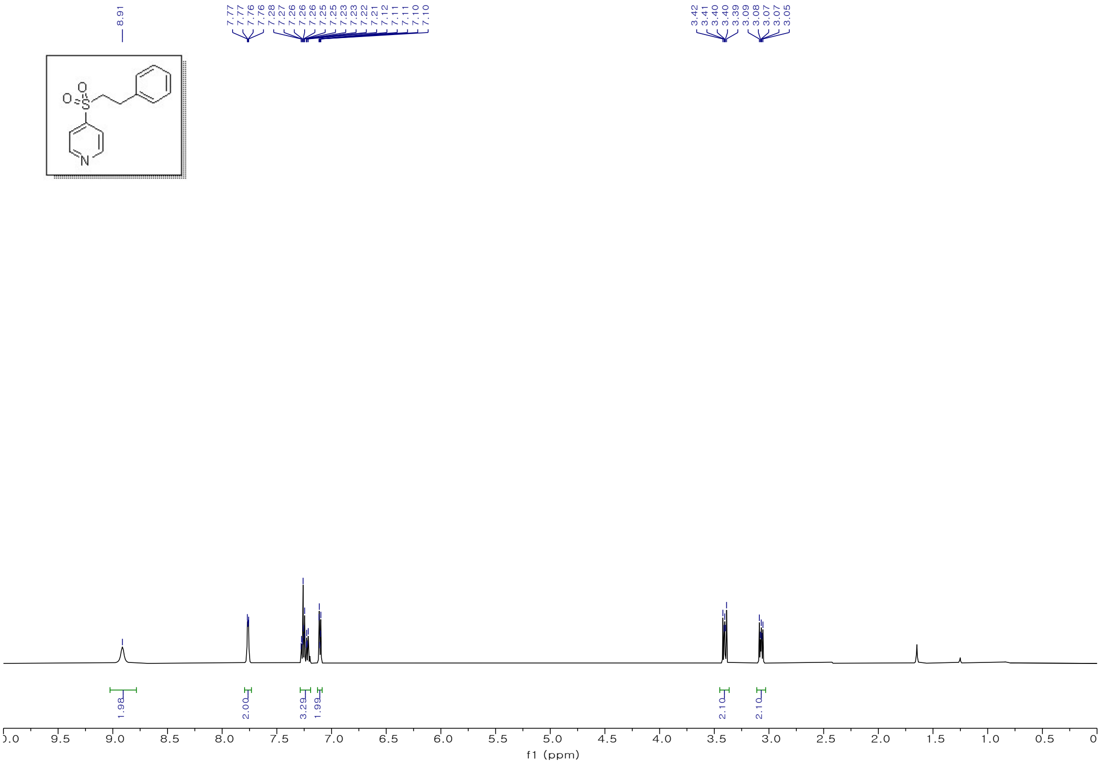

500 MHz, ^{1}H NMR in $CDCl_3$.

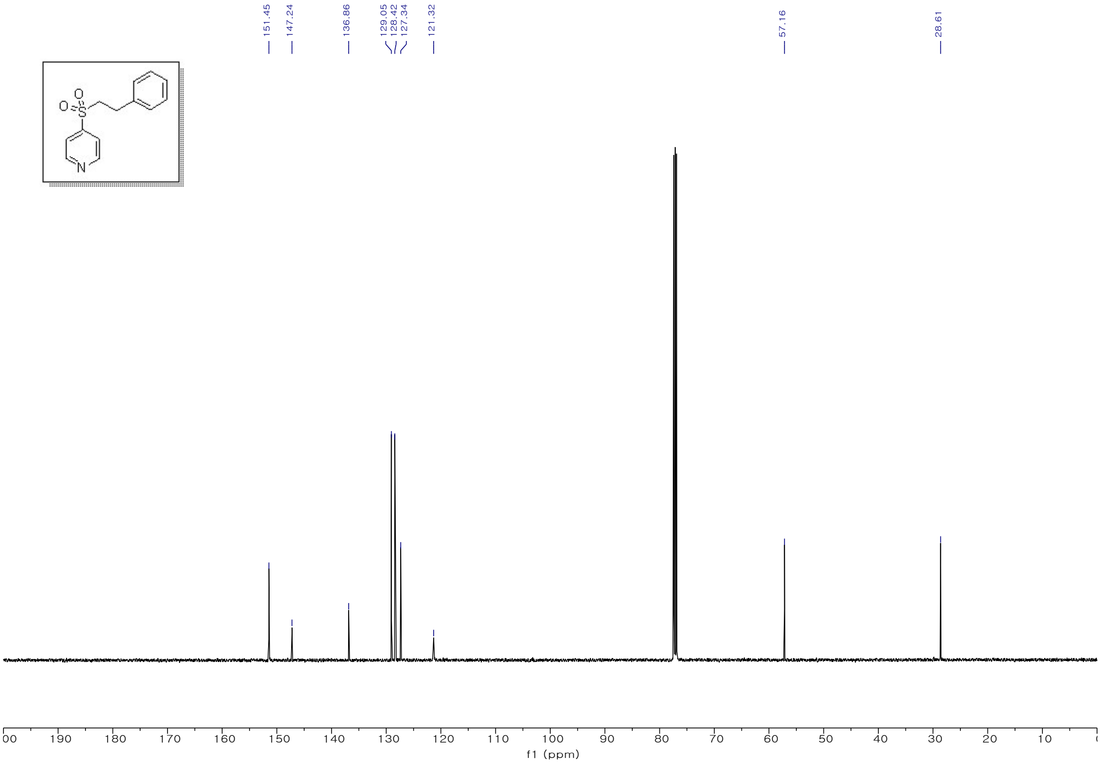

125 MHz, ^{13}C NMR in $CDCl_3$

2-phenyl-4-((tetrahydrofuran-3-yl)sulfonyl)pyridine (5s).

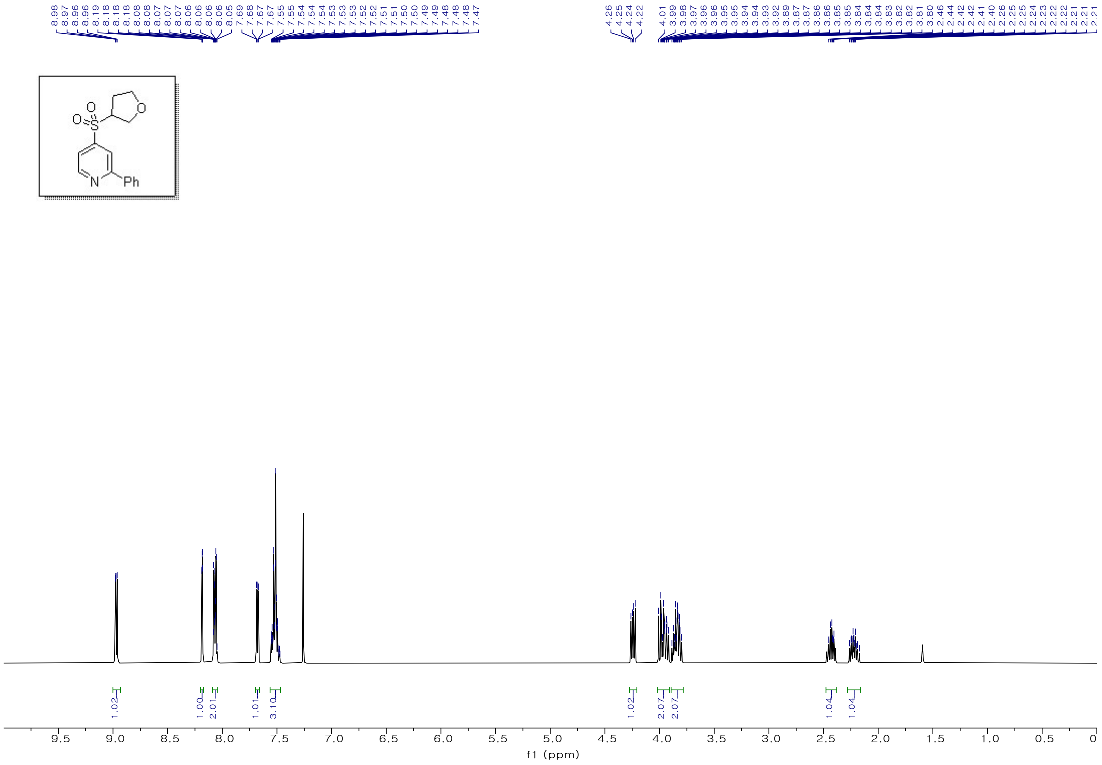

400 MHz, ^{1}H NMR in $CDCl_3$.

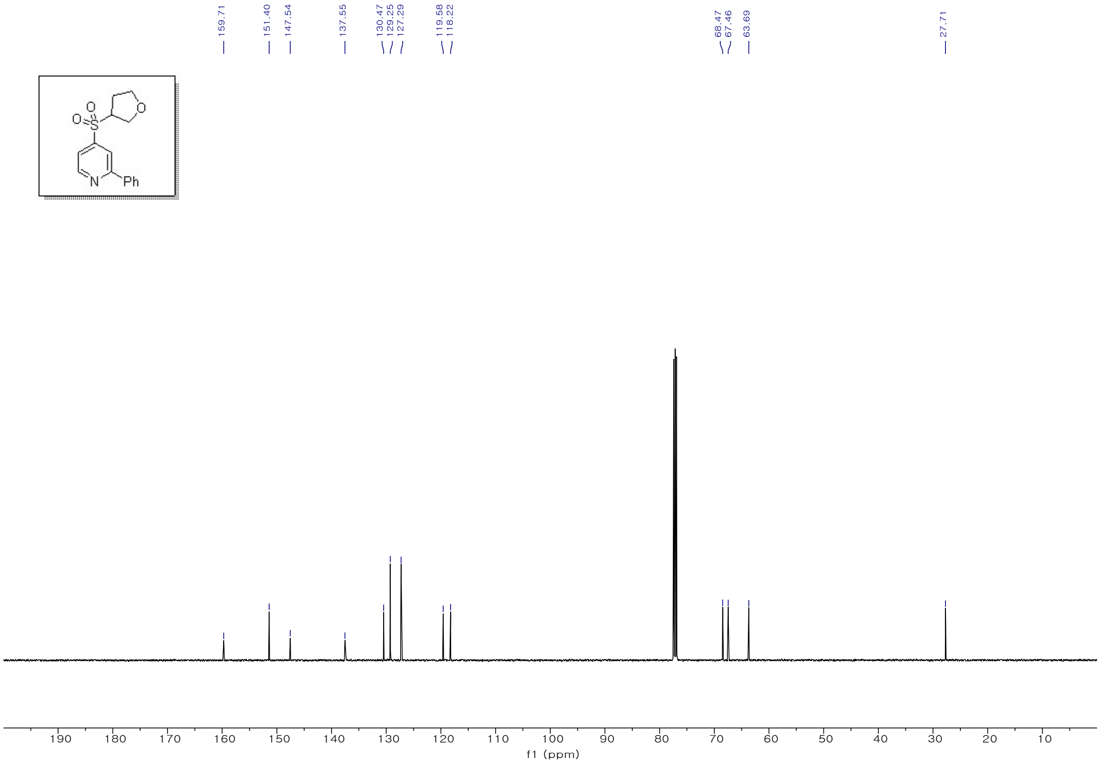

125 MHz, ^{13}C NMR in $CDCl_3$

***tert*-butyl 3-(quinolin-4-ylsulfonyl)azetidine-1-carboxylate (5t).**

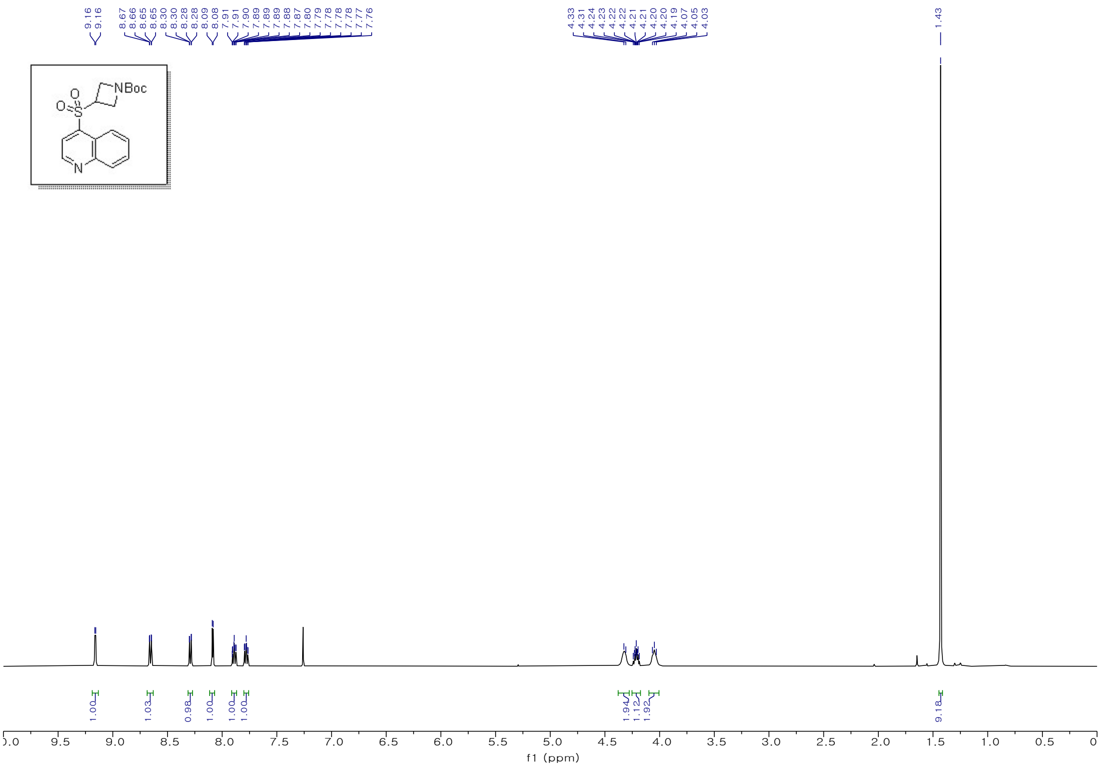

500 MHz, ^{1}H NMR in $CDCl_3$.

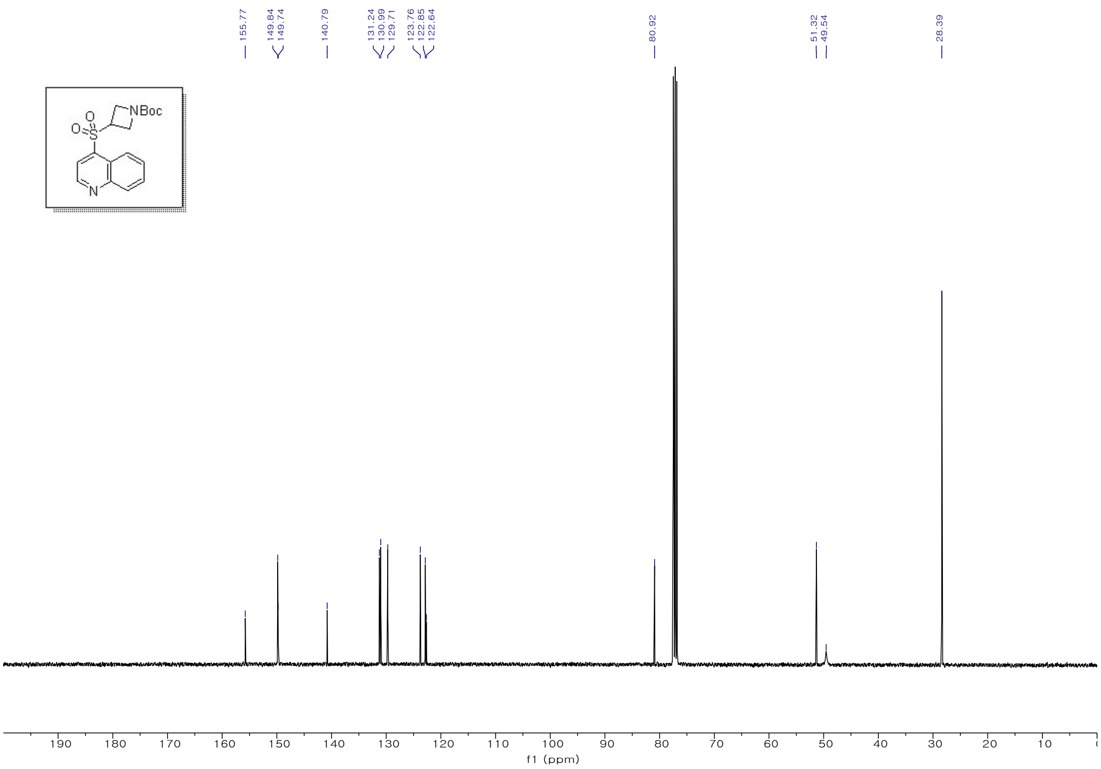

100 MHz, ^{13}C NMR in $CDCl_3$

***N*-(3-(4-(1-butoxy-2-tosylethyl)pyridin-2-yl)-4-chlorophenyl)-2-chloro-4-(methylsulfonyl) benzamide (6).**

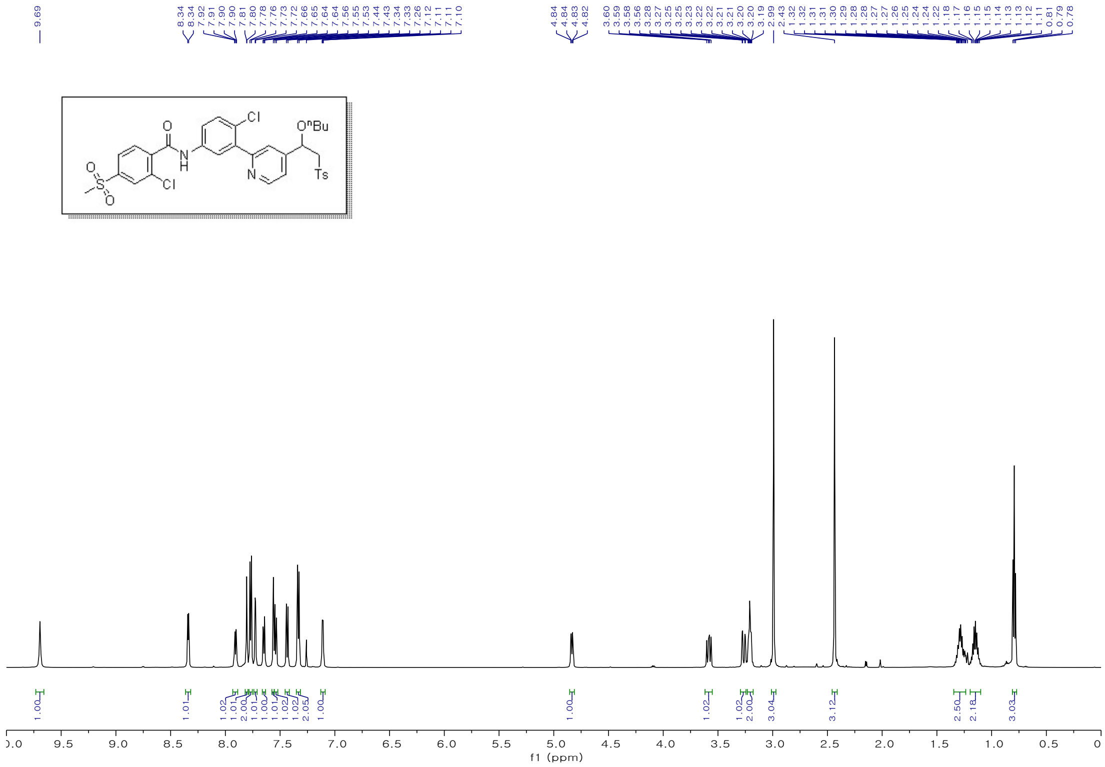

600 MHz, ^{1}H NMR in $CDCl_3$.

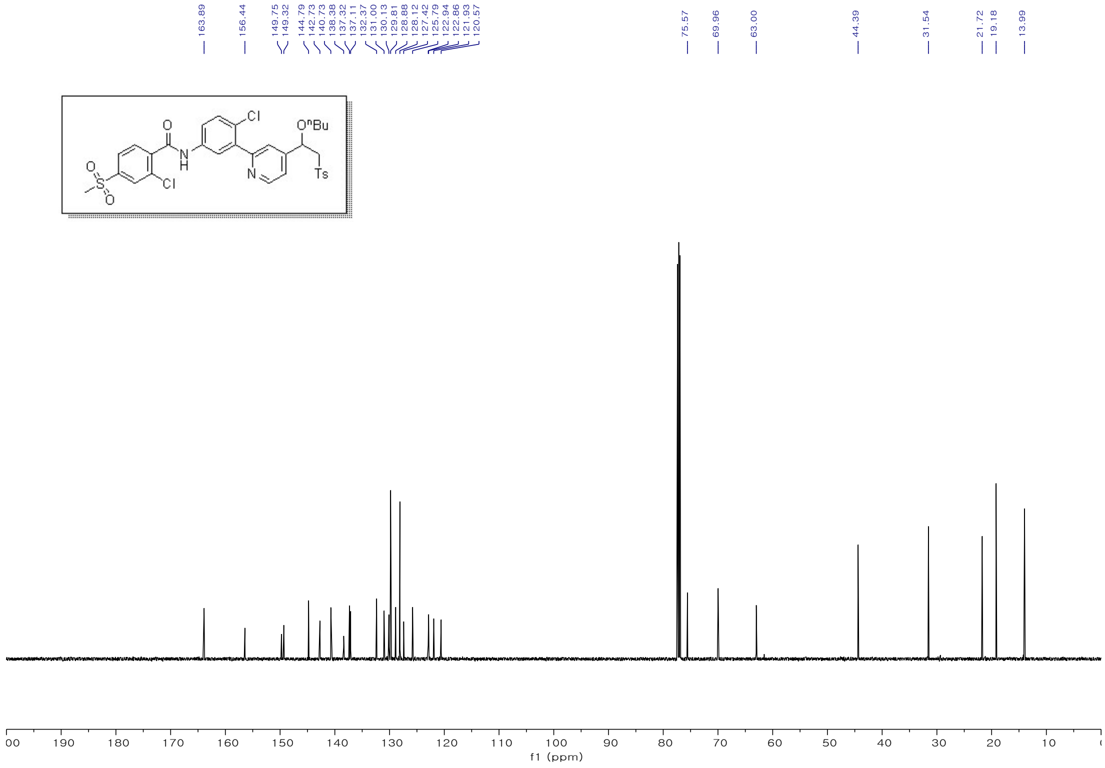

150 MHz, ^{13}C NMR in $CDCl_3$.

2-chloro-*N*-(4-chloro-3-(4-tosylpyridin-2-yl)phenyl)-4-(methylsulfonyl)benzamide (7)

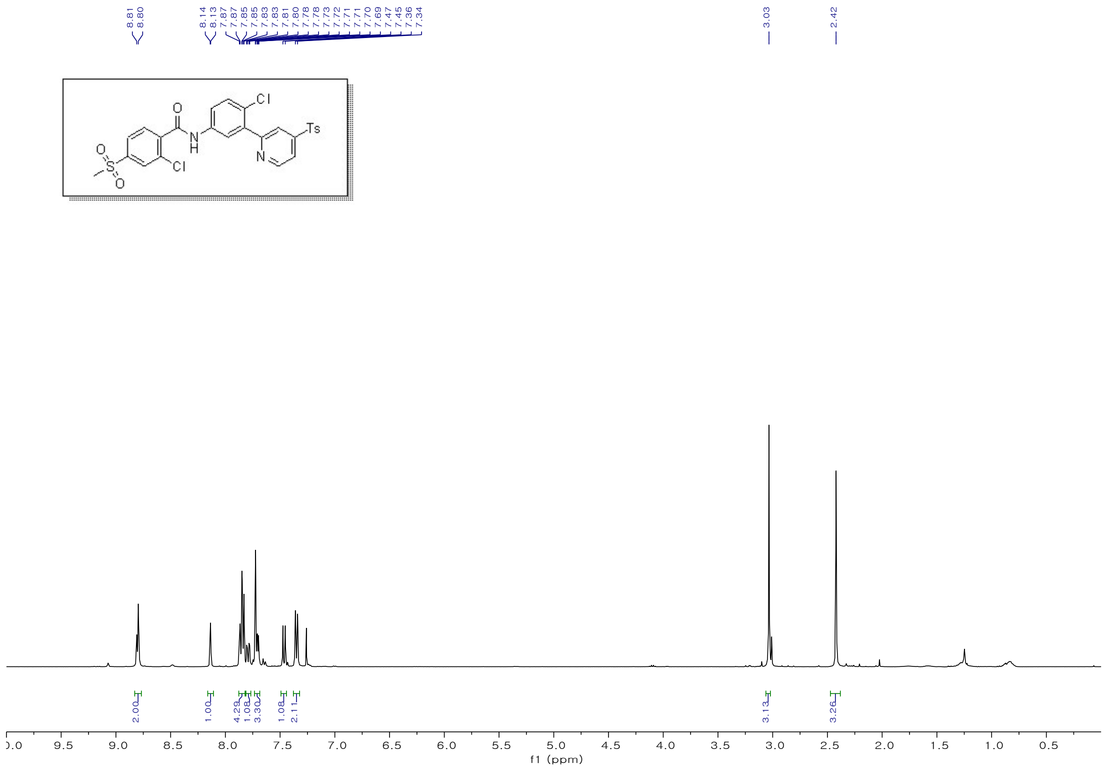

600 MHz, ^{1}H NMR in $CDCl_3$.

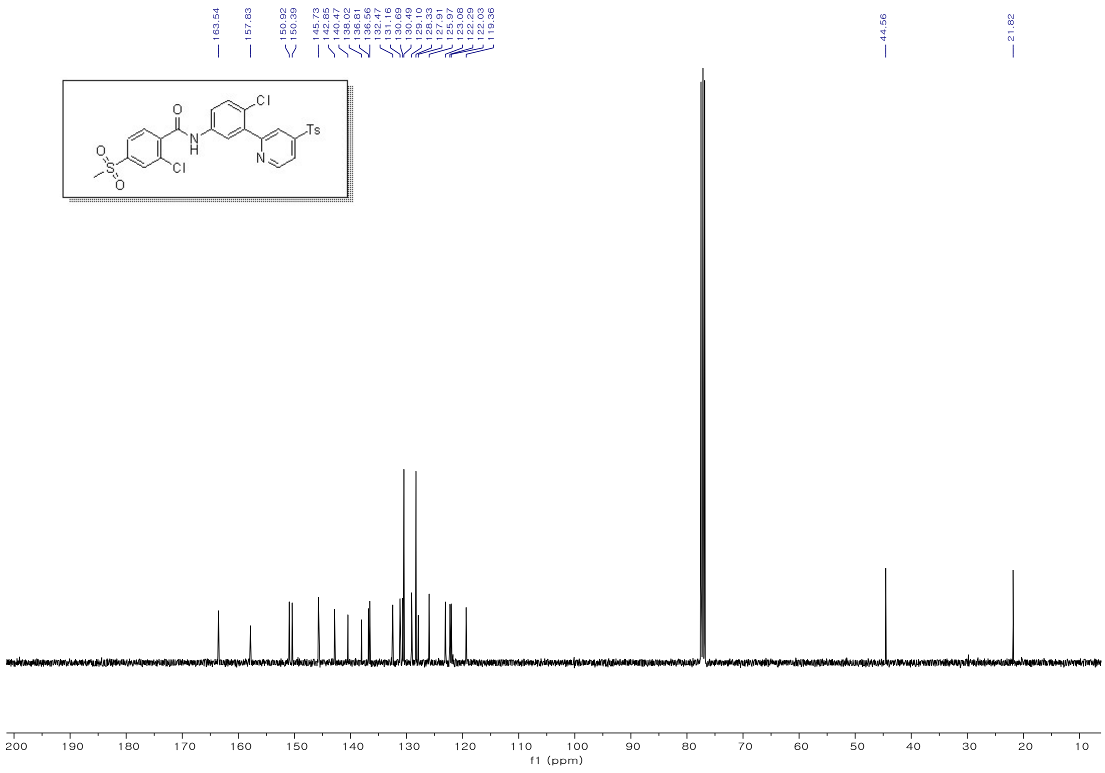

100 MHz, ^{13}C NMR in $CDCl_3$.

((4-(1-butoxy-2-tosylethyl)pyridin-2-yl)methylene)bis(4,1-phenylene) diacetate (8).

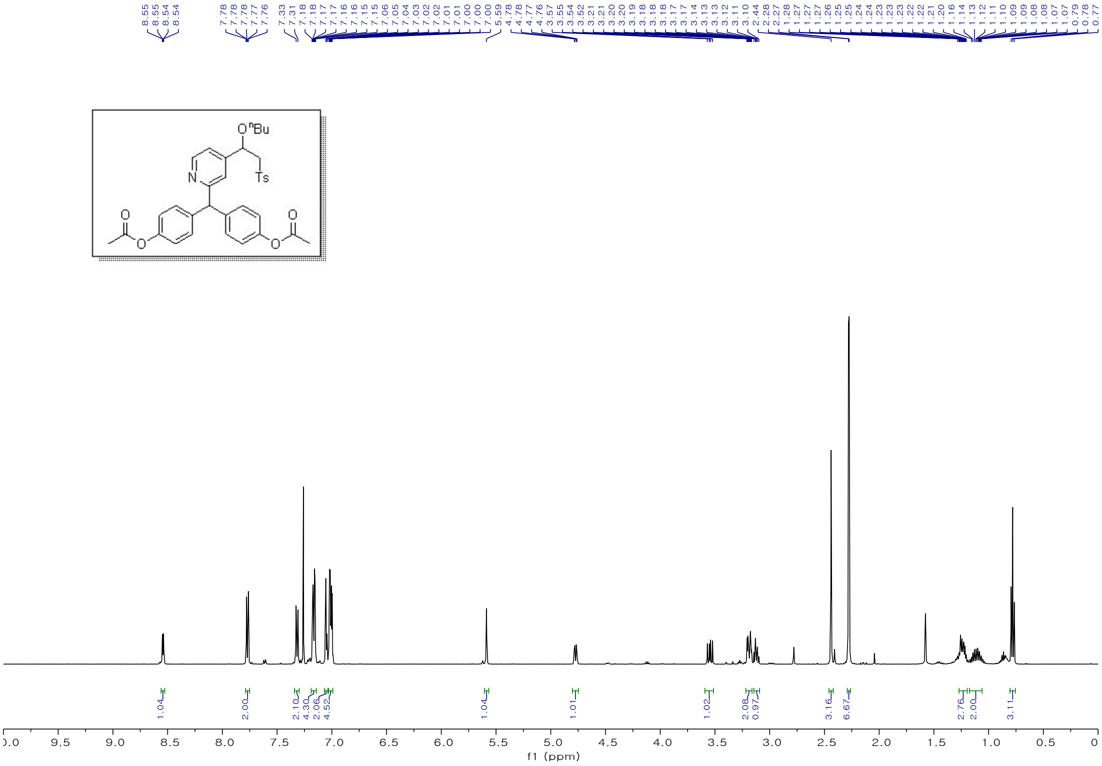

500 MHz, ^{1}H NMR in CDCl$_3$.

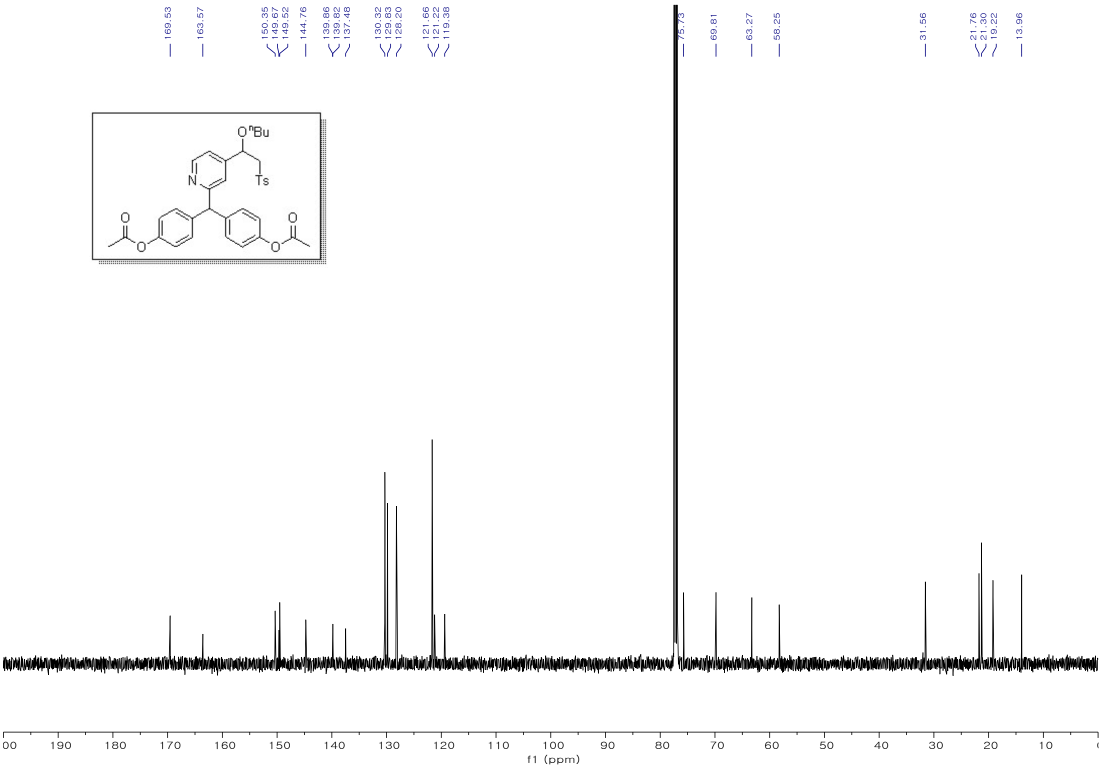

100 MHz, ^{13}C NMR in CDCl$_3$.

((4-tosylpyridin-2-yl)methylene)bis(4,1-phenylene) diacetate (9).

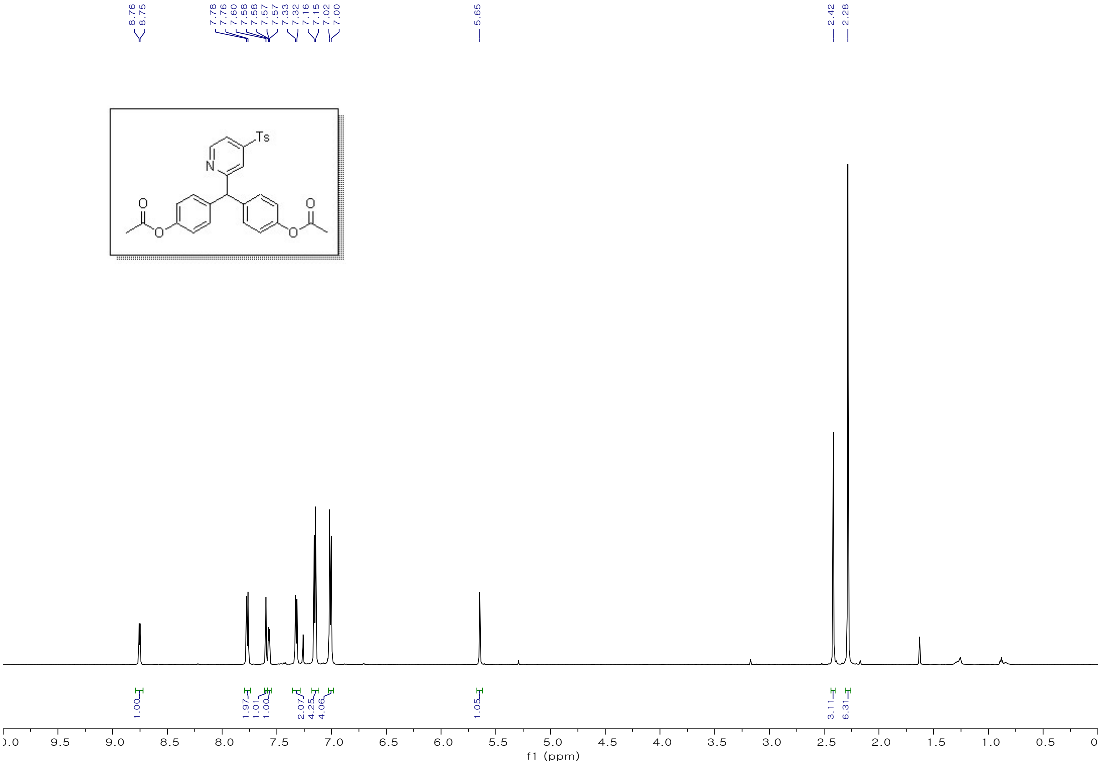

600 MHz, ^{1}H NMR in $CDCl_3$.

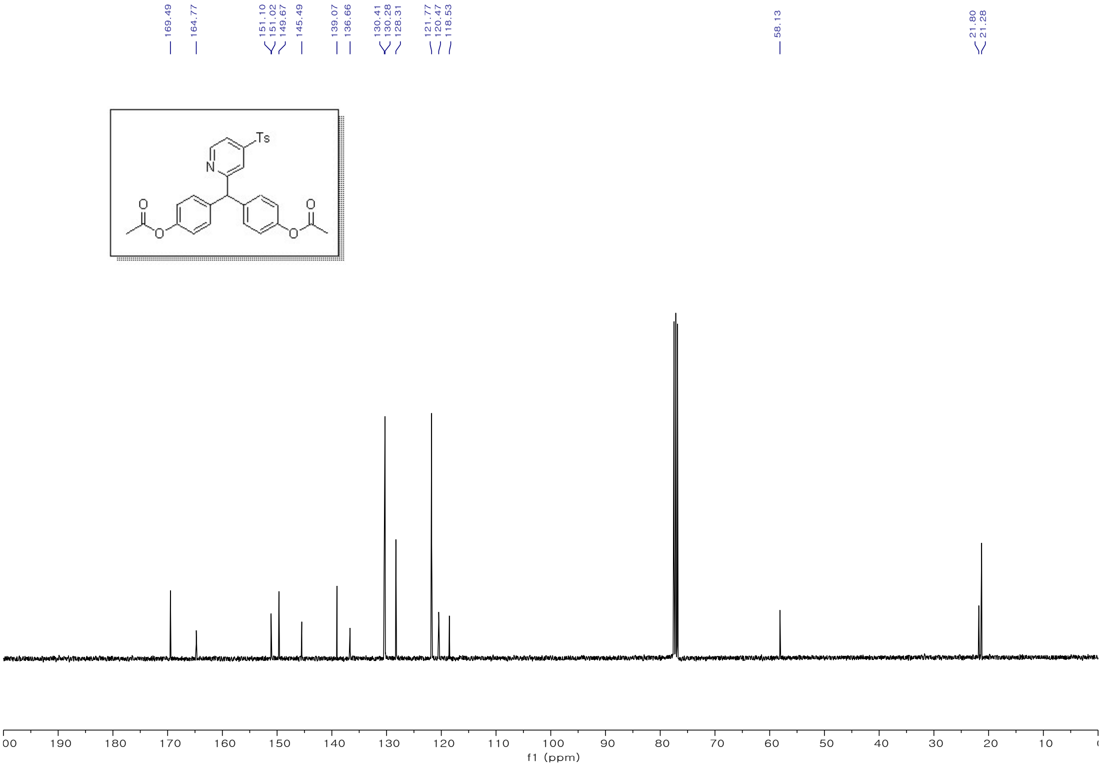

100 MHz, ^{13}C NMR in $CDCl_3$.

4-(1-butoxy-2-tosylethyl)-2-((1-(4-phenoxyphenoxy)propan-2-yl)oxy)pyridine (10).

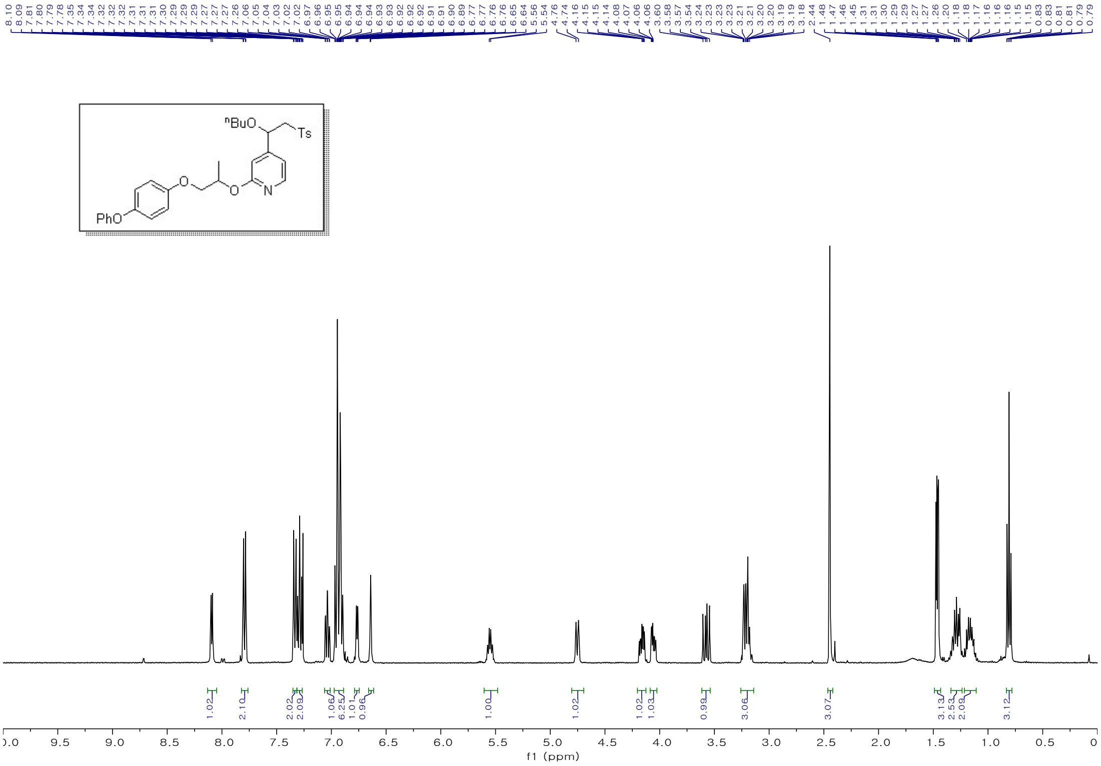

400 MHz, ^{1}H NMR in $CDCl_3$.

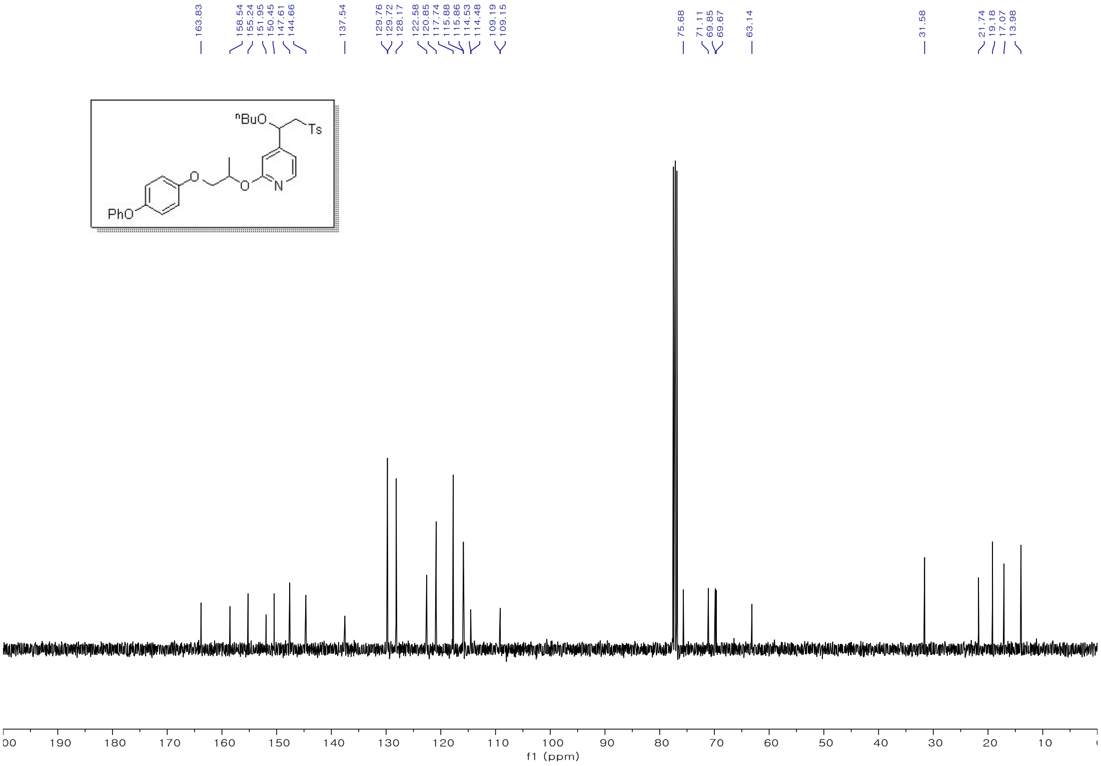

100 MHz, ^{13}C NMR in $CDCl_3$.

2-((1-(4-phenoxyphenoxy)propan-2-yl)oxy)-4-tosylpyridine (11).

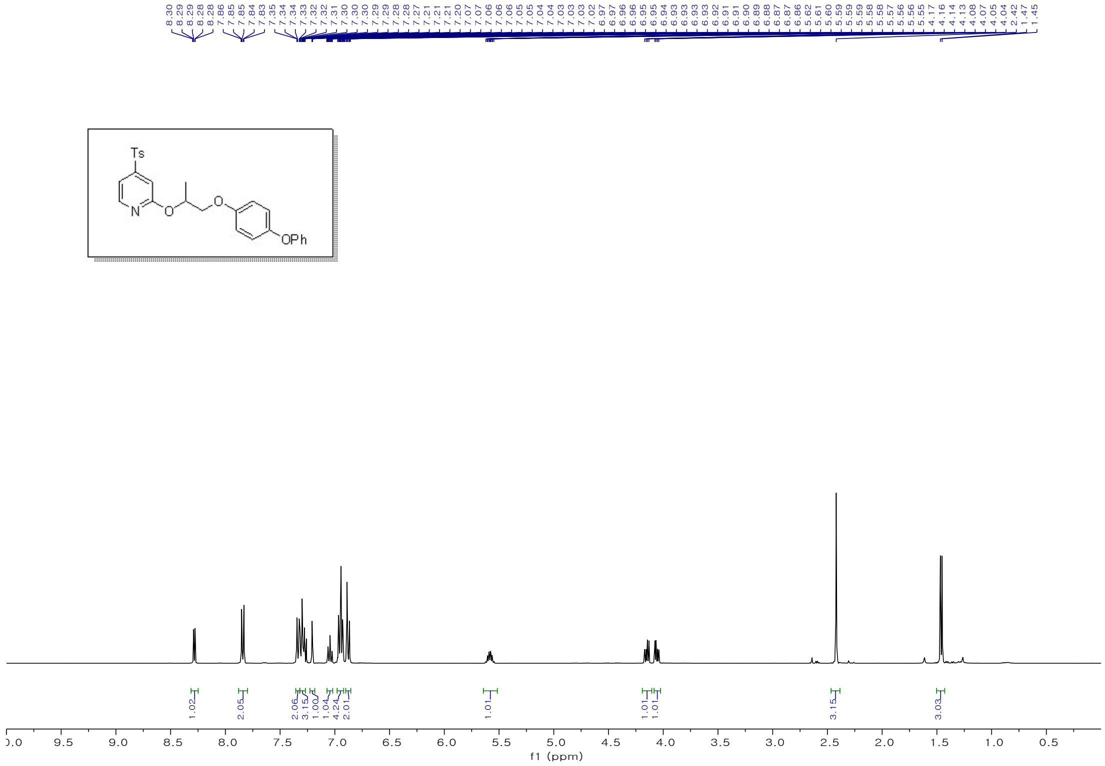

400 MHz, ¹H NMR in CDCl₃.

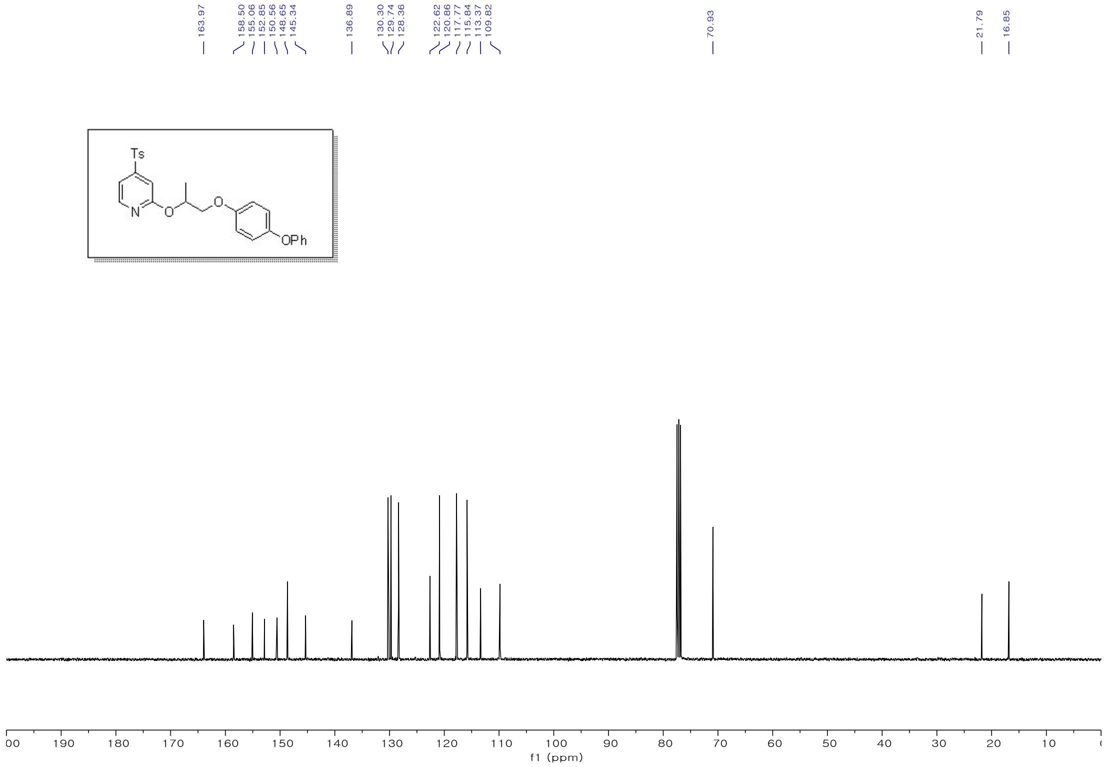

100 MHz, ¹³C NMR in CDCl₃.

***N*-(2-(methylsulfonyl)-1-(2-phenylpyridin-4-yl)ethyl)acetamide (4ba).**

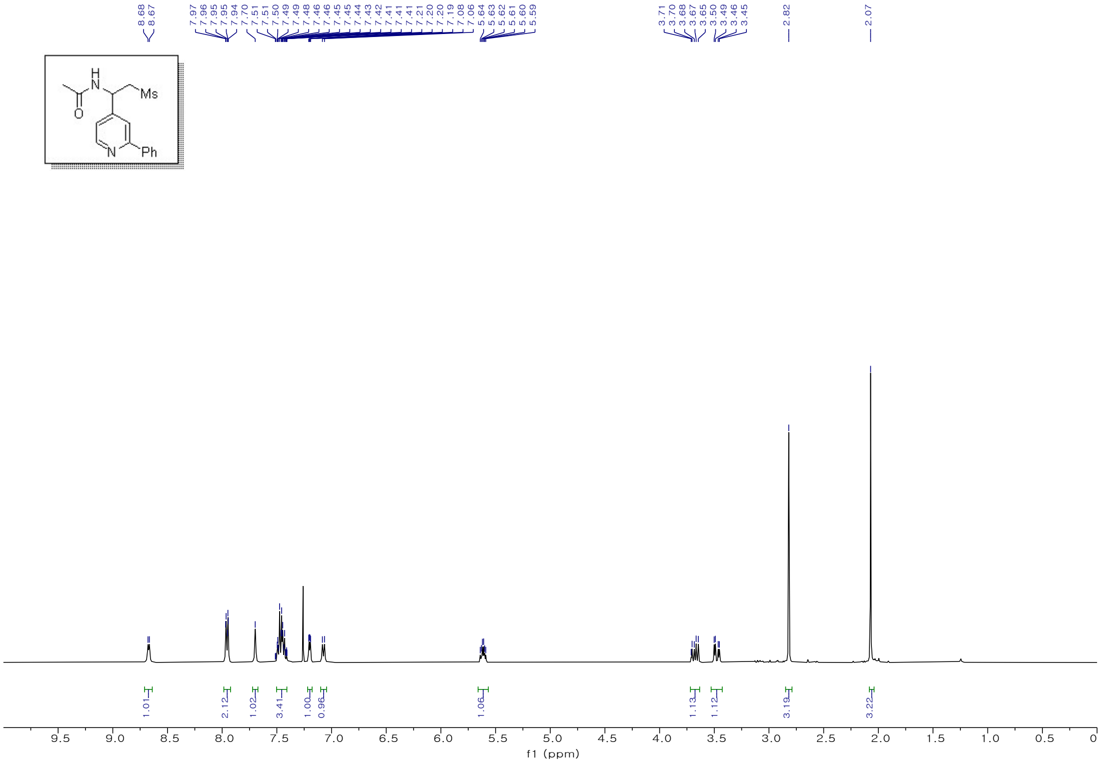

400 MHz, ^{1}H NMR in $CDCl_3$.

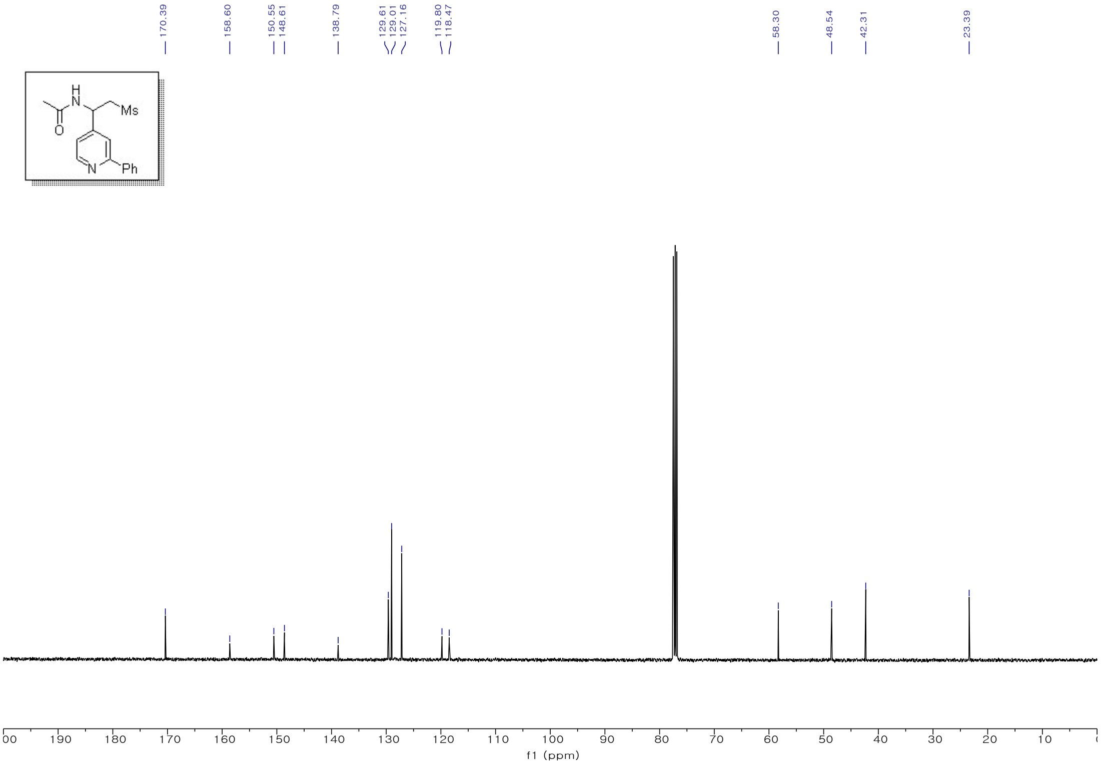

100 MHz, ^{13}C NMR in $CDCl_3$

***N*-(1-(2-phenylpyridin-4-yl)vinyl)acetamide (12).**

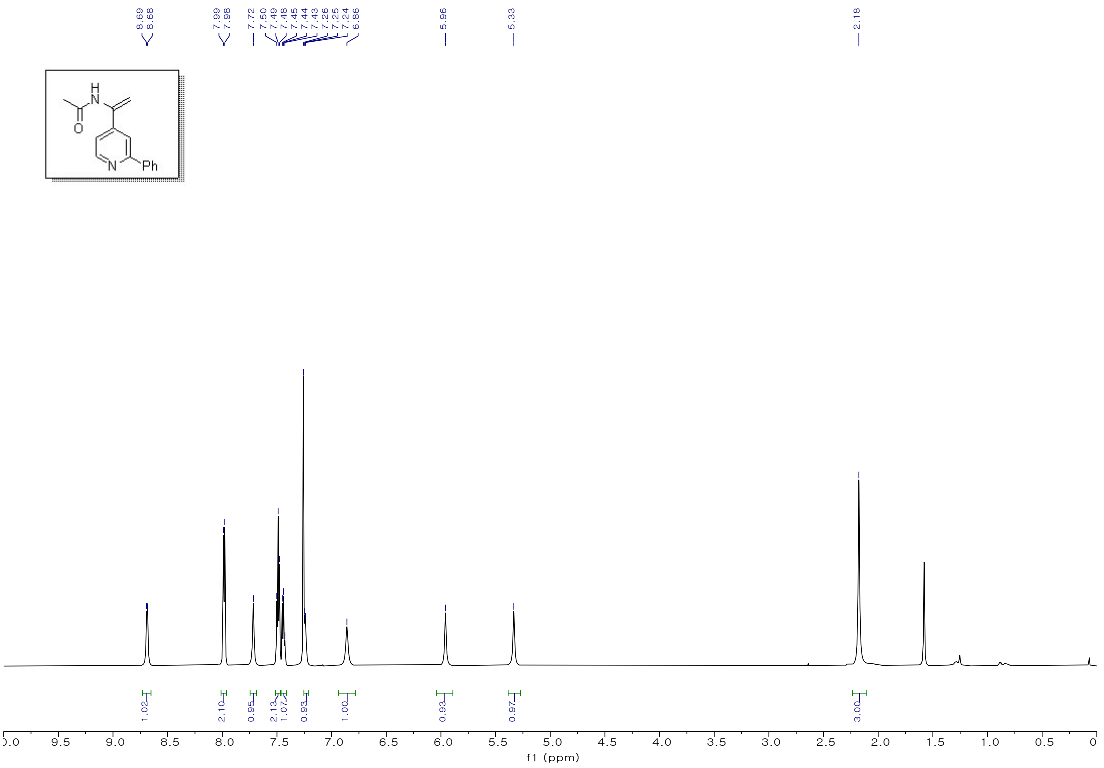

600 MHz, ^{1}H NMR in $CDCl_3$.

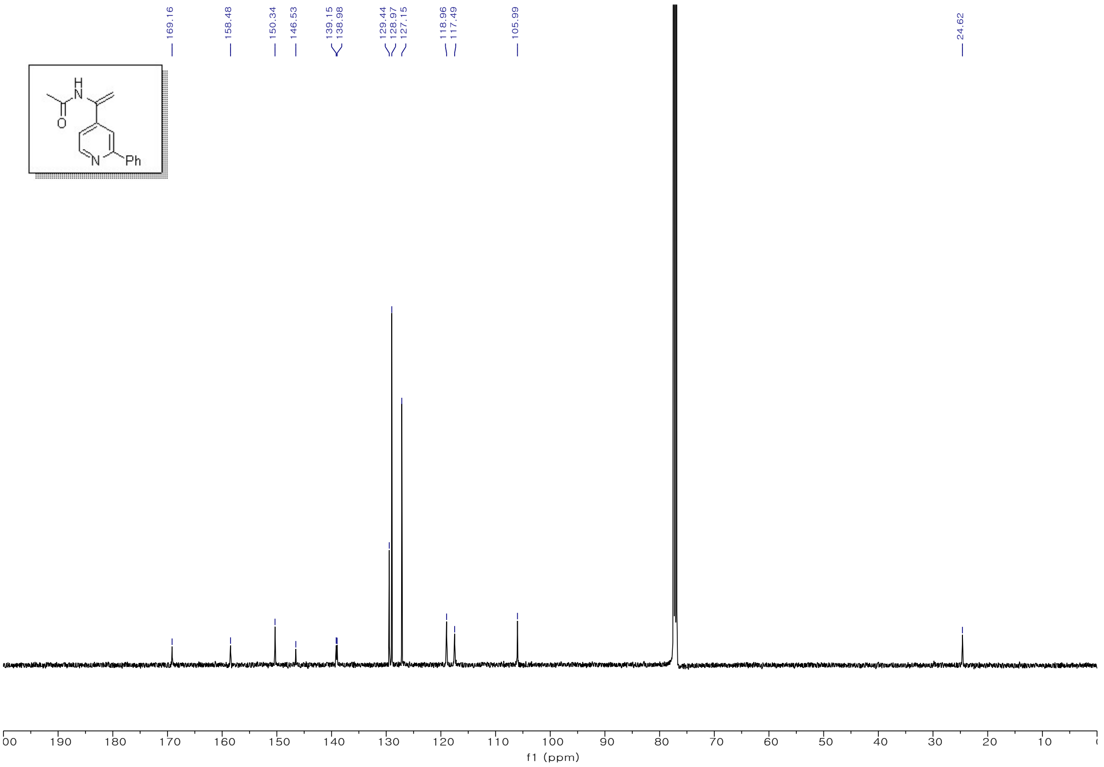

125 MHz, ^{13}C NMR in $CDCl_3$.

2-phenyl-4-(1-phenylethoxy)pyridine (13).

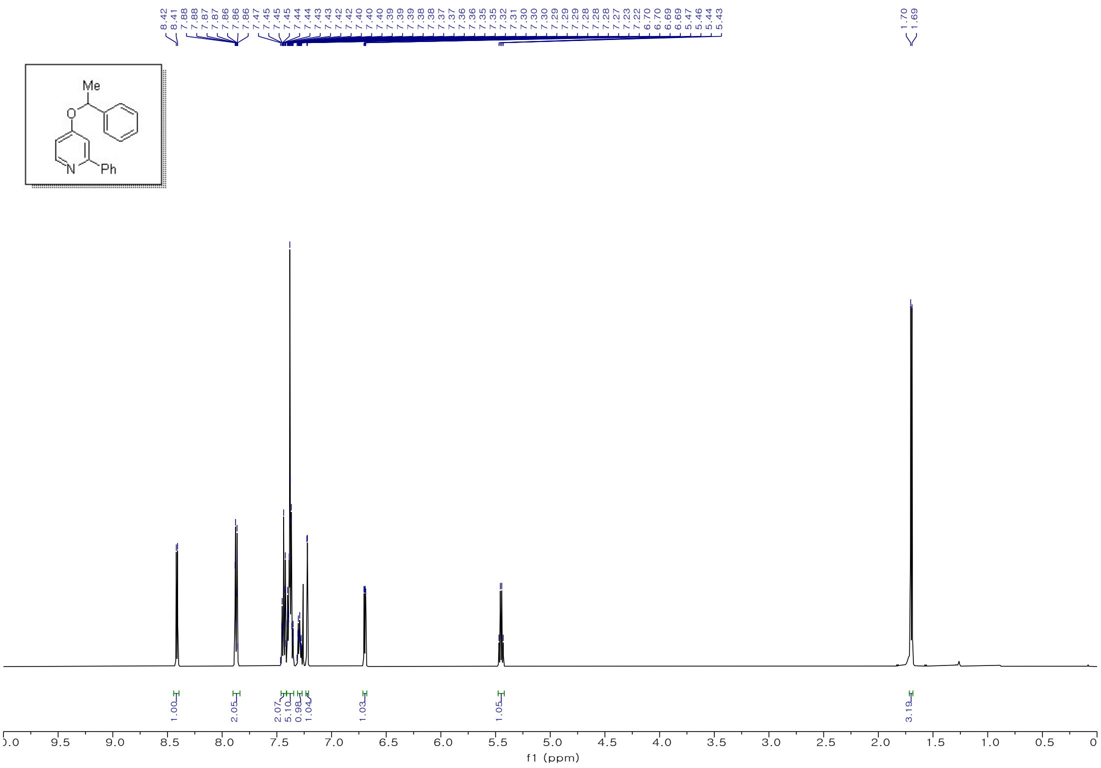

500 MHz, ^{1}H NMR in $CDCl_3$.

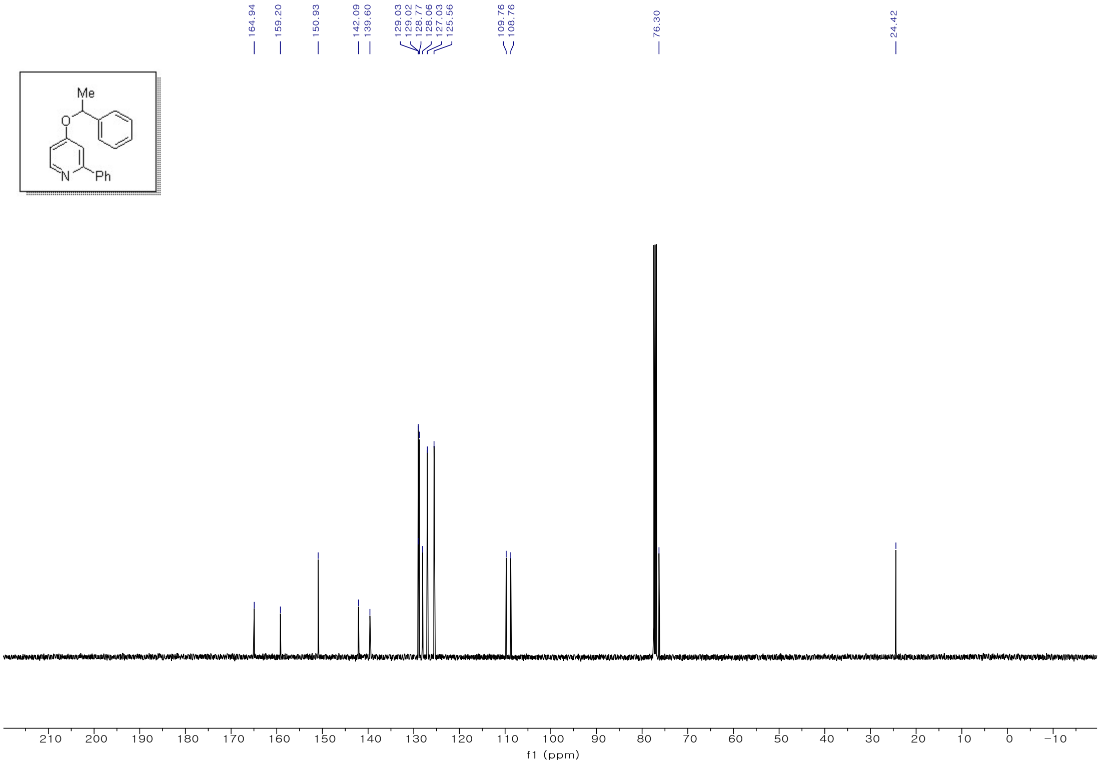

125 MHz, ^{13}C NMR in $CDCl_3$

4-(pyridin-4-ylsulfonyl)morpholine (15).

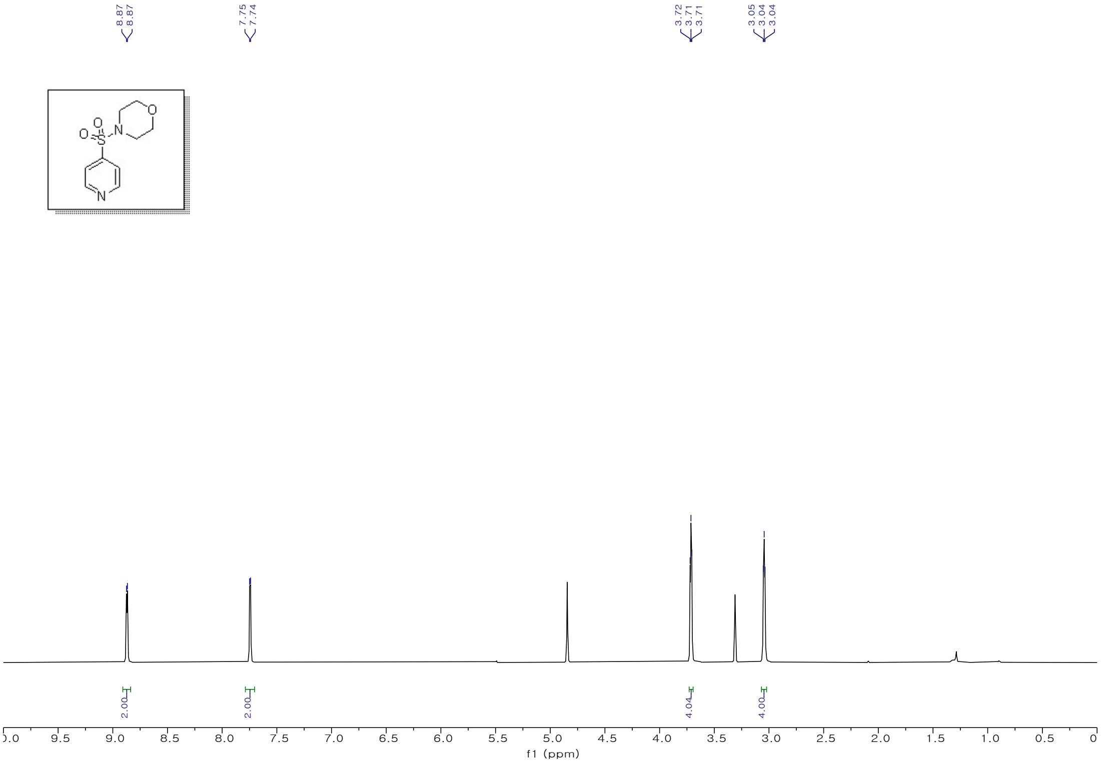

600 MHz, ^{1}H NMR in MeOD.

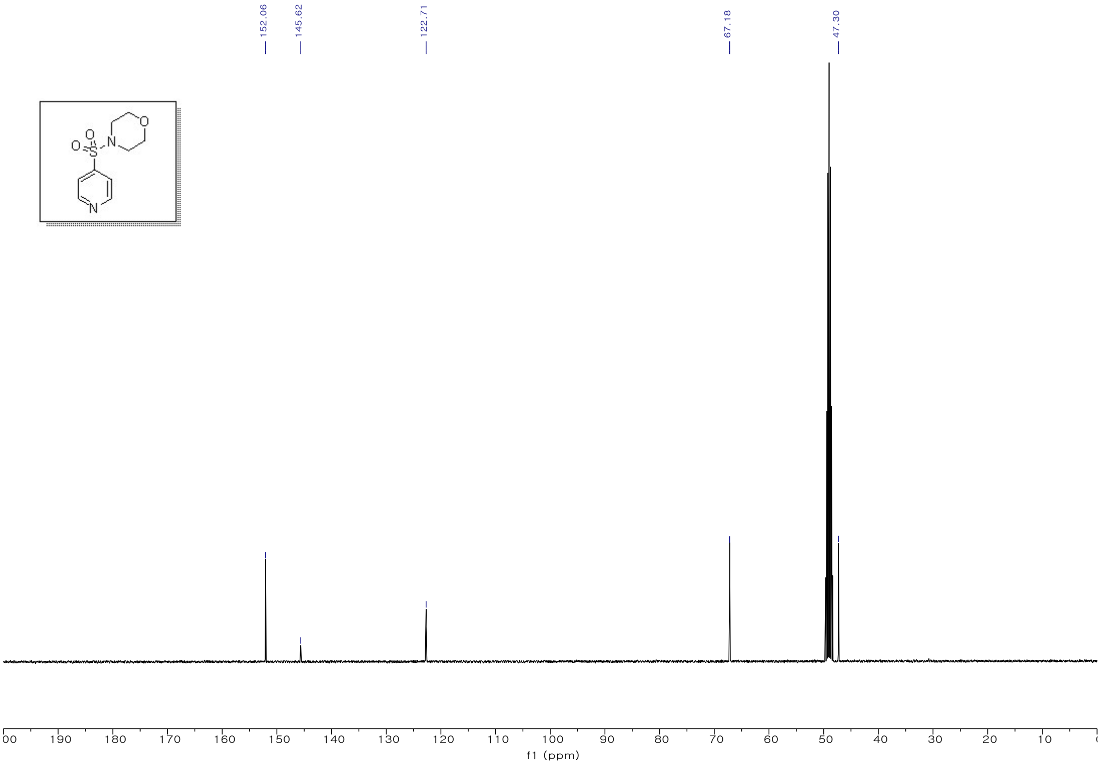

100 MHz, ^{13}C NMR in MeOD.

Appendix Ⅱ

DFT Calculation Data

DFT-optimized structure's energy components

Table 3. Compound energy components for optimized structures

	E(SCF)/(ev)	ZPE/(kcal/mol)	S(gas)/(cal/mol.K)	G(solv)/(kcal/mol)
	PW6B95-D3/def2-QZVP	B3LYP-D3/LACVP**	B3LYP-D3/LACVP**	B3LYP-D3/LACVP**
**1a**	-31672.140	166.384	129.430	-51.95
**3b**	-6335.403	70.945	78.716	-3.87
**A**	-22330.110	79.128	101.137	-8.42
**A-TS**	-28665.548	151.265	134.221	-15.15
**B**	-28665.896	152.236	134.793	-14.02
***o*-B-TS**	-60338.425	319.705	212.532	-51.37
***p*-B-TS**	-60338.489	319.868	222.149	-50.11
***o*-C**	-60338.819	320.938	219.598	-52.06
***p*-C**	-60338.846	320.776	220.093	-50.1
**D**	-60328.256	312.237	229.303	-24.38
**D-TS**	-60327.952	311.423	216.195	-23.06
**E**	-24909.607	106.107	117.214	-10.36
**4w**	-35419.003	204.362	160.527	-17.29
**Ts-**	-22332.491	78.282	97.631	-67.36
**NTsMe-**	-24912.423	106.323	109.200	-67.46
**NHTsMe**	-24927.790	115.277	111.987	-13.23
**amidyl-TS**	-31245.26916	179.402	144.622	-13.28
**amidyl-adduct**	-31245.99916	181.16	146.264	-13.01

Table 4. Cartesian coordinates of the optimized geometries The cartesian coordinates of optimized geometries are given below in the standard XYZ format, and units are in Å.

==============================

1a

==============================

```
C   -1.779647946   0.402214289  -3.280960560
C   -2.176398754   1.615876079  -2.712347269
C   -2.176501036   1.754751444  -1.336982489
N   -1.773038983   0.717248380  -0.558025360
C   -1.400836587  -0.473768532  -1.080859423
C   -1.389350176  -0.652281880  -2.452646017
H   -1.783142328   0.277873486  -4.358924389
H   -2.501704931   2.448440552  -3.325157166
H   -2.513926744   2.643508673  -0.820623815
H   -1.131634474  -1.228971720  -0.354962230
H   -1.087563872  -1.611019373  -2.857676744
N   -1.836322188   0.768792450   0.861965537
C   -1.110971093   1.919475079   1.433872700
H   -0.069667831   1.865687132   1.109711766
H   -1.546330094   2.889903784   1.172823191
H   -1.144702435   1.800776005   2.518089771
S   -3.594709396   0.710915983   1.298599005
O   -4.083866119  -0.388929069   0.471616954
O   -4.100229740   2.077915907   1.146275520
C   -3.491096735   0.265105367   2.998893976
C   -3.633234501   1.259057641   3.973951340
C   -3.285785675  -1.077316403   3.331505060
H   -3.822827578   2.286796331   3.684269428
H   -3.209102869  -1.831273317   2.555769444
C   -3.555496693   0.887065172   5.310783863
C   -3.212558031  -1.421159267   4.676996708
H   -3.673151016   1.643875241   6.080657005
H   -3.061185837  -2.460443974   4.952289581
C   -3.340504646  -0.450802416   5.683949947
C   -3.244660854  -0.824199319   7.140098095
H   -4.052940845  -0.365111917   7.717634201
H   -3.288384914  -1.905682445   7.286195755
H   -2.299955845  -0.465160102   7.565046787
```

==============================

3b

==============================

```
C    3.924592972  -0.775468707  -8.728738785
H    3.909176350   0.140480265  -8.148395538
H    4.141728401  -1.708485842  -8.223531723
C    3.724183798  -0.760635912 -10.046671867
H    3.741266489  -1.673479319 -10.645597458
O    3.513058424   0.392758131 -10.740879059
C    2.979020119   0.236710355 -12.057752609
H    3.615135670  -0.446141779 -12.642428398
H    1.973984599  -0.207750157 -12.004715919
```

C	2.928977251	1.610901833	-12.701927185
H	3.930983782	2.047203541	-12.744305611
H	2.531426430	1.541909695	-13.719141960
H	2.288662195	2.281225920	-12.121488571

==============================

A

==============================

C	0.477931082	1.116004825	-0.042946011
C	1.009001374	-0.102344476	0.375203878
C	0.132797137	-1.147860169	0.664841712
C	-1.254304290	-0.984818697	0.551264584
C	-1.752749562	0.259075254	0.130957246
C	-0.898254871	1.317302942	-0.162397355
S	1.600236535	2.501185179	-0.358126044
O	2.933763266	1.923365831	-0.686711907
O	0.895572245	3.439231873	-1.276749492
C	-2.204609632	-2.113103151	0.870613992
H	2.083098173	-0.229166344	0.456043422
H	0.535117447	-2.106573820	0.980475307
H	-2.826160431	0.397322237	0.029459355
H	-1.279749751	2.276703119	-0.494290709
H	-1.672043562	-3.014420509	1.184955716
H	-2.893294573	-1.829861045	1.674782634
H	-2.816836596	-2.369133711	-0.001567600

==============================

A-TS

==============================

O	2.047509432	2.645209074	-0.269246757
C	1.375490904	1.615495443	0.280142397
C	2.767969131	3.500782967	0.634064198
C	3.344909906	4.640603542	-0.182848245
C	0.438006401	0.937862754	-0.486384243
S	-1.567870259	2.011573076	-0.386842400
C	-1.065261960	3.728335857	-0.143860981
O	-2.247887373	1.930215836	-1.705376506
O	-2.248779774	1.577926993	0.859310389
C	-0.832655787	4.193102360	1.150059462
C	-0.327637017	5.481605530	1.327127695
C	-0.049922597	6.307588577	0.228559583
C	-0.299461156	5.813229084	-1.060980678
C	-0.797962427	4.527050495	-1.255329013
C	0.480145246	7.707859993	0.425358742
H	1.491079092	1.449112773	1.349090576
H	2.074189901	3.877668381	1.395766616
H	3.556794405	2.919339180	1.131598115
H	3.903530598	5.322179317	0.465664089
H	4.019983292	4.259953976	-0.954561710
H	2.537007332	5.196748734	-0.664895236
H	0.515556872	1.017495632	-1.567695498
H	0.061581403	-0.004679632	-0.100954168
H	-1.063249946	3.555810690	1.997394681
H	-0.150285602	5.854014874	2.333124161
H	-0.096262977	6.444705486	-1.922336459
H	-0.989806831	4.141789913	-2.251234531
H	1.015880346	7.804890633	1.374190331
H	1.161609650	7.994609833	-0.381603122
H	-0.338424325	8.438117981	0.434570521

==============================

B

==============================

O	2.171489239	2.504319906	-0.284811974
C	1.527873039	1.352295756	0.031332135
C	2.969825506	3.095365763	0.746657550
C	3.540447712	4.389453411	0.197315529
C	0.394962996	1.022706747	-0.835647225
S	-1.200862527	1.880008101	-0.332423627
C	-0.738246024	3.613861322	-0.224339619
O	-2.160351992	1.721155882	-1.439887166
O	-1.520783901	1.420650363	1.032103419
C	-0.405439943	4.156499386	1.015161633
C	0.017078709	5.484233379	1.081251621
C	0.109983660	6.272116184	-0.073911451
C	-0.244495511	5.701170444	-1.306290507
C	-0.669748604	4.378587723	-1.389525414
C	0.558875263	7.712265015	-0.004514131
H	1.586043358	0.990585148	1.054093361
H	2.333533525	3.288475037	1.621556044
H	3.763564110	2.395934582	1.044642568
H	4.159670353	4.881971359	0.953299463
H	4.155865669	4.193247318	-0.685185492
H	2.727769613	5.063435078	-0.086214893
H	0.560100555	1.322335124	-1.873698473
H	0.111421973	-0.032007188	-0.785966992
H	-0.486607730	3.542976618	1.905803323
H	0.276521891	5.916027546	2.044294834
H	-0.187509134	6.304169178	-2.208976269
H	-0.956810892	3.940999508	-2.340035200
H	0.985510409	7.955619812	0.972226083
H	1.312930107	7.929536343	-0.768542349
H	-0.282656461	8.392899513	-0.179693922

==============================

***o*-B-TS**

==============================

C	1.785476208	-0.642286718	-1.886934280
H	2.402533531	-1.485685110	-2.216360331
H	0.746473789	-0.840240180	-2.154030085
N	1.814880848	-0.414213002	-0.432922721
S	1.380849957	-1.867833614	0.533729553
O	1.633469582	-1.438438416	1.906520009
O	2.099725962	-3.002394915	-0.057531849
C	-0.343560934	-1.968233228	0.168810621
C	-1.197502255	-0.987352967	0.682902575
C	-0.804214418	-3.011247873	-0.638955653
C	-2.548217535	-1.064065099	0.362927765
H	-0.812712550	-0.183409125	1.300887823
C	-2.162461519	-3.065436125	-0.936389327
H	-0.112397276	-3.756319046	-1.016146779
C	-3.051601171	-2.097585440	-0.444510072
H	-3.225768328	-0.305760562	0.744259238
H	-2.538425446	-3.870534420	-1.560832262
C	-4.524498463	-2.180860996	-0.754067719
H	-4.707610130	-2.692057610	-1.702986717
H	-5.046685696	-2.744273424	0.028917482
H	-4.980356693	-1.188035727	-0.802025735
N	3.045683384	0.067144908	0.056504589
C	4.233078957	-0.525659025	-0.250544876
C	2.938208342	1.127941847	0.952460885
C	5.395613670	-0.094782449	0.343182743
H	4.190293312	-1.352208376	-0.946798384
C	4.127559662	1.500495553	1.639214396
H	1.965206027	1.174800873	1.436116576
C	5.337437630	0.934669852	1.316724658
H	6.330361366	-0.579606354	0.089200236
H	4.049695015	2.259139776	2.409566164
H	6.241695881	1.242035747	1.831255198
H	2.102424383	0.275670528	-2.386327028
S	-0.092465051	2.884931803	0.875704706
O	0.081073284	1.857167721	1.927523494
O	-0.545803726	2.500978708	-0.471658856
C	-1.123976827	4.177877426	1.524720311
C	-1.237729192	4.324628830	2.909474611
C	-1.795582294	5.024497509	0.638449728
C	-2.036852837	5.349345684	3.407974243
H	-0.725554109	3.637861967	3.574794769
C	-2.587813377	6.042311192	1.160773277
H	-1.712045550	4.872416973	-0.432804704
C	-2.721604824	6.221016407	2.547327042
H	-2.136770725	5.471354008	4.482637405
H	-3.118349314	6.705440998	0.483620226
C	-3.613855124	7.303214550	3.100219488
H	-3.652826548	8.170427322	2.435480356
H	-4.639176846	6.930118084	3.213035822
H	-3.276929617	7.637309551	4.085169792
C	2.443502426	2.852818727	-0.218153059
H	1.952385426	2.289862633	-1.016204834
C	1.539549470	3.688575029	0.653290391
H	1.962922812	3.884920597	1.642179966
H	1.352675676	4.648582935	0.158143207
O	3.570141554	3.503852606	-0.529684067
C	4.347747803	2.984718561	-1.640468955
H	4.684526920	1.974381089	-1.387307882
H	3.696228266	2.929924726	-2.522248507
C	5.518203735	3.920680761	-1.858337402
H	6.145615101	3.964379072	-0.964086115
H	5.168966770	4.929993153	-2.090247393
H	6.127344131	3.563560486	-2.693626165

==============================

***p*-B-TS**

==============================

C	-2.460758209	-1.239527702	0.079282999
H	-2.378554106	-1.591459870	1.113669395
H	-3.402554512	-1.594633579	-0.341482759
N	-2.466169119	0.233085364	-0.037060354
S	-3.678726435	1.049202204	0.998584092
O	-3.418914557	2.467980146	0.759655356

O	-3.581605911	0.466141611	2.341865778
C	-5.181500435	0.529414535	0.220698059
C	-5.571277618	1.138742685	-0.975650549
C	-5.947649002	-0.472291619	0.821327507
C	-6.751170635	0.720633686	-1.580143452
H	-4.967635155	1.927740097	-1.410686135
C	-7.126451015	-0.871806622	0.197451115
H	-5.627162933	-0.914750755	1.758113027
C	-7.545518398	-0.286735862	-1.006881475
H	-7.067413807	1.184878230	-2.509770393
H	-7.733098507	-1.647868156	0.654860675
C	-8.839865685	-0.703740001	-1.657648444
H	-9.110147476	-1.729840398	-1.394874454
H	-9.658583641	-0.052679125	-1.327594876
H	-8.782918930	-0.629110098	-2.747265577
N	-1.215631485	0.823355675	0.240418047
C	-0.531838953	0.571835458	1.399826884
C	-0.773252785	1.753640890	-0.655444920
C	0.629039109	1.238356113	1.673189044
H	-0.983897150	-0.136785567	2.078571558
C	0.373326302	2.459438801	-0.417203754
H	-1.415587664	1.895553350	-1.512901783
C	1.165541530	2.185250044	0.743601561
H	1.137105107	1.030093431	2.606579542
H	0.682267606	3.219884872	-1.124810338
H	1.780474067	2.986820459	1.139774919
H	-1.624907732	-1.612192154	-0.512679458
S	2.014227390	-1.101885319	-0.537046552
O	0.608264804	-1.087203383	-0.987297654
O	2.343274117	-1.215750456	0.896563590
C	2.882593393	-2.377705812	-1.426995754
C	2.416072130	-2.778298616	-2.680809498
C	4.022466660	-2.946279526	-0.853215277
C	3.116881371	-3.764360189	-3.370262384
H	1.515430570	-2.337795496	-3.095186234
C	4.706099987	-3.930556059	-1.560591578
H	4.349303722	-2.635054111	0.133358359
C	4.267877579	-4.354158401	-2.825108290
H	2.762447119	-4.086984158	-4.345005989
H	5.590468884	-4.384130478	-1.122314453
C	4.997772217	-5.447925091	-3.563095093
H	6.067048073	-5.441515923	-3.334229231
H	4.606722355	-6.430544853	-3.272095203
H	4.875774384	-5.353880405	-4.645459652
C	3.034424782	1.431007743	-0.007987907
H	3.364506006	0.999428511	0.938693464
C	2.771184444	0.466233611	-1.130974293
H	2.104678392	0.879102886	-1.892267466
H	3.725685358	0.207746029	-1.603940725
O	3.667562485	2.531080723	-0.442475617
C	4.435484409	3.281275034	0.537430108
H	3.779290676	3.552326202	1.375096202
H	5.228355885	2.628048182	0.921654582
C	4.990242481	4.509386539	-0.152279675
H	4.180769444	5.140600204	-0.528591931
H	5.631317139	4.224382877	-0.990201533
H	5.583927155	5.092918873	0.556802154

==============================

***o*-C**

==============================

C	2.237885475	-0.927631080	-1.859218717
H	2.949104786	-1.745810270	-2.016787529
H	1.266776204	-1.226622939	-2.257042885
N	2.029219627	-0.585069001	-0.441674978
S	1.448403120	-1.980810881	0.553563714
O	1.590419173	-1.485330224	1.920983076
O	2.194194555	-3.155397654	0.088360377
C	-0.233256221	-2.052647352	0.036047034
C	-1.115883946	-1.072671413	0.501303434
C	-0.629918993	-3.062749863	-0.845511019
C	-2.431171179	-1.114746690	0.055077266
H	-0.781888306	-0.302272916	1.187102199
C	-1.955081701	-3.083321095	-1.267286181
H	0.081952974	-3.810179234	-1.178458810
C	-2.871701956	-2.114471197	-0.828005910
H	-3.127194166	-0.354021162	0.395107925
H	-2.284269571	-3.862942457	-1.947885633
C	-4.310544968	-2.164534330	-1.273186684
H	-4.411983013	-2.636100531	-2.254314899
H	-4.906902790	-2.750960827	-0.563394904
H	-4.749618530	-1.164364219	-1.321607828
N	3.157564640	-0.043758143	0.196955934
C	4.325657845	-0.692163050	0.249691799
C	2.856860876	1.162867308	0.978928268
C	5.380689144	-0.223878965	1.028477192
H	4.392793655	-1.611335397	-0.320794374
C	3.999278307	1.577370167	1.829418659
H	1.993193984	0.898098409	1.615827799
C	5.198310852	0.924248219	1.837599993
H	6.313433170	-0.774915099	1.028125644
H	3.839375019	2.445843935	2.458590031
H	6.007411480	1.268579483	2.472679853
H	2.568750381	-0.028592862	-2.381915808
S	-0.137957469	2.645540953	1.190688610
O	0.093792550	1.617754698	2.234509230
O	-0.761729479	2.260216475	-0.087366633
C	-1.030731678	3.999041796	1.918081999
C	-0.961594999	4.194281101	3.299962282
C	-1.778450251	4.844156265	1.093904614
C	-1.651535153	5.266100883	3.857713938
H	-0.396464348	3.506919861	3.920542002
C	-2.460503817	5.909580708	1.674717665
H	-1.834872961	4.655773640	0.026804671
C	-2.408422947	6.137522221	3.059009552
H	-1.610421300	5.425665379	4.931364536
H	-3.048442364	6.572055340	1.046058178
C	-3.183336496	7.270962238	3.681911230
H	-3.301954985	8.106625557	2.987032652
H	-4.188052654	6.934303761	3.965549707
H	-2.694951057	7.641020298	4.587502003
C	2.366145849	2.333039761	0.031182546
H	1.750160098	1.869386077	-0.751190782
C	1.496742606	3.347426414	0.785421312
H	1.950548172	3.698497772	1.715411782
H	1.324347615	4.204449177	0.131228685
O	3.444359303	3.055073500	-0.503835678
C	3.985458851	2.563894510	-1.737807274
H	4.457259178	1.582684636	-1.582075953
H	3.172829390	2.435508728	-2.469305992
C	5.006038189	3.574079752	-2.226990223
H	5.804164410	3.697713375	-1.490062475
H	4.532394409	4.545457363	-2.390577555
H	5.449459553	3.238297701	-3.168601036

==============================

***p*-C**

==============================

C	-2.704695463	-1.105830312	0.312310249
H	-2.758791208	-1.391091347	1.368641973
H	-3.597343206	-1.468766689	-0.198492944
N	-2.661282301	0.356843233	0.099202983
S	-3.926950216	1.278743744	0.990435660
O	-3.652782440	2.663440466	0.612886608
O	-3.877768278	0.835440159	2.387282610
C	-5.384814262	0.661827505	0.202144697
C	-5.716536999	1.132925391	-1.072129250
C	-6.166181087	-0.288765579	0.864504099
C	-6.851337433	0.623830080	-1.692516565
H	-5.102502346	1.884063482	-1.556863189
C	-7.298727512	-0.781028450	0.222140953
H	-5.891518593	-0.624183357	1.858475089
C	-7.656892776	-0.338908046	-1.060603380
H	-7.121995449	0.979042947	-2.682560682
H	-7.916142464	-1.519245267	0.725333691
C	-8.897980690	-0.860777140	-1.737967968
H	-9.167803764	-1.855361342	-1.373691559
H	-9.746886253	-0.195608735	-1.539522648
H	-8.770815849	-0.911758184	-2.823003531
N	-1.418539166	0.931171954	0.406225026
C	-0.718940675	0.592833996	1.539841771
C	-0.934499264	1.847116113	-0.498969823
C	0.525651336	1.091972351	1.754674435
H	-1.214740396	-0.081461303	2.222430468
C	0.290238678	2.402929306	-0.331053823
H	-1.599496245	2.051836967	-1.325636744
C	1.174690604	2.033079147	0.805035293
H	1.061004162	0.788262486	2.645844460
H	0.663426280	3.109589100	-1.063504934
H	1.426827192	2.948160648	1.370995879
H	-1.812887192	-1.528429270	-0.152996048
S	1.837278008	-1.067556143	-0.315254539
O	0.426122427	-0.987162054	-0.753987849
O	2.160557032	-1.278046370	1.110893488
C	2.653155327	-2.313882589	-1.289286375
C	2.148878336	-2.629996538	-2.553180218
C	3.785898685	-2.948147774	-0.772623718
C	2.804502726	-3.595326185	-3.311787128

H	1.253662229	-2.140432119	-2.921293020
C	4.424284935	-3.910881042	-1.549248576
H	4.142936707	-2.703179598	0.222207665
C	3.947914600	-4.248281479	-2.826009750
H	2.420811176	-3.852064133	-4.295120716
H	5.303124428	-4.414404392	-1.156979442
C	4.627300739	-5.317072392	-3.644193649
H	5.691755772	-5.390188694	-3.406052351
H	4.177349091	-6.296244621	-3.440606833
H	4.525407314	-5.127351284	-4.716290951
C	2.620540619	1.570162773	0.301655769
H	3.150678873	1.186947346	1.184049726
C	2.635578394	0.498795003	-0.797842920
H	2.144555569	0.847453058	-1.708843946
H	3.679280281	0.269254178	-1.023997664
O	3.272317171	2.670020580	-0.272162378
C	3.978336573	3.520938873	0.644700706
H	3.283129692	3.929824352	1.395739675
H	4.733531952	2.928197622	1.180725813
C	4.614804268	4.638259888	-0.159205303
H	3.849879503	5.220309258	-0.680467308
H	5.304980755	4.228298664	-0.901027977
H	5.170595646	5.308075428	0.502808034

================================

D

================================

C	-3.140038013	1.910781264	-2.016010046
C	-3.537434340	1.578623176	-0.753660500
N	-4.634306908	2.217952967	-0.154911101
C	-5.375596046	3.144527197	-0.918843210
C	-4.982987881	3.468615770	-2.181199074
C	-3.839436054	2.877054691	-2.782213211
N	-5.251672268	1.519625664	0.891361713
C	-5.563603878	2.369068384	2.048836231
C	-6.714783669	-0.717034280	1.552532554
C	-7.661082268	-0.665423930	2.576817751
C	-7.693039894	-1.694436431	3.515610218
C	-6.796264172	-2.769651175	3.443259001
C	-5.861737251	-2.795844793	2.396350622
C	-5.812340260	-1.777312756	1.449325562
C	-6.824626923	-3.865253448	4.481256485
S	-6.662530899	0.585429490	0.332785070
O	-7.881334305	1.398938775	0.473887205
O	-6.255474567	0.029563650	-0.958580613
H	-2.274156332	1.404291749	-2.433552504
H	-3.063276529	0.831631064	-0.132386968
H	-6.258858204	3.543295860	-0.439275771
H	-5.582609177	4.169526577	-2.752944708
H	-4.628726006	2.819714069	2.386602402
H	-6.298732281	3.152584076	1.830733299
H	-5.956878662	1.731763005	2.844198942
H	-8.366538048	0.157658637	2.615331650
H	-8.432622910	-1.668016553	4.311450481
H	-5.170554638	-3.630803108	2.318878412
H	-5.103343487	-1.805905104	0.628777325
H	-6.219028473	-3.589807987	5.353708267
H	-7.842193604	-4.052226067	4.837339401
H	-6.423000336	-4.802821159	4.086521626
C	-5.150120735	1.974133015	-5.345239162
O	-4.603127956	3.259783268	-5.057303905
C	-3.469563484	3.228601933	-4.199193478
H	-5.449653625	1.468986630	-4.414912701
H	-4.386334896	1.348890185	-5.835080147
C	-6.344604492	2.183979034	-6.259709358
H	-6.035666943	2.704201221	-7.170724869
H	-7.104367733	2.791139841	-5.758239746
H	-6.791585922	1.222814083	-6.532748699
S	-2.279999733	5.012935638	-5.980791569
C	-2.860589981	4.632353783	-4.282865047
C	-1.108315825	6.348972321	-5.684977055
O	-1.512067795	3.838167191	-6.436841011
O	-3.381656647	5.569249630	-6.780292511
C	0.224240497	6.046403885	-5.396856308
C	1.114962578	7.086132526	-5.146287441
H	0.550006568	5.011126041	-5.393756866
C	-1.551343918	7.669078827	-5.735556602
C	-0.645365834	8.699159622	-5.480628490
H	-2.585213900	7.878226280	-5.989859104
C	0.694795489	8.425752640	-5.180243969
H	2.155727386	6.859370708	-4.928475380
C	1.679628253	9.537164688	-4.906586647
H	-0.984213948	9.730890274	-5.522424221
H	2.126156569	9.431447029	-3.911237001
H	2.501766682	9.520506859	-5.631184101
H	1.204542041	10.520376205	-4.959972858
H	-2.723362923	2.513805151	-4.579669952
H	-3.593464136	5.398185253	-4.018558979
H	-1.987054229	4.715785980	-3.631971836

================================

D-TS

================================

C	-2.653630733	2.079417229	-1.346178770
C	-2.954237223	1.854748249	-0.021202844
N	-3.988142252	2.505858421	0.612408221
C	-4.691942692	3.448823452	-0.111448623
C	-4.442078114	3.676732540	-1.443121672
C	-3.416998386	2.978828669	-2.107263803
N	-4.944368839	1.379345179	1.430186510
C	-5.426901817	2.031663179	2.650054216
C	-6.763263226	-0.627187252	1.245534301
C	-7.931580544	-0.586334527	2.007027626
C	-8.355392456	-1.745566487	2.654567480
C	-7.629655838	-2.939775705	2.550505161
C	-6.459448814	-2.949314117	1.774581790
C	-6.018971443	-1.802613020	1.121862888
C	-8.083187103	-4.185744286	3.271862745
S	-6.214997768	0.846108258	0.395446926
O	-7.330593586	1.808547616	0.392257512
O	-5.567785740	0.452047169	-0.861130357
H	-1.833153605	1.531122804	-1.800854445
H	-2.430740118	1.127392769	0.587607920
H	-5.488232613	3.951374531	0.423787504
H	-5.061952114	4.382269382	-1.986933827
H	-4.549851418	2.372860193	3.202562332
H	-6.100168228	2.877029419	2.461003780
H	-5.960797787	1.293791294	3.258041859
H	-8.503084183	0.333852470	2.063252449
H	-9.270933151	-1.725539327	3.239903927
H	-5.893832684	-3.871955872	1.674245715
H	-5.125570297	-1.812425852	0.506789267
H	-7.603463173	-4.262038708	4.255461216
H	-9.164866447	-4.183970451	3.434488058
H	-7.823187351	-5.089210987	2.712072611
C	-4.993355751	1.631763816	-4.252377987
O	-4.481160164	2.969158411	-4.279700756
C	-3.247564793	3.131172180	-3.604557991
H	-5.133156300	1.294487476	-3.216460466
H	-4.271143436	0.955857456	-4.738024235
C	-6.314129829	1.642190814	-5.000335217
H	-6.170391560	2.002249479	-6.023049355
H	-7.027397633	2.302389383	-4.497939110
H	-6.739321232	0.634134293	-5.033427715
S	-2.359102249	4.654090881	-5.755962849
C	-2.749150515	4.530812263	-3.970189571
C	-1.354470968	6.145650864	-5.804278374
O	-1.486385703	3.506586790	-6.069315434
O	-3.580057383	4.924991608	-6.527615070
C	0.024252048	6.057382584	-5.602094173
C	0.784019709	7.223423958	-5.613475323
H	0.484333247	5.084900856	-5.458903790
C	-1.973538160	7.373891354	-6.028469563
C	-1.196919203	8.532490730	-6.035064220
H	-3.042423725	7.411070824	-6.211277962
C	0.186573595	8.476393700	-5.825515747
H	1.858986378	7.164176941	-5.462350845
C	1.031854630	9.727422714	-5.840037346
H	-1.672654510	9.493407249	-6.211935520
H	1.624581575	9.813976288	-4.922562122
H	1.737340212	9.713305473	-6.678910732
H	0.419214070	10.628121376	-5.932959080
H	-2.507265329	2.399618149	-3.965739965
H	-3.502867699	5.291328430	-3.753345013
H	-1.822602391	4.765293598	-3.440527439

================================

E

================================

C	0.342091560	-0.150346175	-0.058462735
C	0.937112868	-1.287139416	0.484829575
C	0.130854249	-2.374599218	0.818530619
C	-1.253937364	-2.338443279	0.612947285
C	-1.821446300	-1.181827903	0.054591201
C	-1.034376264	-0.086895645	-0.285518676
S	1.361795425	1.243684649	-0.493858993
O	2.751523972	0.783512473	-0.638550043
O	0.702038884	1.996708989	-1.572901249

C	-2.129930973	-3.514548302	0.971640289
N	1.251336813	2.205368042	0.907837749
C	2.111919403	3.369436264	0.814241588
H	2.011659861	-1.318390608	0.628271997
H	0.586434901	-3.266661644	1.239184022
H	-2.893279076	-1.144860744	-0.121729068
H	-1.468023181	0.800566375	-0.733539402
H	-1.547345400	-4.341233253	1.386559963
H	-2.885511160	-3.228355646	1.712062597
H	-2.666107893	-3.886957407	0.091445118
H	2.020624399	3.946015120	1.736480713
H	3.159295559	3.074335814	0.656147957
H	1.821467638	3.996095657	-0.042577416

==============================

4w

==============================

C	-3.093306780	1.880729437	-2.283975840
C	-3.387410879	1.723122478	-0.929091394
N	-4.452266216	2.267130852	-0.325899303
C	-5.265195370	3.007518053	-1.090835929
C	-5.059182167	3.232343912	-2.451996326
C	-3.948511600	2.653753757	-3.070888996
H	-2.213259220	1.427739382	-2.727551937
H	-2.732575178	1.126298904	-0.296131611
H	-6.123357296	3.451169968	-0.588628888
H	-5.750011921	3.854878664	-3.015173435
C	-1.502917171	3.779094219	-4.710001469
O	-2.334927559	2.634200573	-4.904533863
C	-3.688541412	2.835160971	-4.559052944
H	-1.510889769	4.078418732	-3.650423050
H	-1.895677686	4.619141102	-5.302405834
C	-0.103300221	3.404940605	-5.162321568
H	-0.121598825	3.090043068	-6.209226608
H	0.286160171	2.578084946	-4.560639858
H	0.572654605	4.259490490	-5.059865475
S	-4.351926327	2.157084227	-7.156593323
C	-4.496860027	1.812352419	-5.359885216
C	-5.857835770	1.424244285	-7.808947086
O	-4.461196899	3.621128321	-7.307724953
O	-3.214190006	1.411580086	-7.713917732
C	-7.036566734	2.173837185	-7.821329594
C	-8.199959755	1.592873335	-8.315388680
H	-7.025382519	3.200398445	-7.469439983
C	-5.827570438	0.117909670	-8.291958809
C	-7.003748417	-0.448004156	-8.784929276
H	-4.892234325	-0.431843579	-8.296480179
C	-8.202751160	0.275166333	-8.801567078
H	-9.120548248	2.170657158	-8.335580826
C	-9.477012634	-0.331260324	-9.338071823
H	-6.986905098	-1.464363575	-9.168841362
H	-10.259277344	-0.345084041	-8.570609093
H	-9.863745689	0.252366602	-10.181166649
H	-9.323225021	-1.357633233	-9.681394577
H	-4.019122124	3.839293003	-4.864468575
H	-4.127132893	0.798191488	-5.192503929
H	-5.558957577	1.868063927	-5.109652042

==============================

Ts-

==============================

H	3.030880928	3.971944332	-4.988975525
C	3.395308256	1.998810530	-6.844512463
H	2.932752371	2.913022041	-7.234355927
H	2.980020285	1.149637222	-7.399783611
H	4.465280533	2.050453901	-7.092407227
C	3.170876503	1.852994323	-5.355772495
C	3.127295494	0.586182237	-4.754362106
C	2.968896627	0.453284234	-3.373753786
C	2.846956253	1.582195997	-2.562123537
C	2.866223812	2.845794439	-3.153835773
C	3.026171446	2.980319738	-4.534319401
H	3.211832047	-0.301937431	-5.382124424
H	2.905412436	-0.522987843	-2.897983789
S	2.810437918	1.406677246	-0.691309690
H	2.725623846	3.710654020	-2.509108782
O	2.007240295	2.642152548	-0.290779442
O	2.098864317	0.064982049	-0.533274770

==============================

NTsMe-

==============================

C	0.333474696	-0.647635281	-0.362370312
H	0.239530966	-1.060415864	-1.389819026
H	1.193537116	0.043565799	-0.386156291
N	0.411417782	-1.660147190	0.677812338
O	2.988960266	-1.755703926	0.326906264
O	1.789137721	-3.421472788	1.818746567
S	1.739149570	-2.511157274	0.648211777
H	-0.570461452	-0.046983697	-0.188781738
C	1.568701029	-3.641406536	-0.778579533
C	2.017843246	-3.262079477	-2.045692444
C	0.874789178	-4.848147392	-0.635082722
C	1.765222907	-4.074439526	-3.154922247
C	0.637095511	-5.658308506	-1.741768122
C	1.073586702	-5.283030987	-3.022876263
H	2.581582069	-2.339846849	-2.141124249
H	0.541496396	-5.133863926	0.357853949
H	2.116579533	-3.766514778	-4.139212608
H	0.103052758	-6.600029945	-1.617434859
C	0.826961219	-6.181782722	-4.213969231
H	0.941011250	-5.637904644	-5.157923222
H	1.529091835	-7.026813030	-4.238121510
H	-0.183097750	-6.608630180	-4.194138527

==============================

NHTsMe

==============================

C	-0.742057502	-1.953533769	1.181116104
H	-1.444941878	-1.123604536	1.076163173
H	-0.690443575	-2.266460896	2.229957104
N	0.551403999	-1.523436069	0.635740101
O	3.033799171	-1.866519809	0.545271754
O	1.632573605	-3.546132088	1.872872710
S	1.810157537	-2.656646013	0.716700673
H	-1.115605116	-2.790023327	0.583486497
C	1.518164873	-3.626337290	-0.756598115
C	1.893166184	-3.106928825	-1.996231318
C	0.936360836	-4.889418125	-0.648184001
C	1.668175340	-3.865467072	-3.141454697
C	0.716200233	-5.633041859	-1.806229711
C	1.072928429	-5.133664608	-3.066173792
H	2.366106510	-2.132466316	-2.053783417
H	0.682261229	-5.279868126	0.331164628
H	1.963705301	-3.470512390	-4.109719753
H	0.267196864	-6.619915485	-1.729836106
C	0.803048015	-5.938978195	-4.313417435
H	1.414121747	-5.598005295	-5.153472424
H	1.005072832	-7.002857685	-4.153673649
H	-0.248602346	-5.850814819	-4.613056660
H	0.910853088	-0.663463414	1.045338035

==============================

amisyl-TS

==============================

O	-5.968439579	3.486218929	-1.464693785
C	-6.397161007	2.650339842	-2.566331625
H	-5.532625198	2.447579145	-3.213086128
H	-7.150928974	3.201345444	-3.137685299
C	-6.956535339	1.371300459	-1.976151943
H	-7.816574574	1.590347052	-1.336873293
H	-6.200916767	0.854704320	-1.377099991
H	-7.283101559	0.702655256	-2.778437138
C	-8.244434357	5.571798801	-0.106666081
N	-7.437111378	6.561925411	-0.816465557
S	-7.812891006	6.655792236	-2.454203367
H	-7.916899681	5.562816143	0.936484575
H	-9.306250572	5.858671665	-0.124637477
H	-8.157364845	4.560831070	-0.524638891
C	-9.107900620	9.087818146	-2.336285830
C	-9.268139839	7.703744888	-2.428778887
C	-10.536793709	7.127141953	-2.482164145
C	-10.239020348	9.897240639	-2.296859980
H	-8.109910011	9.511770248	-2.309637547
C	-11.528836250	9.345754623	-2.345643520
H	-10.122790337	10.975947380	-2.229598284
C	-11.658989906	7.953923225	-2.437584400
C	-12.745411873	10.239735603	-2.335917473
H	-12.650925636	7.511543274	-2.481788874
H	-10.632215500	6.050893307	-2.577009678
H	-12.589779854	11.120854378	-1.705669522
H	-12.970770836	10.599650383	-3.347429991
H	-13.630839348	9.710856438	-1.971598506
O	-6.721110821	7.395278931	-3.105300665
O	-8.229892731	5.347521305	-3.013421774
C	-5.515076637	4.701698780	-1.804075718
H	-5.348628521	4.896371365	-2.860943556
C	-5.425903320	5.701804161	-0.873080730

H	-5.497786045	5.468252182	0.182345495
H	-4.975593567	6.640346050	-1.162722111

==============================

amidyl-adduct

==============================

C	1.680549145	3.601066828	0.435812265
H	1.861658096	4.070950508	1.402324438
H	1.722087026	4.363085747	-0.358370781
N	2.659679651	2.528458834	0.226136580
S	4.269388199	2.974035501	0.490168840
O	5.076117039	1.767602563	0.286337465
O	4.293211937	3.719408035	1.751071692
C	4.672258854	4.123628139	-0.824469507
C	5.134756088	3.633732557	-2.047476053
C	4.451866627	5.489115715	-0.636923850
C	5.371108532	4.525870800	-3.090317488
H	5.324512959	2.572271109	-2.164403915
C	4.694942474	6.366997719	-1.691199422
H	4.115501404	5.849806786	0.329013765
C	5.154594421	5.901812553	-2.930703163
H	5.735659122	4.149405479	-4.042507172
H	4.529708862	7.431657314	-1.547954559
C	5.447505951	6.864234924	-4.055866718
H	4.804583549	7.747954845	-4.005294323
H	6.486542225	7.212501049	-4.007632256
H	5.306210041	6.393584251	-5.033300877
H	0.685654879	3.154755116	0.430786431
C	1.249756694	0.761905372	-0.760367095
H	1.300864816	-0.015764391	0.001753984
C	2.407961845	1.670856357	-0.965628207
H	3.305227041	1.068195224	-1.119119883
H	2.241648912	2.294331551	-1.857484818
O	0.025362769	1.310464740	-1.007201433
C	-1.098975420	0.481506228	-0.690780103
H	-1.054687262	-0.435315698	-1.294600844
H	-1.050033569	0.189215839	0.368807763
C	-2.360400200	1.273656011	-0.981069446
H	-2.395931721	1.564618349	-2.034649372
H	-2.391549587	2.181427956	-0.371501535
H	-3.245921850	0.671931803	-0.754809380

==============================

D1

==============================

C	-1.910319686	0.003943990	-3.177218199
C	-2.267439842	1.302557349	-2.745542288
C	-2.131363630	1.681966186	-1.439962506
N	-1.597732306	0.781770766	-0.491566360
C	-1.307089448	-0.532964826	-0.891046345
C	-1.437038302	-0.905804813	-2.196650743
H	-2.031560898	-0.297195554	-4.210385799
H	-2.668816328	2.027162790	-3.447096348
H	-2.420950890	2.649616957	-1.054316282
H	-0.969188571	-1.178108454	-0.092032835
H	-1.171898603	-1.923050880	-2.466815472
N	-1.837805271	0.969778061	0.872068048
C	-1.137394786	2.131500959	1.432107925
H	-0.070532009	1.992805123	1.248011827
H	-1.467705250	3.086995363	1.007781267
H	-1.310432911	2.150159836	2.510810852
S	-3.566555262	0.943751454	1.282297492
O	-4.142126560	-0.087247454	0.417794764
O	-4.099153042	2.316506386	1.308172107
C	-3.496215343	0.338272512	2.960268021
C	-3.664102077	1.224245906	4.025510788
C	-3.262193680	-1.020722389	3.176246405
H	-3.867088318	2.270713806	3.825082541
H	-3.156635523	-1.691521287	2.330211163
C	-3.586824179	0.733984232	5.326861858
C	-3.190395832	-1.491268516	4.484235287
H	-3.722145319	1.414988875	6.163150311
H	-3.015355349	-2.548910379	4.662426472
C	-3.345111132	-0.624754369	5.576791763
C	-3.238063574	-1.134164572	6.993684769
H	-4.013751984	-0.699805617	7.632572651
H	-3.329887152	-2.222671509	7.038461208
H	-2.269386768	-0.864778280	7.432065964

==============================

D1-TS

==============================

C	-2.441407919	0.039731860	-2.838836193
C	-2.669889927	1.352138162	-2.401721001
C	-2.115895510	1.794829965	-1.219108462
N	-1.346411467	0.971315503	-0.424961865
C	-1.118384361	-0.315410376	-0.855491459
C	-1.636172891	-0.788287282	-2.040181637
H	-2.878237724	-0.326405823	-3.761035681
H	-3.267966270	2.042016268	-2.988933086
H	-2.270652771	2.798959017	-0.843148887
H	-0.518387556	-0.920204997	-0.186478004
H	-1.406893015	-1.805091858	-2.343350172
N	-1.726758599	0.962354600	1.219981074
C	-1.156962276	2.182115316	1.795970917
H	-0.093651451	2.189393520	1.550226927
H	-1.629681468	3.103386879	1.433091164
H	-1.266448379	2.143846273	2.884874582
S	-3.427499294	0.834401190	1.438500881
O	-3.858680487	-0.273003876	0.580854297
O	-4.073255539	2.155828476	1.341791749
C	-3.499629498	0.306526452	3.144616365
C	-3.793163776	1.232981443	4.144337654
C	-3.242649794	-1.032836676	3.450219393
H	-4.013425827	2.260278940	3.874550343
H	-3.036748409	-1.736333966	2.650633335
C	-3.821028233	0.807282448	5.472156048
C	-3.276931047	-1.438228726	4.779496670
H	-4.056499004	1.521602750	6.256627560
H	-3.087346554	-2.479763746	5.026398182
C	-3.559999466	-0.526612043	5.810092926
C	-3.561965466	-0.981410325	7.249397278
H	-4.077284813	-0.267253757	7.897402287
H	-4.049808979	-1.955411315	7.358752728
H	-2.536859512	-1.089402080	7.624771595

==============================

pyridine

==============================

C	5.297641277	-0.415294021	-2.753362894
C	5.297641277	0.783052981	-2.040919065
C	5.297641277	0.726952016	-0.646122992
N	5.297641277	-0.415294021	0.052907005
C	5.297641277	-1.557540059	-0.646122992
C	5.297641277	-1.613641024	-2.040919065
H	5.297641277	-0.415294021	-3.839605093
H	5.297641277	1.741711020	-2.550136089
H	5.297641277	1.645049930	-0.060958996
H	5.297641277	-2.475637913	-0.060958996
H	5.297641277	-2.572299004	-2.550136089

Table 5. Vibrational frequencies (in cm^{-1}) of the optimized structures

==============================

1a

==============================

7.08	21.95	43.14	46.88	60.60	92.56
152.08	171.71	209.18	234.88	249.42	273.73
281.06	306.71	327.39	354.65	406.92	413.53
416.06	442.99	476.99	501.23	536.48	559.19
580.40	633.95	642.98	655.21	677.30	691.88
705.71	784.32	809.78	819.09	828.44	853.66
865.59	969.42	979.11	992.64	1000.26	1021.07
1030.02	1042.89	1043.95	1060.04	1064.66	1078.78
1087.67	1120.52	1143.46	1147.51	1160.40	1194.14
1196.64	1202.51	1227.15	1236.52	1238.33	1334.93
1343.03	1346.83	1365.52	1380.40	1428.54	1447.92
1461.81	1494.44	1495.73	1504.51	1511.98	1515.52
1530.09	1531.86	1622.20	1624.89	1646.71	1672.13
3049.40	3053.56	3112.09	3141.91	3145.52	3169.60
3199.15	3200.98	3222.38	3223.45	3225.11	3238.20
3241.63	3267.53	3272.31			

==============================

3b

==============================

61.21	87.31	214.60	261.64	440.14	500.98
711.93	832.67	841.93	863.43	984.94	993.40
1092.58	1157.21	1185.26	1232.09	1307.51	1351.42
1402.97	1430.97	1456.75	1496.62	1516.14	1536.81
1733.15	2991.31	3030.33	3055.79	3126.88	3133.17
3137.12	3182.08	3276.60			

==============================

A

==============================

14.21	61.24	99.83	168.44	245.19	294.41
319.50	403.91	416.47	433.90	463.78	523.75
616.24	642.36	707.63	808.66	829.01	854.05
976.16	984.95	1012.79	1018.75	1035.59	1065.81
1086.44	1151.07	1207.75	1220.98	1234.92	1336.52
1354.10	1428.32	1440.89	1496.69	1507.24	1530.01
1625.74	1646.73	3041.87	3099.89	3130.83	3182.61
3187.41	3221.67	3222.29			

==============================

A-TS

==============================

-274.30	15.85	42.33	49.82	55.48	82.81
109.95	123.38	125.69	158.48	168.77	216.24
255.96	267.29	296.78	310.45	346.02	418.78
419.46	436.11	447.63	478.11	498.93	548.17
626.28	645.72	705.10	716.53	812.83	831.26
836.00	837.81	860.41	922.34	965.25	973.02
984.13	1012.12	1019.17	1030.39	1061.62	1064.18
1095.76	1106.88	1142.06	1147.16	1185.33	1218.04
1229.94	1234.28	1256.69	1293.56	1313.69	1335.41
1352.33	1397.20	1427.20	1429.63	1439.37	1455.27
1497.26	1502.31	1504.70	1515.61	1526.07	1534.21
1568.58	1627.27	1652.05	3018.11	3038.82	3058.65
3071.39	3100.01	3125.45	3134.88	3147.66	3148.03
3172.41	3176.40	3178.36	3214.00	3216.40	3246.63

==============================

B

==============================

13.15	26.44	51.99	57.41	86.98	102.02
139.67	157.43	168.84	181.78	238.71	263.11
266.22	292.30	319.72	337.35	416.81	418.77
430.97	445.17	486.53	498.83	507.95	579.37
604.46	646.51	672.53	720.54	815.64	830.31
832.93	840.47	858.43	943.96	975.00	983.61
1017.57	1034.92	1039.89	1064.64	1085.85	1094.81
1127.03	1143.51	1149.71	1183.95	1206.24	1219.10
1222.13	1235.32	1273.62	1305.02	1308.38	1337.91
1355.37	1393.29	1423.94	1428.03	1441.52	1442.90
1483.17	1497.38	1499.20	1507.28	1515.51	1529.07
1537.88	1630.20	1655.94	3010.93	3041.33	3052.85
3057.74	3081.15	3100.29	3128.47	3133.65	3145.02
3152.62	3180.39	3181.81	3188.47	3213.83	3219.98

==============================

***o*-B-TS**

==============================

-219.34	7.21	19.90	25.39	31.44	33.68
39.97	45.90	54.77	58.79	60.47	64.33
68.32	78.95	93.49	102.88	121.68	128.62
144.66	150.31	175.36	176.72	195.08	206.20
211.35	217.67	237.34	262.69	271.90	275.06
278.01	303.63	311.15	320.18	324.01	331.16
346.38	358.69	401.85	415.08	415.79	416.76
432.11	432.99	444.37	467.85	483.55	486.40
498.13	526.60	534.82	559.66	566.77	576.61
627.12	638.75	641.14	643.57	664.15	672.45
687.67	706.76	708.80	747.65	760.01	794.08
813.35	815.42	829.16	834.64	835.45	854.51
855.74	857.56	862.80	894.63	936.88	952.14
981.08	984.99	990.32	1000.45	1003.34	1011.50
1023.05	1023.23	1029.51	1030.30	1036.40	1042.07
1056.91	1064.00	1064.51	1072.72	1078.58	1087.13
1093.00	1107.58	1131.11	1143.31	1144.39	1146.34
1157.61	1163.46	1175.37	1180.85	1188.52	1198.85
1210.77	1225.87	1228.83	1229.85	1235.49	1238.92
1245.38	1272.43	1287.04	1311.65	1330.44	1342.70
1343.50	1349.56	1359.49	1363.02	1379.39	1390.65
1427.63	1428.01	1428.47	1444.92	1446.87	1448.25
1459.68	1473.66	1488.06	1491.29	1496.45	1499.24
1499.37	1503.45	1503.49	1505.74	1512.15	1524.01
1527.97	1535.15	1536.61	1578.02	1623.66	1624.51
1642.70	1649.89	1650.49	3040.24	3045.31	3045.90
3047.94	3062.90	3064.17	3105.80	3109.87	3110.38
3110.77	3129.89	3134.08	3138.32	3139.77	3143.17
3145.72	3150.71	3167.93	3194.06	3194.97	3195.73
3196.51	3215.11	3215.61	3221.16	3224.77	3226.52
3230.55	3238.56	3265.21			

==============================

***p*-B-TS**

==============================

-215.85	12.59	19.59	23.36	26.02	31.07
35.36	36.90	41.53	54.80	61.38	63.28
65.58	72.48	84.76	105.68	121.59	145.00
149.79	151.48	173.55	178.30	200.37	210.02
221.79	245.59	254.80	269.78	275.04	278.54
285.81	297.73	310.85	326.23	326.72	334.96
347.06	358.62	400.99	410.27	414.58	415.25
432.11	439.49	443.74	474.88	480.49	483.94
499.11	529.89	535.54	557.00	567.48	580.04
625.51	644.12	644.54	649.95	657.14	681.71
693.33	706.95	708.49	746.47	785.70	803.89
816.15	817.26	818.54	823.64	829.87	832.41
842.91	855.18	856.75	896.80	909.73	951.51
967.75	976.57	978.98	988.73	990.74	1014.37
1022.90	1023.21	1030.53	1030.74	1037.35	1041.25
1051.90	1064.10	1064.62	1066.64	1082.22	1089.04
1093.51	1099.72	1130.08	1143.38	1144.37	1151.60
1156.95	1158.67	1171.02	1174.96	1185.07	1196.98
1218.88	1225.57	1226.11	1236.90	1237.12	1241.61
1245.83	1271.54	1289.85	1309.22	1328.35	1341.95
1342.98	1347.42	1358.29	1361.71	1366.39	1390.05
1428.19	1428.27	1428.90	1444.61	1446.15	1446.27
1462.28	1470.23	1490.98	1496.81	1499.18	1499.74
1503.40	1503.52	1504.78	1510.32	1517.53	1525.41
1526.04	1534.74	1535.75	1546.63	1625.61	1626.36
1650.03	1651.05	1679.18	3029.33	3045.72	3045.85
3052.16	3063.98	3065.18	3080.11	3110.27	3110.33
3129.47	3135.97	3138.80	3139.01	3144.63	3145.11
3150.09	3184.93	3187.78	3193.49	3193.74	3194.62
3196.51	3218.14	3219.15	3221.80	3225.03	3228.93
3237.63	3273.90	3277.92			

==============================

***o*-C**

==============================

13.85	19.59	25.39	32.59	34.01	41.05
51.40	53.38	57.09	63.07	65.05	68.86
74.41	83.72	101.60	102.20	138.45	144.48
155.20	172.27	179.76	202.66	207.29	220.07
234.36	249.12	262.98	271.07	276.39	280.19
305.84	313.97	317.35	322.64	329.09	351.11
356.97	396.51	414.53	415.64	426.90	432.16
437.38	464.29	474.35	482.26	494.97	516.66
518.53	541.89	556.77	570.79	585.42	590.80
619.26	641.59	644.18	658.80	671.88	689.80
704.88	707.75	731.69	754.54	787.38	810.97
816.33	819.96	834.30	834.88	841.59	854.77
857.65	861.07	906.30	931.25	960.02	973.71
980.97	984.17	989.51	1000.88	1001.93	1023.30
1023.86	1026.37	1028.82	1030.64	1039.80	1048.46
1063.47	1064.42	1067.82	1072.18	1085.44	1086.33
1119.01	1127.98	1141.77	1144.74	1148.96	1157.00
1164.20	1175.87	1184.60	1187.06	1192.70	1210.83
1217.11	1225.59	1229.42	1236.86	1238.38	1239.96
1267.59	1282.29	1306.08	1321.06	1330.37	1342.16
1349.16	1359.12	1364.66	1366.24	1382.55	1392.13
1421.91	1427.48	1428.57	1445.36	1449.40	1450.52
1453.40	1463.94	1472.17	1496.31	1497.04	1499.13
1499.57	1503.47	1503.58	1512.97	1513.52	1522.28
1534.84	1535.55	1539.11	1542.05	1616.06	1622.19
1625.36	1648.26	1650.88	2938.83	2996.20	3036.84
3045.31	3045.59	3047.90	3052.02	3063.17	3094.94
3110.36	3110.40	3139.28	3139.61	3140.57	3142.28
3146.85	3161.01	3170.60	3193.77	3194.10	3196.84
3201.43	3216.23	3217.08	3219.15	3223.37	3230.56
3232.04	3235.18	3246.54			

==============================

***p*-C**

==============================

14.64	17.02	26.78	34.78	35.19	39.49
43.86	51.56	52.72	54.59	60.30	61.93
67.08	86.00	102.52	125.55	148.14	156.99
167.33	172.10	190.48	208.10	211.25	219.36
254.15	265.42	269.74	277.15	280.42	288.03
301.03	311.06	323.24	326.80	334.39	351.82
364.42	391.43	409.60	414.33	415.03	433.39
436.88	468.09	471.71	480.35	497.26	510.63
527.82	539.59	558.55	567.71	584.82	603.72
635.85	643.34	644.60	664.62	667.26	685.21
706.99	707.86	747.66	761.31	772.18	796.98
817.19	818.94	829.42	830.27	833.51	835.19
854.35	856.69	901.53	913.67	935.11	953.76
964.27	977.42	979.29	988.67	991.77	1022.87
1022.92	1026.57	1029.86	1030.87	1043.17	1045.82
1052.76	1063.55	1064.25	1070.04	1082.41	1087.58
1127.00	1137.44	1142.70	1143.68	1148.52	1151.38
1156.69	1157.61	1159.37	1183.31	1189.47	1208.45
1225.21	1226.19	1227.84	1237.17	1237.24	1248.67

1273.02 1276.01 1288.52 1309.44 1319.46 1333.10
1341.78 1343.54 1358.17 1362.48 1368.67 1385.16
1415.42 1423.39 1427.86 1428.55 1444.98 1446.43
1450.12 1462.54 1464.27 1469.50 1496.78 1498.32
1499.39 1503.30 1503.37 1504.50 1511.59 1526.80
1532.86 1533.47 1535.57 1555.50 1624.01 1625.60
1648.17 1651.02 1679.02 2947.87 2986.05 3037.49
3042.24 3046.81 3047.86 3051.18 3063.02 3096.49
3111.16 3112.16 3139.16 3140.33 3142.39 3144.11
3146.32 3162.13 3181.22 3192.37 3194.40 3195.88
3197.83 3218.12 3219.72 3222.11 3225.77 3230.44
3240.25 3277.26 3280.49

==============================

D

==============================

9.72 13.02 14.46 18.59 24.04 29.21
32.74 43.33 45.71 54.66 56.11 60.23
70.01 79.24 96.03 136.84 145.50 147.94
163.55 169.85 173.95 186.32 192.29 217.52
239.04 255.81 266.22 268.16 270.39 280.96
297.82 312.23 322.06 327.23 334.91 346.84
364.94 379.00 416.97 418.01 420.76 430.78
435.20 462.58 473.87 483.65 486.14 500.91
524.16 540.48 554.96 558.30 579.70 606.48
643.94 646.23 648.48 649.46 660.51 674.99
706.53 713.27 719.51 732.79 738.23 761.95
810.10 812.81 820.92 829.89 834.86 840.66
857.29 860.34 863.00 921.54 936.21 939.23
973.89 977.79 984.70 987.30 1002.34 1016.11
1020.83 1023.99 1033.13 1035.68 1049.71 1061.70
1065.03 1066.13 1086.10 1088.50 1110.78 1114.16
1133.50 1135.07 1149.22 1149.50 1150.21 1153.39
1184.65 1192.08 1198.69 1208.26 1220.01 1220.49
1223.30 1234.89 1235.01 1250.33 1255.76 1287.08
1307.00 1309.54 1319.73 1333.69 1337.89 1340.88
1354.80 1357.06 1369.85 1383.71 1398.71 1415.47
1427.88 1428.02 1441.83 1443.97 1448.76 1453.31
1460.95 1486.45 1496.14 1497.51 1501.86 1502.67
1504.67 1508.24 1513.15 1516.83 1531.39 1536.25
1538.15 1542.00 1628.29 1629.98 1653.71 1656.45
1674.42 2986.78 3006.27 3030.22 3039.29 3040.41
3043.38 3051.92 3089.19 3098.14 3101.97 3125.19
3126.92 3127.94 3130.20 3135.99 3156.71 3158.99
3178.24 3183.55 3185.19 3186.32 3187.62 3206.20
3212.47 3216.50 3218.26 3220.52 3252.52 3255.15

==============================

D-TS

==============================

-564.81 4.02 13.75 19.24 21.56 27.50
32.16 32.85 43.53 49.15 53.36 54.70
62.04 75.10 83.36 100.40 144.33 149.60
152.57 167.66 171.94 175.19 186.55 201.34
227.57 237.32 251.53 268.30 270.57 271.88
283.50 298.14 310.46 324.51 330.60 334.31
348.73 365.04 390.81 406.12 416.78 418.29
431.55 445.63 464.92 471.16 488.33 495.85
520.15 528.91 552.18 558.96 561.94 596.12
628.68 643.91 647.11 657.49 661.89 675.49
702.46 712.01 719.41 744.82 800.65 810.03
813.73 815.34 822.84 828.67 835.37 845.35
856.28 860.65 871.46 910.19 934.24 940.62
945.12 972.51 978.43 985.33 985.50 1016.70
1020.06 1030.37 1033.29 1035.79 1044.92 1064.58
1065.98 1067.34 1069.61 1082.34 1089.62 1119.54
1124.12 1131.26 1138.03 1149.19 1150.16 1152.64
1156.78 1167.00 1192.68 1196.93 1217.71 1220.49
1221.96 1233.97 1235.25 1249.12 1264.17 1297.80
1303.41 1310.92 1317.86 1338.17 1339.80 1343.27
1355.98 1356.39 1376.15 1396.68 1414.28 1427.52
1427.85 1442.40 1443.59 1447.66 1449.04 1457.81
1459.23 1495.07 1496.00 1497.53 1498.33 1502.18
1504.22 1507.89 1514.45 1517.14 1533.97 1535.87
1538.40 1547.60 1627.62 1629.83 1630.41 1654.08
1656.96 2989.10 3002.56 3030.56 3039.57 3041.15
3052.25 3060.70 3088.36 3099.30 3101.69 3102.16
3128.14 3128.52 3128.90 3135.78 3147.46 3158.02
3179.33 3183.33 3184.28 3184.46 3187.46 3204.18
3212.68 3216.73 3219.32 3220.84 3226.96 3231.23

==============================

E

==============================

10.55 32.78 64.40 73.11 138.19 164.25
175.29 275.15 277.09 282.81 340.05 364.22
415.54 433.66 457.16 518.96 562.50 630.91
646.82 701.95 717.94 821.76 831.72 856.45
976.32 977.19 984.01 1017.26 1034.55 1039.10
1066.07 1084.56 1139.50 1153.51 1158.37 1223.31
1235.81 1288.27 1340.34 1357.18 1401.62 1427.68
1444.67 1477.56 1486.15 1497.05 1507.80 1540.05
1631.49 1657.68 2997.87 3041.93 3055.42 3100.48
3130.02 3141.23 3183.52 3188.30 3221.03 3222.53

==============================

4w

==============================

12.22 16.63 27.45 32.58 38.14 46.80
66.59 78.69 79.81 149.25 160.88 174.87
189.64 237.23 245.14 271.42 273.05 299.97
324.36 334.10 349.31 390.69 416.73 431.21
451.88 478.85 504.75 509.66 555.92 582.47
620.96 645.13 669.94 683.12 707.71 733.49
771.29 815.61 827.48 834.59 838.84 842.80
859.77 882.22 905.80 940.77 977.90 985.18
985.67 1005.15 1011.38 1016.61 1032.04 1035.61
1066.31 1074.34 1088.17 1097.19 1116.56 1124.80
1140.10 1150.86 1160.28 1191.40 1197.53 1220.86
1229.09 1234.88 1254.04 1268.14 1301.69 1306.42
1309.52 1338.67 1347.88 1356.37 1373.03 1389.55
1417.95 1427.90 1442.53 1450.54 1452.61 1462.26
1496.25 1497.12 1507.73 1516.54 1535.04 1537.18
1538.24 1622.79 1630.17 1652.10 1656.73 2992.32
3017.11 3032.40 3041.65 3053.83 3090.87 3099.81
3128.66 3129.94 3139.77 3160.14 3161.93 3163.43
3180.63 3184.91 3186.28 3213.04 3217.88 3218.14

==============================

Ts-

==============================

53.97 66.34 92.62 157.69 219.56 269.15
320.77 403.73 413.53 426.88 461.60 572.31
589.94 648.63 728.08 801.37 830.35 857.83
965.93 972.99 985.85 1008.16 1033.19 1060.20
1077.31 1094.80 1133.64 1199.66 1229.09 1317.19
1346.69 1419.73 1430.72 1500.80 1508.06 1521.91
1619.59 1645.79 3008.33 3063.83 3094.88 3125.19
3126.38 3177.15 3177.72

==============================

NTsMe-

==============================

39.75 52.78 70.35 125.05 165.42 184.46
220.96 267.37 272.93 324.03 352.47 396.79
420.55 465.08 522.62 549.57 573.26 633.92
648.30 716.66 795.65 825.82 838.61 854.35
964.13 976.02 1009.19 1036.65 1059.95 1061.60
1106.55 1137.05 1139.13 1142.30 1178.28 1209.42
1228.93 1232.83 1326.64 1349.71 1420.37 1435.97
1457.46 1499.83 1500.25 1508.67 1518.38 1531.06
1619.73 1653.28 2876.63 2965.49 3013.85 3029.93
3067.85 3100.15 3141.25 3143.92 3205.63 3209.25

==============================

NHTsMe

==============================

30.94 35.96 57.24 104.01 164.65 188.09
224.48 242.96 284.53 323.53 354.85 391.71
416.86 453.17 485.05 516.90 545.77 616.42
643.13 648.15 714.59 806.23 827.02 841.61
857.71 972.43 985.67 1020.12 1035.57 1064.56
1066.47 1094.37 1139.27 1152.01 1153.99 1165.46
1222.79 1237.17 1316.89 1340.15 1356.96 1412.04
1428.35 1444.03 1467.77 1500.43 1502.87 1506.22
1526.34 1540.24 1631.02 1657.91 3039.71 3043.12
3100.58 3117.92 3130.42 3139.62 3182.72 3185.21
3219.44 3222.46 3539.60

==============================

amisyl-TS

==============================

-217.92 27.17 36.07 46.91 52.58 57.44
67.65 109.12 137.21 149.21 167.20 178.97
221.51 232.04 257.97 269.66 275.08 284.68
325.92 330.98 367.63 395.56 416.93 439.45
462.42 498.83 505.72 535.30 555.25 638.99
647.62 701.55 714.32 792.89 817.97 830.30
837.21 855.62 857.33 927.46 973.00 978.83
982.62 1006.30 1019.32 1033.74 1051.14 1064.20
1072.42 1089.67 1119.39 1128.70 1136.96 1150.72
1155.55 1184.91 1220.91 1235.60 1253.67 1276.63
1318.10 1324.25 1338.61 1354.73 1402.16 1427.03
1431.31 1442.93 1445.61 1460.97 1494.68 1497.18
1500.92 1501.76 1504.88 1516.90 1532.61 1537.91
1616.05 1628.65 1656.05 3002.05 3028.14 3038.74

3055.13	3067.34	3099.91	3105.08	3116.44	3127.02
3133.23	3138.04	3180.24	3181.04	3185.85	3195.87
3219.23	3220.50	3306.55			

==============================

amidyl-adduct

==============================

19.70	36.48	47.37	52.62	54.32	64.88
81.67	138.91	150.13	170.27	178.27	209.74
235.54	255.15	265.10	280.89	303.10	327.73
348.48	374.40	398.43	418.02	434.56	461.23
490.50	514.52	539.22	554.34	617.65	648.20
659.28	714.55	741.96	819.81	831.41	833.48
855.97	860.98	907.33	975.85	978.26	985.51
1020.52	1035.87	1064.41	1065.88	1093.81	1100.17
1129.23	1147.68	1152.80	1154.16	1178.43	1185.33
1221.91	1223.63	1237.38	1264.16	1305.03	1314.00
1329.06	1340.29	1357.50	1380.12	1400.21	1427.26
1430.21	1443.91	1469.24	1470.73	1484.75	1497.62
1502.95	1503.30	1514.02	1516.12	1521.81	1534.18
1539.22	1629.16	1658.42	2997.56	3005.20	3007.38
3040.22	3048.04	3055.83	3102.07	3128.29	3129.87
3130.25	3133.10	3137.18	3144.56	3178.81	3182.14
3182.89	3218.95	3220.48			

==============================

D1

==============================

20.60	26.31	37.33	44.48	61.78	88.15
146.51	168.42	193.09	198.80	228.11	262.29
268.53	300.10	320.18	346.41	356.63	415.89
421.09	435.67	468.27	482.34	509.10	542.99
556.64	605.63	626.70	638.35	644.08	647.00
672.92	700.35	714.33	735.37	761.09	817.13
829.23	857.35	930.06	930.82	972.95	985.19
1005.45	1009.09	1019.22	1034.09	1059.42	1064.76
1087.42	1098.09	1116.71	1135.05	1150.17	1152.55
1201.59	1216.87	1221.98	1231.54	1236.02	1319.43
1330.55	1339.81	1349.12	1356.76	1381.47	1427.95
1444.30	1461.90	1471.26	1499.50	1502.52	1506.66
1512.33	1536.68	1551.35	1628.17	1653.80	1662.72
3040.31	3041.64	3100.82	3122.56	3130.35	3154.49
3182.36	3184.58	3196.89	3200.25	3217.08	3220.05
3228.47	3252.89	3255.92			

==============================

D1-TS

==============================

-581.27	24.41	38.87	40.48	48.36	67.48
80.06	150.87	170.09	197.97	212.83	236.85
251.92	273.17	305.42	323.64	347.67	391.33
401.81	416.22	429.51	473.61	495.02	526.53
555.86	572.79	636.16	645.42	647.21	664.50
675.62	713.66	730.45	813.14	817.06	828.46
849.84	856.09	908.79	945.60	962.83	972.69
982.39	1018.24	1030.62	1034.72	1052.32	1064.62
1072.83	1084.84	1096.93	1133.75	1151.67	1157.87
1163.57	1172.57	1221.02	1221.41	1235.75	1308.53
1318.52	1338.60	1356.47	1368.00	1427.66	1443.89
1447.40	1460.71	1482.19	1496.71	1499.81	1506.30
1514.93	1536.22	1558.06	1619.75	1627.92	1655.04
3029.08	3038.93	3099.16	3100.44	3129.06	3145.82
3180.56	3183.39	3194.23	3199.98	3218.53	3219.26
3220.21	3228.67	3231.51			

==============================

pyridine

==============================

385.71	420.35	613.29	668.66	719.16	763.89
898.89	958.22	998.52	1011.25	1014.40	1050.10
1085.56	1099.40	1182.63	1251.53	1309.78	1389.12
1482.02	1524.02	1636.71	1642.58	3161.91	3164.33
3187.17	3203.79	3212.35			

Appendix I Ⅰ I

Crystallographic Data Obtained in This Study

Crystallographic Data for 4m (CCDC : 2057463)

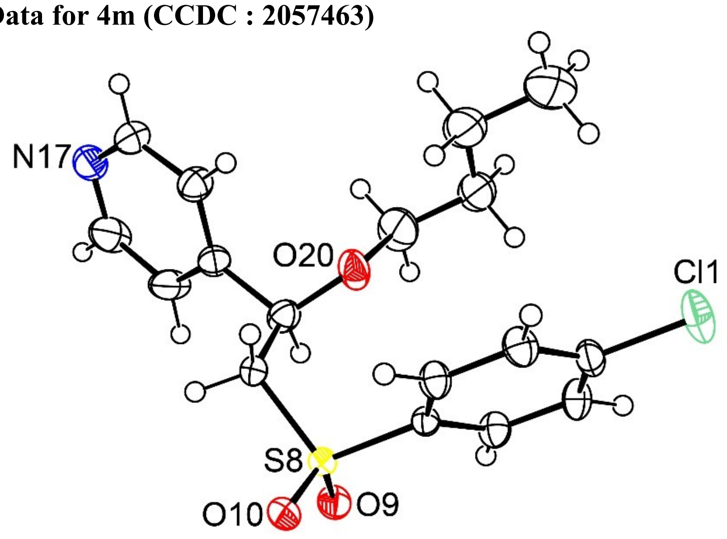

ORTEP representation (50% probability) of the crystal structure of **4m**

Table 1. Crystal data and structure refinement for **4m**.

Empirical formula	C_{17} H_{20} N O_3 S Cl	
Formula weight	353.85	
Temperature	173(2) K	
Wavelength	0.71073 Å	
Crystal system	Monoclinic	
Space group	$P2_1/n$	
Unit cell dimensions	a = 6.8411(2) Å	α = 90°
	b = 15.3599(4) Å	β = 100.7506(11)°
	c = 17.0637(5) Å	γ = 90°
Volume	1761.56(9) $Å^3$	
Z	4	
Density (calculated)	1.334 Mg/m^3	
Absorption coefficient	0.349 mm^{-1}	
F(000)	744	
Crystal size	0.142 x 0.078 x 0.054 mm^3	
Theta range for data collection	3.324 to 27.113°.	
Index ranges	–8<=h<=8, –19<=k<=19, –21<=l<=21	
Reflections collected	29939	
Independent reflections	3858 [R(int) = 0.0285]	
Completeness to theta = 25.242°	99.0 %	
Absorption correction	Semi-empirical from equivalents	
Max. and min. transmission	0.7455 and 0.7026	

Refinement method	Full-matrix least-squares on F^2
Data / restraints / parameters	3858 / 24 / 237
Goodness-of-fit on F^2	1.059
Final R indices [I>2sigma(I)]	R1 = 0.0345, wR2 = 0.0840
R indices (all data)	R1 = 0.0418, wR2 = 0.0885
Largest diff. peak and hole	0.330 and –0.348 e·Å^{-3}

Table 2. Atomic coordinates (x 10^4) and equivalent isotropic displacement parameters ($Å^2$ x 10^3) for **4m**. U(eq) is defined as one third of the trace of the orthogonalized U^{ij} tensor.

	x	y	z	U(eq)
Cl(1)	9539(1)	4969(1)	8868(1)	46(1)
C(2)	7596(2)	4997(1)	8051(1)	28(1)
C(3)	8034(2)	4832(1)	7305(1)	30(1)
C(4)	6527(2)	4861(1)	6641(1)	26(1)
C(5)	4602(2)	5047(1)	6742(1)	21(1)
C(6)	4170(2)	5206(1)	7490(1)	27(1)
C(7)	5689(2)	5186(1)	8156(1)	31(1)
S(8)	2693(1)	5093(1)	5889(1)	21(1)
O(9)	885(1)	5359(1)	6144(1)	29(1)
O(10)	3385(2)	5608(1)	5289(1)	27(1)
C(12)	2334(2)	4013(1)	5503(1)	23(1)
C(13)	1542(2)	3384(1)	6061(1)	29(1)
C(14)	688(2)	2571(1)	5615(1)	28(1)
C(15)	-1331(2)	2491(1)	5333(1)	40(1)
C(16)	-2051(3)	1739(1)	4931(1)	46(1)
N(17)	-913(2)	1073(1)	4796(1)	41(1)
C(18)	1029(3)	1159(1)	5073(1)	37(1)
C(19)	1892(2)	1885(1)	5480(1)	32(1)
O(20)	3166(2)	3199(1)	6681(1)	38(1)
C(21)	2636(4)	2745(1)	7348(1)	51(1)
C(22)	4434(4)	2697(2)	7997(1)	53(1)
C(23)	6186(5)	2263(2)	7749(1)	60(1)
C(24)	8028(13)	2230(4)	8410(4)	70(2)
C(22B)	4670(20)	2197(10)	7751(9)	33(4)
C(23B)	6450(20)	2792(10)	8028(9)	34(4)
C(24B)	8250(70)	2430(30)	8460(20)	44(7)

Table 3. Bond lengths [Å] and angles [°] for **4m**.

Cl(1)-C(2)	1.7381(14)
C(2)-C(7)	1.380(2)
C(2)-C(3)	1.385(2)
C(3)-C(4)	1.383(2)
C(3)-H(3)	0.9500
C(4)-C(5)	1.3887(19)
C(4)-H(4)	0.9500
C(5)-C(6)	1.3850(19)
C(5)-S(8)	1.7663(13)
C(6)-C(7)	1.390(2)
C(6)-H(6)	0.9500
C(7)-H(7)	0.9500
S(8)-O(10)	1.4411(10)
S(8)-O(9)	1.4442(10)
S(8)-C(12)	1.7847(13)
C(12)-C(13)	1.5244(19)
C(12)-H(12A)	0.9900
C(12)-H(12B)	0.9900
C(13)-O(20)	1.4117(18)
C(13)-C(14)	1.522(2)
C(13)-H(13)	0.993(19)
C(14)-C(15)	1.381(2)
C(14)-C(19)	1.382(2)
C(15)-C(16)	1.387(2)
C(15)-H(15)	0.9500
C(16)-N(17)	1.331(2)
C(16)-H(16)	0.9500
N(17)-C(18)	1.331(2)
C(18)-C(19)	1.386(2)
C(18)-H(18)	0.9500
C(19)-H(19)	0.9500
O(20)-C(21)	1.437(2)
C(21)-C(22)	1.495(3)
C(21)-C(22B)	1.662(15)
C(21)-H(21A)	0.99(3)

C(21)-H(21B)	0.99(3)
C(22)-C(23)	1.499(4)
C(22)-H(22A)	0.9900
C(22)-H(22B)	0.9900
C(23)-C(24)	1.527(8)
C(23)-H(23A)	0.9900
C(23)-H(23B)	0.9900
C(24)-H(24A)	0.9800
C(24)-H(24B)	0.9800
C(24)-H(24C)	0.9800
C(22B)-C(23B)	1.53(2)
C(22B)-H(22C)	0.9900
C(22B)-H(22D)	0.9900
C(23B)-C(24B)	1.42(5)
C(23B)-H(23C)	0.9900
C(23B)-H(23D)	0.9900
C(24B)-H(24D)	0.9800
C(24B)-H(24E)	0.9800
C(24B)-H(24F)	0.9800

C(7)-C(2)-C(3)	121.98(13)
C(7)-C(2)-Cl(1)	120.08(11)
C(3)-C(2)-Cl(1)	117.95(12)
C(4)-C(3)-C(2)	119.42(14)
C(4)-C(3)-H(3)	120.3
C(2)-C(3)-H(3)	120.3
C(3)-C(4)-C(5)	118.93(13)
C(3)-C(4)-H(4)	120.5
C(5)-C(4)-H(4)	120.5
C(6)-C(5)-C(4)	121.44(13)
C(6)-C(5)-S(8)	119.99(11)
C(4)-C(5)-S(8)	118.57(10)
C(5)-C(6)-C(7)	119.58(13)
C(5)-C(6)-H(6)	120.2
C(7)-C(6)-H(6)	120.2
C(2)-C(7)-C(6)	118.65(13)
C(2)-C(7)-H(7)	120.7

C(6)-C(7)-H(7)	120.7
O(10)-S(8)-O(9)	118.10(6)
O(10)-S(8)-C(5)	108.44(6)
O(9)-S(8)-C(5)	107.86(6)
O(10)-S(8)-C(12)	106.81(6)
O(9)-S(8)-C(12)	107.94(6)
C(5)-S(8)-C(12)	107.24(6)
C(13)-C(12)-S(8)	113.49(10)
C(13)-C(12)-H(12A)	108.9
S(8)-C(12)-H(12A)	108.9
C(13)-C(12)-H(12B)	108.9
S(8)-C(12)-H(12B)	108.9
H(12A)-C(12)-H(12B)	107.7
O(20)-C(13)-C(14)	112.59(12)
O(20)-C(13)-C(12)	105.88(12)
C(14)-C(13)-C(12)	110.78(12)
O(20)-C(13)-H(13)	110.5(11)
C(14)-C(13)-H(13)	108.0(11)
C(12)-C(13)-H(13)	109.0(11)
C(15)-C(14)-C(19)	117.62(14)
C(15)-C(14)-C(13)	120.80(14)
C(19)-C(14)-C(13)	121.58(14)
C(14)-C(15)-C(16)	119.04(16)
C(14)-C(15)-H(15)	120.5
C(16)-C(15)-H(15)	120.5
N(17)-C(16)-C(15)	124.11(16)
N(17)-C(16)-H(16)	117.9
C(15)-C(16)-H(16)	117.9
C(18)-N(17)-C(16)	116.07(14)
N(17)-C(18)-C(19)	124.15(15)
N(17)-C(18)-H(18)	117.9
C(19)-C(18)-H(18)	117.9
C(14)-C(19)-C(18)	119.00(15)
C(14)-C(19)-H(19)	120.5
C(18)-C(19)-H(19)	120.5
C(13)-O(20)-C(21)	114.29(14)
O(20)-C(21)-C(22)	108.49(17)

O(20)-C(21)-C(22B)	105.0(5)
O(20)-C(21)-H(21A)	107.3(15)
C(22)-C(21)-H(21A)	111.2(14)
C(22B)-C(21)-H(21A)	138.9(15)
O(20)-C(21)-H(21B)	107.8(14)
C(22)-C(21)-H(21B)	113.5(14)
C(22B)-C(21)-H(21B)	84.8(15)
H(21A)-C(21)-H(21B)	108(2)
C(21)-C(22)-C(23)	113.78(19)
C(21)-C(22)-H(22A)	108.8
C(23)-C(22)-H(22A)	108.8
C(21)-C(22)-H(22B)	108.8
C(23)-C(22)-H(22B)	108.8
H(22A)-C(22)-H(22B)	107.7
C(22)-C(23)-C(24)	113.5(4)
C(22)-C(23)-H(23A)	108.9
C(24)-C(23)-H(23A)	108.9
C(22)-C(23)-H(23B)	108.9
C(24)-C(23)-H(23B)	108.9
H(23A)-C(23)-H(23B)	107.7
C(23)-C(24)-H(24A)	109.5
C(23)-C(24)-H(24B)	109.5
H(24A)-C(24)-H(24B)	109.5
C(23)-C(24)-H(24C)	109.5
H(24A)-C(24)-H(24C)	109.5
H(24B)-C(24)-H(24C)	109.5
C(23B)-C(22B)-C(21)	112.5(11)
C(23B)-C(22B)-H(22C)	109.1
C(21)-C(22B)-H(22C)	109.1
C(23B)-C(22B)-H(22D)	109.1
C(21)-C(22B)-H(22D)	109.1
H(22C)-C(22B)-H(22D)	107.8
C(24B)-C(23B)-C(22B)	119(2)
C(24B)-C(23B)-H(23C)	107.5
C(22B)-C(23B)-H(23C)	107.5
C(24B)-C(23B)-H(23D)	107.5
C(22B)-C(23B)-H(23D)	107.5

H(23C)-C(23B)-H(23D)	107.0
C(23B)-C(24B)-H(24D)	109.5
C(23B)-C(24B)-H(24E)	109.5
H(24D)-C(24B)-H(24E)	109.5
C(23B)-C(24B)-H(24F)	109.5
H(24D)-C(24B)-H(24F)	109.5
H(24E)-C(24B)-H(24F)	109.5

Symmetry transformations used to generate equivalent atoms:

Table 4. Anisotropic displacement parameters ($Å^2 \times 10^3$) for **4m**. The anisotropic displacement factor exponent takes the form: $-2\pi^2[h^2a^{*2}U^{11} + ... + 2 h k a^* b^* U^{12}]$

	U^{11}	U^{22}	U^{33}	U^{23}	U^{13}	U^{12}
Cl(1)	38(1)	65(1)	30(1)	3(1)	-11(1)	-6(1)
C(2)	28(1)	30(1)	22(1)	2(1)	-3(1)	-5(1)
C(3)	22(1)	38(1)	29(1)	0(1)	3(1)	0(1)
C(4)	24(1)	33(1)	22(1)	-1(1)	6(1)	0(1)
C(5)	22(1)	20(1)	19(1)	0(1)	1(1)	-2(1)
C(6)	25(1)	33(1)	24(1)	-3(1)	7(1)	1(1)
C(7)	34(1)	40(1)	19(1)	-5(1)	5(1)	-3(1)
S(8)	21(1)	20(1)	19(1)	0(1)	2(1)	1(1)
O(9)	23(1)	33(1)	30(1)	-2(1)	3(1)	6(1)
O(10)	34(1)	25(1)	23(1)	4(1)	3(1)	0(1)
C(12)	26(1)	22(1)	21(1)	-2(1)	0(1)	-1(1)
C(13)	33(1)	24(1)	30(1)	-2(1)	7(1)	-4(1)
C(14)	33(1)	22(1)	29(1)	1(1)	4(1)	-4(1)
C(15)	33(1)	27(1)	56(1)	2(1)	-1(1)	3(1)
C(16)	37(1)	34(1)	60(1)	4(1)	-12(1)	-5(1)
N(17)	53(1)	27(1)	37(1)	2(1)	-7(1)	-7(1)
C(18)	48(1)	27(1)	35(1)	-2(1)	6(1)	2(1)
C(19)	31(1)	31(1)	35(1)	-3(1)	5(1)	-1(1)
O(20)	52(1)	34(1)	24(1)	7(1)	-1(1)	-13(1)
C(21)	79(2)	42(1)	33(1)	10(1)	14(1)	-10(1)
C(22)	96(2)	37(1)	25(1)	6(1)	8(1)	5(1)
C(23)	100(2)	42(1)	36(1)	3(1)	10(1)	14(1)
C(24)	99(4)	47(3)	62(3)	10(2)	5(2)	11(2)
C(24B)	57(12)	48(16)	21(9)	23(9)	-7(8)	2(10)

Table 5. Hydrogen coordinates (x 10^4) and isotropic displacement parameters ($Å^2$ x 10^3) for **4m**.

	x	y	z	U(eq)
H(3)	9358	4699	7249	36
H(4)	6803	4757	6123	32
H(6)	2843	5329	7548	32
H(7)	5421	5300	8673	38
H(12A)	1389	4031	4988	28
H(12B)	3618	3789	5399	28
H(13)	460(30)	3673(12)	6277(11)	43
H(15)	-2215	2946	5414	48
H(16)	-3444	1696	4740	56
H(18)	1878	694	4985	44
H(19)	3290	1911	5663	39
H(21A)	1550(40)	3081(17)	7522(15)	76
H(21B)	2100(40)	2165(18)	7157(14)	76
H(22A)	4085	2373	8454	64
H(22B)	4818	3294	8181	64
H(23A)	6521	2580	7286	72
H(23B)	5810	1662	7574	72
H(24A)	9109	1934	8212	105
H(24B)	7715	1910	8868	105
H(24C)	8441	2823	8574	105
H(22C)	4394	1859	8213	40
H(22D)	5002	1778	7355	40
H(23C)	6779	3080	7549	41
H(23D)	6025	3255	8363	41
H(24D)	9365	2815	8425	66
H(24E)	8479	1858	8237	66
H(24F)	8135	2358	9023	66

Crystallographic Data for 5e (CCDC : 2057471)

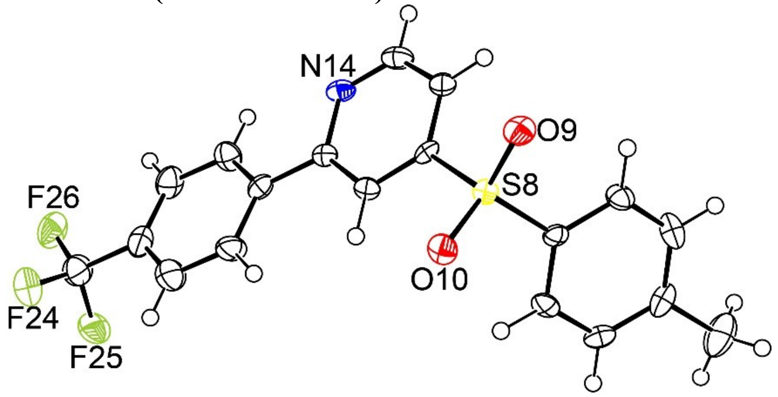

ORTEP representation (50% probability) of the crystal structure of **5e**

Table 1. Crystal data and structure refinement for **5e**.

Empirical formula	$C_{19} H_{14} NO_2 F_3 S$	
Formula weight	377.37	
Temperature	173(2) K	
Wavelength	0.71073 Å	
Crystal system	Monoclinic	
Space group	*P*2$_1$/*c*	
Unit cell dimensions	a = 15.1971(18) Å	α = 90°
	b = 15.1809(16) Å	β = 97.371(4)°
	c = 7.5348(9) Å	γ = 90°
Volume	1724.0(3) Å^3	
Z	4	
Density (calculated)	1.454 Mg/m^3	
Absorption coefficient	0.232 mm^{-1}	
F(000)	776	
Crystal size	0.134 x 0.054 x 0.032 mm^3	
Theta range for data collection	3.005 to 26.474°.	
Index ranges	–19<=h<=18, –18<=k<=19, –9<=l<=9	
Reflections collected	40338	
Independent reflections	3551 [R(int) = 0.1085]	
Completeness to theta = 25.242°	99.5 %	
Absorption correction	Semi-empirical from equivalents	
Max. and min. transmission	0.7454 and 0.5838	
Refinement method	Full-matrix least-squares on F^2	
Data / restraints / parameters	3551 / 421 / 311	

Goodness-of-fit on F^2	1.210
Final R indices [I>2sigma(I)]	R1 = 0.1108, wR2 = 0.1984
R indices (all data)	R1 = 0.1277, wR2 = 0.2055
Largest diff. peak and hole	0.558 and –0.405 e·Å^{-3}

Table 2. Atomic coordinates (x 10^4) and equivalent isotropic displacement parameters ($Å^2$ x 10^3) for **5e**. U(eq) is defined as one third of the trace of the orthogonalized U^{ij} tensor.

	x	y	z	U(eq)
C(1)	8335(5)	4341(6)	11187(9)	53(2)
C(2)	7748(4)	4192(4)	9414(7)	30(1)
C(3)	7985(4)	3579(4)	8193(8)	32(1)
C(4)	7480(3)	3455(4)	6574(7)	25(1)
C(5)	6713(3)	3954(3)	6146(7)	19(1)
C(6)	6454(3)	4555(3)	7363(7)	23(1)
C(7)	6978(4)	4675(3)	8986(7)	26(1)
S(8)	6072(1)	3805(1)	4060(2)	18(1)
O(9)	6627(2)	3417(3)	2880(5)	28(1)
O(10)	5621(2)	4618(2)	3562(5)	26(1)
C(11)	5253(3)	3011(3)	4430(6)	16(1)
C(12)	5449(4)	2129(3)	4301(7)	24(1)
C(13)	4794(4)	1537(4)	4551(7)	28(1)
N(14)	3985(3)	1758(3)	4879(6)	24(1)
C(15)	3794(3)	2623(3)	5003(6)	19(1)
C(16)	4426(3)	3267(3)	4801(6)	19(1)
C(17)	2870(3)	2830(3)	5295(7)	22(1)
C(18)	2546(4)	3686(4)	5174(8)	33(1)
C(19)	1675(4)	3869(5)	5374(9)	40(2)
C(20)	1111(4)	3206(4)	5725(8)	32(1)
C(21)	1427(4)	2348(4)	5875(8)	35(1)
C(22)	2294(4)	2171(4)	5683(8)	30(1)
C(23)	164(19)	3418(16)	5930(40)	38(2)
F(24)	-225(6)	3918(11)	4626(14)	52(2)
F(25)	133(7)	3880(9)	7505(16)	43(2)
F(26)	-331(6)	2700(6)	6080(20)	45(2)
C(23B)	200(60)	3330(40)	6040(110)	41(3)
F(24B)	-320(12)	3422(18)	4410(30)	49(3)
F(25B)	70(15)	4090(14)	6850(30)	44(3)
F(26B)	-196(15)	2698(15)	6860(30)	47(3)
C(23C)	160(20)	3489(17)	5940(40)	42(3)
F(24C)	-133(15)	4254(15)	5270(40)	45(4)

F(25C)	110(18)	3510(20)	7760(20)	46(4)
F(26C)	-413(17)	2867(16)	5300(40)	47(4)

Table 3. Bond lengths [Å] and angles [°] for **5e**.

C(1)-C(2)	1.525(8)
C(1)-H(1A)	0.9800
C(1)-H(1B)	0.9800
C(1)-H(1C)	0.9800
C(2)-C(7)	1.383(8)
C(2)-C(3)	1.388(8)
C(3)-C(4)	1.368(8)
C(3)-H(3)	0.9500
C(4)-C(5)	1.393(7)
C(4)-H(4)	0.9500
C(5)-C(6)	1.386(7)
C(5)-S(8)	1.755(5)
C(6)-C(7)	1.383(7)
C(6)-H(6)	0.9500
C(7)-H(7)	0.9500
S(8)-O(9)	1.430(4)
S(8)-O(10)	1.438(4)
S(8)-C(11)	1.780(5)
C(11)-C(16)	1.377(7)
C(11)-C(12)	1.378(7)
C(12)-C(13)	1.371(8)
C(12)-H(12)	0.9500
C(13)-N(14)	1.328(7)
C(13)-H(13)	0.9500
N(14)-C(15)	1.351(6)
C(15)-C(16)	1.393(7)
C(15)-C(17)	1.483(7)
C(16)-H(16)	0.9500
C(17)-C(22)	1.384(7)
C(17)-C(18)	1.389(7)
C(18)-C(19)	1.380(8)
C(18)-H(18)	0.9500
C(19)-C(20)	1.370(9)
C(19)-H(19)	0.9500
C(20)-C(21)	1.388(9)
C(20)-C(23B)	1.44(8)
C(20)-C(23)	1.50(3)

C(20)-C(23C)	1.53(3)
C(21)-C(22)	1.371(8)
C(21)-H(21)	0.9500
C(22)-H(22)	0.9500
C(23)-F(24)	1.32(2)
C(23)-F(26)	1.34(3)
C(23)-F(25)	1.38(3)
C(23B)-F(25B)	1.33(8)
C(23B)-F(26B)	1.33(8)
C(23B)-F(24B)	1.38(8)
C(23C)-F(24C)	1.32(2)
C(23C)-F(26C)	1.34(3)
C(23C)-F(25C)	1.38(3)

C(2)-C(1)-H(1A)	109.5
C(2)-C(1)-H(1B)	109.5
H(1A)-C(1)-H(1B)	109.5
C(2)-C(1)-H(1C)	109.5
H(1A)-C(1)-H(1C)	109.5
H(1B)-C(1)-H(1C)	109.5
C(7)-C(2)-C(3)	118.9(5)
C(7)-C(2)-C(1)	120.5(6)
C(3)-C(2)-C(1)	120.6(6)
C(4)-C(3)-C(2)	121.4(5)
C(4)-C(3)-H(3)	119.3
C(2)-C(3)-H(3)	119.3
C(3)-C(4)-C(5)	119.2(5)
C(3)-C(4)-H(4)	120.4
C(5)-C(4)-H(4)	120.4
C(6)-C(5)-C(4)	120.4(5)
C(6)-C(5)-S(8)	120.3(4)
C(4)-C(5)-S(8)	119.3(4)
C(7)-C(6)-C(5)	119.4(5)
C(7)-C(6)-H(6)	120.3
C(5)-C(6)-H(6)	120.3
C(6)-C(7)-C(2)	120.7(5)
C(6)-C(7)-H(7)	119.6
C(2)-C(7)-H(7)	119.6
O(9)-S(8)-O(10)	119.4(2)

O(9)-S(8)-C(5)	107.8(2)
O(10)-S(8)-C(5)	108.3(2)
O(9)-S(8)-C(11)	107.4(2)
O(10)-S(8)-C(11)	107.6(2)
C(5)-S(8)-C(11)	105.4(2)
C(16)-C(11)-C(12)	120.0(4)
C(16)-C(11)-S(8)	121.0(4)
C(12)-C(11)-S(8)	119.0(4)
C(13)-C(12)-C(11)	117.4(5)
C(13)-C(12)-H(12)	121.3
C(11)-C(12)-H(12)	121.3
N(14)-C(13)-C(12)	124.4(5)
N(14)-C(13)-H(13)	117.8
C(12)-C(13)-H(13)	117.8
C(13)-N(14)-C(15)	118.1(4)
N(14)-C(15)-C(16)	121.2(5)
N(14)-C(15)-C(17)	115.6(4)
C(16)-C(15)-C(17)	123.2(4)
C(11)-C(16)-C(15)	118.9(4)
C(11)-C(16)-H(16)	120.5
C(15)-C(16)-H(16)	120.5
C(22)-C(17)-C(18)	117.5(5)
C(22)-C(17)-C(15)	121.0(5)
C(18)-C(17)-C(15)	121.5(5)
C(19)-C(18)-C(17)	121.2(5)
C(19)-C(18)-H(18)	119.4
C(17)-C(18)-H(18)	119.4
C(20)-C(19)-C(18)	120.4(6)
C(20)-C(19)-H(19)	119.8
C(18)-C(19)-H(19)	119.8
C(19)-C(20)-C(21)	119.2(5)
C(19)-C(20)-C(23B)	125(3)
C(21)-C(20)-C(23B)	116(3)
C(19)-C(20)-C(23)	119.6(10)
C(21)-C(20)-C(23)	121.3(10)
C(19)-C(20)-C(23C)	115.7(11)
C(21)-C(20)-C(23C)	125.2(11)
C(22)-C(21)-C(20)	120.2(5)
C(22)-C(21)-H(21)	119.9

C(20)-C(21)-H(21)	119.9
C(21)-C(22)-C(17)	121.5(5)
C(21)-C(22)-H(22)	119.2
C(17)-C(22)-H(22)	119.2
F(24)-C(23)-F(26)	109(2)
F(24)-C(23)-F(25)	106.6(18)
F(26)-C(23)-F(25)	105.1(19)
F(24)-C(23)-C(20)	112.8(19)
F(26)-C(23)-C(20)	113.0(17)
F(25)-C(23)-C(20)	109.6(19)
F(25B)-C(23B)-F(26B)	108(6)
F(25B)-C(23B)-F(24B)	103(4)
F(26B)-C(23B)-F(24B)	104(6)
F(25B)-C(23B)-C(20)	113(6)
F(26B)-C(23B)-C(20)	119(5)
F(24B)-C(23B)-C(20)	109(6)
F(24C)-C(23C)-F(26C)	108(2)
F(24C)-C(23C)-F(25C)	107(2)
F(26C)-C(23C)-F(25C)	105(2)
F(24C)-C(23C)-C(20)	119(2)
F(26C)-C(23C)-C(20)	110(2)
F(25C)-C(23C)-C(20)	107(2)

Symmetry transformations used to generate equivalent atoms:

Table 4. Anisotropic displacement parameters (Å^2 x 10^3) for **5e**. The anisotropic displacement factor exponent takes the form: $-2\pi^2[h^2a^{*2}U^{11} + ... + 2 h k a^* b^* U^{12}]$

	U^{11}	U^{22}	U^{33}	U^{23}	U^{13}	U^{12}
C(1)	45(4)	76(5)	34(4)	-3(4)	-11(3)	-15(4)
C(2)	25(3)	39(3)	24(3)	1(2)	0(2)	-13(2)
C(3)	21(3)	44(4)	32(3)	8(3)	4(2)	8(2)
C(4)	27(3)	29(3)	21(3)	-3(2)	6(2)	5(2)
C(5)	21(2)	13(2)	23(3)	-1(2)	5(2)	-4(2)
C(6)	26(3)	12(2)	31(3)	6(2)	7(2)	2(2)
C(7)	36(3)	17(2)	25(3)	-2(2)	6(2)	-6(2)
S(8)	21(1)	19(1)	16(1)	2(1)	4(1)	0(1)
O(9)	31(2)	35(2)	18(2)	-5(2)	8(2)	-2(2)
O(10)	30(2)	21(2)	26(2)	9(2)	1(2)	2(2)
C(11)	24(2)	17(2)	5(2)	2(2)	-2(2)	-5(2)
C(12)	25(3)	27(3)	21(3)	-7(2)	4(2)	2(2)
C(13)	41(3)	18(3)	25(3)	0(2)	5(2)	4(2)
N(14)	33(3)	15(2)	23(2)	-1(2)	4(2)	-1(2)
C(15)	25(3)	18(2)	15(2)	1(2)	2(2)	1(2)
C(16)	24(3)	15(2)	19(2)	3(2)	4(2)	1(2)
C(17)	27(3)	24(3)	15(2)	0(2)	0(2)	-4(2)
C(18)	28(3)	22(3)	51(4)	-6(3)	11(3)	-5(2)
C(19)	30(3)	41(4)	50(4)	-5(3)	9(3)	1(3)
C(20)	23(3)	48(4)	25(3)	-6(3)	3(2)	-4(3)
C(21)	28(3)	34(3)	41(3)	5(3)	4(3)	-10(3)
C(22)	30(3)	28(3)	33(3)	7(2)	4(2)	2(2)
C(23)	31(3)	43(4)	40(4)	-4(3)	8(3)	-2(3)
F(24)	35(4)	59(5)	59(4)	1(4)	-5(3)	10(4)
F(25)	34(3)	46(5)	50(4)	-6(4)	15(4)	6(4)
F(26)	28(4)	58(4)	49(5)	-5(4)	6(4)	-12(3)
C(23B)	36(4)	45(4)	43(4)	-2(3)	6(3)	-1(4)
F(24B)	38(5)	55(6)	52(5)	1(5)	-3(4)	5(5)
F(25B)	36(5)	48(5)	50(6)	-2(5)	11(5)	5(5)
F(26B)	37(6)	58(5)	46(6)	3(5)	10(5)	-3(5)
C(23C)	36(4)	45(4)	44(4)	-2(4)	6(3)	0(3)
F(24C)	35(6)	46(5)	54(6)	-4(5)	4(5)	4(5)

F(25C)	37(6)	54(6)	50(5)	0(5)	12(5)	1(6)
F(26C)	37(6)	52(6)	50(6)	1(5)	2(6)	-7(5)

Table 5. Hydrogen coordinates (x 10^4) and isotropic displacement parameters ($Å^2$ x 10^3) for **5e**.

	x	y	z	U(eq)
H(1A)	8013	4698	11976	80
H(1B)	8877	4649	10969	80
H(1C)	8490	3772	11756	80
H(3)	8508	3239	8487	39
H(4)	7651	3033	5751	30
H(6)	5921	4882	7085	28
H(7)	6808	5093	9815	31
H(12)	6015	1939	4049	29
H(13)	4929	928	4485	33
H(16)	4289	3874	4917	23
H(18)	2931	4155	4950	40
H(19)	1464	4459	5267	48
H(21)	1041	1882	6112	42
H(22)	2505	1583	5818	36
